# Reducing Bias and Quantifying Uncertainty in Fluorescence Produced by PCR

**DOI:** 10.1007/s11538-023-01182-z

**Published:** 2023-08-14

**Authors:** Robert F. DeJaco, Matthew J. Roberts, Erica L. Romsos, Peter M. Vallone, Anthony J. Kearsley

**Affiliations:** 1 Applied and Computational Mathematics Division, National Institute of Standards and Technology, 100 Bureau Dr., MS 8910, Gaithersburg, MD 20899-8910 USA; 2 Department of Chemistry and Biochemistry, University of Maryland, 8051 Regents Dr., College Park, MD 20742-4454 USA; 3 Cost Analysis and Research Division, Institute for Defense Analyses, 730 E. Glebe Rd., Alexandria, VA 22305-3086 USA; 4 Biomolecular Measurement Division, National Institute of Standards and Technology, 100 Bureau Dr., MS 8314, Gaithersburg, MD 20899-8314 USA

**Keywords:** Real-time polymerase chain reaction, Stochastic branching process, Uncertainty quantification

## Abstract

We present a new approach for relating nucleic-acid content to fluorescence in a real-time Polymerase Chain Reaction (PCR) assay. By coupling a two-type branching process for PCR with a fluorescence analog of Beer’s Law, the approach reduces bias and quantifies uncertainty in fluorescence. As the two-type branching process distinguishes between complementary strands of DNA, it allows for a stoichiometric description of reactions between fluorescent probes and DNA and can capture the initial conditions encountered in assays targeting RNA. Analysis of the expected copy-number identifies additional dynamics that occur at short times (or, equivalently, low cycle numbers), while investigation of the variance reveals the contributions from liquid volume transfer, imperfect amplification, and strand-specific amplification (i.e., if one strand is synthesized more efficiently than its complement). Linking the branching process to fluorescence by the Beer’s Law analog allows for an *a priori* description of background fluorescence. It also enables uncertainty quantification (UQ) in fluorescence which, in turn, leads to analytical relationships between amplification efficiency (probability) and limit of detection. This work sets the stage for UQ-PCR, where both the input copy-number and its uncertainty are quantified from fluorescence kinetics.

**Supplementary Information:**

The online version contains supplementary material available at 10.1007/s11538-023-01182-z.

## Introduction

Polymerase Chain Reaction (PCR) is a hallmark of molecular biology and applied genetics. When the dynamics of PCR are monitored by a fluorescent probe, the initial amount of target sequence can be quantified (qPCR) by a computational algorithm equipped with a mathematical model and a set of control experiments (Ruijter et al. 2009; Lievens et al. 2012; Zhao and Fernald 2005; Peirson et al. 2003; Tichopad et al. 2003; Boggy and Woolf 2010; Guescini et al. 2008; Ruijter et al. 2013). Quantification by PCR is routinely exploited in many applications, including analysis of forensic evidence (Nicklas and Buel 2003; Bauer 2007), monitoring of food safety (Elizaquível et al. 2014), and clinical diagnostics (Kaltenboeck and Wang 2005; Bustin et al. 2021).

The accuracy and precision of the quantification process is limited by the mathematical model relating DNA content to fluorescence. Current models possess subjective and systematic bias and do not account for the uncertainty in fluorescence that arises from imperfect amplification and pipetting errors.

Systematic bias originates from assuming that the initial DNA type is double-stranded and that the fluorescence increases each time *either* complementary strand is replicated. The former is obviously not true when the initial DNA is produced by reverse-transcription of single-stranded RNA (RT, as in RT-qPCR), as only *one* of the complementary DNA strands is present at the beginning of PCR. The second statement is not true for common probes that possess a fluorophore covalently attached to an oligonucleotide. Since the oligonucleotide only hybridizes to *one* of the complementary strands, the fluorescence only increases when *one* of the complementary strands is replicated. The second assumption also does not appear to be true for fluorescent dyes that bind non-specifically to DNA, as the amount of dye bound to each DNA strand depends on the amount of DNA in solution.

The impact of several of these assumptions was assessed by Ruijter et al. (2014). The authors found that, depending on the initial DNA type and fluorescent probe chemistry, the background-subtracted fluorescence could differ by up to a factor of 2 in the exponential phase. However, the authors’ analysis was rooted in the assumption of perfect amplification. They also noted that what actually occurs during the first few cycles of PCR is unknown.

Another source of systematic bias in the mathematical description of fluorescence arises when the initial DNA content is very small. Current approaches are deterministic and do not take into account the fact that the number of DNA strands is an integer. While the kinetics of PCR have been investigated in the framework of stochastic branching processes (Nedelman et al. 1992; Sun 1995; Weiss and von Haeseler 1995; Stolovitzky and Cecchi 1996; Jacob and Peccoud 1996b, a), the first of which was published in this journal, such models have not been linked to the fluorescence reported by probes. Like the deterministic approaches described above, these stochastic models neither discriminate between complementary strands nor describe initial conditions encountered in RT-qPCR.

A mathematical model that discriminates between complementary DNA strands can investigate another source of bias: the assumption that the efficiency of synthesis is independent of directionality (i.e., reverse or forward). Since primers are complementary to different ends of the target sequence, and are specifically chosen not to be complementary to each other (i.e., avoiding dimerization), the formation of one primer–target complex may be more efficient than the other. In addition, the yield of the strand whose replication is being monitored by the fluorescent probe may be affected by the monitoring process. These arguments are also supported by the fact that optimal concentrations of each primer can be different (see, for example, Bustin (2004), where the two concentrations differ by a factor of 3).

To address these challenges, we present a two-type stochastic branching process model in Sect. 2.1 that differentiates between complementary strands and amplification probabilities. Analysis of the expected value in Sect. 2.2 identifies a new timescale that is prevalent during the first few cycles. This timescale explains some of the unknown behavior that occurs during the first few cycles of PCR, explaining some of the aforementioned unknown behavior. At short times, there is a lag in exponential growth where the ratio of expected strand counts changes from its initial to critical value. The critical ratio is related to the amplification probability of each complementary strand, being unity when the probabilities are identical. The analysis also demonstrates that the popular parameter describing PCR efficiency (or amplification probability) is really the geometric mean of the efficiencies of both complementary strands.

Quantification by real-time PCR is also limited by a subjective and empirical description of the fluorescence that is not associated with amplification, or the background fluorescence. The description of background fluorescence is usually taken *a posteriori* (i.e., after measurements of fluorescence monitoring amplification). Without a clear connection to the chemical and physical processes occurring in solution, the background fluorescence is often assumed to be a linear function of cycle, or a ‘baseline.’

In Sect. 3.1, we address these concerns by using the fluorescence analog of Beer’s Law to relate fluorescence to the concentration of each fluorescent species. While such expressions have often been used to describe the fluorescence of dyes interacting with known amounts of DNA (Biver et al. 2003, 2005), we are not aware of any adaptation to real-time PCR. We discriminate between the fluorescent species by referring to the form present before PCR as the inactive species and the form activated by PCR as the active species. For hydrolysis probes, we show how the relevant parameters can be extracted from a few control experiments (see Sect. 3.2). In contrast to other approaches, we can quantify the validity of the model of background fluorescence. The relevant contributions can be determined without interrogating or adjusting fluorescence data associated with amplification. We find that the model agrees well with experiment and observe that the incremental increase in fluorescence is not independent of cycle.

A final limitation of real-time PCR is the lack of a mathematical expression relating errors arising from pipetting and imperfect amplification to uncertainty in fluorescence. To quantify the variance in copy number (Peccoud and Jacob 1996), we investigate the stochastic branching process (Sect. 2.3) in a manner similar to previous reports analyzing error in high-throughput sequencing (Kebschull and Zador 2015; Schwabe and Falcke 2022). After validating that the fluorescence parameter (the fluorescence per mole) for each species is approximately constant for each cycle and well (Sect. 3.2), the models for PCR and fluorescence are combined (Sect. 3.3). This yields analytical expressions of the first two central moments of fluorescence in terms of reaction efficiencies, input content, and input type (i.e., double-stranded DNA, forward-stranded RNA, and reverse-stranded RNA).

Together with the parameters determined from experiment, fluorescence curves computed with uncertainty identify regimes under which certain sources of error are more prevalent than others (see Sect. 3.3). When the expected initial-strand-number is sufficiently large, or the cycle number sufficiently small, the error in fluorescence in a specific well is less than the well-to-well variation in expected value. As the initial strand count decreases and the fluorescence rises above initial levels, however, the variance in input copy-number and imperfect amplification become the dominant contributions to error. Finally, in Sect. 3.4, we use the fluorescence model to develop analytical expressions for the limit of detection as a function of amplification efficiency and nucleic acid type. These expressions may be particularly useful for application in epidemic diseases, as false positives or false negatives may be instead termed inconclusive.

## Strand-Specific Branching Process

In this section, we model PCR as a two-type branching-process. The model distinguishes between complementary DNA strands and amplification efficiencies. We then derive analytical expressions relating the first two central-moments of strand counts before PCR to those after each cycle has completed. Compartmentalizing DNA amplification and fluorescence, the linking of the two phenomena is postponed until Sect. 3.

**Fig. 1:**
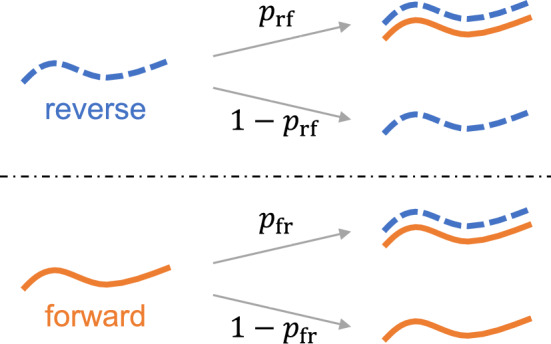
Illustration of strand-specific model of DNA amplification. (top) After each cycle *i*, the outcome of synthesis of a forward strand (orange, solid) from each reverse strand (blue, dashed) present in the previous cycle is modeled as a Bernoulli random-variable with probability of success $p_\text {rf}$. (bottom) Similarly, the outcome of synthesis of a reverse strand (blue, dashed) from each forward strand (orange, solid) present in the previous cycle is modeled as a Bernoulli random-variable with probability of success $p_\text {fr}$ (Color figure online)

### Mathematical Model

PCR consists of a series of *n* cycles, with *n* usually ranging between 35 and 50. Each cycle consists of a melting, annealing, and elongation step to synthesize new DNA from existing DNA (i.e., a chain reaction). A variety of resources on PCR are available online for further information (e.g., National Institutes of Health, National Human Genome Research Institute 2023).

To distinguish between the two complementary strands of DNA, we refer to one as the forward strand and the other as the reverse strand. We let the discrete random variables $X_i$ and $Y_i$ represent the number of forward and reverse strands, respectively, present after $i=0$ to *n* cycles have been completed. We represent the strand counts after completion of *i* cycles as the random vector

$$\begin{aligned} \varvec{U}_i = \begin{pmatrix} X_i \\ Y_i \end{pmatrix}, \end{aligned}$$

and refer to $\varvec{U}_0$ as the initial strand count. However, we distinguish $\varvec{U}_0$ from the strand count *input* to the reaction mixture, denoting the latter by the random vector $$\displaystyle \begin{pmatrix} I_X \\ I_Y \end{pmatrix} $$, where $I_X$ and $I_Y$ are discrete random variables representing the number of forward and reverse strands input, respectively. This is important to distinguish between assays targeting DNA and RNA sequences, as discussed in Sect. 2.1.2.

#### Relationship Between Consecutive Cycles

After completing $i-1$ cycles, the biochemical events occurring during the next cycle involve the attempt to produce one forward strand from each of the $Y_{i-1}$ reverse strands and the attempt to produce one reverse strand from each of the $X_{i-1}$ forward strands (see Fig. 1). The outcome of synthesis of a forward strand from each reverse strand is modeled as a Bernoulli random-variable with probability of success $p_\text {rf}\in (0, 1)$. Similarly, the outcome of synthesis of a reverse strand from each forward strand is modeled as a Bernoulli random-variable with probability of success $p_\text {fr}\in (0, 1)$. (The subscripts rf and fr denote the direction reverse-to-forward and forward-to-reverse, respectively.) This corresponds to the mathematical model

1 $$\begin{aligned} \varvec{U}_i = \varvec{U}_{i-1} + \begin{pmatrix} 0 &{} 1 \\ 1 &{} 0 \end{pmatrix} \varvec{B}\left( \varvec{U}_{i-1}; p_\text {fr}, p_\text {rf}\right) , \end{aligned}$$

after completing *i* cycles, where

$$\begin{aligned} \varvec{B}\left( \varvec{U}_{i-1}; p_\text {fr}, p_\text {rf}\right) :=\begin{bmatrix} \textsf{B}\left( X_{i-1}; p_\text {fr}\right) \\ \textsf{B}\left( Y_{i-1}; p_\text {rf}\right) \end{bmatrix}, \end{aligned}$$

and $\textsf{B}\left( a; b\right)$ denotes a Binomial random-variable of *a* trials with probability of success *b*. All Bernoulli trials, whether associated with the outcome of synthesis of a forward or reverse strand, are taken to be independent.

To compare to previous approaches that do not discriminate between complementary strands and efficiencies, we will use

$$\begin{aligned} N_i = X_i + Y_i \end{aligned}$$

to denote the total number of strands after *i* cycles have been completed. We will see that an appropriate characterization of the average amplification efficiency of both complementary strands is

2 $$\begin{aligned} {\bar{p}} :=\sqrt{p_\text {rf}p_\text {fr}}, \end{aligned}$$

and that an appropriate parameter for the deviation in efficiencies is

3 $$\begin{aligned} R :=\sqrt{\frac{p_\text {rf}}{p_\text {fr}}}. \end{aligned}$$

To avoid changing notation, we will subsequently only investigate $\displaystyle p_\text {fr}= \frac{{\bar{p}}}{R}$ and $p_\text {rf}= {\bar{p}}R$ in terms of ${\bar{p}}$ and *R*, so that

$$\begin{aligned} \varvec{B}\left( \varvec{U}_{i-1}; p_\text {fr}, p_\text {rf}\right) = \varvec{B}\left( \varvec{U}_{i-1}; \dfrac{{\bar{p}}}{R}, {\bar{p}}R\right) = \begin{bmatrix} \textsf{B}\left( X_{i-1}; \dfrac{{\bar{p}}}{R}\right) \\ \textsf{B}\left( Y_{i-1}; {\bar{p}}R\right) \end{bmatrix}. \end{aligned}$$

#### Relationship Between Initial and Input Condition

The relationship between the input number $$\displaystyle \begin{pmatrix}I_X \\ I_Y \end{pmatrix}$$ and initial number $\varvec{U}_0$ of strands depends on whether the nucleic acids input to the reaction mixture are forward-stranded RNA (fs-RNA, referred to as Case RF), reverse-stranded RNA (rs-RNA, referred to as Case RR), or double-stranded DNA (ds-DNA, which consists of both fs-DNA and rs-DNA, referred to as Case D). If ds-DNA is generated by transferring rs-DNA and fs-DNA separately into the reaction mixture, $X_0$ and $Y_0$ can be modeled as independent and identically distributed (i.i.d.). Here,

$$\begin{aligned} \textsf{Case}~\textsf{D}: \qquad \varvec{U}_0 = \begin{pmatrix} X_0 \\ Y_0 \end{pmatrix} = \begin{pmatrix} I_X \\ I_Y \end{pmatrix}, \qquad \begin{array}{l} I_X (\text {or } I_Y)\text { represents number } \\ \text { of fs-DNA (or rs-DNA) strands.} \end{array} \end{aligned}$$

When RNA is input to the reaction mixture, on the other hand, the nucleic acids all possess the same strandedness (i.e., they are all fs-RNA or rs-RNA). The RT step yields DNA strands that are complementary to the RNA. As all RNA strands are fs-RNA (or rs-RNA), the RT step yields rs-DNA (or fs-DNA). Modeling the outcome of synthesis of each cDNA from each RNA strand as a Bernoulli random-variable with probability of success $r\in (0, 1)$, $\varvec{U}_0$ is related to $$\displaystyle \begin{pmatrix}I_X \\ I_Y\end{pmatrix}$$ via either

$$\begin{aligned} \textsf{Case}~\textsf{RF}: \qquad \varvec{U}_0 = \begin{pmatrix} X_0 \\ Y_0 \end{pmatrix} = \begin{bmatrix} 0 \\ \textsf{B}\left( I_X; r\right) \end{bmatrix}, \qquad \begin{array}{l} I_X\text { represents number of}\\ \text { fs-RNA strands},\, I_Y = 0, \end{array} \end{aligned}$$

or

$$\begin{aligned} \textsf{Case}~\textsf{RR}: \qquad \varvec{U}_0 = \begin{pmatrix} X_0 \\ Y_0 \end{pmatrix} = \begin{bmatrix} \textsf{B}\left( I_Y; r\right) \\ 0\end{bmatrix}, \qquad \begin{array}{l} I_Y \text { represents number of} \\ \text { rs-RNA strands},\,I_X = 0. \end{array} \end{aligned}$$

In comparison to the conventional PCR amplification efficiency $p:=p_\text {fr}=p_\text {rf}$ when $R=1$, which is usually between 0.8 and 0.99, the RT efficiency *r* can adopt a relatively large range of values (Bustin et al. 2015; Schwaber et al. 2019).

The nucleic acids are input to the reaction mixture by transferring liquids from one container to another using a pipette. Since the process of transferring such liquids is independent of the type of nucleic acids (i.e., independent of whether they are fs-RNA, rs-RNA, fs-DNA, or rs-DNA), the number of strands of each type input to the reaction mixture obey the same distribution. We will let this distribution be obeyed by the discrete random variable *I*. As a result, it follows that

$$\begin{aligned} {\left\{ \begin{array}{ll} I_X, I_Y {\mathop {\sim }\limits ^{\text {i.i.d.}}}I, &{} \textsf{Case}~\textsf{D}, \\ I_X {\mathop {\sim }\limits ^{\text {i.d.}}}I, \quad I_Y = 0, &{} \textsf{Case}~\textsf{RF}, \\ I_X = 0,\quad I_Y {\mathop {\sim }\limits ^{\text {i.d.}}}I, &{} \textsf{Case}~\textsf{RR}, \\ \end{array}\right. } \end{aligned}$$

where i.d. denotes identically distributed.

### Expected Value

In this section, we derive relationships between $\mathbb {E}\left[ I\right]$ and expected copy-numbers after *i* cycles have been completed. After using the total law of expectation to obtain a relationship in expected copies between two sequential cycles, we use induction to generate the desired results. We first investigate the conventional branching process for PCR which results from assuming $R=1$ and does not distinguish between fs-DNA and rs-DNA. Subsequently, we consider the two-type branching process (1) where *R* may not be 1 and fs-DNA is distinguished from rs-DNA.

#### Conventional Branching Process

The conventional branching process model for PCR occurs when $R=1$, implying from (3) that $p_\text {rf}=p_\text {fr}=:p$. In this case, the system (1) can be summed to yield

4 $$\begin{aligned} N_i = N_{i-1} + \textsf{B}\left( N_{i-1}; p\right) , \end{aligned}$$

as has been investigated elsewhere (Nedelman et al. 1992; Sun 1995; Weiss and von Haeseler 1995; Peccoud and Jacob 1996; Jacob and Peccoud 1996b, a; Stolovitzky and Cecchi 1996). Using the law of total expectation and (4), one finds that

5 $$\begin{aligned} \mathbb {E}\left[ N_i\right] = \mathbb {E}\left[ \mathbb {E}\left[ N_i \mid N_{i-1}\right] \right] = \mathbb {E}\left[ N_{i-1}\right] \left( 1 + p\right) , \end{aligned}$$

for any two consecutive cycles. From induction, it follows that (5) is identical to

6 $$\begin{aligned} \mathbb {E}\left[ N_i\right] = \mathbb {E}\left[ N_0\right] (1 + p)^i, \end{aligned}$$

as reported elsewhere (Nedelman et al. 1992; Sun 1995; Weiss and von Haeseler 1995; Peccoud and Jacob 1996; Jacob and Peccoud 1996b, a; Stolovitzky and Cecchi 1996).

#### Strand-Specific Branching Process

Below, we develop relationships between $\mathbb {E}\left[ I\right]$ and each $\mathbb {E}\left[ \varvec{U}_i\right]$ for the more general case of (1) where forward strands are distinguished from reverse strands and *R* may not be 1. The law of total expectation and (1) lead to the relation

7 $$\begin{aligned} \mathbb {E}\left[ \varvec{U}_i\right] =&\mathbb {E}\left[ \mathbb {E}\left[ \varvec{U}_i \mid \varvec{U}_{i-1}\right] \right] = \mathbb {E}\left\{ \varvec{U}_{i-1} + \begin{pmatrix}0 &{} 1\\ 1 &{} 0 \end{pmatrix}\mathbb {E}\left[ \varvec{B}\left( \varvec{U}_{i-1}; \dfrac{{\bar{p}}}{R}, {\bar{p}}R\right) \Bigm \vert \varvec{U}_{i-1}\right] \right\} \nonumber \\ =&\varvec{A} \mathbb {E}\left[ \varvec{U}_{i-1}\right] = \varvec{A}^i\mathbb {E}\left[ \varvec{U}_0\right] , \end{aligned}$$

where $\varvec{A}$ is defined as

$$\begin{aligned} \varvec{A} = \begin{pmatrix} 1 &{} {\bar{p}}R \\ {\bar{p}}/R &{} 1 \end{pmatrix}, \end{aligned}$$

and the last step follows from induction. The relationship between $\mathbb {E}\left[ \varvec{U}_0\right]$ in (7) depends on $\mathbb {E}\left[ I\right]$ through

8 $$\begin{aligned} \mathbb {E}\left[ \varvec{U}_0\right] = \left\{ \begin{aligned} \begin{pmatrix} \mathbb {E}\left[ I_X\right] \\ \mathbb {E}\left[ I_Y\right] \end{pmatrix} =&\begin{pmatrix} 1 \\ 1 \end{pmatrix}\mathbb {E}\left[ I\right] ,&\textsf{Case}&\mathsf { \, D};\\ \begin{pmatrix} 0 \\ r\mathbb {E}\left[ I_X\right] \end{pmatrix} =&\begin{pmatrix} 0 \\ 1 \end{pmatrix} r\mathbb {E}\left[ I\right] ,&\textsf{Case}&\mathsf { \, RF}; \\ \begin{pmatrix} r\mathbb {E}\left[ I_Y\right] \\ 0 \end{pmatrix} =&\begin{pmatrix}1 \\ 0 \end{pmatrix} r\mathbb {E}\left[ I\right] ,&\textsf{Case}&\mathsf { \,RR}; \end{aligned}\right. \end{aligned}$$

which follows from $\mathbb {E}\left[ \varvec{U}_0\right] = \mathbb {E}\left[ \mathbb {E}\left[ \varvec{U}_0 \mid I_X, I_Y\right] \right]$ and the case-by-case relationships presented in Sect. 2.1.2.

We will see that the matrix $\varvec{A}$ plays a central role in dynamics of the first two central-moments of strand counts. $\varvec{A}$ has two distinct eigenvalues,

$$\begin{aligned} \lambda _1&:=1 + {\bar{p}}, \\ \lambda _2&:=1 - {\bar{p}}, \end{aligned}$$

and can be decomposed as

9 $$\begin{aligned} \varvec{A} = \varvec{X}\varvec{\Lambda } \varvec{Z} = \sum _{j=1}^2\lambda _j \varvec{x}_j\varvec{z}_j^\top , \end{aligned}$$

where

$$\begin{aligned} \varvec{X} :=\begin{pmatrix}\varvec{x}_1&\varvec{x}_2\end{pmatrix} :=\frac{1}{\sqrt{2}}\begin{pmatrix}R &{} R \\ 1 &{} -1\end{pmatrix}, \qquad \varvec{\Lambda } :=\begin{pmatrix} \lambda _1 &{} 0 \\ 0 &{} \lambda _2 \end{pmatrix}, \end{aligned}$$

and

$$\begin{aligned} \varvec{Z} :=\varvec{X}^{-1} = \frac{1}{R\sqrt{2}}\begin{pmatrix}1 &{} R \\ 1 &{} -R\end{pmatrix} =:\begin{pmatrix}\varvec{z}_1^\top \\ \varvec{z}_2^\top \end{pmatrix}. \end{aligned}$$

If $R=1$, the scale factor $\displaystyle \frac{1}{\sqrt{2}}$ in the definition of $\varvec{X}$ implies that

10 $$\begin{aligned} \Vert \varvec{x}_1\Vert = \Vert \varvec{x}_2\Vert = \Vert \varvec{z}_1\Vert = \Vert \varvec{z}_2\Vert =1, \end{aligned}$$

where $\Vert \cdot \Vert$ denotes the Euclidean norm. If $R\ne 1$, on the other hand, no scale factor can be chosen to satisfy (10).

Since $$\varvec{Z}:=\varvec{X}^{-1}$$,

11 $$\begin{aligned} \varvec{x}_i^\top \varvec{z}_j = {\left\{ \begin{array}{ll} 1, &{} \text {if}\; i = j, \\ 0, &{} \text {otherwise}, \end{array}\right. } \end{aligned}$$

for $i,j\in \{1,2\}$, and (after substitution of (9))

12 $$\begin{aligned} \varvec{A}^i = \left( \sum _{j=1}^2 \lambda _j \varvec{x}_j\varvec{z}_j^\top \right) ^i = \sum _{j=1}^2 \lambda _j^i \varvec{x}_j\varvec{z}_j^\top . \end{aligned}$$

Substituting (12) into (7) leads to

13 $$\begin{aligned} \mathbb {E}\left[ \varvec{U}_i\right] =&\varvec{z}_1^\top \mathbb {E}\left[ \varvec{U}_0\right] \lambda _1^i \varvec{x}_1 + \varvec{z}_2^\top \mathbb {E}\left[ \varvec{U}_0\right] \lambda _2^i \varvec{x}_2 \nonumber \\ =&\mathbb {E}\left[ \frac{X_0 + RY_0}{2}\right] \lambda _1^i\begin{pmatrix}1 \\ R^{-1}\end{pmatrix} + \mathbb {E}\left[ \frac{X_0 - RY_0}{2}\right] \lambda _2^i\begin{pmatrix}1 \\ -R^{-1}\end{pmatrix}, \end{aligned}$$

where second expression aids in physical interpretation (below) of Case D, RF, and RR.

Where Equation (6) has $1 + p$, Equation (13) has two values $\lambda _1$ and $\lambda _2$. In the case that $R=1$, $\lambda _1:=1 + {\bar{p}}=1+p$, as is present in (6). The eigenvalue $\lambda _2$ does not have an analog in (6).

The quantities $X_i + RY_i$ and $X_i - RY_i$ in the second expression of (13) arise frequently in the investigation of the first two central-moments of $\varvec{U}_i$. The quantity $X_i + RY_i$ is referred to as the weighted sum of strand counts after *i* cycles have been completed, while $X_i - RY_i$ is referred to as the weighted difference. Multiplying each side of the equation (13) by the row vector $$\displaystyle \begin{pmatrix}1 \\ \pm R\end{pmatrix}^\top $$ demonstrates^1^ that

$$\begin{aligned} \left\{ \begin{aligned} \mathbb {E}\left[ X_i + RY_i\right] =&\mathbb {E}\left[ X_0 + RY_0\right] \lambda _1^i, \\ \mathbb {E}\left[ X_i - RY_i\right] =&\mathbb {E}\left[ X_0 - RY_0\right] \lambda _2^i. \end{aligned} \right. \end{aligned}$$

As $\lambda _1 > 1$, the expected weighted-sum exhibits exponential growth. As $\lambda _2 < 1$, on the other hand, the expected weighted-difference exhibits exponential decay.

The term in (13) associated with $\lambda _2$ is always present when the input is RNA (i.e., for Case RF or RR and any ${\bar{p}}\in (0, 1)$ and $R\in (0, \infty )$), as $\mathbb {E}\left[ X_0\right] \ne R\mathbb {E}\left[ Y_0\right]$. However, it may not be present if the input is DNA, as the term vanishes ($\mathbb {E}\left[ X_0-RY_0\right] = (1-R)\mathbb {E}\left[ I\right]$) for Case D when $R=1$.

**Fig. 2:**
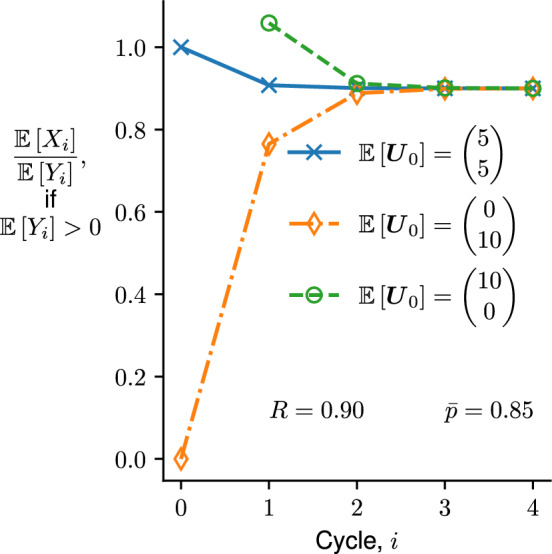
Visualization of convergence of the ratio of expected-forward to expected-reverse strands to *R*. The initial condition $\mathbb {E}\left[ \varvec{U}_0\right]$ is taken from (8) with Case D, RF, or RR. The values of ${\bar{p}}$ and *R* are annotated, while *r* and $\mathbb {E}\left[ I\right]$ are chosen so that $\mathbb {E}\left[ N_0\right] = \mathbb {E}\left[ X_0 + Y_0\right] = 10$

When $\mathbb {E}\left[ X_0\right] \ne R \mathbb {E}\left[ Y_0\right]$, the term involving $\lambda _2^i$ is usually negligible after a few cycles, as ${\bar{p}}$ is usually above 0.8. After the lag time is over (*i* is large enough for $\lambda _2^i$ to be negligible), the *ratio* of expected forward to reverse strand counts reaches its critical value, as

14 $$\begin{aligned} \lim _{i\rightarrow \infty }\frac{\mathbb {E}\left[ X_i\right] }{\mathbb {E}\left[ Y_i\right] } = \lim _{i\rightarrow \infty }\frac{\varvec{e}_1^\top \mathbb {E}\left[ \varvec{U}_i\right] }{\varvec{e}_2^\top \mathbb {E}\left[ \varvec{U}_i\right] } = \frac{\varvec{e}_1^\top \varvec{x}_1}{\varvec{e}_2^\top \varvec{x}_1} = R, \end{aligned}$$

where $$\displaystyle \varvec{e}_1 = \begin{pmatrix}1 \\ 0 \end{pmatrix}$$ and $$\displaystyle \varvec{e}_2=\begin{pmatrix}0 \\ 1\end{pmatrix}$$ are the standard unit vectors. An illustration of the transition of $\displaystyle \frac{\mathbb {E}\left[ X_i\right] }{\mathbb {E}\left[ Y_i\right] }$ (when $\mathbb {E}\left[ Y_i\right] > 0$) to *R* is depicted in Fig. 2.

To compare the two-type branching process to (6), we compute from (13)

$$\begin{aligned} \mathbb {E}\left[ N_i\right]&= \mathbb {E}\left[ X_i + Y_i\right] = \begin{pmatrix}1 \\ 1\end{pmatrix}^\top \mathbb {E}\left[ \varvec{U}_i\right] \nonumber \\&= \varvec{z}_1^\top \mathbb {E}\left[ \varvec{U}_0\right] \lambda _1^i \begin{pmatrix}1 \\ 1\end{pmatrix}^\top \varvec{x}_1 + \varvec{z}_2^\top \mathbb {E}\left[ \varvec{U}_0\right] \lambda _2^i \begin{pmatrix}1 \\ 1\end{pmatrix}^\top \varvec{x}_2 \\&=\mathbb {E}\left[ \frac{X_0 + RY_0}{2}\right] \lambda _1^i\left( \frac{R + 1}{R}\right) + \mathbb {E}\left[ \frac{X_0 - RY_0}{2}\right] \lambda _2^i\left( \frac{R - 1}{R}\right) , \end{aligned}$$

and, for Case D,

15 $$\begin{aligned} \mathbb {E}\left[ N_i\right] = \mathbb {E}\left[ N_0\right] \left[ \frac{\left( R + 1\right) ^2}{4R}\lambda _1^i - \frac{\left( R - 1\right) ^2}{4R}\lambda _2^i\right] , \end{aligned}$$

as (8) implies that $\mathbb {E}\left[ I\right] =\mathbb {E}\left[ X_0\right] =\mathbb {E}\left[ X_0 + Y_0\right] /2 = \mathbb {E}\left[ N_0\right] /2$. Equation (15) demonstrates that the two approaches are equal if $R=1$. Otherwise, the absolute difference between $\mathbb {E}\left[ N_i\right]$ calculated from (6) and (15) increases exponentially with increasing *i*. In the future, determining *R* and using (15) instead of (6) may therefore be important to reduce bias in quantification.

On the other hand, when $R=1$, the expected amount of each strand is eventually independent of the composition at the start of PCR. That is,

16 $$\begin{aligned} \lim _{i\rightarrow \infty }\frac{\mathbb {E}\left[ X_i\right] }{\lambda _1^i} = \lim _{i\rightarrow \infty }\frac{\mathbb {E}\left[ Y_i\right] }{\lambda _1^i} = \lim _{i\rightarrow \infty }\frac{\mathbb {E}\left[ N_i\right] }{2\lambda _1^i} = \frac{\mathbb {E}\left[ N_0\right] }{2}, \qquad \text {if}\; R = 1, \end{aligned}$$

only depends on the expected initial-sum, or $\mathbb {E}\left[ N_0\right]$. This result is in contrast to the report of Ruijter et al. (2014), who used a deterministic model with perfect amplification efficiency. When $R=1$ and the input is RNA, the directionality of RNA (i.e., fs-RNA or rs-RNA) only dictates the characteristic length of the lag time.

### Variance

In this section, we derive the relationship between $\textsf{Var}\left[ I\right]$ and the variance after *i* cycles have been completed. The procedure is similar to the previous section, except the law of total variance is used instead of the law of total expectation.

#### Conventional Branching Process

Using the law of total variance and (4), one obtains

17 $$\begin{aligned} \textsf{Var}\left[ N_i\right] =&\textsf{Var}\left[ \mathbb {E}\left[ N_i \mid N_{i-1}\right] \right] + \mathbb {E}\left[ \textsf{Var}\left[ N_i \mid N_{i-1}\right] \right] \nonumber \\ =&\textsf{Var}\left[ N_{i-1}\right] \left( 1 + p\right) ^2 + \mathbb {E}\left[ N_{i-1}\right] p\left( 1 - p\right) . \end{aligned}$$

Induction can be used to show that (17) is equivalent to

18 $$\begin{aligned} \textsf{Var}\left[ N_i\right] = \textsf{Var}\left[ N_0\right] \left( 1 + p\right) ^{2i} + p\left( 1 - p\right) \left( 1 + p\right) ^{2(i-1)}\sum \limits _{j=0}^{i-1}\frac{\mathbb {E}\left[ N_j\right] }{\left( 1 + p\right) ^{2j}}. \end{aligned}$$

Substitution of (6), simplification of the resultant geometric series, and rearrangement leads to the expression

19 $$\begin{aligned} \textsf{Var}\left[ N_i\right] =&\left\{ \textsf{Var}\left[ N_0\right] + \mathbb {E}\left[ N_0\right] \left( \frac{1 - p}{1 + p}\right) \right\} \left( 1 + p\right) ^{2i} \nonumber \\&-\mathbb {E}\left[ N_0\right] \left( 1 - p\right) \left( 1 + p\right) ^{i-1}. \end{aligned}$$

When $\textsf{Var}\left[ N_0\right] = 0$, Equation (19) becomes identical to what has been reported elsewhere (Sun 1995; Weiss and von Haeseler 1995; Jacob and Peccoud 1996b, a; Stolovitzky and Cecchi 1996) (Nedelman et al. (1992); Peccoud and Jacob (1996) report the leading-order approximation for large *i*). The more general result, Equation (19), demonstrates that $\textsf{Var}\left[ N_0\right]$ can significantly impact $\textsf{Var}\left[ N_i\right]$, as it is part of the dominant term.

The growth in variance with increasing *i* by an exponent twice that of the expected value explains why very large cycles, where the expected copy-number is also very large, are not of interest. It also explains why quantification by real-time PCR is more reproducible than end-point PCR.

#### Strand-Specific Branching Process

The variance–covariance matrix of $\varvec{U}_i$ is defined as

$$\begin{aligned} \textsf{Var}\left[ \varvec{U}_i\right] = \mathbb {E}\left[ \varvec{U}_i \varvec{U}_i^\top \right] - \mathbb {E}\left[ \varvec{U}_i\right] \mathbb {E}\left[ \varvec{U}_i\right] ^\top , \end{aligned}$$

for each *i*. From the law of total variance,

$$\begin{aligned} \textsf{Var}\left[ \varvec{U}_i\right] = \textsf{Var}\left[ \mathbb {E}\left[ \varvec{U}_i \mid \varvec{U}_{i-1}\right] \right] + \mathbb {E}\left[ \textsf{Var}\left[ \varvec{U}_i\mid \varvec{U}_{i-1}\right] \right] . \end{aligned}$$

In a manner similar to (7), it follows that

$$\begin{aligned} \textsf{Var}\left[ \mathbb {E}\left[ \varvec{U}_i \mid \varvec{U}_{i-1}\right] \right] = \textsf{Var}\left[ \varvec{A}\varvec{U}_{i-1}\right] = \varvec{A}\textsf{Var}\left[ \varvec{U}_{i-1}\right] \varvec{A}^\top . \end{aligned}$$

In addition, after substitution of (1), it follows that

$$\begin{aligned} \mathbb {E}\left[ \textsf{Var}\left[ \varvec{U}_i\mid \varvec{U}_{i-1}\right] \right] =&\mathbb {E}\left\{ \textsf{Var}\left[ \begin{pmatrix}0 &{} 1 \\ 1 &{} 0 \end{pmatrix}\varvec{B}\left( \varvec{U}_{i-1}; \dfrac{{\bar{p}}}{R}, {\bar{p}}R\right) \Bigm \vert \varvec{U}_{i-1}\right] \right\} \\ =&\mathbb {E}\left[ \textsf{Var} \left\{ \begin{bmatrix} \textsf{B}\left( Y_{i-1}; {\bar{p}}R\right) \\ \textsf{B}\left( X_{i-1}; \dfrac{{\bar{p}}}{R}\right) \\ \end{bmatrix} \Biggm \vert \varvec{U}_{i-1} \right\} \right] \\ =&\begin{bmatrix} {\bar{p}}R\left( 1 - {\bar{p}}R\right) \mathbb {E}\left[ Y_{i-1}\right] &{} 0 \\ 0 &{} \dfrac{{\bar{p}}}{R}\left( 1 - \dfrac{{\bar{p}}}{R}\right) \mathbb {E}\left[ X_{i-1}\right] \end{bmatrix}. \end{aligned}$$

Combining the two expressions, we obtain the two-type analog of (17),

20 $$\begin{aligned} \textsf{Var}\left[ \varvec{U}_i\right] =&\varvec{A}\textsf{Var}\left[ \varvec{U}_{i-1}\right] \varvec{A}^\top \nonumber \\&+ \begin{bmatrix} {\bar{p}}R\left( 1 - {\bar{p}}R\right) \mathbb {E}\left[ Y_{i-1}\right] &{} 0 \\ 0 &{} \dfrac{{\bar{p}}}{R}\left( 1 - \dfrac{{\bar{p}}}{R}\right) \mathbb {E}\left[ X_{i-1}\right] \end{bmatrix}. \end{aligned}$$

Before using induction to relate $\textsf{Var}\left[ \varvec{U}_i\right]$ to $\textsf{Var}\left[ \varvec{U}_0\right]$, it is useful to simplify (20) by substituting $\mathbb {E}\left[ Y_{i-1}\right] =\varvec{e}_2^\top \mathbb {E}\left[ \varvec{U}_{i-1}\right]$ and $\mathbb {E}\left[ X_{i-1}\right] =\varvec{e}_1^\top \mathbb {E}\left[ \varvec{U}_{i-1}\right]$ with $\mathbb {E}\left[ \varvec{U}_{i-1}\right]$ provided by the first expression of (13). Collecting terms multiplying each eigenvalue, we find that

21 $$\begin{aligned} \textsf{Var}\left[ \varvec{U}_i\right] = \varvec{A} \textsf{Var}\left[ \varvec{U}_{i-1}\right] \varvec{A}^\top + \sum _{\ell =1}^2 \lambda _{\ell }^{i-1}\varvec{K}_{\ell }, \end{aligned}$$

where

22 $$\begin{aligned} \varvec{K}_\ell :=\begin{bmatrix} {\bar{p}}R\left( 1 - {\bar{p}}R\right) \varvec{e}_2^\top \varvec{x}_{\ell } &{} 0 \\ 0 &{} \dfrac{{\bar{p}}}{R}\left( 1 - \dfrac{{\bar{p}}}{R}\right) \varvec{e}_1^\top \varvec{x}_{\ell } \end{bmatrix} \varvec{z}_\ell ^\top \mathbb {E}\left[ \varvec{U}_0\right] . \end{aligned}$$

From induction, it follows that (21) is identical to

23 $$\begin{aligned} \textsf{Var}\left[ \varvec{U}_i\right] = \varvec{A}^i \textsf{Var}\left[ \varvec{U}_{0}\right] \left( \varvec{A}^\top \right) ^i + \sum _{\ell =1}^2\sum _{q=0}^{i-1}\lambda _{\ell }^q \varvec{A}^{i-1-q}\varvec{K}_{\ell } \left( \varvec{A}^\top \right) ^{i-1-q}. \end{aligned}$$

Substitution of (12) and simplification of the resultant geometric series leads to (see Sect. B.1)

24 $$\begin{aligned} \textsf{Var}\left[ \varvec{U}_i\right] =&\sum _{j=1}^2 \sum _{k=1}^2 \left\{ \left[ \nu _{j,k} + \sum _{\ell = 1}^2\eta _{j, k}^\ell \right] \left( \lambda _j\lambda _k\right) ^i - \sum _{\ell =1}^2 \eta _{j,k}^\ell \lambda _{\ell }^i \right\} \varvec{x}_j\varvec{x}_k^\top , \end{aligned}$$

where

25a $$\begin{aligned} \nu _{j,k} :=&\varvec{z}_j^\top \textsf{Var}\left[ \varvec{U}_0\right] \varvec{z}_k, \end{aligned}$$

25b $$\begin{aligned} \eta _{j,k}^{\ell } :=&\frac{\varvec{z}_j^\top \varvec{K}_{\ell }\varvec{z}_k}{\lambda _j\lambda _k - \lambda _\ell }. \end{aligned}$$

In (24), the dependence of $\textsf{Var}\left[ \varvec{U}_i\right]$ on *I* arises through $\textsf{Var}\left[ \varvec{U}_0\right]$ in each $\nu _{j,k}$ and $\mathbb {E}\left[ \varvec{U}_0\right]$ in each $\eta _{j,k}^\ell$ (through (22)). While the dependence of $\mathbb {E}\left[ \varvec{U}_0\right]$ on $\mathbb {E}\left[ I\right]$ is given in (8), the relationship between $\textsf{Var}\left[ \varvec{U}_0\right]$ and $\textsf{Var}\left[ I\right]$ for Case D, RF, or RR is given by

26a $$\begin{aligned} \textsf{Var}\left[ \varvec{U}_0\right]&= \left\{ \begin{aligned}\begin{pmatrix} \textsf{Var}\left[ I_X\right] &{}{} 0 \\ 0 &{}{} \textsf{Var}\left[ I_Y\right] \end{pmatrix} =&\begin{pmatrix} 1 &{}{} 0 \\ 0 &{}{} 1 \end{pmatrix}\textsf{Var}\left[ I\right] ,&{}\textsf{Case}&\mathsf {\, D}; \\ \begin{pmatrix} 0 &{}{} 0 \\ 0 &{}{} \textsf{Var}\left[ \textsf{B}\left( I_X; r\right) \right] \end{pmatrix} =&\begin{pmatrix} 0 &{}{} 0 \\ 0 &{}{} 1 \end{pmatrix} \textsf{Var}\left[ \textsf{B}\left( I; r\right) \right] ,&\textsf{Case}&\mathsf {\; RF}; \\ \begin{pmatrix} \textsf{Var}\left[ \textsf{B}\left( I_Y; r\right) \right] &{}{} 0 \\ 0 &{}{} 0 \end{pmatrix} =&\begin{pmatrix} 1 &{}{} 0 \\ 0 &{}{} 0 \end{pmatrix} \textsf{Var}\left[ \textsf{B}\left( I; r\right) \right] ,&\textsf{Case}&\mathsf {\,RR}, \end{aligned}\right. \end{aligned}$$

26b $$\begin{aligned} \textsf{Var}\left[ \textsf{B}\left( I; r\right) \right] =&\textsf{Var}\left[ \mathbb {E}\left[ \textsf{B}\left( I; r\right) \mid I\right] \right] + \mathbb {E}\left[ \textsf{Var}\left[ \textsf{B}\left( I; r\right) \mid I\right] \right] \nonumber \\ =&\textsf{Var}\left[ I\right] r^2 + r\left( 1 - r\right) \mathbb {E}\left[ I\right] \nonumber \\ =&\textsf{Var}\left[ \textsf{B}\left( I_X; r\right) \right] = \textsf{Var}\left[ \textsf{B}\left( I_Y; r\right) \right] , \end{aligned}$$

where (26b) utilizes the law of total variance. Since $X_0$ and $Y_0$ are independent for any case, $\textsf{Cov}\left[ X_0,Y_0\right] = 0$. As such, we will often use the substitution

27 $$\begin{aligned} \textsf{Var}\left[ \varvec{U}_0\right] = \begin{pmatrix} \textsf{Var}\left[ X_0\right] &{} 0 \\ 0 &{} \textsf{Var}\left[ Y_0\right] \end{pmatrix} \end{aligned}$$

without specifying a particular case.

In contrast to (19), which only has terms proportional to $(1 + p)^{2i}$ and $(1 + p)^{i-1}$, Equation (24) has terms proportional to $\lambda _1^{2i}> \lambda _1^i> (\lambda _1\lambda _2)^i> \lambda _2^i > \lambda _2^{2i}$ (if $i > 0$). While physical interpretation of all terms can be complicated, it is useful to examine the leading-order expression as $i\rightarrow \infty$,

28 $$\begin{aligned} \textsf{Var}\left[ \varvec{U}_i\right] = \left( \nu _{1,1} + \eta _{1,1}^{(1)} + \eta _{1,1}^{(2)}\right) \lambda _1^{2i}\varvec{x}_1\varvec{x}_1^\top + \begin{pmatrix}1 &{} 1 \\ 1 &{} 1 \end{pmatrix}O\left( \lambda _1^i\right) , \end{aligned}$$

and compute the terms explicitly as

29a $$\begin{aligned} \nu _{1,1} =&\textsf{Var}\left[ \frac{X_0 + RY_0}{2}\right] \frac{2}{R^2}, \end{aligned}$$

29b $$\begin{aligned} \eta _{1,1}^{(1)} =&\mathbb {E}\left[ \frac{X_0 + RY_0}{2}\right] \left( \frac{\lambda _2}{\lambda _1}\right) \frac{R + 1}{2R^2}, \end{aligned}$$

29c $$\begin{aligned} \eta _{1,1}^{(2)} =&\mathbb {E}\left[ \frac{X_0 - RY_0}{2}\right] \left( \frac{{\bar{p}}\lambda _1}{\lambda _1^2 - \lambda _2}\right) \frac{R - 1}{2R^2}, \end{aligned}$$

where we have used (27). Note that the moments of $(X_0 \pm RY_0)/2$ arise in (28) as they did in (13). The term $\nu _{1,1}$ represents the contribution from the variance at the start of PCR. The term $\eta _{1,1}^{(1)}$ accounts for the variance due to imperfect amplification, as ${\bar{p}}\approx 1$ implies that $\lambda _2 \approx 0 \approx \eta _{1,1}^{(1)}$. If $R=1$,

$$\begin{aligned} \eta _{1,1}^{(1)} = \frac{\mathbb {E}\left[ N_0\right] }{2}\left( \frac{ 1 - p}{1 + p}\right) , \end{aligned}$$

one-half the second term in brackets of (19). The third term $\eta _{1,1}^{(2)}$ does not have a counterpart in (19). It accounts for differences in strand-specific amplification, as it vanishes when $R\rightarrow 1$.

The expressions developed for expected value and variance can be used to produce expressions in the squared coefficients of variation, defined as

30 $$\begin{aligned} \textsf{CV}\left[ \varvec{U}_i\right] ^2 = \textsf{Var}\left[ \varvec{U}_i\right] \oslash \mathbb {E}\left[ \varvec{U}_i\right] \mathbb {E}\left[ \varvec{U}_i\right] ^\top , \end{aligned}$$

where $\oslash$ denotes element-wise division. Substitution of (13) and (28) into (30) yields

31 $$\begin{aligned} \textsf{CV}\left[ \varvec{U}_i\right] ^2 = \begin{pmatrix} 1 &{} 1 \\ 1 &{} 1 \end{pmatrix} \left\{ \frac{\nu _{1,1} + \eta _{1,1}^{(1)} + \eta _{1,1}^{(2)}}{\left( \varvec{z}_1^\top \mathbb {E}\left[ \varvec{U}_0\right] \right) ^2} + O\left( \lambda _1^{-i}\right) \right\} . \end{aligned}$$

As the term $O\left( \lambda _1^{-i}\right)$ rapidly approaches zero with increasing *i*, and $\lambda _1$ is usually more than 1.8, the dominant term in (31) is a useful approximation. The dominant term is independent of *i* and is therefore a very practical tool for estimating the error present in PCR. As such, it is useful to express (31) as

32 $$\begin{aligned} \varvec{e}_j^\top \textsf{CV}\left[ \varvec{U}_i\right] ^2\varvec{e}_k \sim&\textsf{CV}\left[ X_0 + RY_0\right] ^2 + \frac{1}{\mathbb {E}\left[ X_0 + RY_0\right] }\left( \frac{\lambda _2}{\lambda _1}\right) \frac{R + 1}{2} \nonumber \\&+ \frac{\mathbb {E}\left[ X_0 - RY_0\right] }{\mathbb {E}\left[ X_0 + RY_0\right] ^2}\left( \frac{{\bar{p}}\lambda _1}{\lambda _1^2 - \lambda _2}\right) \frac{R - 1}{2} \nonumber \\ =&\alpha \textsf{CV}\left[ I\right] ^2 + \frac{\beta }{\mathbb {E}\left[ I\right] }, \end{aligned}$$

for any $j,k\in \{1, 2\}$, where (27) is used in the first step and the second step follows from simplification with (8) and (26). The quantities $\alpha$ and $\beta$, defined as

33a $$\begin{aligned} \alpha&=\left\{ \begin{aligned} \dfrac{R^2 + 1}{\left( R + 1\right) ^2},{} & {} {} \textsf{Case }&\mathsf {\,D}; \\ 1,{} & {} {} \textsf {Case}&\mathsf {\, RF} \text{ or } \textsf {RR}; \end{aligned} \right. \end{aligned}$$

33b $$\begin{aligned} \beta&=\left\{ \begin{aligned} \left( \dfrac{\lambda _2}{\lambda _1}\right) \dfrac{1}{2} - \dfrac{{\bar{p}}\lambda _1}{\lambda _1^2 - \lambda _2}\left( \dfrac{R - 1}{R + 1}\right) ^2\dfrac{1}{2},{} & {} \textsf {Case}&\mathsf {\, D}; \\ \dfrac{1 - r}{r} + \left( \dfrac{\lambda _2}{\lambda _1}\right) \dfrac{R + 1}{2Rr} - \left( \dfrac{{\bar{p}}\lambda _1}{\lambda _1^2 - \lambda _2}\right) \dfrac{R - 1}{2Rr},{} & {} \textsf {Case}&\mathsf {\, RF}; \\ \dfrac{1 - r}{r} + \left( \dfrac{\lambda _2}{\lambda _1}\right) \dfrac{R + 1}{2r} + \left( \dfrac{{\bar{p}}\lambda _1}{\lambda _1^2 - \lambda _2}\right) \dfrac{R - 1}{2r},{} & {} \textsf {Case}&\mathsf {\, RR}; \end{aligned}\right. \end{aligned}$$

relate the reaction efficiencies to the coefficient of variation, as $\alpha =\alpha \left( R\right)$ and $\beta = \beta \left( R, {\bar{p}}, r\right)$. In Sect. 3.4, we will see that (32) also plays a central role in the limit of detection.

## Fluorescence Dynamics

### Mathematical Model

To adapt the approach to the fluorescence measured in real-time PCR, it is necessary to relate the DNA content in solution to the monitoring chemistry. When a fluorescent probe is used to monitor the kinetics of PCR, the inactive and active probe species usually make significant contributions to fluorescence. With these two fluorescent species, the fluorescence analog of Beer’s Law is

34 $$\begin{aligned} F_{i,w} = f_{i,w}^{-}C_{i,w}^{-} + f^{+}_{i,w} C_{i,w}^{+}, \end{aligned}$$

after^2^ each cycle $i=1$ to *n* and for each well $w=1$ to *m*. Here, $F_{i,w}$ is the fluorescence measured, $f_{i,w}^{-}$ (or $f_{i,w}^{+}$) is a constant representing the fluorescence per mole of inactive (or active) probe, and $C_{i,w}^{-}$ (or $C_{i,w}^{+}$) is the molar concentration of inactive (or active) probes. Each molar fluorescence, $f_{i,w}^{-}$ or $f_{i,w}^{+}$ (also denoted as $f_{i,w}^\pm$), may depend on *i* due to photobleaching. Each may also depend on *w* due to spatial variation in electronics and temperature. The terms $F_{i,w}$, $C_{i,w}^{-}$, and $C_{i,w}^{+}$ are random variables through their dependence on DNA content (see below).

**Fig. 3:**
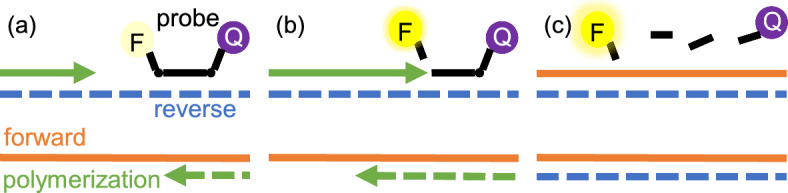
Illustration of relationship between successful DNA replication and changes in fluorescence associated with a hydrolysis probe binding to the reverse strand. (a) As polymerization begins, the hydrolysis probe binds to the reverse strand (blue, dashed line). The probe is in its inactive state, where fluorescence emitted by the fluorophore (‘F’) is quenched by the quencher (‘Q’) in close proximity. (b) As polymerization (green arrow) reaches the location of the probe, the probe is hydrolyzed. (c) After successful production of a forward strand (orange, solid line) from a reverse strand, the fluorophore is activated, as it is no longer in close proximity to the quencher. When a reverse strand is produced from a forward strand, however, the fluorescence does not change (Color figure online)

Assuming that probe is not degraded during cycling,

35 $$\begin{aligned} C = C_{i,w}^{-} + C_{i,w}^{+} \end{aligned}$$

for all *i* and *w*, where *C* is a known constant representing the total concentration of probe in solution. As DNA is replicated, inactive probe is converted to active probe in a manner that depends on the reaction stochiometry. For hydrolysis probes, an inactive probe is activated, or hydrolyzed, each time *one* of the complementary strands is replicated. Without loss of generality, we consider the case where the hydrolysis probe binds to the reverse strand (see Fig. 3). After completing *i* PCR cycles, the concentration of active probe in each well *w* is then

36 $$\begin{aligned} C_{i,w}^{+} = \frac{\Delta X_{i,w}}{\mathcal {V}\textsf {N}_\textsf {a}}, \end{aligned}$$

where $\mathcal {V}$ and $\textsf {N}_\textsf {a}$ are constants representing the volume of solution and Avogadro’s number, respectively, and $\Delta X_{i,w}:=X_{i,w} - X_{0,w}$. We assume for each $i=0$ to *n* that $X_{i,1},\ldots , X_{i,m}$ are independent and distributed identically to $X_i$, and similarly that $Y_{i,1},\ldots , Y_{i,m}$ are independent and distributed identically to $Y_i$. By combining (34), (35), and (36), the fluorescence model becomes

37 $$\begin{aligned} F_{i,w} = b_{i,w} + d_{i,w}\Delta X_{i,w}, \end{aligned}$$

where

38 $$\begin{aligned} b_{i,w} :=f_{i,w}^{-}C \end{aligned}$$

represents the contribution of background fluorescence, and

39 $$\begin{aligned} d_{i,w} :=\frac{f_{i,w}^{+} - f_{i,w}^{-}}{\mathcal {V}\textsf {N}_\textsf {a}} \end{aligned}$$

represents the increase in fluorescence per synthesis of forward strand.

From (37), the first two central-moments of $F_{i,w}$ are

40a $$\begin{aligned} \mathbb {E}\left[ F_{i,w}\right]&= b_{i,w} + d_{i,w}\mathbb {E}\left[ \Delta X_i\right] , \end{aligned}$$

40b $$\begin{aligned} \textsf{Var}\left[ F_{i,w}\right]&= d_{i,w}^2\textsf{Var}\left[ \Delta X_i\right] , \end{aligned}$$

where $\Delta X_i :=X_i - X_0$. Here, $\mathbb {E}\left[ \Delta X_i\right]$ can be viewed as a function of ${\bar{p}}$, *R*, *r*, and $\mathbb {E}\left[ I\right]$ by substituting (8) into (13). Similarly, $\textsf{Var}\left[ X_i\right]$ can be viewed as a function of ${\bar{p}}$, *R*, *r*, $\mathbb {E}\left[ I\right]$, and $\textsf{Var}\left[ I\right]$ by substituting (8) and (26) into (24). While $\textsf{Var}\left[ X_0\right]$ is a function of *r*, $\mathbb {E}\left[ I\right]$, and $\textsf{Var}\left[ I\right]$ through (26), an expression for $\textsf{Cov}\left[ X_i, X_0\right]$ is needed for (40b). The methods described in Sect. 2.2 and 2.3 can be readily adapted to the cross-covariance matrix (see Sect. B.2) to obtain

41 $$\begin{aligned} \textsf{Cov}\left[ X_i, X_0\right] = \frac{\textsf{Var}\left[ X_0\right] }{2}\left( \lambda _1^i + \lambda _2^i\right) . \end{aligned}$$

From (13), (28), and (41), it follows that (40) can be expressed as

42 $$\begin{aligned} \mathbb {E}\left[ F_{i,w}\right] = \left( \frac{d_{i,w}}{2}\right) \mathbb {E}\left[ X_0 + RY_0\right] \lambda _1^i + O(1), \end{aligned}$$

and

43 $$\begin{aligned} \textsf{Var}\left[ F_{i,w}\right] = \frac{d_{i,w}^2}{4}&\left\{ \textsf{Var}\left[ X_0 + RY_0\right] + \mathbb {E}\left[ X_0 + RY_0\right] \left( \frac{\lambda _2}{\lambda _1}\right) \frac{R + 1}{2} \right. \nonumber \\&\left. + \mathbb {E}\left[ X_0 - RY_0\right] \left( \frac{{\bar{p}}\lambda _1}{\lambda _1^2 - \lambda _2}\right) \frac{R - 1}{2} \right\} \lambda _1^{2i} + O\left( \lambda _1^i\right) , \end{aligned}$$

as $i\rightarrow \infty$. As the dominant term of $\textsf{Var}\left[ F_{i,w}\right]$ is proportional to $\lambda _1^{2i} \gg \lambda _1^i$, it is independent of $\textsf{Cov}\left[ X_i, X_0\right] = O\left( \lambda _1^i\right)$. Instead, the dominant term of $\textsf{Var}\left[ F_{i,w}\right]$ arises from $\textsf{Var}\left[ X_i\right]$ as in (28). As in (32), Equations (42) and (43) imply

44 $$\begin{aligned} \textsf{CV}\left[ F_{i,w}\right] ^2 = \alpha \textsf{CV}\left[ I\right] ^2 + \frac{\beta }{\mathbb {E}\left[ I\right] } + O\left( \lambda _1^{-i}\right) , \end{aligned}$$

where $\alpha =\alpha \left( R\right)$ and $\beta =\beta \left( R, {\bar{p}}, r\right)$ were defined in (33) for Case D, RF, and RR.

In contrast to other models (e.g., Ruijter et al. 2013, 2014), the fluorescence model (37) is consistent with stoichiometric reactions involving hydrolysis probes and DNA polymerase. By using the fluorescence analog of Beer’s Law, it provides a physical basis for description of the background fluorescence $b_{i,w}$. Finally, unlike conventional approaches, the more mechanistic model demonstrates that $d_{i,w}$ may depend on cycle *i*.

When $R\ne 1$, *R* complicates the relationship between DNA content and fluorescence. However, for Case RR, Equations (8), (13), and (40a) lead to

$$\begin{aligned} \mathbb {E}\left[ F_{i,w}\right] = b_{i,w} + \left( \frac{rd_{i,w}}{2}\right) \mathbb {E}\left[ I\right] \left( \lambda _1^i + \lambda _2^i - 2\right) , \qquad \textsf{Case}~\textsf{RR}, \end{aligned}$$

which is independent of *R*. Since the choice of forward and reverse strands was arbitrary, this demonstrates that the monitoring probe can be chosen so that $\mathbb {E}\left[ F_{i,w}\right]$ is independent of *R*, and may be a useful design-rule for RT-qPCR assays.

### Extraction of Molar Fluorescence

In real-time PCR, control experiments containing all reagents except nucleic acid template are often performed to check for contamination. In this section, we will show how they can also be used to calculate $f_{i,w}^\pm$.

Since template is not present (i.e., $\varvec{U}_0= 0$), amplification does not occur and the probe cannot be activated. Equation (37) with (38) becomes

45 $$\begin{aligned} F_{i,w} = f_{i,w}^{-}C, \end{aligned}$$

where $F_{i,w}$ is instead deterministic. After filling *m* wells of a PCR plate with inactive probe at known *C* (and appropriate solvation environment) and measuring $F_{i,w}$ after each $i \ge 1$, $f_{i,w}^{-}$ can be calculated pointwise from (45) by division. However, to get a more realistic estimate of each $f_{i,w}^{-}$, the experiment can be repeated with *q* different plates having a total probe concentration $C^1< \cdots < C^q$ in all *m* wells. With

$$\begin{aligned} \varvec{C} :=\left( C^1,\ldots ,C^q\right) ^\top , \end{aligned}$$

these experiments yield the measurements

$$\begin{aligned} \varvec{F}_{i,w} :=\left( F_{i,w}^1, \ldots , F_{i,w}^q\right) ^\top \end{aligned}$$

for each cycle *i* and well *w*. Under the assumption that $\displaystyle \frac{F^j_{i,w}}{C^j}$ for $j=1$ to *q* are i.i.d. to a normal distribution, we can compute $f_{i,w}^{-}$ via

46 $$\begin{aligned} f_{i,w}^{\pm } = \,{\text {*}}{arg min}_f\, \Vert \varvec{F}_{i,w} - f \varvec{C}\Vert ^2 = \frac{\varvec{F}_{i,w}^\top \varvec{C}}{\varvec{C}^\top \varvec{C}}. \end{aligned}$$

The same procedure can be used to calculate $f_{i,w}^{+}$ after performing the identical experiments with active probe instead of inactive probe. The standard deviation in $f_{i,w}^\pm$ can be estimated pointwise by

47 $$\begin{aligned} \sigma _{i,w}^\pm = \frac{\Vert \varvec{F}_{i,w} - f_{i,w}^{\pm }\varvec{C}\Vert }{\sqrt{q - 1}}. \end{aligned}$$

Using hydrolysis probes, we selected the fluorophore (‘F’ in Fig. 3) to represent the active probe. We used $q=4$ concentrations for the active probe, and $q=3$ concentrations for the inactive probe. Additional details of the experimental procedure can be found in Appendix A. Visual comparisons between the model and experimental data can be found in Figs. S1 to S96 in the Supplementary Information (SI). The pointwise values of $f_{i,w}^{\pm }$ and $\sigma _{i,w}^{\pm }$ are tabulated in Tables S1 to S96 of the SI.

**Fig. 4:**
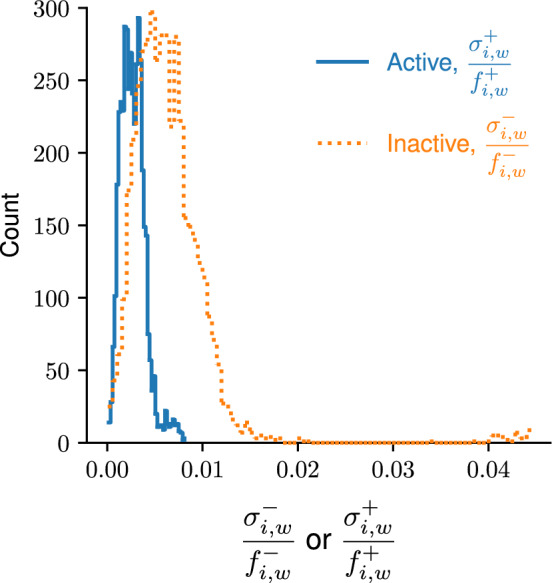
Total number of cycles and wells for all plates (count, vertical axis) possessing values of $\sigma _{i,w}^{\pm }/f_{i,w}^\pm$ in a certain interval (bin, horizontal axis). The bin widths are obtained from the Freedman–Diaconis rule. All counts for $\sigma _{i,w}^{-}/f_{i,w}^{-} > 0.022$ correspond to $w=2$ (well A2, see Fig. S2 and Table S2 in the SI)

To assess the validity of approximating the measured fluorescence by $f_{i,w}^\pm C$ for each cycle *i* and well *w*, the coefficient of variation, or $\displaystyle \frac{\sigma _{i,w}^{\pm }}{f_{i,w}^{\pm }},$ was calculated. A histogram of all values (i.e., all *n* cycles, all *m* wells, and all *q* plates) for each probe species is depicted in Fig. 4. For the active probe, the coefficient of variation is often very small, typically much less than 0.01. For the inactive probe, the coefficient of variation can be larger but is still often less than 0.02. This is an indication that the model is realistic.

**Fig. 5:**
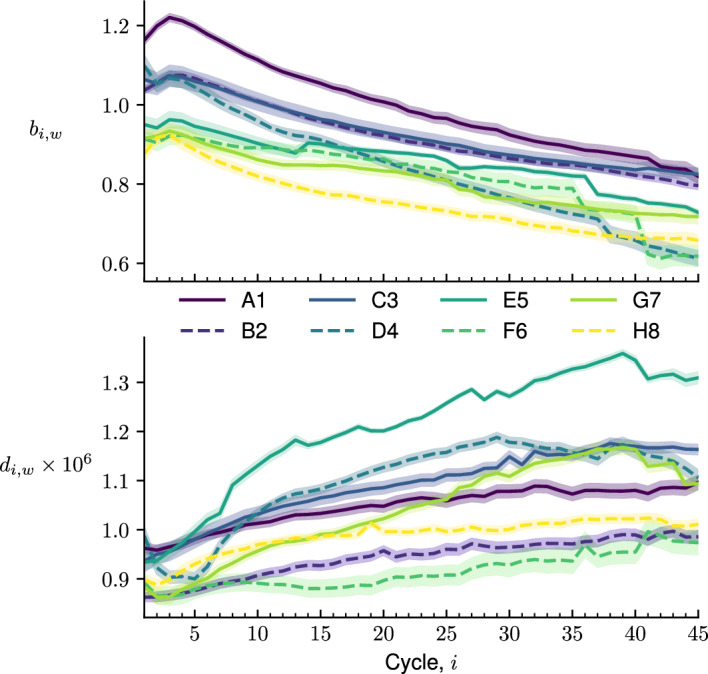
Background (top) and incremental increase (bottom) as a function of cycle *i* for a selection of eight different wells (colors, see legend) with $C=0.125$ pmol/L and $\mathcal {V}=20$ $\mu$L. The partially transparent shadings at each *i* depict an estimate of a 95 % confidence interval (Color figure online)

Having validated the use of the Beer’s Law analog in describing the fluorescence, we used the molar-fluorescence parameters in Fig. 5 to assess how the background $b_{i,w}$ and incremental increase $d_{i,w}$ change with cycle *i* (for a fixed well *w*). Here, we see that $b_{i,w}$ is not a linear function of *i*. In fact, for several *w*, $b_{i,w}$ possesses a maximum in cycle around $i=2$ or $i=3$. The decreases in $b_{i,w}$ with increasing *i* are attributed to result from photobleaching.

Since the fluorescence of an active probe is larger than the fluorescence of an inactive probe, $d_{i,w}$ is positive. Figure 5 also illustrates that $d_{i,w}$ is not independent of cycle; instead it often increases with *i*. This reveals another source of systematic error, as most models assume that $d_{i,w}$ is independent of cycle (see, e.g., Ruijter et al. (2009), Equation (4); Lievens et al. (2012), Equation (7); Liu and Saint (2002), Equation (2)).

### Calculation of Fluorescence Profiles

Having calculated $f_{i,w}^-$ and $f_{i,w}^+$ pointwise through (46), we leverage (40) with (8), (13), (24), (26), and (41) to compute fluorescence curves with uncertainty. We prescribe common values for assay parameters *C*, $\mathcal {V}$, ${\bar{p}}$, and *R* and assume $\mathbb {E}\left[ I\right]$ is known. However, we also need to specify the relationship between $\textsf{Var}\left[ I\right]$ and $\mathbb {E}\left[ I\right]$, as well as the distribution of each $F_{i,w}$.

We will assume for simplicity that

48 $$\begin{aligned} \textsf{Var}\left[ I\right] = \chi \mathbb {E}\left[ I\right] \end{aligned}$$

for some constant $\chi > 0$. If we make the conventional assumption (Nedelman et al. 1992; Sundberg et al. 2010; Tellinghuisen and Spiess 2015; Ruiz-Villalba et al. 2021) that *I* is a Poisson random-variable,^3^ then $\chi = 1$. However, (48) can also correspond to different probability distributions. If *I* obeys a negative binomial distribution with probability of success $\varphi \in (0, 1)$, for example, then $\chi = 1/\varphi > 1$.

Our assumption on the distribution of each $F_{i,w}$ is rooted in the characteristic values of the fluorescence parameters $b_{i,w}$ and $d_{i,w}$. As Fig. 5 demonstrates that $b_{i,w}$ and $d_{i,w}$ are typically around 1 and $10^{-6}$, respectively, this implies with (40a) that $\mathbb {E}\left[ \Delta X_i\right]$ should be more than $10^6$ for $\mathbb {E}\left[ F_{i,w}\right] > b_{i,w}$. That is, the expected number of successful Bernoulli trials over all *i* cycles should be more than $10^6$ for the fluorescence to reach levels above background. With such a large sample size, it is natural to invoke the central limit theorem and assume that $F_{i,w}$ obeys a normal distribution with mean $\mathbb {E}\left[ F_{i,w}\right]$ from (40a) and variance $\textsf{Var}\left[ F_{i,w}\right]$ from (40b).

**Fig. 6:**
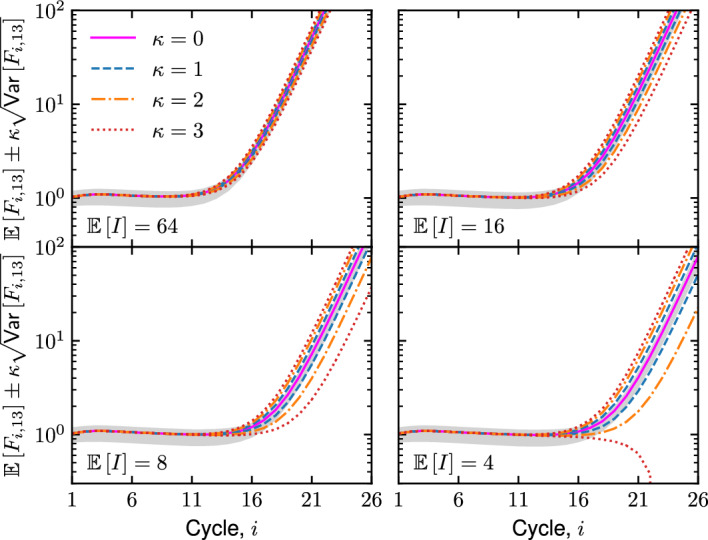
Fluorescence curves calculated from (40) with (8), (13), (24), (26), and (41) for $w=13$. Each subplot is associated with a different input copy-number $\mathbb {E}\left[ I\right]$, as depicted in each bottom-left corner, while all subplots have $C=0.125$ pmol/L, $\mathcal {V}=20$ $\mu$L, ${\bar{p}}=0.9$, and $R=1$. The initial distribution is taken as Case D with (48) and $\chi = 1$. The light-grey shadings at each *i* depict the region between $\min \limits _{1\le w\le 96}\mathbb {E}\left[ F_{i,w}\right]$ and $\max \limits _{1\le w\le 96}\mathbb {E}\left[ F_{i,w}\right]$

In Fig. 6, fluorescence curves are computed with uncertainty for ds-DNA for $w=13$. Different $\mathbb {E}\left[ I\right]$ ranging from 64 (top-left subplot) down to 4 (bottom-right subplot) are investigated. The expected fluorescence depicts behavior that is characteristic of the background and exponential phase observed in typical measurements. During the initial cycles, the term $b_{i,w}$ is much larger than $d_{i,w}\mathbb {E}\left[ \Delta X_i\right]$ and only small changes in fluorescence are observed. Here, the fluorescence is in the background regime. After more cycles are performed, however, the expected value of fluorescence increases exponentially with cycle. The change in $\mathbb {E}\left[ F_{i,13}\right]$ with $\mathbb {E}\left[ I\right]$ is also in line with typical trends. As $\mathbb {E}\left[ I\right]$ is decreased, the expected value of the fluorescence in the exponential phase shifts to the right. In other words, more cycles are required to reach the same expected fluorescence value.

This approach provides quantitative estimates of sources and magnitudes of uncertainty in different regimes, which are difficult to determine from replicate experiments alone. For large $\mathbb {E}\left[ I\right]$ and small *i*, the well-to-well variation in expected value (light-grey, shaded regions in Fig. 6) is larger or comparable to the error in fluorescence in each well. As such, spatial variation has a significant impact on the error. After many cycles have been completed, on the other hand, the uncertainty in fluorescence is strongly dependent on the expected initial copy-number, increasing drastically with decreasing $\mathbb {E}\left[ I\right]$. At $\mathbb {E}\left[ I\right] =4$, the fluorescence does not reach values that are larger than the background fluorescence by an amount that is statistically significant (for $\kappa = 3$). This observation suggests that quantifying the uncertainty in fluorescence can provide limitations on the measurement.

### Limit of Detection

After performing *n* PCR cycles, the fluorescence produced by PCR is only useful if it is larger than background levels by a statistically significant amount. Requiring the increase to be at least some $0< \kappa < \infty$ standard deviations, this amounts to the constraint

49 $$\begin{aligned} \mathbb {E}\left[ F_{n,w}\right] - \kappa \sqrt{\textsf{Var}\left[ F_{n,w}\right] } \ge b_{n,w}, \end{aligned}$$

for some well *w*. Equation (49) describes a feasible region of design space for a real-time PCR assay. It can be considered to depend on *n*, $b_{n,w}$, $d_{n,w}$, ${\bar{p}}$, *R*, *r*, $\mathbb {E}\left[ I\right]$, and $\textsf{Var}\left[ I\right]$ through (8), (13), (24), (26), and (41). However, (49) can be further simplified by substituting (40) and rearranging, leading to

$$\begin{aligned} \textsf{CV}\left[ \Delta X_n\right] \le \frac{1}{\kappa }, \end{aligned}$$

an expression that is no longer dependent on *w*. Since $\textsf{CV}\left[ \Delta X_n\right] \ge 0$ and $\kappa > 0$, it follows that

50 $$\begin{aligned} \textsf{CV}\left[ \Delta X_n\right] ^2 \le \frac{1}{\kappa ^2}. \end{aligned}$$

In addition, since

$$\begin{aligned} \textsf{CV}\left[ \Delta X_n\right] ^2 = \frac{\textsf{Var}\left[ X_n\right] + \textsf{Var}\left[ X_0\right] - 2\textsf{Cov}\left[ X_n,X_0\right] }{\mathbb {E}\left[ X_n\right] ^2 - 2\mathbb {E}\left[ X_n\right] \mathbb {E}\left[ X_0\right] + \mathbb {E}\left[ X_0\right] ^2} = \textsf{CV}\left[ X_n\right] ^2 + O\left( \lambda _1^{-n}\right) \end{aligned}$$

by (13), (28), and (41), and *n* typically ranges from 35 to 50, the error in approximating $\textsf{CV}\left[ \Delta X_n\right] ^2$ by $\textsf{CV}\left[ X_n\right] ^2$ is extremely small, often less than machine precision. As a result, the left-hand-side of (50) is expressed as the term on the right-hand-side of (32), or

51 $$\begin{aligned} \alpha \left( R\right) \textsf{CV}\left[ I\right] ^2 + \frac{\beta \left( R, {\bar{p}}, r\right) }{\mathbb {E}\left[ I\right] } \le \frac{1}{\kappa ^2}, \end{aligned}$$

where $\alpha$ and $\beta$ are defined in (33) for Case D, where the input is ds-DNA; Case RF, where the input is fs-RNA; and Case RR, where the input is rs-RNA. If we let *I* satisfy (48), Equation (51) can be rearranged to

52 $$\begin{aligned} \mathbb {E}\left[ I\right] \ge \left( \chi \alpha + \beta \right) \kappa ^2. \end{aligned}$$

The limit of detection, *L*, or the smallest expected-initial-copy-number that can be detected reliably, is then

53 $$\begin{aligned} L = \min {\left\{ y\in \mathbb {N} \mid y \ge \left( \chi \alpha + \beta \right) \kappa ^2\right\} }. \end{aligned}$$

The *largest* coefficient of variation in *I* that can be detected, *M*, is estimated from (53) and (48), or

54 $$\begin{aligned} M :=\sqrt{\frac{\chi }{L}}. \end{aligned}$$

To compute typical values of *L* and *M*, we evaluated them as in (53) and (54) with $\chi = 1$, $\kappa = 3$, for 100 equally-spaced ${\bar{p}}\in [0.8, 0.99]$, $R\in [0.9, 1.1]$, and $r \in [0.2, 0.99]$ (including endpoints). For *I* representing ds-DNA (Case D), we find that *L* is either 5 or 6, corresponding to *M* of 0.447 and 0.408, respectively. For *I* representing RNA as in Case RF or Case RR, *L* ranges between 9 and 52, corresponding to *M* of 1/3 and 0.139, respectively. The range of *L* is the same for fs-RNA and rs-RNA.

## Conclusions and Future Work

In this work, we presented a new model for fluorescence in real-time PCR that reduced bias and quantified uncertainty. Distinguishing between complementary strands provided a stoichiometric description of fluorescence reported by hydrolysis probes and permitted application to initial conditions encountered in RT-qPCR. Viewing the fluorescence as a Beer’s Law analog enabled the background fluorescence to be determined without extrapolation or assuming a certain relationship with cycle. It also allowed for measurement and calculation of background fluorescence without adjusting amplification data. Incorporating the variance in copy number into the fluorescence model enabled quantification of fluorescence uncertainty and analytical expressions for the limit of detection.

In addition to their practical utility, the two-type branching-process and repurposed fluorescence-model provided new intuition on the physics and chemistry in PCR. At short times, there is a lag in exponential growth (usually at most 5 cycles) as the ratio of expected strand counts changes from its initial to critical value, *R*. The quantity *R* represents the square root of the ratio of the two synthesis efficiencies (see (3)). In constrast to a previous report investigating deterministic models, we found that the initial composition only impacts the dynamics after the lag phase if $R\ne 1$.

The variance in the fluorescence is dominated by a term that increases exponentially by twice the factor of the expected value. This explains, in part, why quantification by end-point PCR is not reproducible. The three terms dominating the variance were attributed to arise from the initial variance ($\nu _{1,1}$, see (29a)), imperfect amplification ($\eta _{1,1}^{(1)}$, see (29b)), and deviation in directional efficiencies (i.e., $R\ne 1$; see $\eta _{1,1}^{(2)}$ in (29c)). The fluorescence model for hydrolysis probes demonstrated that the background fluorescence originates from the molar fluorescence of the inactive probe times the total concentration of probe. The incremental increase in fluorescence is proportional to the difference in molar fluorescence between active and inactive probe and, like the background fluorescence, is neither independent of cycle nor a linear function of cycle.

The stochastic view of PCR explains, in part, why deterministic methods that use reaction-specific amplification probabilities are generally less accurate (Ruijter et al. 2013). This is because, for each well *w* and cycle *i*, $N_{i,w}/N_{i-1,w}\ne 1 + p$ (see Equation (5)). Even if $R=1$ and $N_{i,1},\ldots , N_{i,m}$ are independent and distributed identically to $N_i$ for each *i*, this is not necessarily true because not every realization of a random variable is equal to its expected value.

While this work applied the stochastic model of PCR to the fluorescence reported by hydrolysis probes, it can readily be extended to other chemistries. For example, for probes that anneal to forward-stranded DNA, (37) instead becomes

$$\begin{aligned} F_{i,w} = b_{i,w} + d_{i,w} X_{i-1,w}. \end{aligned}$$

For these probes, the fluorescence is measured during the annealing portion of each cycle where only $i-1$ cycles of PCR have been completed.

To capture the fluorescence reported by DNA-binding dyes, on the other hand, an extension to the model is needed. This is because the amount of dye bound to a DNA strand depends on the total amount of DNA present in solution (this includes DNA that is not template, like primers (Ruijter et al. 2009)). Application to fluorescent dyes represents an interesting direction for future generalizations of the fluorescence model.

**Fig. 7:**
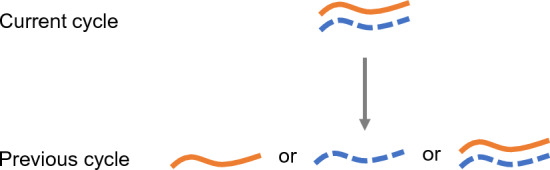
Conceptualization of probabilistic view of PCR in backwards time. Each ds-DNA complex present after *i* cycles have been completed may have originated from a forward strand or a reverse strand. Each complex may also be identical to a ds-DNA complex present at the end of the previous cycle

Finally, our approach in this work focused on quantifying the dynamics and uncertainty of fluorescence when the initial amount of each complementary strand is known, as well as their amplification probabilities. (That is, we assumed that $\mathbb {E}\left[ I\right]$, $\textsf{Var}\left[ I\right]$, ${\bar{p}}$, *R*, and *r* were known.) However, the ultimate goal of monitoring the kinetics of PCR by fluorescent probes is to infer $\mathbb {E}\left[ I\right]$, the expected input number. As such, it is of interest to extend the approach to UQ-PCR, or uncertainty quantification of the initial amount of DNA. To this end, another direction for future work is the investigation of the probabilistic nature of PCR in backwards time (see Fig. 7).

### Supplementary Information

Below is the link to the electronic supplementary material.

Supplementary file 1 (pdf 18281 KB)

## Data Availability

The experimental data and software associated with this study are available at https://github.com/usnistgov/bias-uq-pcr. Additional parameters computed from experimental data are tabulated in the Supplementary Information.

## Appendix A Experimental

Reagents were obtained from suppliers for use in experiments. A DNA suspension buffer (TE$^{-4}$) of pH 8.0 with 10 mmol/L Tris and 0.1 mmol/L EDTA was obtained from Teknova (Hollister, CA). 6-Carboxyfluorescein (6-FAM), single isomer, was purchased in solid form from Thermo Fisher Scientific (Waltham, MA). The working solution of 6-FAM was prepared by dissolving 10.1 mg 6-FAM in 1.75 mL of absolute ethanol (Sigma-Aldrich, St. Louis, MO). Subsequently, 10.0 $\mu$L of the ethanolic solution was dissolved in 500 mL of TE$^{-4}$. A TaqMan minor groove binder probe with non-fluorescent quencher (Applied Biosystems, Waltham, MA), possessing the sequence $5^\prime -$ACCCCGCATTACGTTTGGTGGACC$-3^\prime$ and reporter (6-FAM) of the National Center for Immunization and Respiratory Diseases (U.S.). Division of Viral Diseases (2020) 2019-nCoV_N1 assay, was obtained from Thermo Fisher Scientific. Working solutions of probe were prepared by adding TE$^{-4}$ to a portion of the stock solution to yield a solution of concentration 5 $\mu$mol/L probe.

In a typical experiment, a solution was prepared with 25 vol. % TE$^{-4}$ at a specific concentration of either 6-FAM (active probe) or TaqMan probe (inactive probe). After mixing, 20 $\mu$L was transferred into each well of a 96-well plate. The plate was covered with adhesive film and centrifuged. After assessing that air bubbles were not visible, the plate was placed in an Applied Biosystems 7500 HID Real-Time PCR instrument. The thermal cycling protocol consisted of a 2 min holding stage at 55 $^\circ$C (328 K), followed by 45 cycles. Each cycle consisted of 30 s at 55 $^\circ$C (328 K), followed by 3 s at 95 $^\circ$C (368 K). Data collection occurred during the 55 $^\circ$C step in each cycle. The raw data through filter 1 was exported with HID Real-Time PCR Analysis Software, Version 1.2 (Applied Biosystems). The fluorescence values were divided by $10^6$ before subsequent analysis.

## Appendix B Extended Derivations

### B.1 Derivation of (24) from (23)

Consider some $\varvec{B}\in \mathbb {R}^{2\times 2}$. From (12), we can write

B1 $$\begin{aligned} \varvec{A}^i \varvec{B} \left( \varvec{A}^\top \right) ^i =&\left( \sum _{j=1}^2 \lambda _j^i \varvec{x}_j\varvec{z}_j^\top \right) \varvec{B}\left( \sum _{k=1}^2 \lambda _k^i \varvec{z}_k\varvec{x}_k^\top \right) \nonumber \\ =&\sum _{j=1}^2\sum _{k=1}^2 \left( \lambda _j\lambda _k\right) ^i \varvec{x}_j\varvec{z}_j^\top \varvec{B}\varvec{z}_k\varvec{x}_k^\top ,\nonumber \\ =&\sum _{j=1}^2\sum _{k=1}^2 \left( \lambda _j\lambda _k\right) ^i \varvec{T}_{j,k}\left( \varvec{B}\right) , \end{aligned}$$

where $\varvec{T}_{j,k}:\mathbb {R}^{2\times 2}\mapsto \mathbb {R}^{2\times 2}$ is the linear operator

B2 $$\begin{aligned} \varvec{T}_{j,k}\left( \varvec{B}\right) = \varvec{x}_j\varvec{z}_j^\top \varvec{B} \varvec{z}_k \varvec{x}_k^\top = \left( \varvec{z}_j^\top \varvec{B} \varvec{z}_k\right) \varvec{x}_j\varvec{x}_k^\top . \end{aligned}$$

Application of (B1) to (23) leads to

B3 $$\begin{aligned} \textsf{Var}\left[ \varvec{U}_i\right] =&\sum _{j=1}^2 \sum _{k=1}^2 \left( \lambda _j\lambda _k\right) ^i \varvec{T}_{j,k}\left( \textsf{Var}\left[ \varvec{U}_0\right] \right) \nonumber \\&+\sum _{j=1}^2 \sum _{k=1}^2 \sum _{\ell =1}^2\sum _{q=0}^{i-1}\lambda _\ell ^q\left( \lambda _j\lambda _k\right) ^{i-1-q}\varvec{T}_{j,k}\left( \varvec{K}_\ell \right) . \end{aligned}$$

For each $b\in \{\lambda _1^2, \lambda _1\lambda _2, \lambda _2^2\}$, the geometric series in (B3) can be simplified via

$$\begin{aligned} \sum _{q=0}^{i-1}\lambda _{\ell }^q b^{i - 1 - q} = b^{i-1} \sum _{q=0}^{i-1}\left( \frac{\lambda _{\ell }}{b}\right) ^q = b^{i-1}\left( \frac{1 - \left( \dfrac{\lambda _{\ell }}{b}\right) ^i}{1 - \dfrac{\lambda _{\ell }}{b}}\right) = \frac{b^i - \lambda _\ell ^i}{b - \lambda _\ell }. \end{aligned}$$

This admits the expression

B4 $$\begin{aligned} \textsf{Var}\left[ \varvec{U}_i\right] =&\sum _{j{=}1}^2 \sum _{k=1}^2 \left( \lambda _j\lambda _k\right) ^i\varvec{T}_{j,k} \left( \textsf{Var}\left[ \varvec{U}_0\right] \right) {+} \sum _{j=1}^2 \sum _{k=1}^2 \sum _{\ell =1}^2 \left( \frac{\left( \lambda _j\lambda _k\right) ^i - \lambda _\ell ^i}{\lambda _j\lambda _k {-} \lambda _\ell }\right) \varvec{T}_{j,k}\left( \varvec{K}_\ell \right) , \nonumber \\ =&\sum _{j=1}^2 \sum _{k=1}^2 \left( \lambda _j\lambda _k\right) ^i\varvec{T}_{j,k}\left( \textsf{Var}\left[ \varvec{U}_0\right] + \sum _{\ell =1}^2 \frac{\varvec{K}_\ell }{\lambda _j\lambda _k - \lambda _\ell }\right) \nonumber \\&- \sum _{j=1}^2 \sum _{k=1}^2 \sum _{\ell =1}^2 \lambda _\ell ^i\varvec{T}_{j,k}\left( \frac{ \varvec{K}_\ell }{\lambda _j\lambda _k - \lambda _\ell }\right) . \end{aligned}$$

Since, from (25) and (B2),

$$\begin{aligned} \left\{ \begin{aligned} \varvec{T}_{j,k}\left( \textsf{Var}\left[ \varvec{U}_0\right] \right) =&\nu _{j,k}\varvec{x}_j\varvec{x}_k^\top , \\ \varvec{T}_{j,k}\left( \frac{\varvec{K}_{\ell }}{\lambda _j\lambda _k - \lambda _\ell }\right) =&\eta _{j,k}^\ell \varvec{x}_j\varvec{x}_k^\top , \\ \end{aligned} \right. \end{aligned}$$

it is evident that (B4) is identical to (24).

### B.2 Derivation of (41)

In this section, the derivation of (41) is completed by investigating the cross-covariance matrix, defined as

$$\begin{aligned} \mathcal {K}\left[ \varvec{X}, \varvec{Y}\right] = \mathbb {E}\left[ \varvec{X} \varvec{Y}^\top \right] - \mathbb {E}\left[ \varvec{X}\right] \mathbb {E}\left[ \varvec{Y}\right] ^\top \end{aligned}$$

for any two random vectors $\varvec{X}$ and $\varvec{Y}$. Notice that $\mathcal {K}\left[ \varvec{X}, \varvec{X}\right] = \textsf{Var}\left[ \varvec{X}\right]$.

From the law of total expectation,

$$\begin{aligned} \mathbb {E}\left[ \varvec{U}_i\right] \mathbb {E}\left[ \varvec{U}_0\right] ^\top =\mathbb {E}\left[ \mathbb {E}\left[ \varvec{U}_i \mid \varvec{U}_{i-1}\right] \right] \mathbb {E}\left[ \varvec{U}_0\right] ^\top = \varvec{A}\mathbb {E}\left[ \varvec{U}_{i-1}\right] \mathbb {E}\left[ \varvec{U}_0\right] ^\top , \end{aligned}$$

and

$$\begin{aligned} \begin{aligned} \mathbb {E}\left[ \varvec{U}_i \varvec{U}_0^\top \right] =&\mathbb {E}\left[ \mathbb {E}\left[ \varvec{U}_i\varvec{U}_0^\top \mid \varvec{U}_{i-1}, \varvec{U}_0\right] \right] = \mathbb {E}\left[ \mathbb {E}\left[ \varvec{U}_i \mid \varvec{U}_{i-1}\right] \varvec{U}_0^\top \right] \\ =&\varvec{A}\mathbb {E}\left[ \varvec{U}_{i-1}\varvec{U}_0^\top \right] . \end{aligned} \end{aligned}$$

The latter expression follows because $\varvec{U}_i$ and $\varvec{U}_0$ are independent when conditioned on $\varvec{U}_{i-1}$. Thus,

$$\begin{aligned} \begin{aligned} \mathcal {K}\left[ \varvec{U}_i, \varvec{U}_0\right] =&\varvec{A}\mathcal {K}\left[ \varvec{U}_{i-1}, \varvec{U}_0\right] = \varvec{A}^i\textsf{Var}\left[ \varvec{U}_0\right] \\ =&\sum _{j=1}^2 \lambda _j^i \varvec{x}_j\varvec{z}_j^\top \textsf{Var}\left[ \varvec{U}_0\right] \\ =&\sum _{j=1}^2 \lambda _j^i \varvec{x}_j\varvec{z}_j^\top \begin{pmatrix}\textsf{Var}\left[ X_0\right] &{} 0 \\ 0 &{} \textsf{Var}\left[ Y_0\right] \end{pmatrix}, \end{aligned} \end{aligned}$$

where the second, third, and fourth step follow from induction, Equation (12), and the independence of $X_0$ and $Y_0$, respectively. In particular, it follows that

$$\begin{aligned} \begin{aligned} \textsf{Cov}\left[ X_i, X_0\right] =&\varvec{e}_1^\top \mathcal {K}\left[ \varvec{U}_i, \varvec{U}_0\right] \varvec{e}_1 \nonumber \\ =&\varvec{e}_1^\top \left( \sum _{j=1}^2 \lambda _j^i \varvec{x}_j\varvec{z}_j^\top \right) \varvec{e}_1\textsf{Var}\left[ X_0\right] \nonumber \\ =&\frac{\textsf{Var}\left[ X_0\right] }{2}\left( \lambda _1^i + \lambda _2^i\right) , \end{aligned} \end{aligned}$$

which is the same as (41).

## Footnotes

1 The expression which follows could have also been developed by substituting the first expression of (9) into the final expression of (7) and left-multiplying by $\varvec{Z}$, yielding

$$\begin{aligned} \varvec{Z}\mathbb {E}\left[ \varvec{U}_i\right] = \varvec{\Lambda }^i\varvec{Z}\mathbb {E}\left[ \varvec{U}_0\right] . \end{aligned}$$

2 In this work, we assume that the fluorescence is measured after each PCR cycle. This is typical for assays utilizing hydrolysis probes, but is not always the case.

3 There is some limited experimental evidence for assuming that *I* is a Poisson random-variable (Beer et al. 2007); however, its validity is challenging to assess experimentally. (If the initial number of DNA strands could be easily measured, there would be no need for qPCR.) In the future, it would be interesting to compare (48) to a result obtained from error propagation for a conventional experimental procedure (see, e.g., Hedges (2002)).

## Declarations

### Disclaimer

Certain equipment, instruments, software, or materials, commercial or non-commercial, are identified in this paper in order to specify the experimental procedure adequately. Such identification is not intended to imply recommendation or endorsement of any product or service by the National Institute of Standards and Technology, nor is it intended to imply that the materials or equipment identified are necessarily the best available for the purpose.
