# Supplementary material for: Reducing Bias and Quantifying Uncertainty in Fluorescence Produced by PCR
Source: Bull Math Biol. 2023 Aug 14;85(9):83. doi: 10.1007/s11538-023-01182-z (PMC10423706; doi:10.1007/s11538-023-01182-z)
Supplement: Supplementary file 1 — (pdf 18281 KB) [file 11538_2023_1182_MOESM1_ESM.pdf]

Supplementary Information for
Reducing Bias and Quantifying Uncertainty in Fluorescence Produced by PCR

Robert F. DeJaco[1,2,†], Matthew J. Roberts[1,3], Erica L. Romsos[4], Peter M. Vallone[4], and Anthony J. Kearsley[1]

[1]Applied and Computational Mathematics Division, National Institute of Standards and Technology, Gaithersburg, MD, USA
[2]Department of Chemistry and Biochemistry, University of Maryland, College Park, MD, USA
[3]Cost Analysis and Research Division, Institute for Defense Analyses, Alexandria, VA, USA
[4]Biomolecular Measurement Division, National Institute of Standards and Technology, Gaithersburg, MD, USA
[†]To whom correspondence should be addressed. Email: robert.dejaco@nist.gov

May 12, 2023

List of Tables

S1 Molar Fluorescence Parameters for Well A1 ($w = 1$) . . . 2
S2 Molar Fluorescence Parameters for Well A2 ($w = 2$) . . . 4
S3 Molar Fluorescence Parameters for Well A3 ($w = 3$) . . . 6
S4 Molar Fluorescence Parameters for Well A4 ($w = 4$) . . . 8
S5 Molar Fluorescence Parameters for Well A5 ($w = 5$) . . . 10
S6 Molar Fluorescence Parameters for Well A6 ($w = 6$) . . . 12
S7 Molar Fluorescence Parameters for Well A7 ($w = 7$) . . . 14
S8 Molar Fluorescence Parameters for Well A8 ($w = 8$) . . . 16
S9 Molar Fluorescence Parameters for Well A9 ($w = 9$) . . . 18
S10 Molar Fluorescence Parameters for Well A10 ($w = 10$) . . . 20
S11 Molar Fluorescence Parameters for Well A11 ($w = 11$) . . . 22
S12 Molar Fluorescence Parameters for Well A12 ($w = 12$) . . . 24
S13 Molar Fluorescence Parameters for Well B1 ($w = 13$) . . . 26
S14 Molar Fluorescence Parameters for Well B2 ($w = 14$) . . . 28
S15 Molar Fluorescence Parameters for Well B3 ($w = 15$) . . . 30
S16 Molar Fluorescence Parameters for Well B4 ($w = 16$) . . . 32
S17 Molar Fluorescence Parameters for Well B5 ($w = 17$) . . . 34
S18 Molar Fluorescence Parameters for Well B6 ($w = 18$) . . . 36
S19 Molar Fluorescence Parameters for Well B7 ($w = 19$) . . . 38
S20 Molar Fluorescence Parameters for Well B8 ($w = 20$) . . . 40
S21 Molar Fluorescence Parameters for Well B9 ($w = 21$) . . . 42
S22 Molar Fluorescence Parameters for Well B10 ($w = 22$) . . . 44
S23 Molar Fluorescence Parameters for Well B11 ($w = 23$) . . . 46
S24 Molar Fluorescence Parameters for Well B12 ($w = 24$) . . . 48
S25 Molar Fluorescence Parameters for Well C1 ($w = 25$) . . . 50

S26 Molar Fluorescence Parameters for Well C2 ($w = 26$) 52
S27 Molar Fluorescence Parameters for Well C3 ($w = 27$) 54
S28 Molar Fluorescence Parameters for Well C4 ($w = 28$) 56
S29 Molar Fluorescence Parameters for Well C5 ($w = 29$) 58
S30 Molar Fluorescence Parameters for Well C6 ($w = 30$) 60
S31 Molar Fluorescence Parameters for Well C7 ($w = 31$) 62
S32 Molar Fluorescence Parameters for Well C8 ($w = 32$) 64
S33 Molar Fluorescence Parameters for Well C9 ($w = 33$) 66
S34 Molar Fluorescence Parameters for Well C10 ($w = 34$) 68
S35 Molar Fluorescence Parameters for Well C11 ($w = 35$) 70
S36 Molar Fluorescence Parameters for Well C12 ($w = 36$) 72
S37 Molar Fluorescence Parameters for Well D1 ($w = 37$) 74
S38 Molar Fluorescence Parameters for Well D2 ($w = 38$) 76
S39 Molar Fluorescence Parameters for Well D3 ($w = 39$) 78
S40 Molar Fluorescence Parameters for Well D4 ($w = 40$) 80
S41 Molar Fluorescence Parameters for Well D5 ($w = 41$) 82
S42 Molar Fluorescence Parameters for Well D6 ($w = 42$) 84
S43 Molar Fluorescence Parameters for Well D7 ($w = 43$) 86
S44 Molar Fluorescence Parameters for Well D8 ($w = 44$) 88
S45 Molar Fluorescence Parameters for Well D9 ($w = 45$) 90
S46 Molar Fluorescence Parameters for Well D10 ($w = 46$) 92
S47 Molar Fluorescence Parameters for Well D11 ($w = 47$) 94
S48 Molar Fluorescence Parameters for Well D12 ($w = 48$) 96
S49 Molar Fluorescence Parameters for Well E1 ($w = 49$) 98
S50 Molar Fluorescence Parameters for Well E2 ($w = 50$) 100
S51 Molar Fluorescence Parameters for Well E3 ($w = 51$) 102
S52 Molar Fluorescence Parameters for Well E4 ($w = 52$) 104
S53 Molar Fluorescence Parameters for Well E5 ($w = 53$) 106
S54 Molar Fluorescence Parameters for Well E6 ($w = 54$) 108
S55 Molar Fluorescence Parameters for Well E7 ($w = 55$) 110
S56 Molar Fluorescence Parameters for Well E8 ($w = 56$) 112
S57 Molar Fluorescence Parameters for Well E9 ($w = 57$) 114
S58 Molar Fluorescence Parameters for Well E10 ($w = 58$) 116
S59 Molar Fluorescence Parameters for Well E11 ($w = 59$) 118
S60 Molar Fluorescence Parameters for Well E12 ($w = 60$) 120
S61 Molar Fluorescence Parameters for Well F1 ($w = 61$) 122
S62 Molar Fluorescence Parameters for Well F2 ($w = 62$) 124
S63 Molar Fluorescence Parameters for Well F3 ($w = 63$) 126
S64 Molar Fluorescence Parameters for Well F4 ($w = 64$) 128
S65 Molar Fluorescence Parameters for Well F5 ($w = 65$) 130
S66 Molar Fluorescence Parameters for Well F6 ($w = 66$) 132
S67 Molar Fluorescence Parameters for Well F7 ($w = 67$) 134
S68 Molar Fluorescence Parameters for Well F8 ($w = 68$) 136
S69 Molar Fluorescence Parameters for Well F9 ($w = 69$) 138
S70 Molar Fluorescence Parameters for Well F10 ($w = 70$) 140
S71 Molar Fluorescence Parameters for Well F11 ($w = 71$) 142
S72 Molar Fluorescence Parameters for Well F12 ($w = 72$) 144
S73 Molar Fluorescence Parameters for Well G1 ($w = 73$) 146
S74 Molar Fluorescence Parameters for Well G2 ($w = 74$) 148
S75 Molar Fluorescence Parameters for Well G3 ($w = 75$) 150

S76 Molar Fluorescence Parameters for Well G4 ($w = 76$) . . . 152
S77 Molar Fluorescence Parameters for Well G5 ($w = 77$) . . . 154
S78 Molar Fluorescence Parameters for Well G6 ($w = 78$) . . . 156
S79 Molar Fluorescence Parameters for Well G7 ($w = 79$) . . . 158
S80 Molar Fluorescence Parameters for Well G8 ($w = 80$) . . . 160
S81 Molar Fluorescence Parameters for Well G9 ($w = 81$) . . . 162
S82 Molar Fluorescence Parameters for Well G10 ($w = 82$) . . . 164
S83 Molar Fluorescence Parameters for Well G11 ($w = 83$) . . . 166
S84 Molar Fluorescence Parameters for Well G12 ($w = 84$) . . . 168
S85 Molar Fluorescence Parameters for Well H1 ($w = 85$) . . . 170
S86 Molar Fluorescence Parameters for Well H2 ($w = 86$) . . . 172
S87 Molar Fluorescence Parameters for Well H3 ($w = 87$) . . . 174
S88 Molar Fluorescence Parameters for Well H4 ($w = 88$) . . . 176
S89 Molar Fluorescence Parameters for Well H5 ($w = 89$) . . . 178
S90 Molar Fluorescence Parameters for Well H6 ($w = 90$) . . . 180
S91 Molar Fluorescence Parameters for Well H7 ($w = 91$) . . . 182
S92 Molar Fluorescence Parameters for Well H8 ($w = 92$) . . . 184
S93 Molar Fluorescence Parameters for Well H9 ($w = 93$) . . . 186
S94 Molar Fluorescence Parameters for Well H10 ($w = 94$) . . . 188
S95 Molar Fluorescence Parameters for Well H11 ($w = 95$) . . . 190
S96 Molar Fluorescence Parameters for Well H12 ($w = 96$) . . . 192

List of Figures

S1 Experimental data points (symbols, $(C^\ell, F^\ell_{i,1})$ for each $\ell = 1$ to q at fixed cycle i) compared to model for inactive probe (blue, dashed lines) and active probe (orange, straight lines). The subplots depict cycles $i = 1, 5, 10, 15, 20, 25, 30$, and 40 in left-to-right and top-to-bottom order. Well $w = 1$ is also called well A1. . . . 1
S2 As Figure S1 with well $w = 2$ (or A2). . . . 3
S3 As Figure S1 with well $w = 3$ (or A3). . . . 5
S4 As Figure S1 with well $w = 4$ (or A4). . . . 7
S5 As Figure S1 with well $w = 5$ (or A5). . . . 9
S6 As Figure S1 with well $w = 6$ (or A6). . . . 11
S7 As Figure S1 with well $w = 7$ (or A7). . . . 13
S8 As Figure S1 with well $w = 8$ (or A8). . . . 15
S9 As Figure S1 with well $w = 9$ (or A9). . . . 17
S10 As Figure S1 with well $w = 10$ (or A10). . . . 19
S11 As Figure S1 with well $w = 11$ (or A11). . . . 21
S12 As Figure S1 with well $w = 12$ (or A12). . . . 23
S13 As Figure S1 with well $w = 13$ (or B1). . . . 25
S14 As Figure S1 with well $w = 14$ (or B2). . . . 27
S15 As Figure S1 with well $w = 15$ (or B3). . . . 29
S16 As Figure S1 with well $w = 16$ (or B4). . . . 31
S17 As Figure S1 with well $w = 17$ (or B5). . . . 33
S18 As Figure S1 with well $w = 18$ (or B6). . . . 35
S19 As Figure S1 with well $w = 19$ (or B7). . . . 37
S20 As Figure S1 with well $w = 20$ (or B8). . . . 39
S21 As Figure S1 with well $w = 21$ (or B9). . . . 41
S22 As Figure S1 with well $w = 22$ (or B10). . . . 43
S23 As Figure S1 with well $w = 23$ (or B11). . . . 45

S24 As Figure S1 with well $w = 24$ (or B12). 47
S25 As Figure S1 with well $w = 25$ (or C1). 49
S26 As Figure S1 with well $w = 26$ (or C2). 51
S27 As Figure S1 with well $w = 27$ (or C3). 53
S28 As Figure S1 with well $w = 28$ (or C4). 55
S29 As Figure S1 with well $w = 29$ (or C5). 57
S30 As Figure S1 with well $w = 30$ (or C6). 59
S31 As Figure S1 with well $w = 31$ (or C7). 61
S32 As Figure S1 with well $w = 32$ (or C8). 63
S33 As Figure S1 with well $w = 33$ (or C9). 65
S34 As Figure S1 with well $w = 34$ (or C10). 67
S35 As Figure S1 with well $w = 35$ (or C11). 69
S36 As Figure S1 with well $w = 36$ (or C12). 71
S37 As Figure S1 with well $w = 37$ (or D1). 73
S38 As Figure S1 with well $w = 38$ (or D2). 75
S39 As Figure S1 with well $w = 39$ (or D3). 77
S40 As Figure S1 with well $w = 40$ (or D4). 79
S41 As Figure S1 with well $w = 41$ (or D5). 81
S42 As Figure S1 with well $w = 42$ (or D6). 83
S43 As Figure S1 with well $w = 43$ (or D7). 85
S44 As Figure S1 with well $w = 44$ (or D8). 87
S45 As Figure S1 with well $w = 45$ (or D9). 89
S46 As Figure S1 with well $w = 46$ (or D10). 91
S47 As Figure S1 with well $w = 47$ (or D11). 93
S48 As Figure S1 with well $w = 48$ (or D12). 95
S49 As Figure S1 with well $w = 49$ (or E1). 97
S50 As Figure S1 with well $w = 50$ (or E2). 99
S51 As Figure S1 with well $w = 51$ (or E3). 101
S52 As Figure S1 with well $w = 52$ (or E4). 103
S53 As Figure S1 with well $w = 53$ (or E5). 105
S54 As Figure S1 with well $w = 54$ (or E6). 107
S55 As Figure S1 with well $w = 55$ (or E7). 109
S56 As Figure S1 with well $w = 56$ (or E8). 111
S57 As Figure S1 with well $w = 57$ (or E9). 113
S58 As Figure S1 with well $w = 58$ (or E10). 115
S59 As Figure S1 with well $w = 59$ (or E11). 117
S60 As Figure S1 with well $w = 60$ (or E12). 119
S61 As Figure S1 with well $w = 61$ (or F1). 121
S62 As Figure S1 with well $w = 62$ (or F2). 123
S63 As Figure S1 with well $w = 63$ (or F3). 125
S64 As Figure S1 with well $w = 64$ (or F4). 127
S65 As Figure S1 with well $w = 65$ (or F5). 129
S66 As Figure S1 with well $w = 66$ (or F6). 131
S67 As Figure S1 with well $w = 67$ (or F7). 133
S68 As Figure S1 with well $w = 68$ (or F8). 135
S69 As Figure S1 with well $w = 69$ (or F9). 137
S70 As Figure S1 with well $w = 70$ (or F10). 139
S71 As Figure S1 with well $w = 71$ (or F11). 141
S72 As Figure S1 with well $w = 72$ (or F12). 143
S73 As Figure S1 with well $w = 73$ (or G1). 145

S74 As Figure S1 with well $w = 74$ (or G2). 147
S75 As Figure S1 with well $w = 75$ (or G3). 149
S76 As Figure S1 with well $w = 76$ (or G4). 151
S77 As Figure S1 with well $w = 77$ (or G5). 153
S78 As Figure S1 with well $w = 78$ (or G6). 155
S79 As Figure S1 with well $w = 79$ (or G7). 157
S80 As Figure S1 with well $w = 80$ (or G8). 159
S81 As Figure S1 with well $w = 81$ (or G9). 161
S82 As Figure S1 with well $w = 82$ (or G10). 163
S83 As Figure S1 with well $w = 83$ (or G11). 165
S84 As Figure S1 with well $w = 84$ (or G12). 167
S85 As Figure S1 with well $w = 85$ (or H1). 169
S86 As Figure S1 with well $w = 86$ (or H2). 171
S87 As Figure S1 with well $w = 87$ (or H3). 173
S88 As Figure S1 with well $w = 88$ (or H4). 175
S89 As Figure S1 with well $w = 89$ (or H5). 177
S90 As Figure S1 with well $w = 90$ (or H6). 179
S91 As Figure S1 with well $w = 91$ (or H7). 181
S92 As Figure S1 with well $w = 92$ (or H8). 183
S93 As Figure S1 with well $w = 93$ (or H9). 185
S94 As Figure S1 with well $w = 94$ (or H10). 187
S95 As Figure S1 with well $w = 95$ (or H11). 189
S96 As Figure S1 with well $w = 96$ (or H12). 191

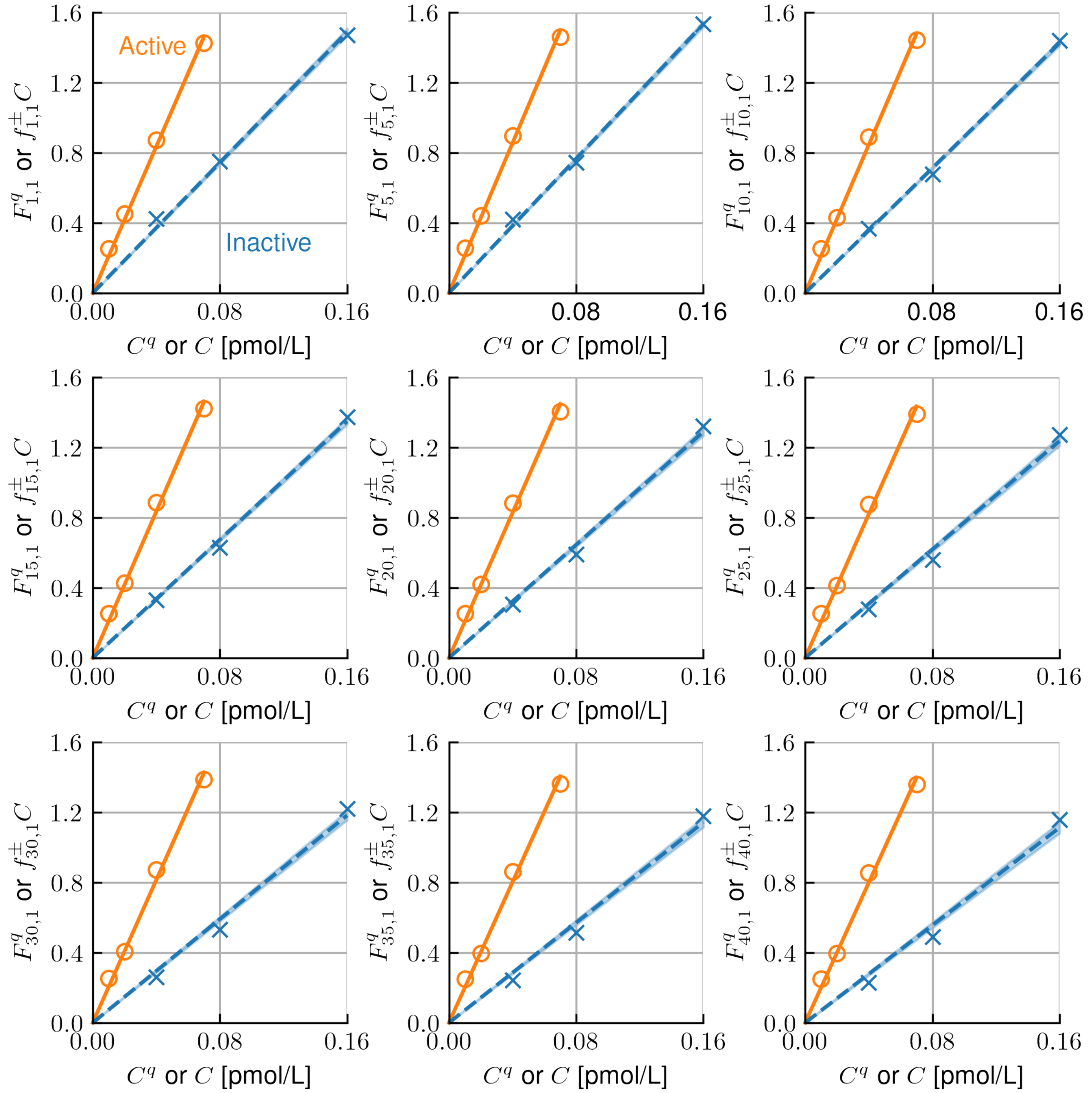

Fig. S1: Experimental data points (symbols, $(C^\ell, F^\ell_{i,1})$ for each $\ell = 1$ to q at fixed cycle i) compared to model for inactive probe (blue, dashed lines) and active probe (orange, straight lines). The subplots depict cycles $i = 1, 5, 10, 15, 20, 25, 30$, and 40 in left-to-right and top-to-bottom order. Well $w = 1$ is also called well A1.

Table S1: Molar Fluorescence Parameters for Well A1 ($w = 1$)

Cycle	Inactive		Active	
i	$f^-_{i,1}$	$\sigma^-_{i,1}$	$f^+_{i,1}$	$\sigma^+_{i,1}$
1	9.30	0.039	20.90	0.044
2	9.59	0.036	21.13	0.037
3	9.76	0.035	21.39	0.039
4	9.70	0.033	21.39	0.041
5	9.58	0.032	21.39	0.041
6	9.42	0.030	21.34	0.042
7	9.29	0.028	21.27	0.042
8	9.16	0.029	21.22	0.042
9	9.02	0.029	21.18	0.042
10	8.91	0.029	21.11	0.043
11	8.77	0.030	21.01	0.043
12	8.67	0.031	21.00	0.043
13	8.59	0.033	20.99	0.043
14	8.51	0.035	20.93	0.043
15	8.43	0.037	20.88	0.045
16	8.34	0.037	20.83	0.046
17	8.29	0.042	20.82	0.048
18	8.19	0.042	20.79	0.047
19	8.11	0.041	20.75	0.047
20	8.06	0.046	20.68	0.048
21	7.99	0.049	20.72	0.046
22	7.87	0.046	20.66	0.048
23	7.82	0.048	20.65	0.048
24	7.74	0.050	20.54	0.050
25	7.73	0.054	20.48	0.049
26	7.63	0.053	20.47	0.049
27	7.57	0.049	20.45	0.050
28	7.53	0.053	20.39	0.050
29	7.50	0.055	20.48	0.048
30	7.39	0.054	20.38	0.049
31	7.33	0.054	20.32	0.049
32	7.26	0.054	20.38	0.049
33	7.22	0.054	20.32	0.049
34	7.19	0.055	20.19	0.049
35	7.14	0.056	20.06	0.051
36	7.08	0.060	20.09	0.049
37	7.06	0.060	20.07	0.050
38	7.03	0.062	20.02	0.049
39	6.99	0.064	19.98	0.048
40	6.95	0.065	19.96	0.048
41	6.92	0.067	19.85	0.051
42	6.75	0.064	19.81	0.047
43	6.72	0.068	19.80	0.044
44	6.70	0.071	19.76	0.045
45	6.56	0.065	19.76	0.046

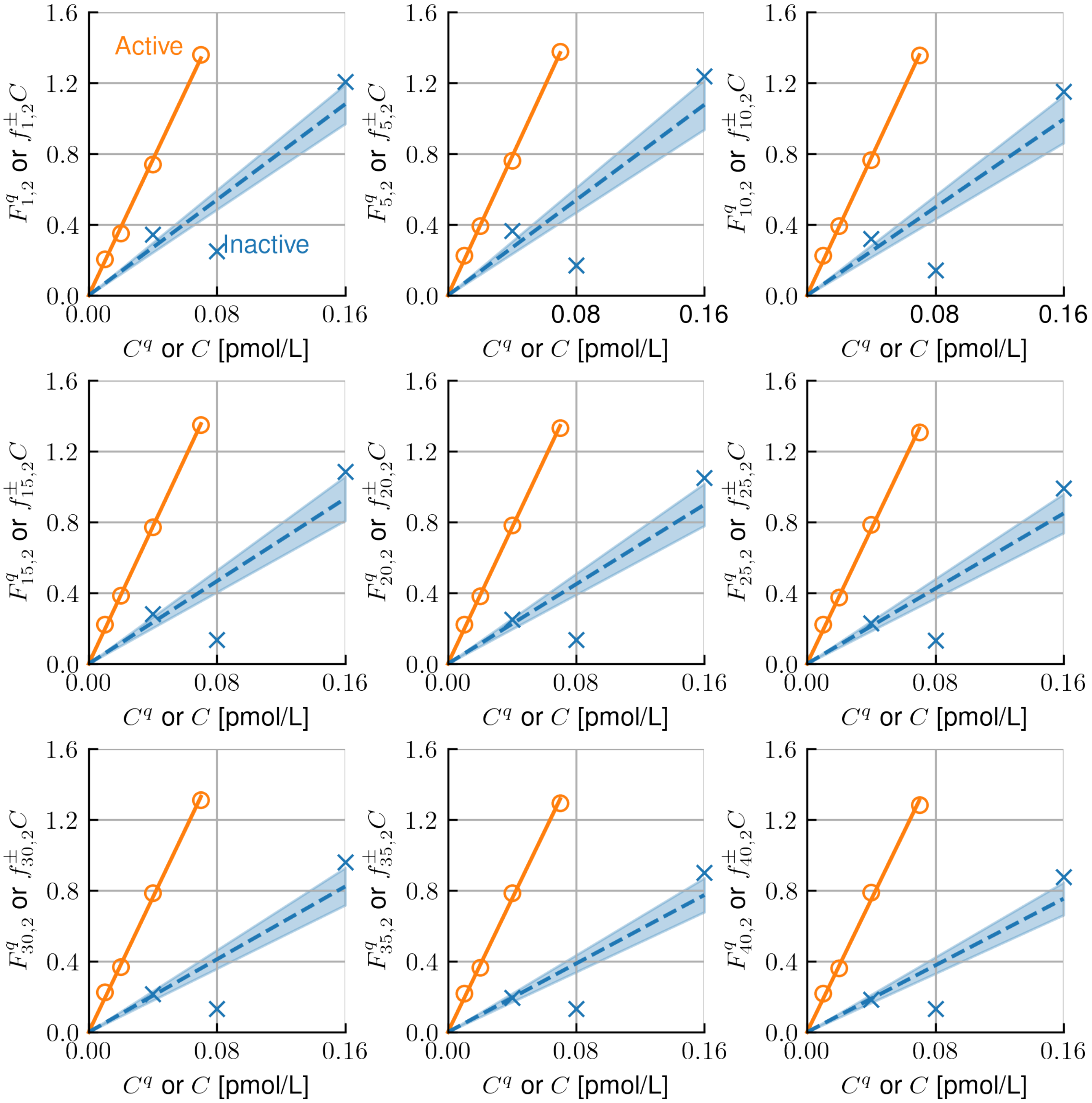

Fig. S2: As Figure S1 with well $w = 2$ (or A2).

Table S2: Molar Fluorescence Parameters for Well A2 ($w = 2$)

Cycle	Inactive		Active	
i	$f^-_{i,2}$	$\sigma^-_{i,2}$	$f^+_{i,2}$	$\sigma^+_{i,2}$
1	6.8	0.23	19.14	0.027
2	6.8	0.26	19.22	0.024
3	6.9	0.28	19.47	0.024
4	6.8	0.29	19.56	0.023
5	6.7	0.29	19.56	0.022
6	6.6	0.29	19.55	0.022
7	6.5	0.29	19.51	0.021
8	6.4	0.29	19.45	0.021
9	6.3	0.28	19.42	0.020
10	6.2	0.28	19.38	0.019
11	6.1	0.27	19.42	0.019
12	6.0	0.27	19.37	0.019
13	6.0	0.27	19.34	0.018
14	5.9	0.26	19.32	0.018
15	5.8	0.26	19.34	0.018
16	5.8	0.26	19.32	0.018
17	5.7	0.25	19.23	0.019
18	5.7	0.25	19.27	0.020
19	5.6	0.25	19.21	0.020
20	5.6	0.25	19.19	0.021
21	5.5	0.24	19.17	0.021
22	5.5	0.24	19.13	0.022
23	5.4	0.23	19.15	0.023
24	5.4	0.23	19.10	0.024
25	5.3	0.23	18.99	0.028
26	5.3	0.23	19.06	0.027
27	5.3	0.23	18.97	0.026
28	5.2	0.23	19.01	0.027
29	5.2	0.22	19.00	0.027
30	5.1	0.22	18.99	0.028
31	5.0	0.21	18.96	0.025
32	4.9	0.21	18.88	0.026
33	4.9	0.20	18.77	0.030
34	4.9	0.20	18.80	0.029
35	4.8	0.20	18.80	0.030
36	4.8	0.20	18.80	0.031
37	4.8	0.20	18.71	0.033
38	4.8	0.20	18.75	0.032
39	4.7	0.19	18.72	0.033
40	4.7	0.19	18.70	0.034
41	4.7	0.19	18.68	0.036
42	4.7	0.19	18.60	0.036
43	4.6	0.19	18.58	0.037
44	4.6	0.19	18.59	0.038
45	4.6	0.18	18.56	0.038

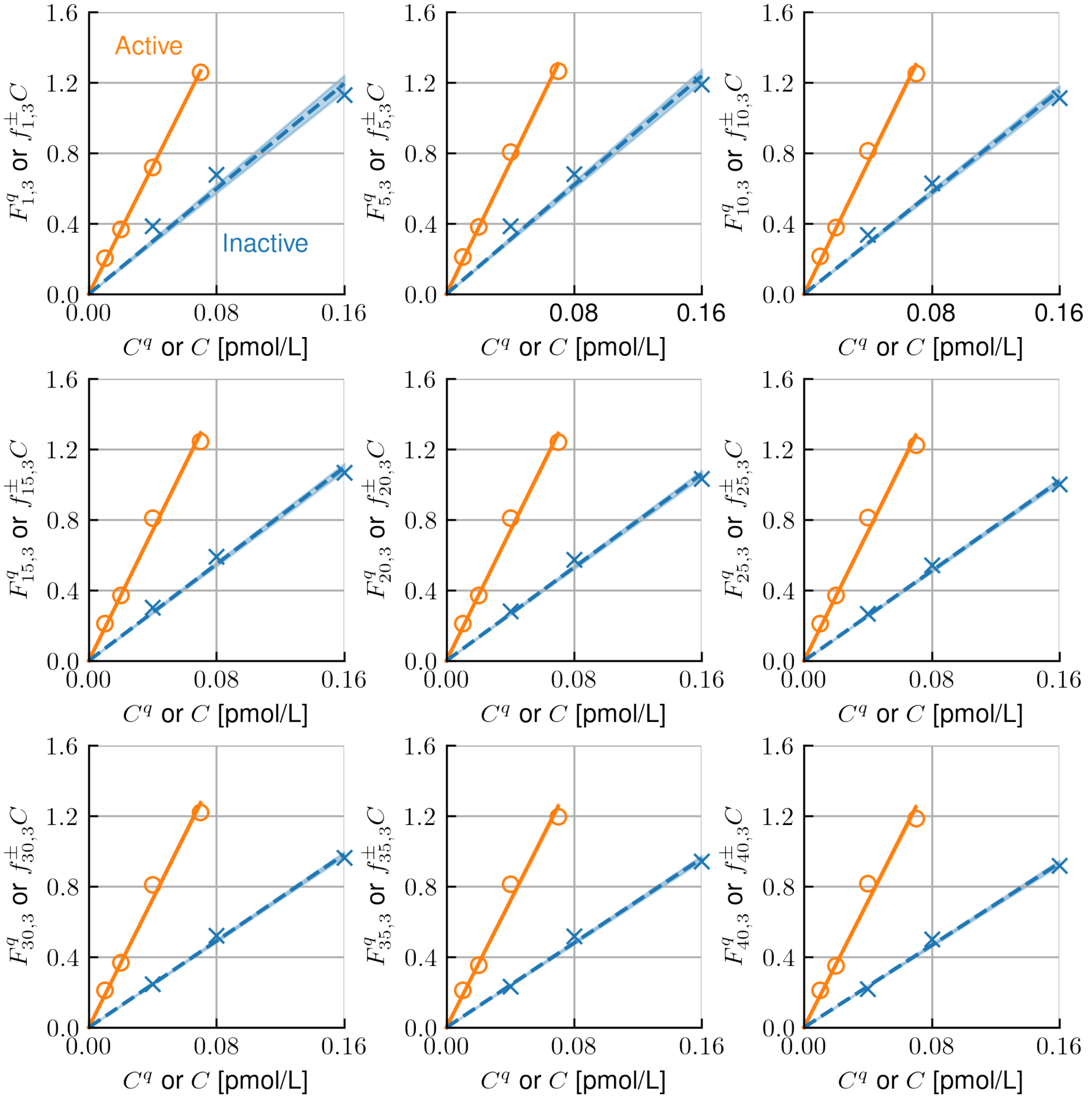

Fig. S3: As Figure S1 with well $w = 3$ (or A3).

Table S3: Molar Fluorescence Parameters for Well A3 ($w = 3$)

Cycle	Inactive		Active	
i	$f^-_{i,3}$	$\sigma^-_{i,3}$	$f^+_{i,3}$	$\sigma^+_{i,3}$
1	7.46	0.095	18.07	0.015
2	7.73	0.085	18.16	0.032
3	7.84	0.086	18.46	0.041
4	7.81	0.083	18.61	0.042
5	7.74	0.078	18.64	0.045
6	7.63	0.074	18.65	0.047
7	7.51	0.069	18.64	0.049
8	7.41	0.064	18.62	0.050
9	7.31	0.061	18.59	0.051
10	7.21	0.058	18.57	0.052
11	7.13	0.053	18.54	0.052
12	7.06	0.050	18.52	0.052
13	6.99	0.048	18.48	0.053
14	6.93	0.044	18.48	0.053
15	6.85	0.043	18.44	0.053
16	6.81	0.041	18.47	0.054
17	6.74	0.041	18.57	0.050
18	6.69	0.038	18.47	0.053
19	6.64	0.037	18.41	0.054
20	6.62	0.038	18.40	0.054
21	6.54	0.036	18.37	0.057
22	6.49	0.033	18.30	0.059
23	6.49	0.030	18.24	0.060
24	6.44	0.029	18.41	0.055
25	6.37	0.028	18.27	0.059
26	6.40	0.026	18.24	0.063
27	6.36	0.023	18.18	0.061
28	6.29	0.023	18.14	0.061
29	6.18	0.026	18.09	0.062
30	6.14	0.026	18.19	0.060
31	6.11	0.025	18.06	0.062
32	6.07	0.023	18.01	0.065
33	6.06	0.023	17.99	0.065
34	5.97	0.027	17.95	0.067
35	5.99	0.029	17.93	0.067
36	5.96	0.026	17.88	0.069
37	5.92	0.027	17.88	0.070
38	5.89	0.026	17.87	0.072
39	5.86	0.027	17.96	0.078
40	5.83	0.027	17.85	0.073
41	5.80	0.029	17.86	0.072
42	5.74	0.030	17.82	0.073
43	5.67	0.031	17.78	0.076
44	5.64	0.032	17.70	0.076
45	5.62	0.033	17.70	0.076

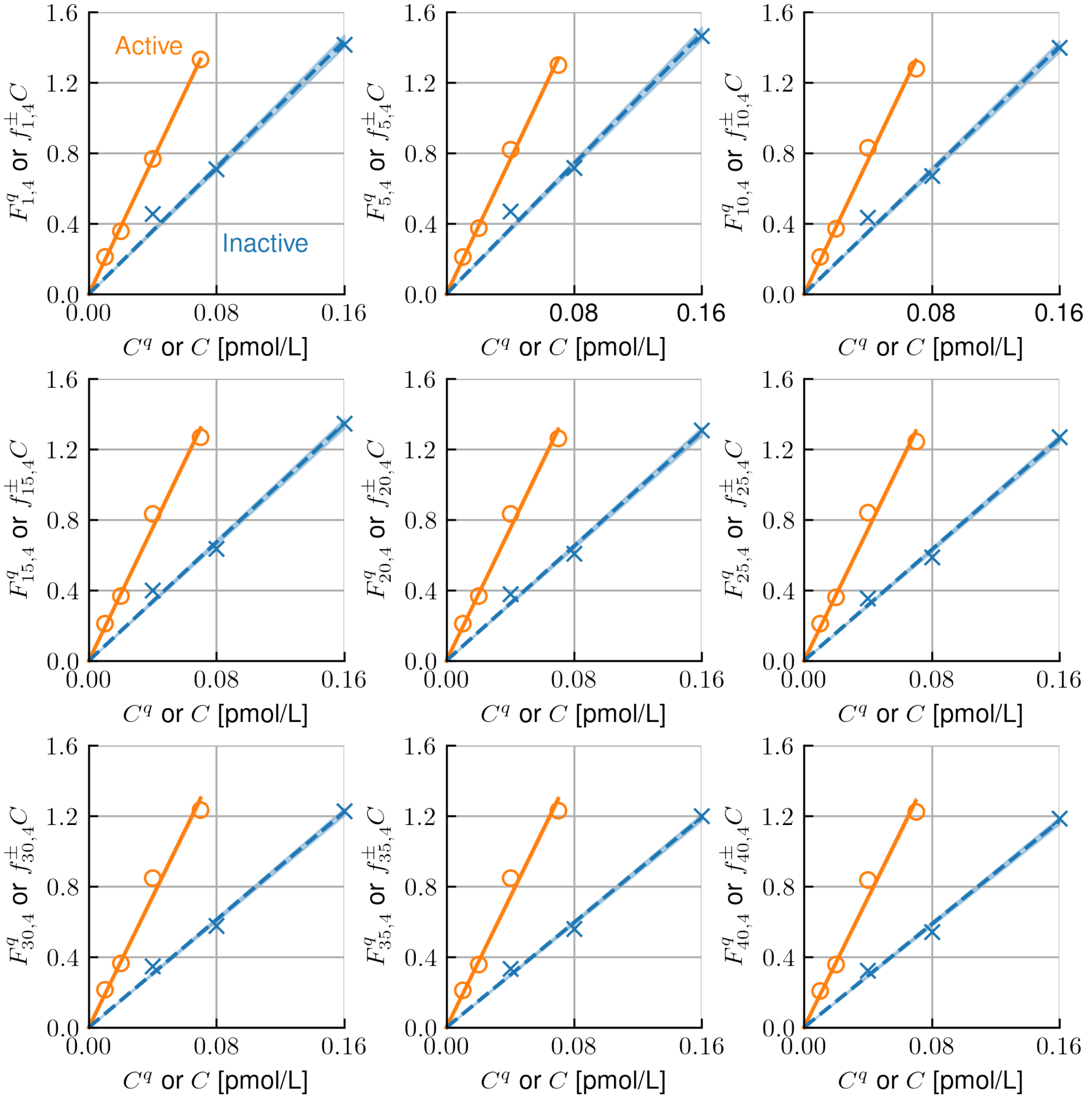

Fig. S4: As Figure S1 with well $w = 4$ (or A4).

Table S4: Molar Fluorescence Parameters for Well A4 ($w = 4$)

Cycle	Inactive		Active	
i	$f^-_{i,4}$	$\sigma^-_{i,4}$	$f^+_{i,4}$	$\sigma^+_{i,4}$
1	8.97	0.070	19.03	0.018
2	9.10	0.075	18.75	0.026
3	9.27	0.075	18.92	0.032
4	9.30	0.074	19.02	0.037
5	9.24	0.073	19.06	0.041
6	9.16	0.071	19.05	0.044
7	9.06	0.069	19.01	0.048
8	8.98	0.067	18.99	0.050
9	8.87	0.065	18.95	0.050
10	8.78	0.063	18.93	0.052
11	8.70	0.061	18.91	0.054
12	8.62	0.058	18.85	0.052
13	8.56	0.056	18.89	0.054
14	8.47	0.054	18.88	0.056
15	8.40	0.053	18.84	0.057
16	8.32	0.051	18.85	0.055
17	8.26	0.048	18.81	0.058
18	8.20	0.048	18.79	0.059
19	8.23	0.051	18.74	0.059
20	8.12	0.049	18.74	0.058
21	8.06	0.047	18.85	0.055
22	8.01	0.047	18.67	0.063
23	7.98	0.046	18.65	0.065
24	7.91	0.045	18.63	0.064
25	7.87	0.043	18.60	0.067
26	7.83	0.044	18.69	0.066
27	7.79	0.042	18.63	0.067
28	7.73	0.042	18.56	0.071
29	7.70	0.040	18.55	0.072
30	7.64	0.040	18.56	0.074
31	7.61	0.037	18.56	0.071
32	7.52	0.033	18.51	0.077
33	7.50	0.032	18.47	0.077
34	7.49	0.034	18.44	0.072
35	7.44	0.036	18.51	0.074
36	7.45	0.030	18.36	0.077
37	7.41	0.035	18.39	0.075
38	7.38	0.036	18.37	0.075
39	7.35	0.035	18.35	0.076
40	7.32	0.039	18.35	0.073
41	7.29	0.038	18.32	0.075
42	7.30	0.033	18.37	0.072
43	7.28	0.036	18.21	0.078
44	7.25	0.034	18.14	0.076
45	7.24	0.039	18.13	0.077

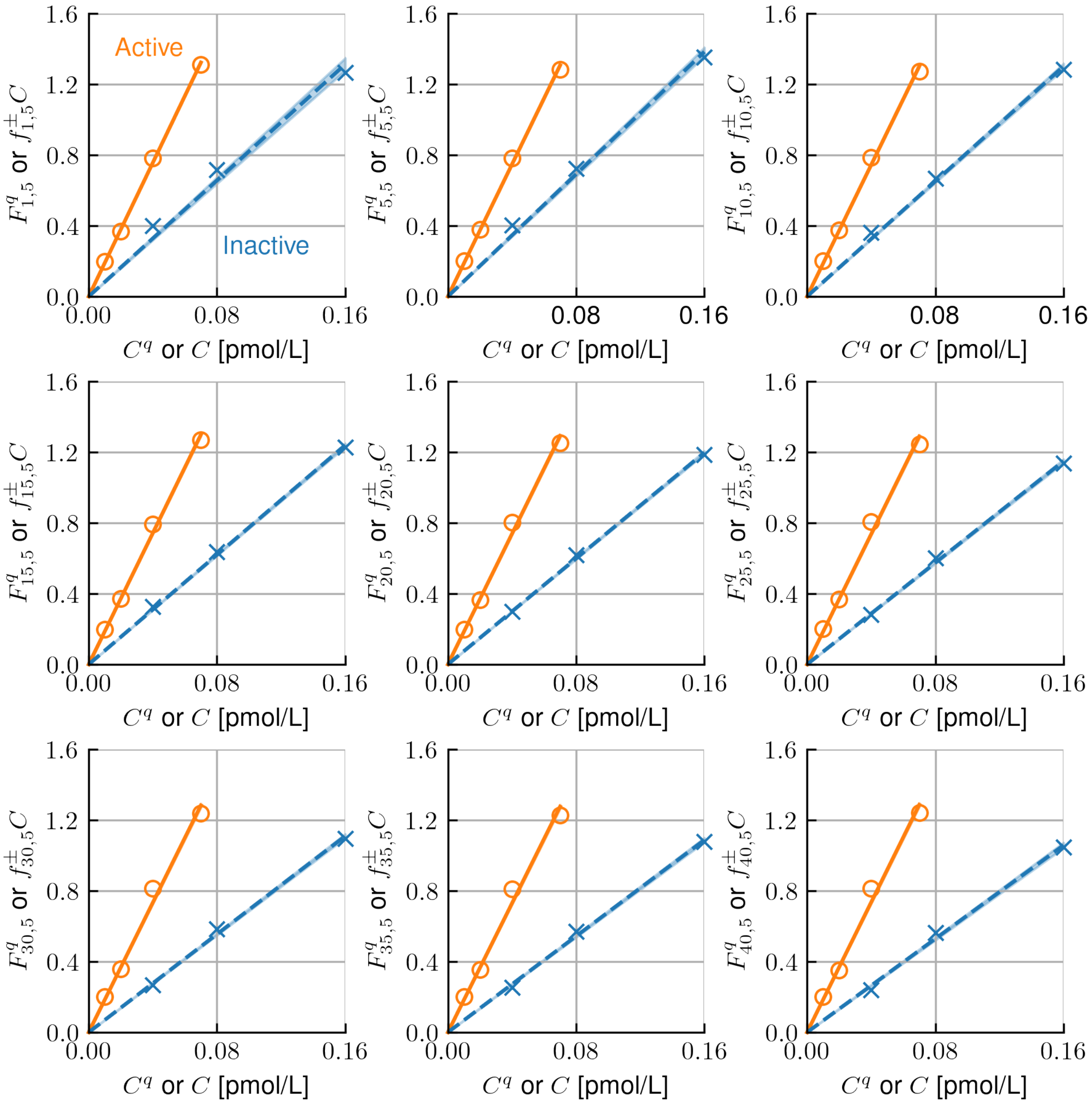

Fig. S5: As Figure S1 with well $w = 5$ (or A5).

Table S5: Molar Fluorescence Parameters for Well A5 ($w = 5$)

Cycle	Inactive		Active	
i	$f_{i,5}^{-}$	$\sigma_{i,5}^{-}$	$f_{i,5}^{+}$	$\sigma_{i,5}^{+}$
1	8.20	0.073	18.90	0.019
2	8.41	0.063	18.40	0.022
3	8.66	0.056	18.55	0.023
4	8.70	0.054	18.62	0.025
5	8.64	0.052	18.67	0.026
6	8.56	0.050	18.67	0.027
7	8.46	0.046	18.69	0.028
8	8.35	0.043	18.63	0.029
9	8.24	0.039	18.62	0.030
10	8.14	0.032	18.59	0.030
11	8.04	0.029	18.57	0.031
12	7.96	0.027	18.55	0.029
13	7.89	0.024	18.55	0.030
14	7.82	0.021	18.58	0.033
15	7.75	0.019	18.56	0.035
16	7.70	0.017	18.55	0.038
17	7.66	0.017	18.54	0.039
18	7.58	0.018	18.51	0.043
19	7.52	0.019	18.51	0.043
20	7.49	0.017	18.45	0.045
21	7.43	0.018	18.43	0.046
22	7.40	0.017	18.41	0.046
23	7.31	0.019	18.44	0.047
24	7.23	0.021	18.38	0.048
25	7.18	0.021	18.41	0.049
26	7.12	0.022	18.40	0.050
27	7.07	0.024	18.45	0.049
28	7.04	0.022	18.38	0.052
29	6.99	0.023	18.35	0.052
30	6.93	0.023	18.33	0.055
31	6.89	0.025	18.29	0.056
32	6.86	0.024	18.26	0.053
33	6.81	0.026	18.22	0.055
34	6.82	0.027	18.21	0.056
35	6.80	0.025	18.23	0.056
36	6.78	0.025	18.19	0.057
37	6.75	0.031	18.27	0.054
38	6.72	0.038	18.21	0.056
39	6.67	0.035	18.20	0.055
40	6.61	0.032	18.37	0.054
41	6.57	0.033	18.18	0.057
42	6.51	0.028	18.18	0.056
43	6.49	0.026	18.27	0.051
44	6.46	0.030	18.07	0.056
45	6.43	0.030	18.01	0.059

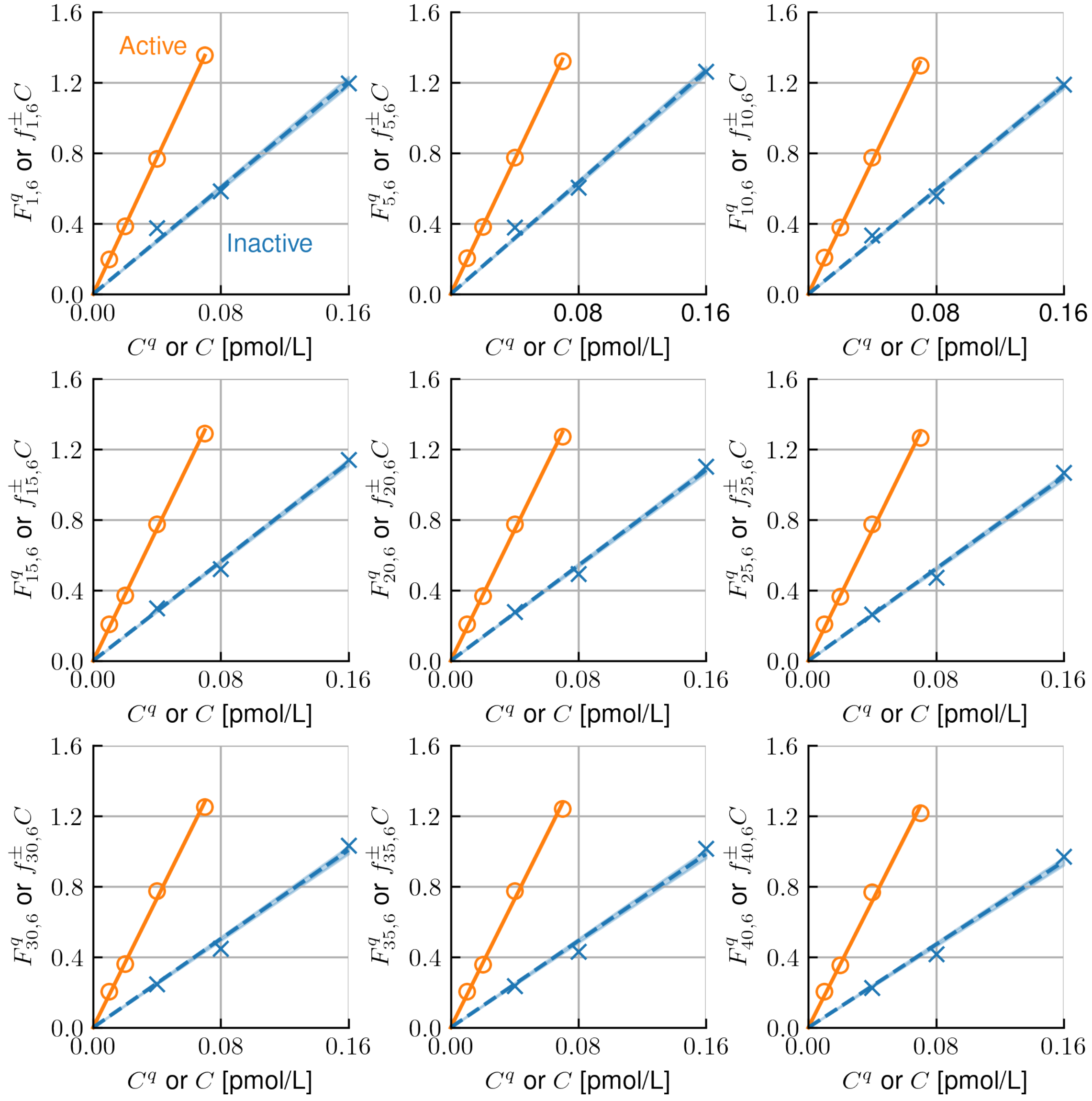

Fig. S6: As Figure S1 with well $w = 6$ (or A6).

Table S6: Molar Fluorescence Parameters for Well A6 ($w = 6$)

Cycle	Inactive		Active	
i	$f_{i,6}^{-}$	$\sigma_{i,6}^{-}$	$f_{i,6}^{+}$	$\sigma_{i,6}^{+}$
1	7.54	0.055	19.323	0.0042
2	7.77	0.048	18.772	0.0097
3	7.93	0.048	18.93	0.010
4	7.96	0.048	19.02	0.013
5	7.91	0.048	19.03	0.014
6	7.82	0.047	18.99	0.015
7	7.70	0.044	18.92	0.017
8	7.60	0.041	18.86	0.019
9	7.51	0.040	18.85	0.020
10	7.39	0.038	18.79	0.021
11	7.31	0.036	18.77	0.021
12	7.24	0.036	18.75	0.023
13	7.18	0.035	18.74	0.023
14	7.09	0.034	18.66	0.025
15	7.04	0.034	18.69	0.024
16	6.97	0.033	18.61	0.025
17	6.91	0.034	18.62	0.027
18	6.85	0.035	18.57	0.027
19	6.79	0.035	18.55	0.028
20	6.76	0.037	18.52	0.027
21	6.71	0.036	18.53	0.028
22	6.66	0.036	18.60	0.030
23	6.62	0.037	18.49	0.030
24	6.57	0.040	18.46	0.030
25	6.53	0.038	18.43	0.030
26	6.56	0.046	18.41	0.031
27	6.57	0.050	18.37	0.031
28	6.43	0.046	18.36	0.031
29	6.33	0.042	18.33	0.032
30	6.29	0.043	18.30	0.033
31	6.27	0.045	18.28	0.032
32	6.22	0.045	18.23	0.035
33	6.24	0.050	18.23	0.036
34	6.16	0.047	18.21	0.036
35	6.15	0.048	18.18	0.037
36	6.03	0.044	18.26	0.045
37	6.00	0.043	18.00	0.039
38	5.95	0.041	17.96	0.042
39	5.92	0.043	17.89	0.040
40	5.89	0.044	17.90	0.040
41	5.85	0.044	17.86	0.040
42	5.84	0.045	17.88	0.039
43	5.80	0.044	17.83	0.040
44	5.77	0.044	17.81	0.037
45	5.76	0.046	17.81	0.038

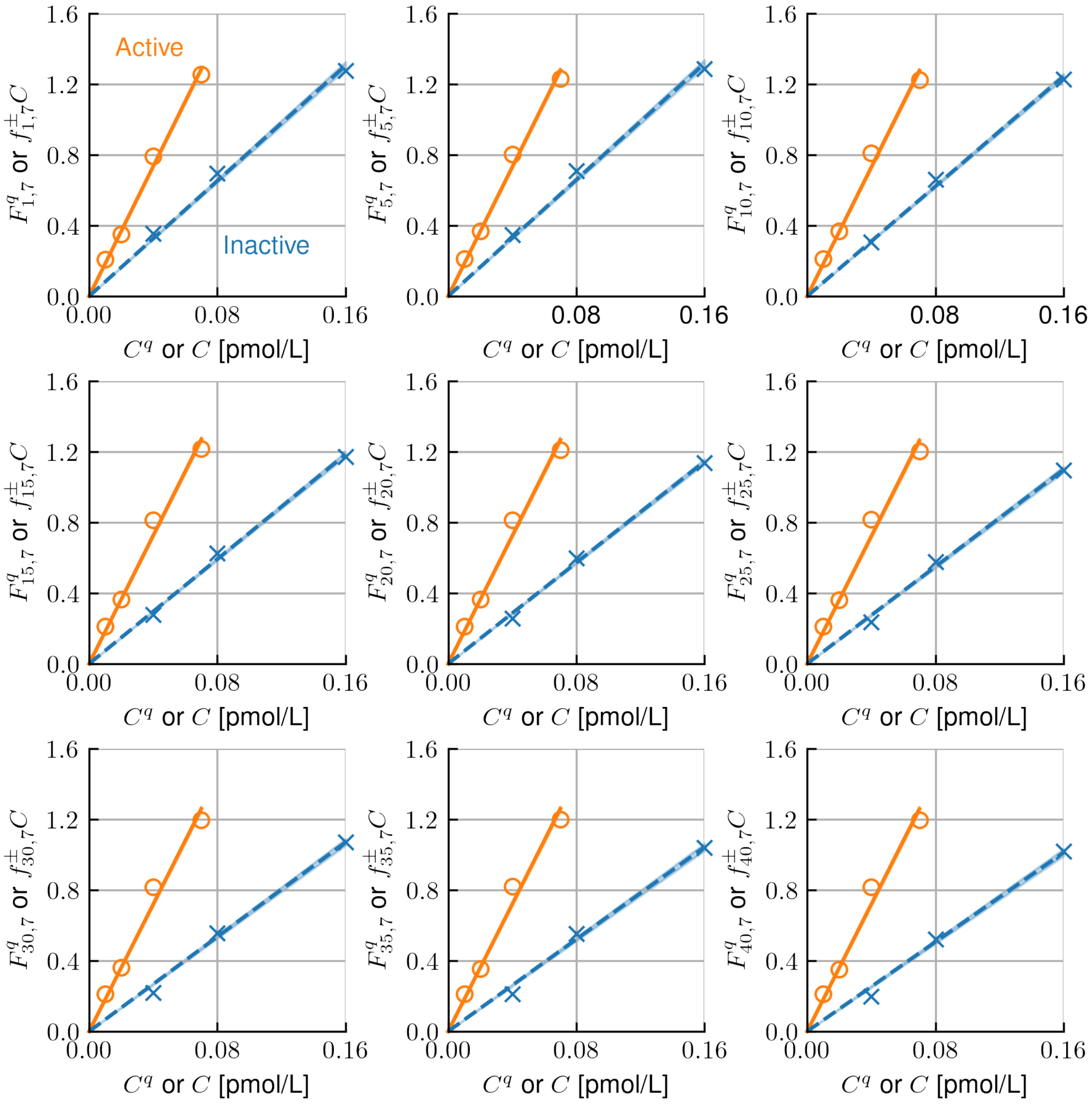

Fig. S7: As Figure S1 with well $w = 7$ (or A7).

Table S7: Molar Fluorescence Parameters for Well A7 ($w = 7$)

Cycle	Inactive		Active	
i	$f^{-}_{i,7}$	$\sigma^{-}_{i,7}$	$f^{+}_{i,7}$	$\sigma^{+}_{i,7}$
1	8.16	0.041	18.37	0.042
2	8.16	0.042	18.05	0.046
3	8.33	0.041	18.17	0.049
4	8.32	0.043	18.23	0.051
5	8.24	0.043	18.27	0.053
6	8.14	0.041	18.29	0.055
7	8.05	0.037	18.27	0.056
8	7.95	0.034	18.24	0.057
9	7.87	0.032	18.25	0.058
10	7.78	0.030	18.23	0.057
11	7.69	0.029	18.23	0.059
12	7.62	0.029	18.20	0.059
13	7.54	0.028	18.18	0.060
14	7.50	0.027	18.15	0.061
15	7.41	0.029	18.15	0.062
16	7.35	0.030	18.19	0.062
17	7.28	0.028	18.16	0.063
18	7.32	0.027	18.17	0.063
19	7.24	0.026	18.16	0.065
20	7.16	0.027	18.10	0.065
21	7.07	0.029	18.11	0.066
22	7.00	0.032	18.12	0.066
23	6.96	0.031	18.03	0.068
24	6.89	0.032	18.04	0.068
25	6.87	0.034	18.03	0.068
26	6.83	0.033	18.07	0.077
27	6.78	0.030	18.03	0.072
28	6.76	0.032	18.04	0.074
29	6.75	0.035	18.03	0.068
30	6.69	0.037	17.99	0.069
31	6.62	0.038	17.98	0.070
32	6.61	0.041	17.96	0.071
33	6.57	0.039	17.94	0.072
34	6.53	0.036	18.08	0.068
35	6.52	0.042	18.00	0.071
36	6.45	0.039	17.88	0.075
37	6.41	0.040	17.87	0.072
38	6.36	0.038	17.91	0.079
39	6.30	0.038	17.84	0.076
40	6.33	0.040	17.97	0.070
41	6.28	0.041	17.93	0.072
42	6.19	0.039	17.80	0.075
43	6.18	0.041	17.84	0.075
44	6.15	0.040	17.73	0.079
45	6.17	0.043	17.71	0.079

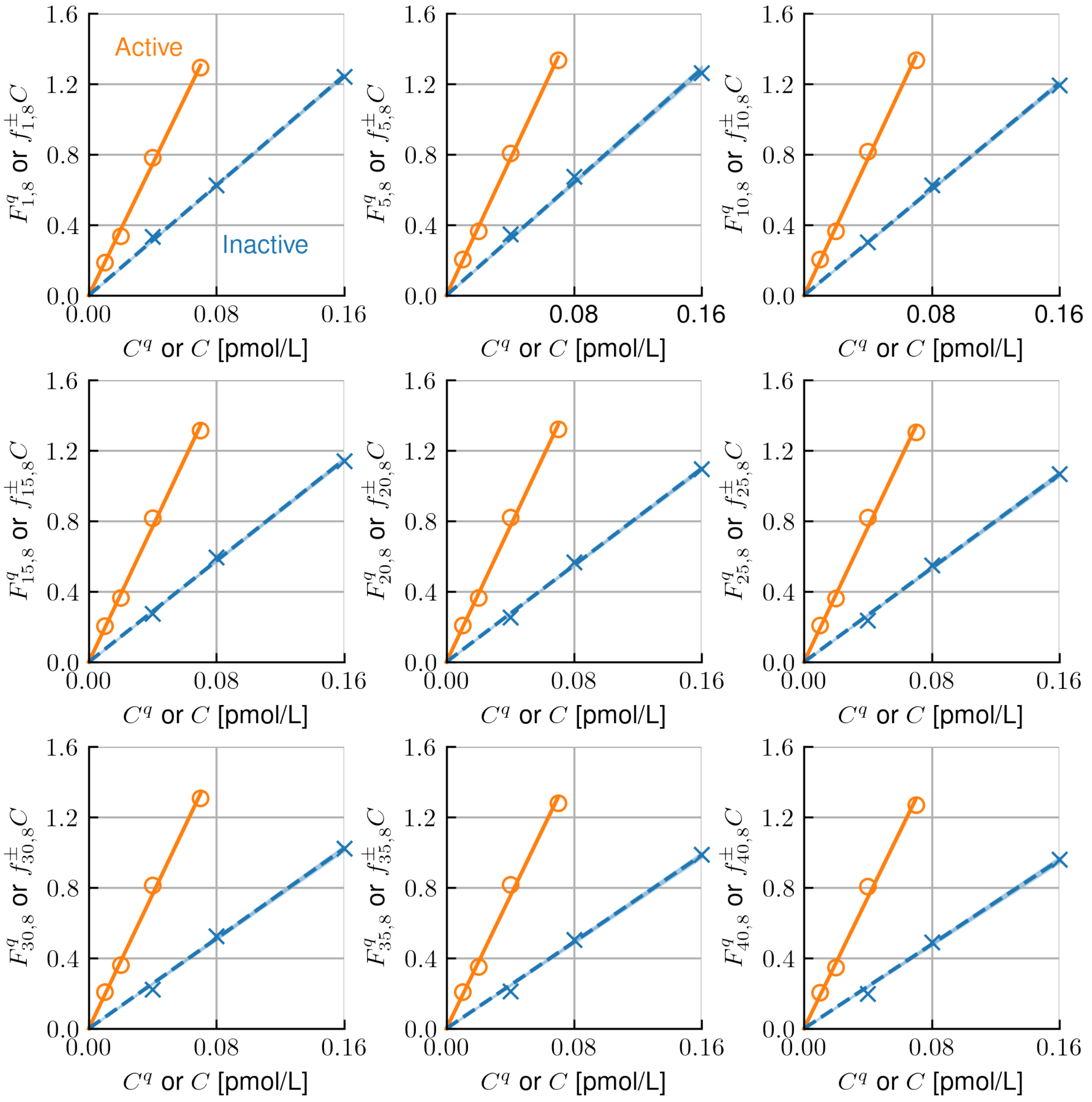

Fig. S8: As Figure S1 with well $w = 8$ (or A8).

Table S8: Molar Fluorescence Parameters for Well A8 ($w = 8$)

Cycle	Inactive		Active	
i	$f^-_{i,8}$	$\sigma^-_{i,8}$	$f^+_{i,8}$	$\sigma^+_{i,8}$
1	7.80	0.015	18.64	0.030
2	7.92	0.031	18.84	0.023
3	8.08	0.034	19.11	0.026
4	8.09	0.035	19.25	0.026
5	8.03	0.034	19.32	0.026
6	7.92	0.032	19.38	0.027
7	7.82	0.029	19.38	0.028
8	7.72	0.025	19.36	0.030
9	7.63	0.022	19.29	0.028
10	7.53	0.020	19.36	0.030
11	7.43	0.019	19.32	0.030
12	7.39	0.018	19.26	0.031
13	7.31	0.017	19.23	0.032
14	7.24	0.017	19.19	0.035
15	7.18	0.018	19.18	0.036
16	7.15	0.016	19.22	0.034
17	7.08	0.017	19.18	0.035
18	7.00	0.018	19.17	0.038
19	6.98	0.017	19.24	0.036
20	6.86	0.019	19.22	0.036
21	6.82	0.020	19.15	0.038
22	6.77	0.020	19.12	0.039
23	6.71	0.021	19.13	0.039
24	6.66	0.022	19.09	0.042
25	6.66	0.025	19.08	0.041
26	6.59	0.029	19.07	0.042
27	6.51	0.026	19.06	0.040
28	6.47	0.026	19.04	0.042
29	6.48	0.029	19.17	0.037
30	6.39	0.026	19.05	0.038
31	6.37	0.029	19.19	0.035
32	6.28	0.027	18.92	0.045
33	6.25	0.027	18.84	0.045
34	6.20	0.029	18.78	0.045
35	6.15	0.027	18.77	0.048
36	6.13	0.027	18.74	0.049
37	6.10	0.027	18.72	0.047
38	6.05	0.028	18.69	0.048
39	6.02	0.029	18.67	0.048
40	5.98	0.030	18.60	0.045
41	5.97	0.029	18.49	0.046
42	5.96	0.030	18.49	0.046
43	5.95	0.031	18.53	0.046
44	5.93	0.032	18.49	0.046
45	5.91	0.033	18.47	0.047

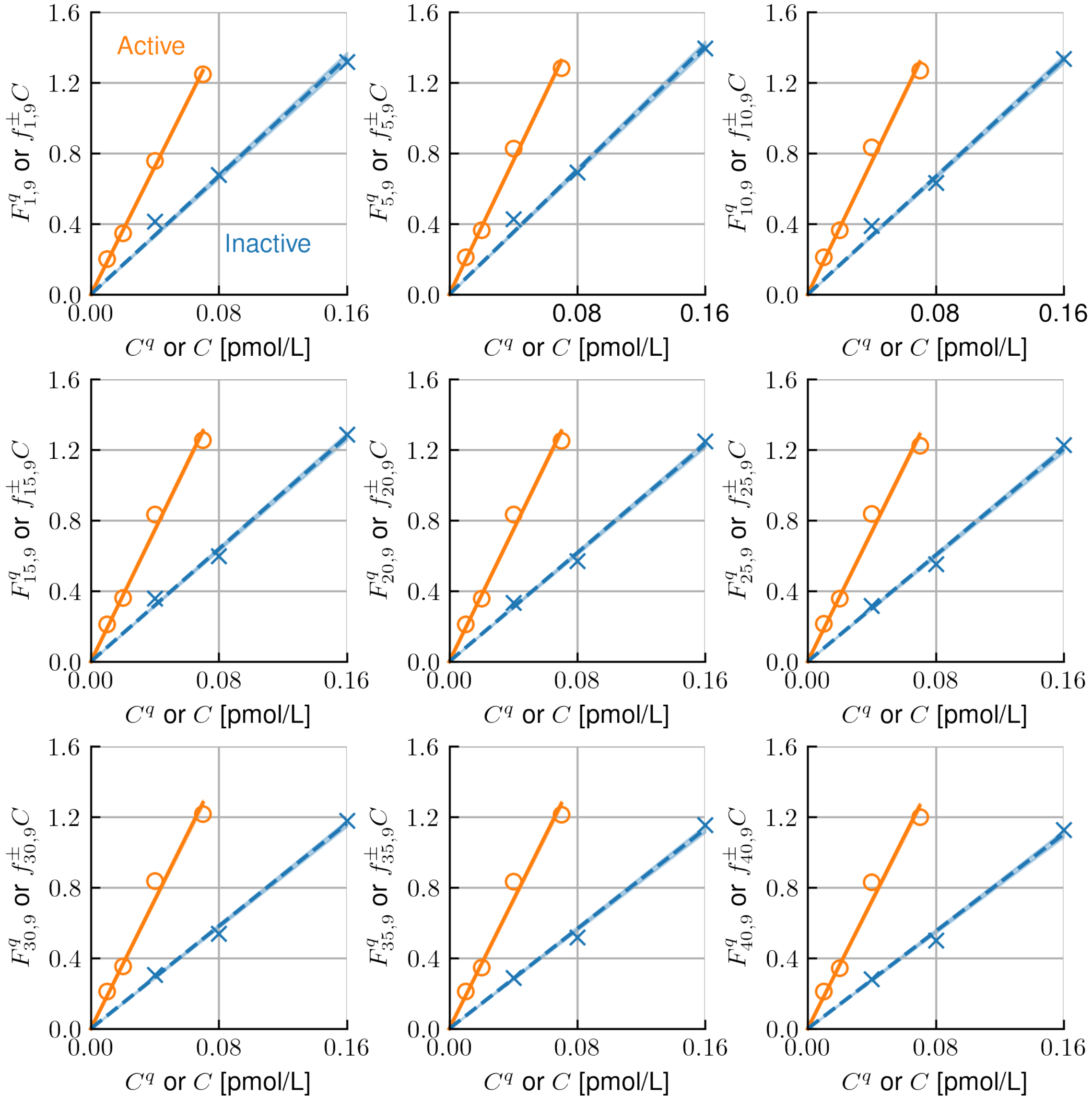

Fig. S9: As Figure S1 with well $w = 9$ (or A9).

Table S9: Molar Fluorescence Parameters for Well A9 ($w = 9$)

Cycle	Inactive		Active	
i	$f^-_{i,9}$	$\sigma^-_{i,9}$	$f^+_{i,9}$	$\sigma^+_{i,9}$
1	8.38	0.059	18.10	0.027
2	8.64	0.054	18.39	0.041
3	8.83	0.054	18.69	0.045
4	8.87	0.055	18.85	0.048
5	8.80	0.054	18.91	0.051
6	8.72	0.052	18.90	0.053
7	8.63	0.049	18.86	0.055
8	8.50	0.048	18.84	0.056
9	8.42	0.047	18.82	0.057
10	8.33	0.046	18.80	0.057
11	8.24	0.044	18.75	0.059
12	8.16	0.043	18.74	0.059
13	8.10	0.042	18.69	0.060
14	8.01	0.040	18.67	0.061
15	7.97	0.040	18.66	0.062
16	7.93	0.041	18.61	0.063
17	7.85	0.038	18.63	0.063
18	7.80	0.039	18.62	0.063
19	7.74	0.039	18.62	0.063
20	7.70	0.038	18.63	0.062
21	7.67	0.038	18.51	0.066
22	7.60	0.037	18.49	0.067
23	7.58	0.038	18.43	0.069
24	7.54	0.039	18.38	0.071
25	7.53	0.040	18.37	0.072
26	7.43	0.037	18.35	0.072
27	7.39	0.036	18.32	0.073
28	7.35	0.038	18.33	0.072
29	7.32	0.034	18.31	0.074
30	7.27	0.035	18.28	0.074
31	7.20	0.037	18.31	0.074
32	7.18	0.039	18.28	0.074
33	7.15	0.040	18.25	0.073
34	7.12	0.041	18.25	0.073
35	7.08	0.039	18.21	0.074
36	7.06	0.039	18.16	0.075
37	6.97	0.038	18.14	0.075
38	6.96	0.041	18.09	0.077
39	6.93	0.040	18.06	0.076
40	6.89	0.039	18.04	0.077
41	6.91	0.041	17.87	0.082
42	6.89	0.041	17.92	0.080
43	6.86	0.040	17.90	0.082
44	6.87	0.039	17.76	0.071
45	6.82	0.038	17.76	0.073

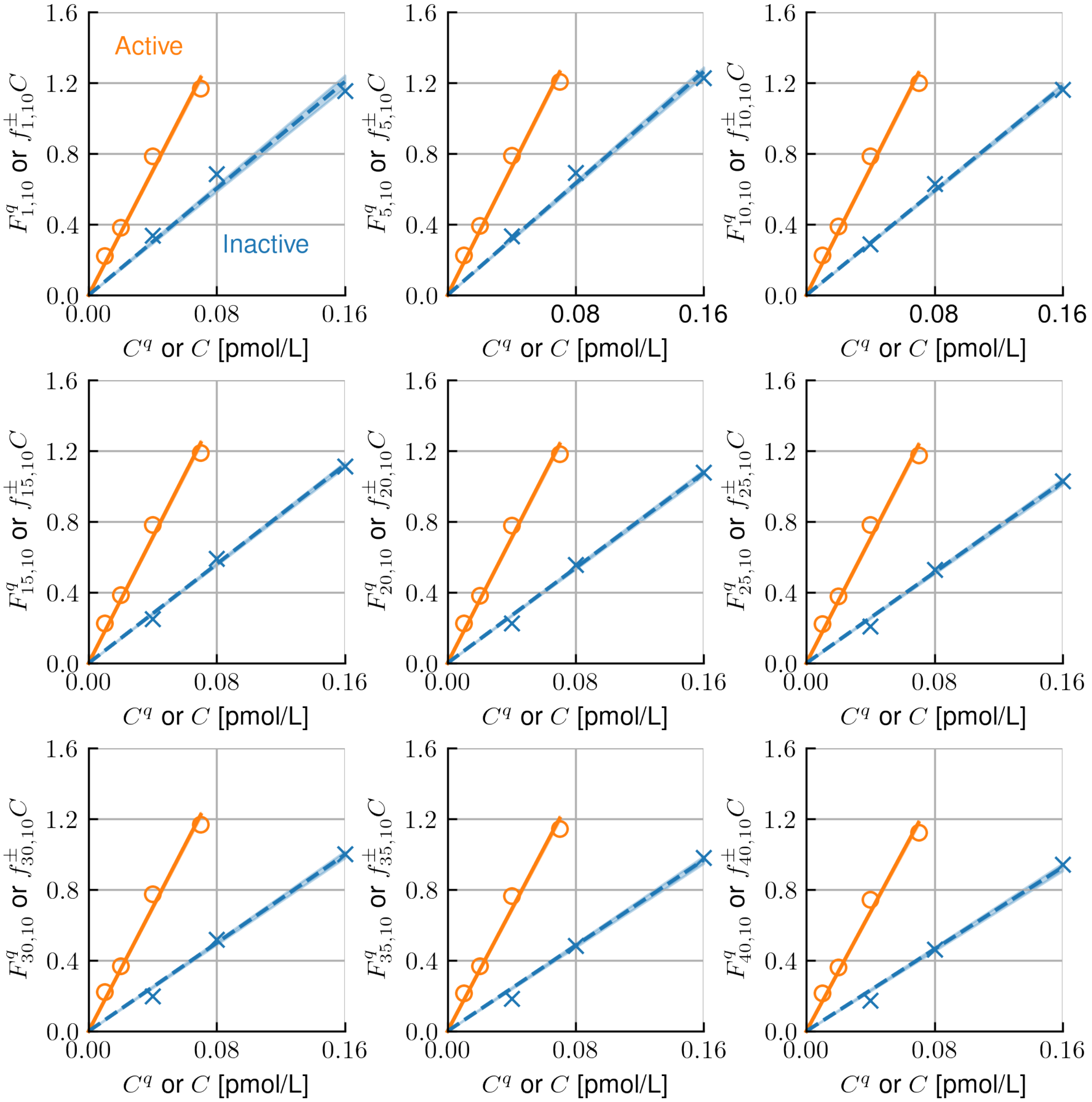

Fig. S10: As Figure S1 with well $w = 10$ (or A10).

Table S10: Molar Fluorescence Parameters for Well A10 ($w = 10$)

Cycle	Inactive		Active	
i	$f^-_{i,10}$	$\sigma^-_{i,10}$	$f^+_{i,10}$	$\sigma^+_{i,10}$
1	7.54	0.073	17.57	0.067
2	7.82	0.066	17.55	0.061
3	7.99	0.064	17.83	0.062
4	7.97	0.058	17.94	0.061
5	7.89	0.051	18.00	0.060
6	7.79	0.045	18.00	0.060
7	7.68	0.041	17.98	0.061
8	7.57	0.037	17.94	0.061
9	7.48	0.034	17.94	0.061
10	7.38	0.031	17.92	0.061
11	7.31	0.030	17.88	0.062
12	7.24	0.028	17.86	0.062
13	7.18	0.028	17.85	0.062
14	7.10	0.028	17.82	0.062
15	7.01	0.029	17.79	0.061
16	6.95	0.030	17.79	0.062
17	6.94	0.030	17.76	0.062
18	6.87	0.030	17.70	0.062
19	6.78	0.032	17.73	0.062
20	6.73	0.032	17.68	0.062
21	6.72	0.039	17.68	0.061
22	6.60	0.033	17.76	0.058
23	6.54	0.034	17.63	0.061
24	6.48	0.035	17.62	0.061
25	6.41	0.035	17.61	0.064
26	6.36	0.035	17.57	0.062
27	6.32	0.034	17.55	0.062
28	6.29	0.033	17.51	0.063
29	6.26	0.036	17.46	0.062
30	6.24	0.040	17.48	0.062
31	6.20	0.038	17.31	0.066
32	6.16	0.039	17.25	0.066
33	6.11	0.040	17.23	0.065
34	6.11	0.041	17.20	0.064
35	6.05	0.042	17.18	0.064
36	6.02	0.042	17.13	0.063
37	5.90	0.040	17.12	0.064
38	5.86	0.041	16.94	0.054
39	5.82	0.042	16.97	0.056
40	5.79	0.043	16.83	0.060
41	5.71	0.042	16.77	0.062
42	5.67	0.043	16.78	0.062
43	5.63	0.044	16.78	0.062
44	5.60	0.045	16.76	0.062
45	5.57	0.046	16.75	0.062

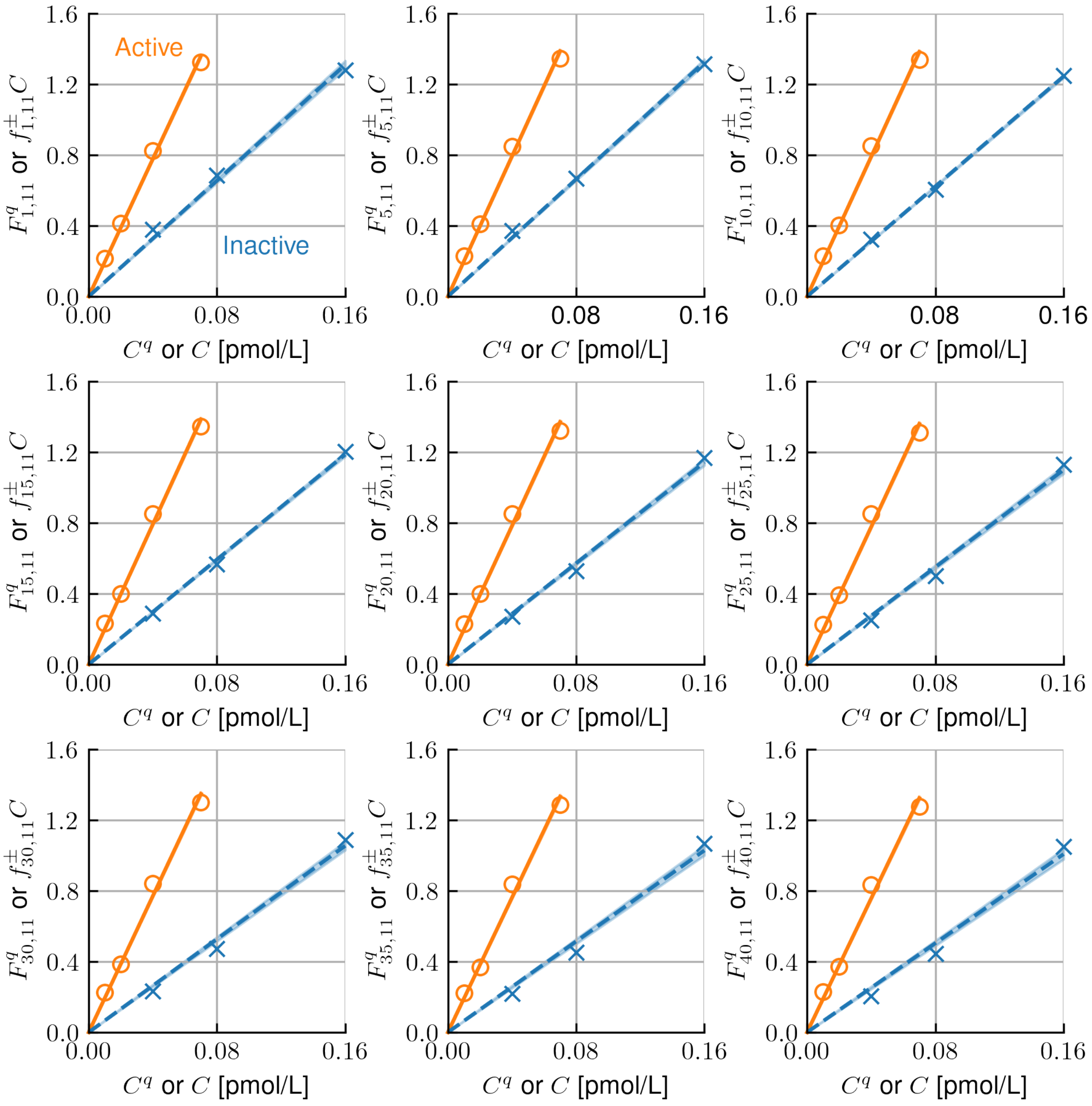

Fig. S11: As Figure S1 with well $w = 11$ (or A11).

Table S11: Molar Fluorescence Parameters for Well A11 ($w = 11$)

Cycle	Inactive		Active	
i	$f^-_{i,11}$	$\sigma^-_{i,11}$	$f^+_{i,11}$	$\sigma^+_{i,11}$
1	8.19	0.048	19.43	0.038
2	8.34	0.043	19.38	0.036
3	8.42	0.041	19.61	0.040
4	8.39	0.036	19.78	0.044
5	8.29	0.031	19.82	0.046
6	8.17	0.025	19.81	0.046
7	8.07	0.021	19.81	0.048
8	7.97	0.018	19.78	0.048
9	7.88	0.016	19.75	0.049
10	7.78	0.016	19.75	0.049
11	7.70	0.016	19.78	0.048
12	7.63	0.019	19.73	0.050
13	7.56	0.021	19.68	0.050
14	7.49	0.023	19.65	0.051
15	7.42	0.023	19.79	0.046
16	7.34	0.024	19.68	0.050
17	7.35	0.033	19.65	0.051
18	7.26	0.033	19.77	0.047
19	7.20	0.033	19.62	0.052
20	7.15	0.037	19.58	0.053
21	7.09	0.039	19.54	0.055
22	7.02	0.039	19.48	0.056
23	6.94	0.039	19.53	0.058
24	6.89	0.044	19.49	0.057
25	6.87	0.045	19.43	0.056
26	6.78	0.043	19.50	0.061
27	6.70	0.043	19.39	0.051
28	6.67	0.043	19.40	0.050
29	6.61	0.049	19.36	0.051
30	6.59	0.050	19.26	0.053
31	6.58	0.051	19.12	0.056
32	6.53	0.053	19.12	0.056
33	6.50	0.056	19.11	0.055
34	6.50	0.060	19.12	0.055
35	6.43	0.058	19.04	0.056
36	6.39	0.057	19.08	0.059
37	6.35	0.058	19.03	0.057
38	6.33	0.058	19.07	0.054
39	6.30	0.059	18.97	0.057
40	6.30	0.061	18.92	0.057
41	6.25	0.060	18.89	0.058
42	6.22	0.060	18.74	0.061
43	6.17	0.061	18.81	0.060
44	6.17	0.065	18.81	0.060
45	6.15	0.065	18.78	0.062

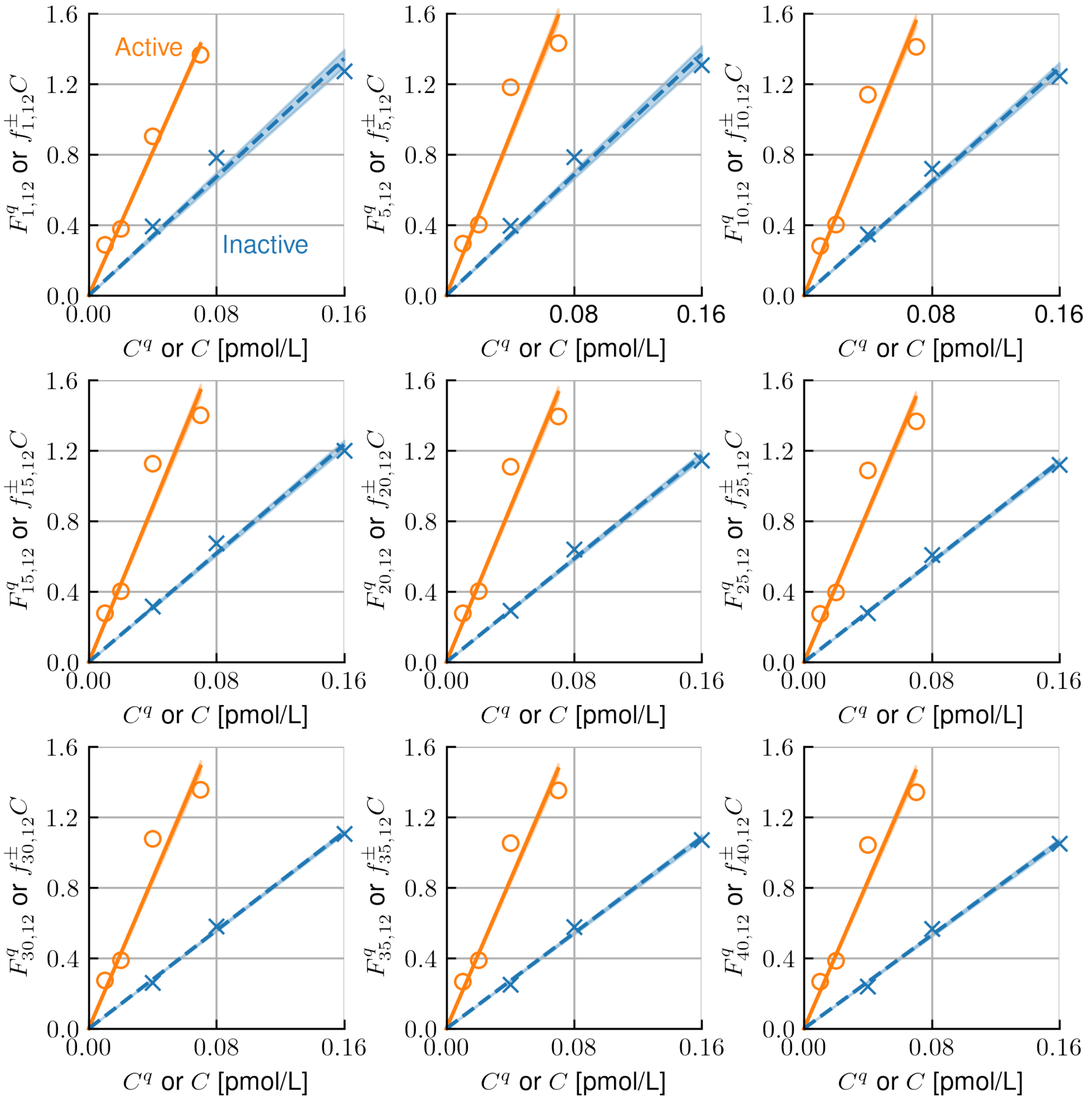

Fig. S12: As Figure S1 with well $w = 12$ (or A12).

Table S12: Molar Fluorescence Parameters for Well A12 ($w = 12$)

Cycle	Inactive		Active	
i	$f^-_{i,12}$	$\sigma^-_{i,12}$	$f^+_{i,12}$	$\sigma^+_{i,12}$
1	8.4	0.10	20.35	0.081
2	8.6	0.10	21.0	0.10
3	8.7	0.10	22.2	0.17
4	8.64	0.100	22.5	0.19
5	8.56	0.092	22.6	0.19
6	8.44	0.086	22.5	0.18
7	8.34	0.079	22.4	0.18
8	8.24	0.073	22.3	0.17
9	8.15	0.069	22.3	0.17
10	8.06	0.064	22.2	0.17
11	7.98	0.060	22.1	0.17
12	7.90	0.057	22.1	0.17
13	7.83	0.053	22.1	0.17
14	7.78	0.050	22.0	0.17
15	7.70	0.048	22.0	0.17
16	7.64	0.046	22.0	0.17
17	7.54	0.047	22.0	0.17
18	7.48	0.044	22.0	0.16
19	7.44	0.047	21.9	0.16
20	7.32	0.042	21.8	0.16
21	7.30	0.042	21.8	0.16
22	7.23	0.037	21.8	0.16
23	7.21	0.034	21.7	0.16
24	7.15	0.032	21.6	0.16
25	7.11	0.031	21.4	0.16
26	7.07	0.030	21.3	0.16
27	7.07	0.027	21.3	0.16
28	7.01	0.027	21.3	0.15
29	7.01	0.024	21.2	0.15
30	6.97	0.021	21.2	0.16
31	6.95	0.029	21.2	0.15
32	6.89	0.030	21.1	0.15
33	6.85	0.030	21.1	0.15
34	6.81	0.029	21.0	0.14
35	6.77	0.030	21.0	0.15
36	6.73	0.033	21.0	0.15
37	6.72	0.030	21.0	0.14
38	6.70	0.031	21.0	0.14
39	6.65	0.031	20.9	0.14
40	6.63	0.032	20.9	0.14
41	6.44	0.029	20.7	0.13
42	6.43	0.034	20.5	0.14
43	6.39	0.030	20.6	0.14
44	6.36	0.029	20.6	0.14
45	6.35	0.026	20.6	0.14

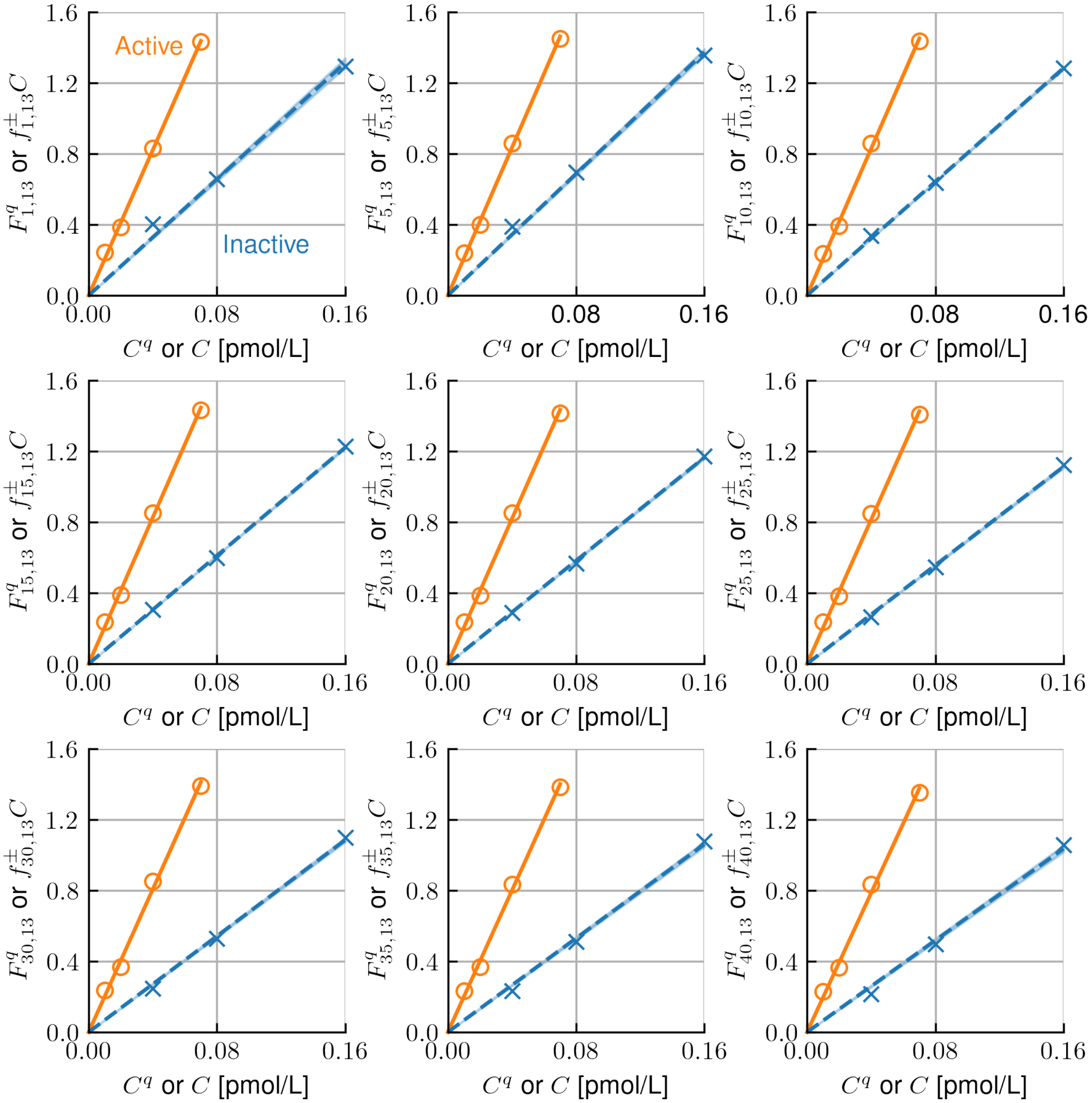

Fig. S13: As Figure S1 with well $w = 13$ (or B1).

Table S13: Molar Fluorescence Parameters for Well B1 ($w = 13$)

Cycle	Inactive		Active	
i	$f^-_{i,13}$	$\sigma^-_{i,13}$	$f^+_{i,13}$	$\sigma^+_{i,13}$
1	8.21	0.054	20.53	0.026
2	8.58	0.048	20.57	0.023
3	8.75	0.046	20.78	0.025
4	8.70	0.041	20.86	0.025
5	8.58	0.036	20.88	0.026
6	8.45	0.030	20.84	0.025
7	8.33	0.024	20.77	0.028
8	8.24	0.019	20.80	0.027
9	8.12	0.015	20.75	0.027
10	8.03	0.013	20.73	0.029
11	7.93	0.010	20.65	0.029
12	7.844	0.0098	20.68	0.027
13	7.76	0.011	20.63	0.028
14	7.695	0.0096	20.59	0.029
15	7.63	0.010	20.66	0.028
16	7.59	0.014	20.62	0.032
17	7.51	0.015	20.67	0.033
18	7.40	0.013	20.55	0.031
19	7.36	0.014	20.51	0.031
20	7.28	0.013	20.49	0.032
21	7.24	0.015	20.47	0.032
22	7.23	0.020	20.46	0.032
23	7.16	0.026	20.45	0.031
24	7.01	0.015	20.41	0.032
25	6.96	0.015	20.37	0.033
26	6.92	0.015	20.35	0.033
27	6.88	0.018	20.36	0.033
28	6.85	0.021	20.29	0.035
29	6.82	0.021	20.22	0.037
30	6.78	0.023	20.18	0.039
31	6.75	0.024	20.15	0.038
32	6.70	0.025	20.14	0.040
33	6.68	0.027	20.03	0.035
34	6.66	0.028	20.00	0.035
35	6.63	0.030	20.01	0.035
36	6.60	0.032	20.01	0.035
37	6.57	0.034	19.68	0.037
38	6.54	0.038	19.80	0.036
39	6.51	0.039	19.73	0.038
40	6.48	0.039	19.65	0.040
41	6.44	0.044	19.66	0.039
42	6.42	0.045	19.69	0.038
43	6.44	0.049	19.68	0.039
44	6.24	0.041	19.66	0.039
45	6.18	0.040	19.63	0.037

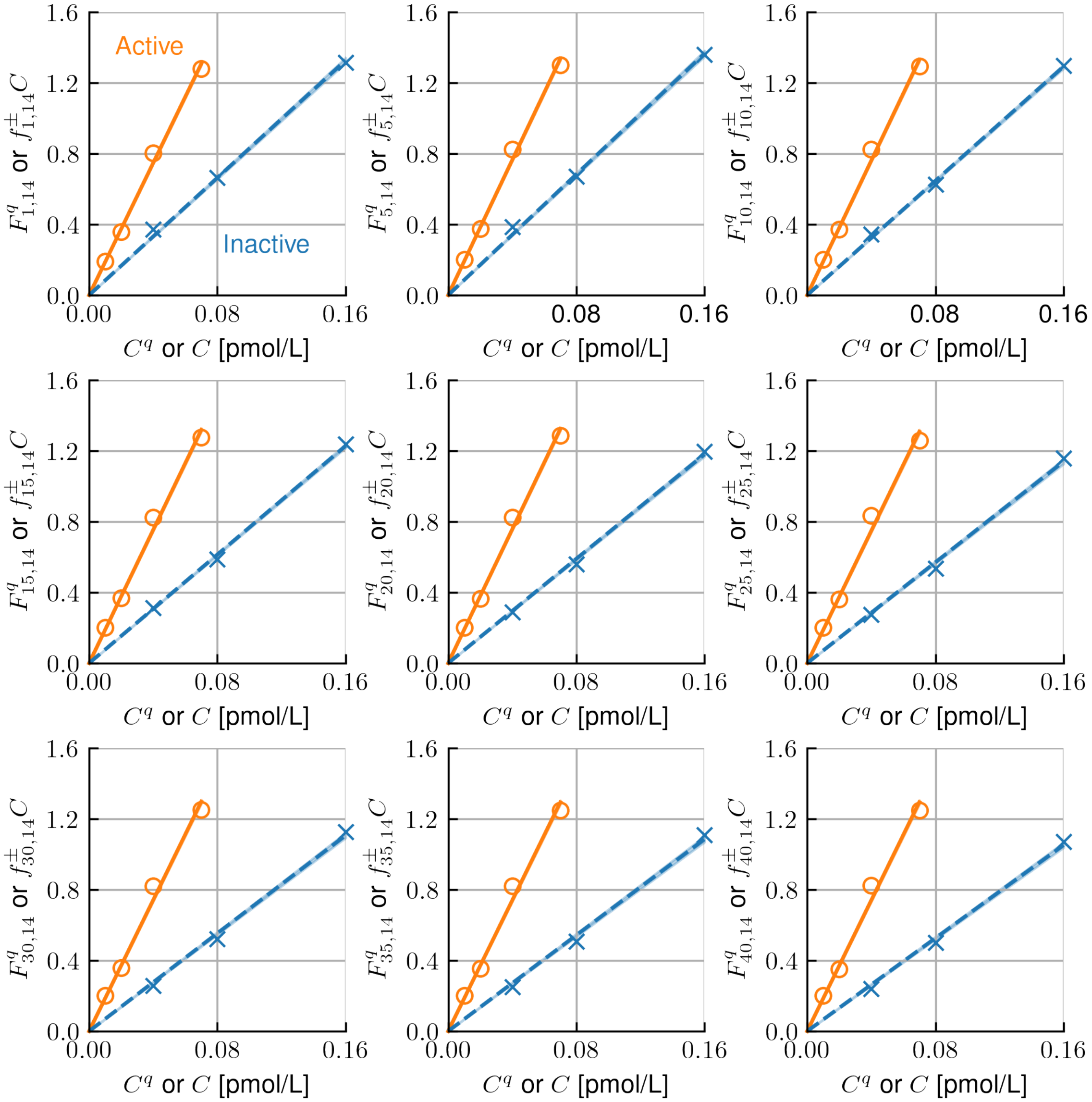

Fig. S14: As Figure S1 with well $w = 14$ (or B2).

Table S14: Molar Fluorescence Parameters for Well B2 ($w = 14$)

Cycle	Inactive		Active	
i	$f^{-}_{i,14}$	$\sigma^{-}_{i,14}$	$f^{+}_{i,14}$	$\sigma^{+}_{i,14}$
1	8.29	0.030	18.68	0.038
2	8.44	0.038	18.84	0.039
3	8.60	0.037	19.03	0.039
4	8.60	0.035	19.08	0.040
5	8.53	0.033	19.08	0.041
6	8.44	0.031	19.07	0.042
7	8.36	0.028	19.08	0.042
8	8.25	0.026	19.03	0.043
9	8.15	0.023	18.98	0.045
10	8.08	0.022	19.00	0.044
11	8.00	0.020	18.99	0.045
12	7.91	0.021	18.94	0.046
13	7.82	0.020	18.95	0.046
14	7.73	0.019	18.91	0.048
15	7.67	0.020	18.83	0.049
16	7.61	0.020	18.81	0.049
17	7.54	0.020	18.84	0.049
18	7.48	0.021	18.83	0.050
19	7.42	0.022	18.82	0.049
20	7.38	0.024	18.91	0.046
21	7.32	0.027	18.70	0.052
22	7.25	0.025	18.70	0.052
23	7.23	0.025	18.66	0.053
24	7.18	0.028	18.65	0.053
25	7.12	0.027	18.69	0.059
26	7.06	0.028	18.62	0.054
27	7.05	0.032	18.75	0.053
28	7.01	0.031	18.65	0.054
29	6.96	0.031	18.56	0.055
30	6.92	0.028	18.53	0.055
31	6.88	0.026	18.52	0.055
32	6.85	0.027	18.53	0.054
33	6.81	0.026	18.52	0.054
34	6.80	0.030	18.50	0.055
35	6.79	0.033	18.48	0.055
36	6.76	0.033	18.51	0.054
37	6.72	0.032	18.48	0.055
38	6.67	0.031	18.54	0.057
39	6.62	0.030	18.55	0.056
40	6.57	0.029	18.49	0.057
41	6.54	0.032	18.35	0.060
42	6.51	0.032	18.46	0.056
43	6.46	0.034	18.47	0.056
44	6.40	0.032	18.26	0.061
45	6.37	0.035	18.24	0.061

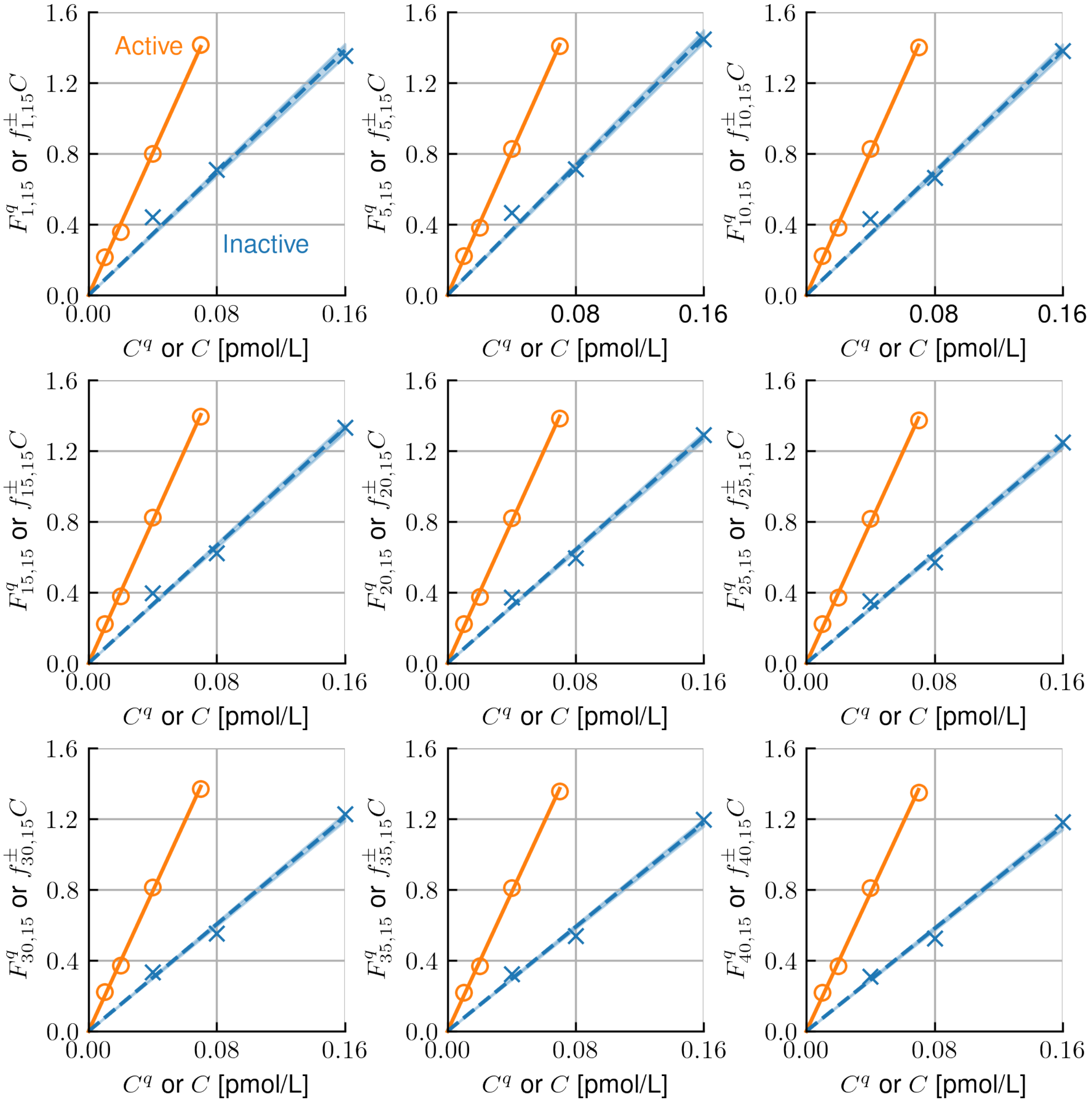

Fig. S15: As Figure S1 with well $w = 15$ (or B3).

Table S15: Molar Fluorescence Parameters for Well B3 ($w = 15$)

Cycle	Inactive		Active	
i	$f^-_{i,15}$	$\sigma^-_{i,15}$	$f^+_{i,15}$	$\sigma^+_{i,15}$
1	8.65	0.073	20.05	0.027
2	9.07	0.074	19.86	0.019
3	9.21	0.075	20.07	0.021
4	9.19	0.075	20.19	0.020
5	9.14	0.074	20.21	0.021
6	9.04	0.072	20.24	0.021
7	8.95	0.069	20.22	0.021
8	8.86	0.067	20.22	0.021
9	8.78	0.065	20.21	0.022
10	8.67	0.063	20.17	0.022
11	8.59	0.061	20.17	0.023
12	8.51	0.059	20.15	0.023
13	8.44	0.058	20.11	0.023
14	8.36	0.057	20.08	0.024
15	8.30	0.055	20.05	0.024
16	8.23	0.053	20.03	0.024
17	8.16	0.052	20.03	0.024
18	8.11	0.052	20.00	0.024
19	8.06	0.051	19.96	0.025
20	8.01	0.050	19.92	0.024
21	7.95	0.049	19.91	0.024
22	7.90	0.049	19.89	0.024
23	7.90	0.049	19.85	0.024
24	7.80	0.046	19.84	0.024
25	7.73	0.045	19.79	0.026
26	7.69	0.045	19.81	0.024
27	7.65	0.045	19.80	0.025
28	7.64	0.046	19.85	0.024
29	7.60	0.045	19.79	0.024
30	7.56	0.045	19.75	0.025
31	7.50	0.044	19.87	0.023
32	7.45	0.045	19.80	0.024
33	7.41	0.044	19.74	0.023
34	7.38	0.041	19.65	0.025
35	7.37	0.043	19.58	0.026
36	7.33	0.041	19.53	0.026
37	7.34	0.045	19.86	0.022
38	7.31	0.044	19.65	0.023
39	7.27	0.044	19.59	0.027
40	7.24	0.044	19.49	0.028
41	7.21	0.045	19.39	0.028
42	7.20	0.047	19.36	0.029
43	7.24	0.050	19.63	0.025
44	7.18	0.050	19.44	0.025
45	7.11	0.048	19.56	0.027

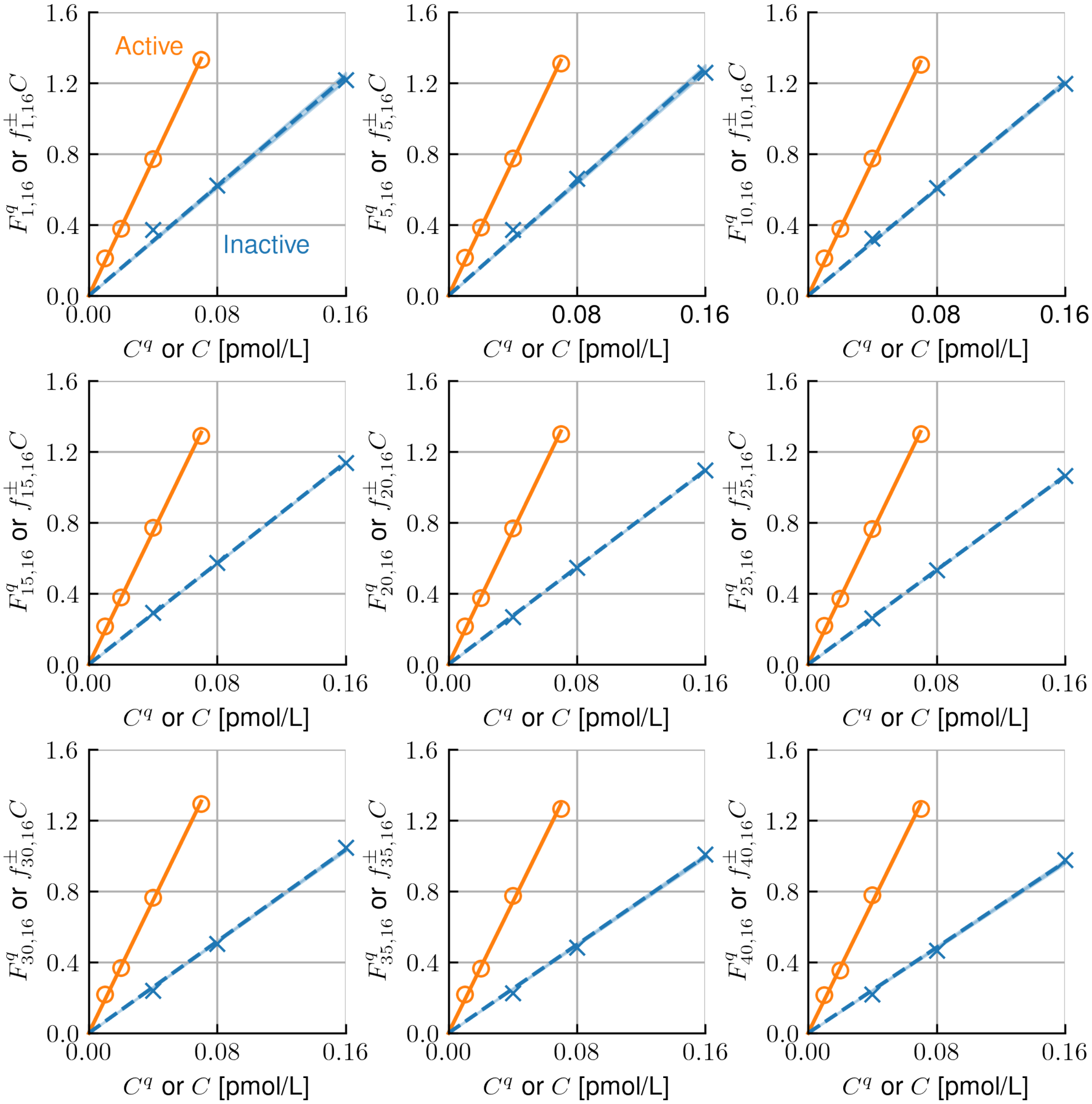

Fig. S16: As Figure S1 with well $w = 16$ (or B4).

Table S16: Molar Fluorescence Parameters for Well B4 ($w = 16$)

Cycle	Inactive		Active	
i	$f^-_{i,16}$	$\sigma^-_{i,16}$	$f^+_{i,16}$	$\sigma^+_{i,16}$
1	7.72	0.047	19.11	0.013
2	8.01	0.048	18.83	0.014
3	8.13	0.047	18.92	0.017
4	8.09	0.046	18.91	0.019
5	8.01	0.043	18.96	0.020
6	7.91	0.038	18.97	0.020
7	7.82	0.032	18.93	0.020
8	7.71	0.028	18.93	0.020
9	7.62	0.023	18.88	0.020
10	7.53	0.019	18.85	0.021
11	7.44	0.015	18.84	0.021
12	7.36	0.012	18.82	0.021
13	7.282	0.0095	18.76	0.022
14	7.195	0.0080	18.72	0.024
15	7.132	0.0054	18.69	0.024
16	7.051	0.0041	18.80	0.020
17	6.984	0.0032	18.84	0.019
18	6.953	0.0014	18.88	0.018
19	6.9002	0.00057	18.83	0.019
20	6.845	0.0040	18.79	0.020
21	6.815	0.0064	18.82	0.024
22	6.756	0.0082	18.78	0.022
23	6.714	0.0067	18.73	0.022
24	6.70	0.012	18.80	0.021
25	6.648	0.0048	18.74	0.022
26	6.601	0.0089	18.69	0.023
27	6.59	0.016	18.67	0.022
28	6.55	0.014	18.65	0.023
29	6.55	0.017	18.65	0.024
30	6.47	0.017	18.67	0.023
31	6.40	0.014	18.74	0.022
32	6.34	0.013	18.68	0.024
33	6.29	0.020	18.91	0.021
34	6.31	0.024	18.79	0.024
35	6.23	0.021	18.43	0.032
36	6.19	0.019	18.48	0.031
37	6.15	0.023	18.45	0.031
38	6.09	0.024	18.53	0.038
39	6.06	0.021	18.47	0.036
40	6.03	0.022	18.43	0.034
41	6.03	0.023	18.38	0.033
42	6.03	0.023	18.39	0.034
43	6.07	0.025	18.27	0.034
44	5.95	0.023	18.28	0.033
45	5.96	0.022	18.26	0.032

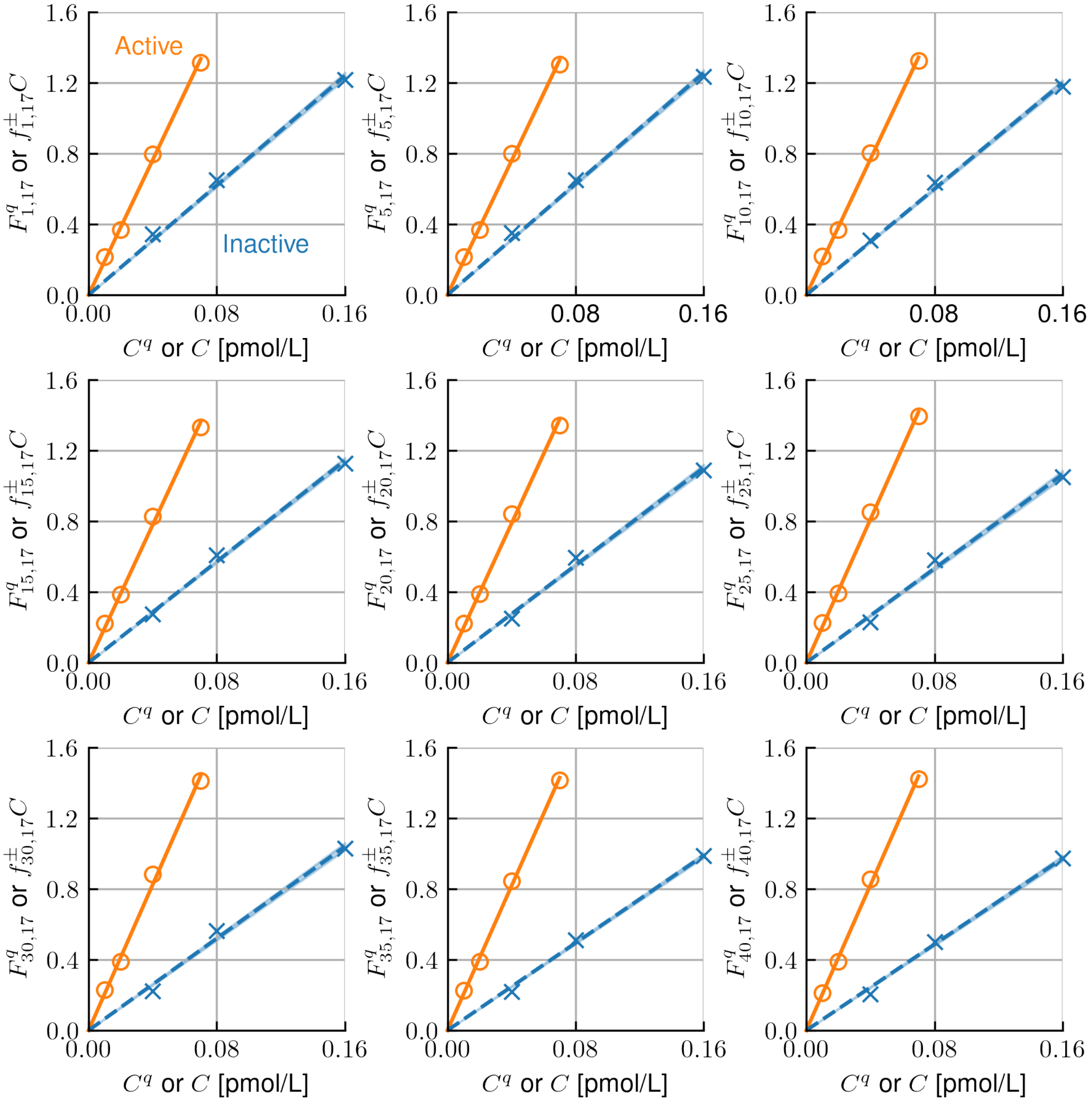

Fig. S17: As Figure S1 with well $w = 17$ (or B5).

Table S17: Molar Fluorescence Parameters for Well B5 ($w = 17$)

Cycle	Inactive		Active	
i	$f^-_{i,17}$	$\sigma^-_{i,17}$	$f^+_{i,17}$	$\sigma^+_{i,17}$
1	7.75	0.037	19.07	0.027
2	7.63	0.042	18.56	0.029
3	7.81	0.040	18.67	0.030
4	7.87	0.037	18.83	0.030
5	7.85	0.033	18.98	0.030
6	7.78	0.029	19.05	0.030
7	7.68	0.024	19.07	0.030
8	7.64	0.027	19.11	0.029
9	7.57	0.030	19.14	0.029
10	7.50	0.030	19.19	0.029
11	7.42	0.029	19.22	0.032
12	7.34	0.028	19.29	0.035
13	7.28	0.029	19.34	0.036
14	7.21	0.030	19.44	0.036
15	7.15	0.030	19.47	0.037
16	7.10	0.032	19.52	0.037
17	7.05	0.033	19.54	0.038
18	7.00	0.034	19.60	0.038
19	6.94	0.034	19.64	0.039
20	6.89	0.037	19.68	0.040
21	6.84	0.039	19.85	0.037
22	6.81	0.039	19.89	0.037
23	6.76	0.040	19.97	0.037
24	6.63	0.044	20.19	0.032
25	6.65	0.044	20.26	0.032
26	6.59	0.045	20.32	0.033
27	6.57	0.043	20.38	0.036
28	6.57	0.042	20.48	0.041
29	6.49	0.043	20.52	0.042
30	6.50	0.041	20.60	0.043
31	6.44	0.039	20.68	0.045
32	6.40	0.040	20.67	0.047
33	6.24	0.022	20.73	0.048
34	6.21	0.023	20.43	0.023
35	6.18	0.024	20.42	0.025
36	6.15	0.026	20.41	0.027
37	6.10	0.026	20.41	0.027
38	6.13	0.027	20.46	0.024
39	6.10	0.028	20.52	0.025
40	6.08	0.029	20.54	0.026
41	6.30	0.036	20.57	0.026
42	6.28	0.038	20.56	0.028
43	6.28	0.039	20.59	0.029
44	6.27	0.040	20.58	0.034
45	6.24	0.043	20.67	0.035

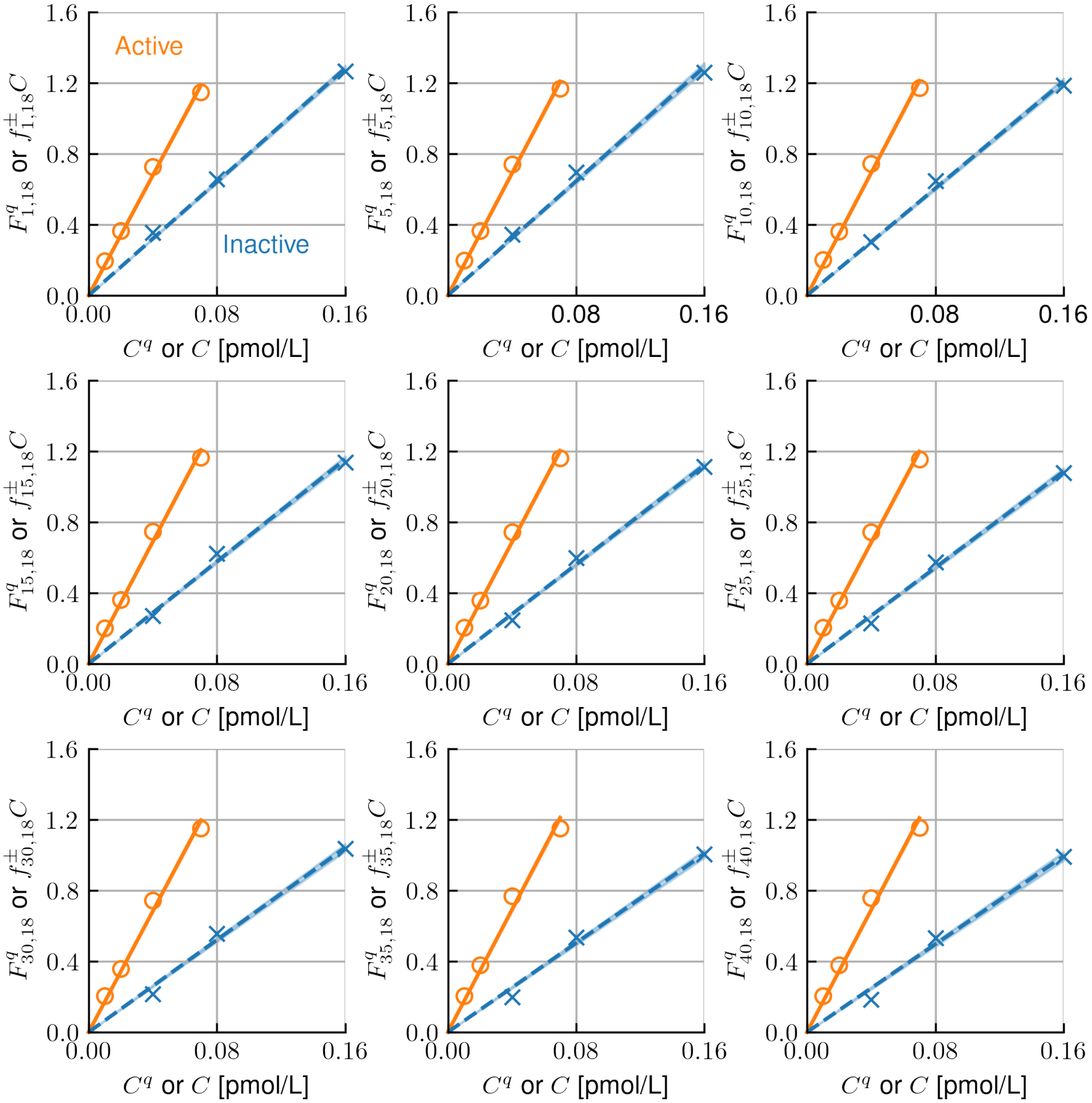

Fig. S18: As Figure S1 with well $w = 18$ (or B6).

Table S18: Molar Fluorescence Parameters for Well B6 ($w = 18$)

Cycle	Inactive		Active	
i	$f^-_{i,18}$	$\sigma^-_{i,18}$	$f^+_{i,18}$	$\sigma^+_{i,18}$
1	8.01	0.030	16.95	0.043
2	7.89	0.044	16.76	0.042
3	8.05	0.043	17.03	0.039
4	8.09	0.044	17.14	0.042
5	8.07	0.046	17.24	0.041
6	7.96	0.044	17.29	0.042
7	7.86	0.040	17.30	0.042
8	7.74	0.037	17.30	0.043
9	7.64	0.033	17.31	0.044
10	7.55	0.033	17.30	0.043
11	7.50	0.035	17.29	0.044
12	7.41	0.037	17.31	0.044
13	7.34	0.037	17.26	0.044
14	7.26	0.037	17.26	0.045
15	7.22	0.036	17.23	0.045
16	7.17	0.035	17.22	0.045
17	7.11	0.035	17.20	0.045
18	7.10	0.034	17.19	0.046
19	7.06	0.034	17.17	0.046
20	7.01	0.036	17.17	0.046
21	6.95	0.035	17.13	0.046
22	6.90	0.036	17.02	0.050
23	6.90	0.036	17.11	0.047
24	6.85	0.039	17.12	0.047
25	6.78	0.038	17.11	0.048
26	6.74	0.039	17.11	0.048
27	6.75	0.038	17.13	0.050
28	6.64	0.039	17.10	0.048
29	6.57	0.042	17.09	0.048
30	6.51	0.041	17.08	0.049
31	6.46	0.040	17.10	0.052
32	6.44	0.041	17.27	0.062
33	6.44	0.041	17.22	0.059
34	6.34	0.041	17.33	0.066
35	6.29	0.043	17.29	0.062
36	6.29	0.045	17.25	0.062
37	6.35	0.046	17.25	0.060
38	6.32	0.047	17.25	0.060
39	6.32	0.048	17.23	0.059
40	6.21	0.050	17.26	0.056
41	6.16	0.049	17.23	0.067
42	6.13	0.046	17.78	0.055
43	6.11	0.047	17.78	0.050
44	6.11	0.047	17.75	0.049
45	6.05	0.045	17.73	0.049

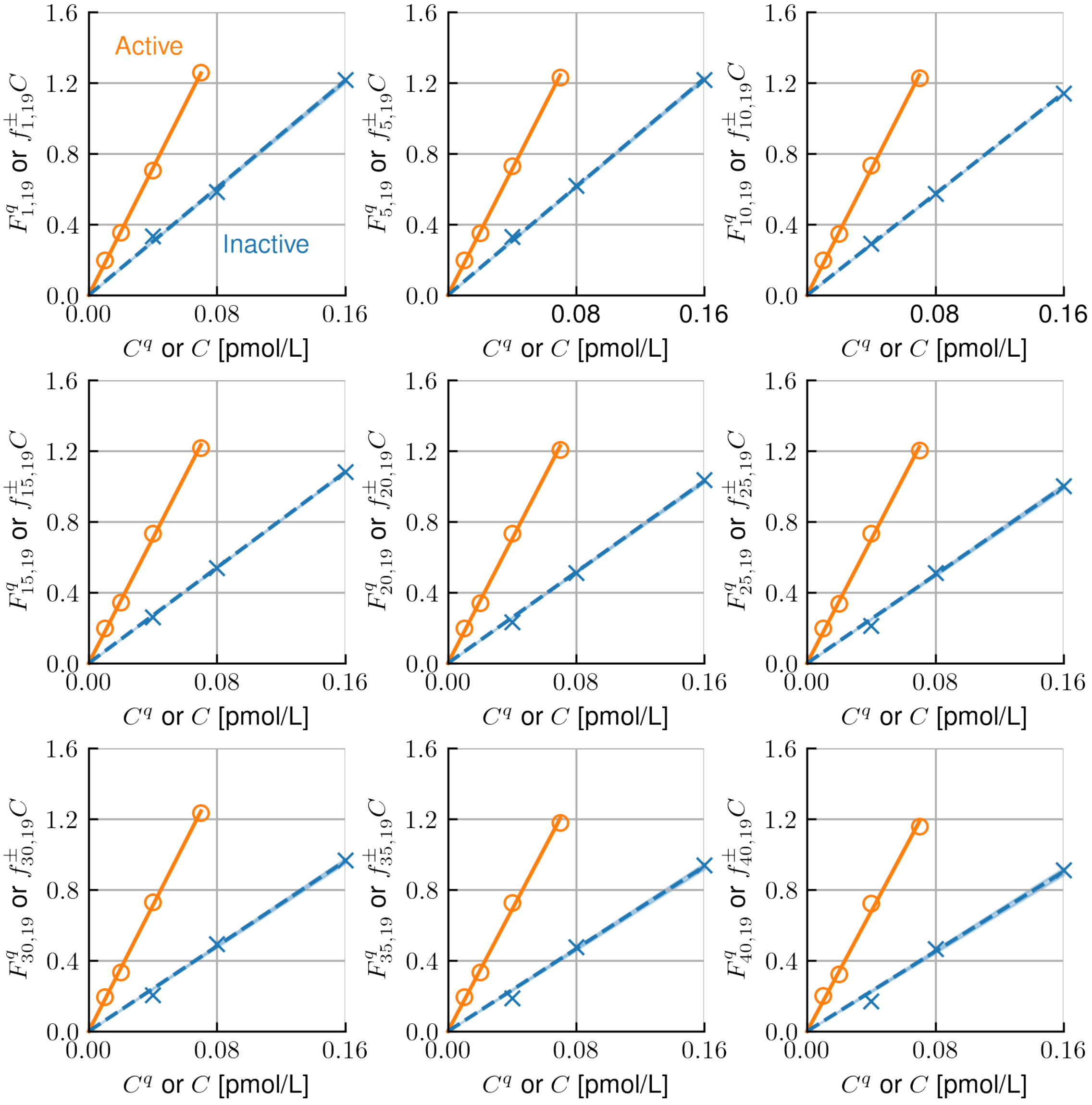

Fig. S19: As Figure S1 with well $w = 19$ (or B7).

Table S19: Molar Fluorescence Parameters for Well B7 ($w = 19$)

Cycle	Inactive		Active	
i	$f^-_{i,19}$	$\sigma^-_{i,19}$	$f^+_{i,19}$	$\sigma^+_{i,19}$
1	7.58	0.027	17.92	0.014
2	7.49	0.024	17.44	0.013
3	7.65	0.022	17.66	0.014
4	7.68	0.021	17.77	0.015
5	7.66	0.019	17.75	0.018
6	7.59	0.016	17.78	0.018
7	7.46	0.015	17.78	0.019
8	7.34	0.012	17.76	0.020
9	7.254	0.0081	17.79	0.020
10	7.153	0.0048	17.76	0.021
11	7.071	0.0022	17.76	0.022
12	6.978	0.0021	17.72	0.022
13	6.915	0.0041	17.71	0.023
14	6.843	0.0066	17.67	0.023
15	6.755	0.0080	17.64	0.024
16	6.69	0.010	17.61	0.024
17	6.63	0.011	17.61	0.024
18	6.54	0.013	17.57	0.026
19	6.49	0.016	17.54	0.026
20	6.44	0.019	17.53	0.027
21	6.41	0.021	17.53	0.026
22	6.39	0.023	17.50	0.027
23	6.34	0.025	17.50	0.027
24	6.29	0.027	17.49	0.027
25	6.24	0.027	17.47	0.027
26	6.21	0.027	17.45	0.027
27	6.17	0.028	17.44	0.028
28	6.11	0.024	17.44	0.027
29	6.06	0.025	17.42	0.028
30	6.02	0.027	17.77	0.021
31	5.98	0.029	17.47	0.025
32	5.94	0.029	17.40	0.026
33	5.94	0.030	17.36	0.027
34	5.88	0.031	17.25	0.029
35	5.83	0.033	17.18	0.031
36	5.78	0.037	17.12	0.032
37	5.74	0.036	17.13	0.031
38	5.71	0.038	17.16	0.032
39	5.69	0.037	17.12	0.033
40	5.64	0.040	16.93	0.038
41	5.66	0.043	17.05	0.034
42	5.61	0.041	17.00	0.035
43	5.59	0.046	17.02	0.033
44	5.51	0.041	16.97	0.035
45	5.49	0.039	17.08	0.046

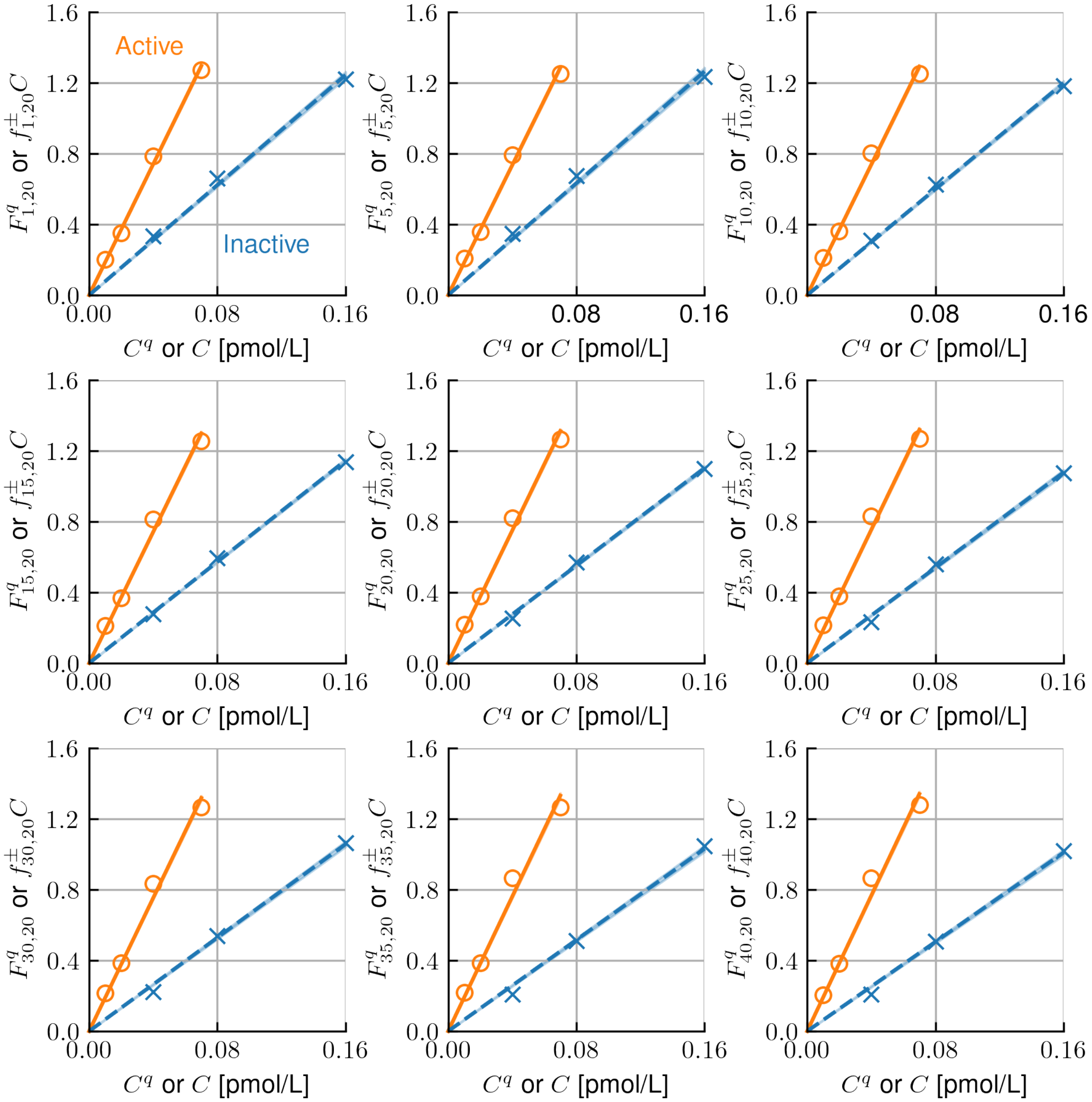

Fig. S20: As Figure S1 with well $w = 20$ (or B8).

Table S20: Molar Fluorescence Parameters for Well B8 ($w = 20$)

Cycle	Inactive		Active	
i	$f^-_{i,20}$	$\sigma^-_{i,20}$	$f^+_{i,20}$	$\sigma^+_{i,20}$
1	7.78	0.036	18.51	0.033
2	7.70	0.044	18.03	0.037
3	7.89	0.043	18.22	0.038
4	7.93	0.044	18.29	0.041
5	7.90	0.043	18.37	0.041
6	7.81	0.040	18.40	0.042
7	7.73	0.034	18.41	0.043
8	7.65	0.029	18.44	0.044
9	7.56	0.026	18.43	0.045
10	7.49	0.023	18.45	0.048
11	7.42	0.020	18.52	0.047
12	7.34	0.018	18.51	0.048
13	7.28	0.018	18.51	0.049
14	7.22	0.018	18.55	0.051
15	7.16	0.017	18.55	0.051
16	7.10	0.017	18.58	0.050
17	7.04	0.019	18.64	0.051
18	6.98	0.019	18.69	0.051
19	6.94	0.018	18.71	0.052
20	6.89	0.020	18.74	0.053
21	6.86	0.023	18.85	0.053
22	6.81	0.025	18.85	0.056
23	6.80	0.025	18.81	0.055
24	6.75	0.027	18.84	0.054
25	6.74	0.029	18.85	0.055
26	6.68	0.029	18.83	0.056
27	6.65	0.029	18.86	0.061
28	6.61	0.030	18.83	0.056
29	6.59	0.030	18.74	0.055
30	6.61	0.031	18.83	0.058
31	6.60	0.034	18.82	0.057
32	6.56	0.034	19.03	0.068
33	6.48	0.035	18.99	0.068
34	6.47	0.034	19.14	0.066
35	6.45	0.036	19.02	0.073
36	6.42	0.035	18.90	0.069
37	6.39	0.034	19.06	0.067
38	6.41	0.031	19.03	0.071
39	6.35	0.030	19.01	0.073
40	6.31	0.032	19.15	0.067
41	6.28	0.033	19.15	0.069
42	6.28	0.034	19.05	0.075
43	6.26	0.034	19.02	0.077
44	6.24	0.035	18.95	0.078
45	6.23	0.035	18.86	0.075

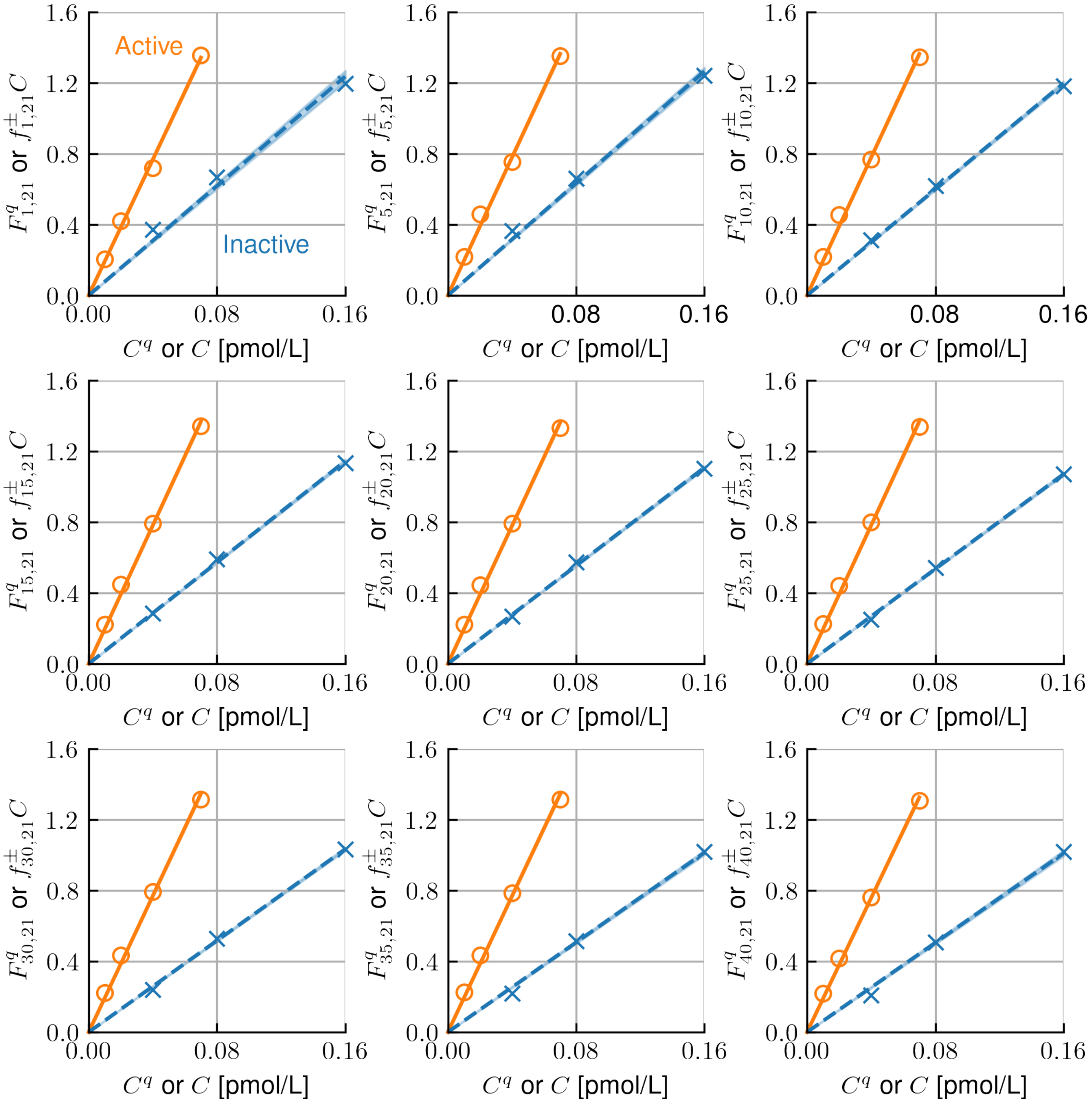

Fig. S21: As Figure S1 with well $w = 21$ (or B9).

Table S21: Molar Fluorescence Parameters for Well B9 ($w = 21$)

Cycle	Inactive		Active	
i	$f^-_{i,21}$	$\sigma^-_{i,21}$	$f^+_{i,21}$	$\sigma^+_{i,21}$
1	7.73	0.064	19.18	0.036
2	7.92	0.049	18.99	0.046
3	8.03	0.045	19.27	0.046
4	7.97	0.044	19.43	0.045
5	7.91	0.043	19.48	0.045
6	7.81	0.036	19.50	0.044
7	7.73	0.031	19.50	0.043
8	7.66	0.025	19.52	0.043
9	7.57	0.023	19.50	0.042
10	7.49	0.021	19.48	0.042
11	7.42	0.018	19.53	0.041
12	7.34	0.017	19.53	0.040
13	7.29	0.014	19.57	0.041
14	7.23	0.015	19.57	0.040
15	7.15	0.016	19.54	0.040
16	7.09	0.017	19.49	0.041
17	7.05	0.016	19.49	0.040
18	6.99	0.016	19.53	0.040
19	6.95	0.016	19.51	0.040
20	6.92	0.016	19.46	0.041
21	6.88	0.016	19.44	0.043
22	6.86	0.015	19.45	0.041
23	6.80	0.017	19.48	0.042
24	6.85	0.016	19.54	0.039
25	6.69	0.013	19.55	0.041
26	6.64	0.011	19.62	0.039
27	6.60	0.013	19.39	0.044
28	6.55	0.014	19.29	0.042
29	6.51	0.016	19.26	0.042
30	6.46	0.015	19.24	0.042
31	6.43	0.016	19.26	0.043
32	6.41	0.017	19.16	0.039
33	6.38	0.015	19.27	0.037
34	6.37	0.024	19.26	0.038
35	6.34	0.024	19.19	0.040
36	6.33	0.026	19.15	0.036
37	6.32	0.028	19.30	0.030
38	6.30	0.029	19.31	0.032
39	6.21	0.028	19.03	0.028
40	6.31	0.031	18.94	0.030
41	6.23	0.032	18.91	0.032
42	6.20	0.031	18.89	0.033
43	6.25	0.035	18.88	0.035
44	6.16	0.033	18.89	0.035
45	6.14	0.040	18.90	0.035

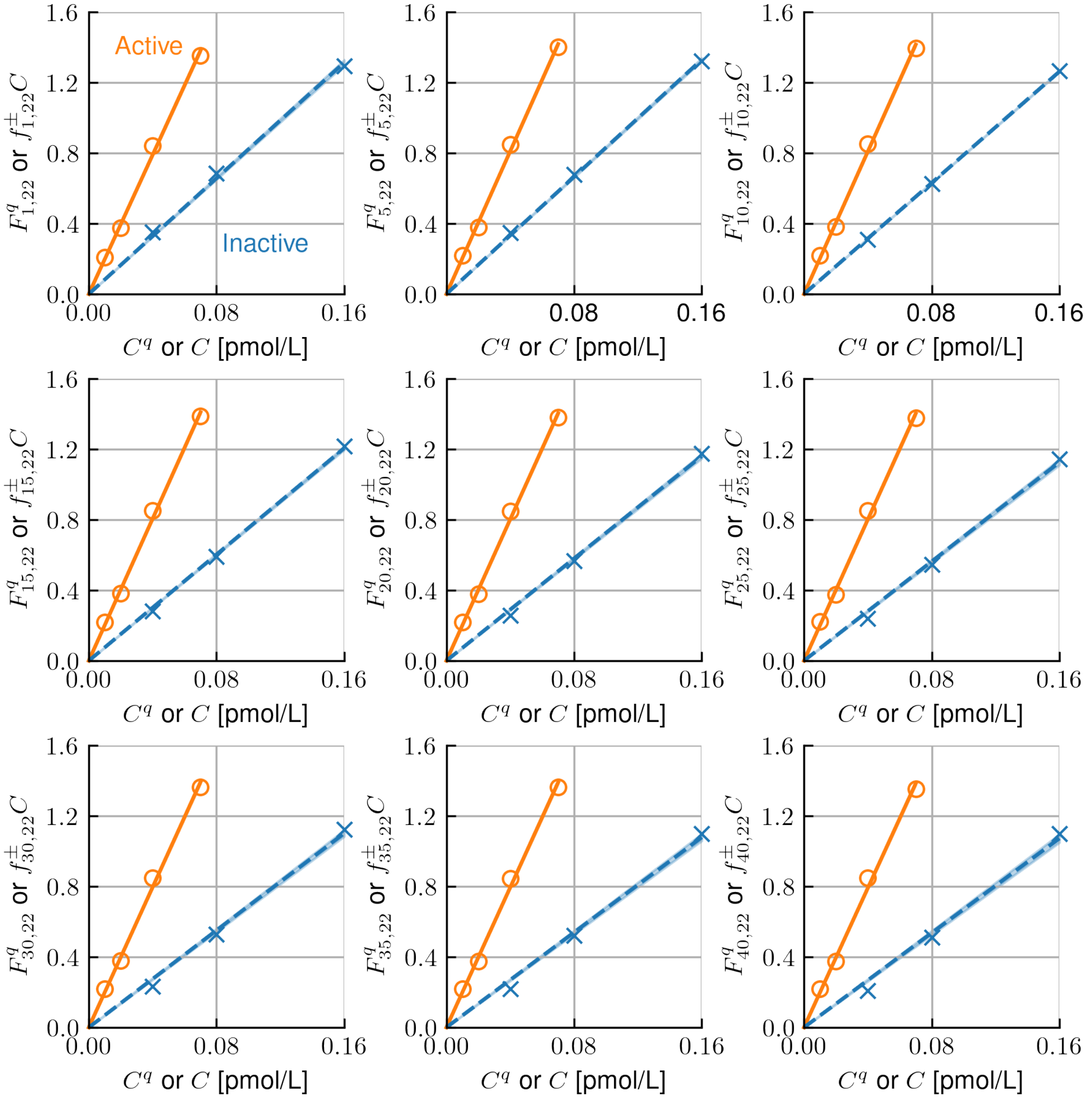

Fig. S22: As Figure S1 with well $w = 22$ (or B10).

Table S22: Molar Fluorescence Parameters for Well B10 ($w = 22$)

Cycle	Inactive		Active	
i	$f^-_{i,22}$	$\sigma^-_{i,22}$	$f^+_{i,22}$	$\sigma^+_{i,22}$
1	8.21	0.029	19.73	0.036
2	8.31	0.025	19.79	0.028
3	8.44	0.021	20.08	0.026
4	8.40	0.022	20.24	0.028
5	8.31	0.016	20.26	0.031
6	8.248	0.0091	20.24	0.031
7	8.142	0.0051	20.26	0.031
8	8.062	0.0011	20.24	0.032
9	7.976	0.0025	20.26	0.032
10	7.888	0.0055	20.22	0.032
11	7.807	0.0083	20.21	0.032
12	7.74	0.011	20.21	0.033
13	7.66	0.013	20.16	0.033
14	7.60	0.017	20.15	0.034
15	7.53	0.018	20.14	0.034
16	7.47	0.019	20.09	0.035
17	7.42	0.022	20.16	0.033
18	7.36	0.024	20.06	0.035
19	7.32	0.026	20.11	0.034
20	7.26	0.027	20.05	0.035
21	7.20	0.028	20.04	0.035
22	7.17	0.029	20.02	0.035
23	7.12	0.031	20.00	0.038
24	7.09	0.033	20.09	0.033
25	7.04	0.033	20.03	0.037
26	7.00	0.035	19.96	0.036
27	6.98	0.036	19.99	0.037
28	6.94	0.035	19.93	0.039
29	6.91	0.035	19.89	0.038
30	6.88	0.037	19.89	0.038
31	6.91	0.039	20.00	0.034
32	6.87	0.037	19.93	0.036
33	6.83	0.036	19.88	0.037
34	6.80	0.037	19.95	0.037
35	6.74	0.040	19.86	0.038
36	6.71	0.042	19.78	0.035
37	6.65	0.039	19.83	0.042
38	6.62	0.036	19.78	0.041
39	6.68	0.046	19.74	0.038
40	6.70	0.048	19.77	0.041
41	6.71	0.054	19.97	0.035
42	6.68	0.053	19.95	0.035
43	6.64	0.051	19.85	0.033
44	6.66	0.053	19.86	0.037
45	6.67	0.053	19.79	0.038

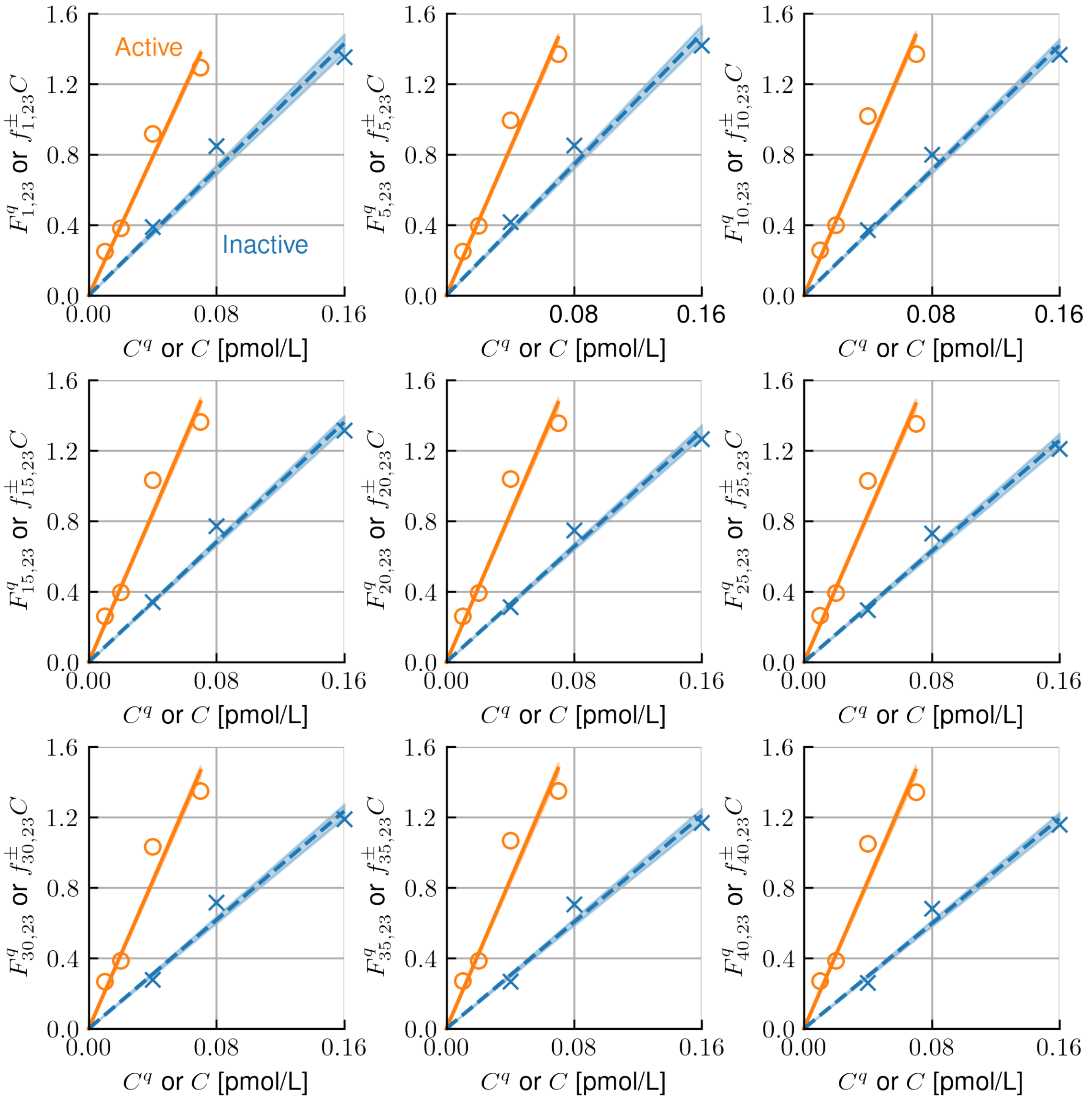

Fig. S23: As Figure S1 with well $w = 23$ (or B11).

Table S23: Molar Fluorescence Parameters for Well B11 ($w = 23$)

Cycle	Inactive		Active	
i	$f^-_{i,23}$	$\sigma^-_{i,23}$	$f^+_{i,23}$	$\sigma^+_{i,23}$
1	8.9	0.11	19.63	0.094
2	9.2	0.10	20.15	0.099
3	9.37	0.100	20.5	0.11
4	9.36	0.100	20.7	0.11
5	9.28	0.097	20.9	0.11
6	9.22	0.090	20.9	0.11
7	9.13	0.084	21.0	0.12
8	9.02	0.082	21.0	0.12
9	8.94	0.077	21.1	0.12
10	8.86	0.075	21.1	0.12
11	8.77	0.076	21.1	0.12
12	8.70	0.075	21.1	0.13
13	8.64	0.075	21.1	0.13
14	8.57	0.074	21.1	0.13
15	8.51	0.073	21.1	0.13
16	8.44	0.072	21.0	0.13
17	8.36	0.073	21.0	0.13
18	8.30	0.074	21.0	0.13
19	8.25	0.072	21.0	0.13
20	8.18	0.074	21.0	0.14
21	8.15	0.073	21.0	0.14
22	8.01	0.080	21.0	0.14
23	7.98	0.078	21.0	0.14
24	7.92	0.080	20.9	0.13
25	7.86	0.081	20.9	0.13
26	7.82	0.081	20.9	0.14
27	7.78	0.081	20.9	0.14
28	7.76	0.080	20.8	0.13
29	7.72	0.081	20.9	0.14
30	7.71	0.080	20.9	0.14
31	7.63	0.084	21.0	0.14
32	7.60	0.085	20.9	0.14
33	7.56	0.084	21.0	0.15
34	7.54	0.083	21.0	0.15
35	7.56	0.080	21.1	0.15
36	7.55	0.081	21.0	0.16
37	7.56	0.078	21.0	0.16
38	7.52	0.070	21.1	0.16
39	7.50	0.070	21.1	0.16
40	7.44	0.071	20.9	0.15
41	7.36	0.074	20.9	0.15
42	7.34	0.074	20.9	0.15
43	7.35	0.073	20.9	0.15
44	7.38	0.070	20.9	0.15
45	7.31	0.073	20.9	0.15

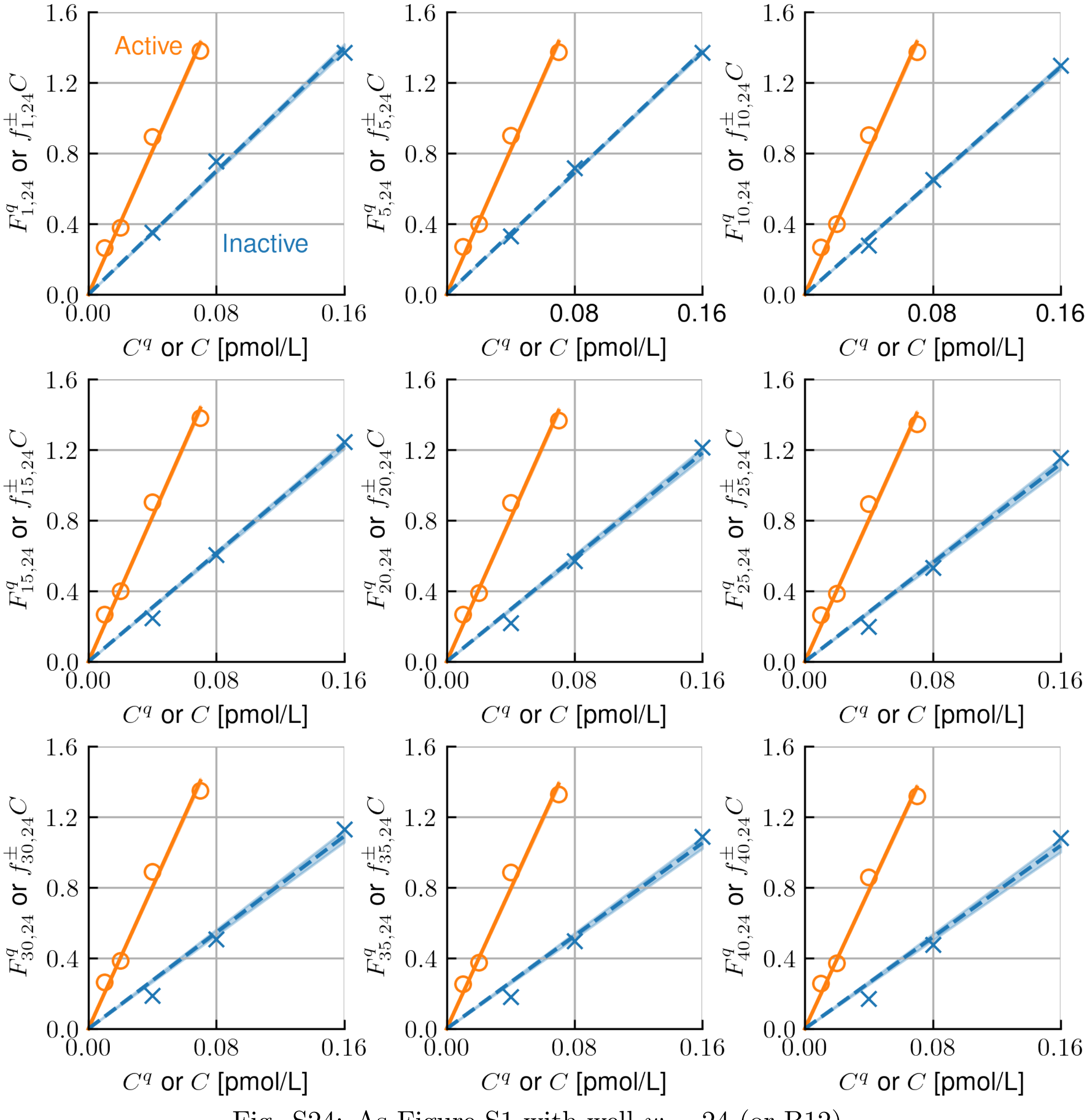

Fig. S24: As Figure S1 with well $w = 24$ (or B12).

Table S24: Molar Fluorescence Parameters for Well B12 ($w = 24$)

Cycle	Inactive		Active	
i	$f^-_{i,24}$	$\sigma^-_{i,24}$	$f^+_{i,24}$	$\sigma^+_{i,24}$
1	8.73	0.044	20.37	0.066
2	8.80	0.041	20.13	0.066
3	8.85	0.033	20.32	0.070
4	8.76	0.027	20.40	0.070
5	8.62	0.022	20.45	0.071
6	8.50	0.021	20.44	0.071
7	8.38	0.023	20.47	0.071
8	8.27	0.025	20.44	0.071
9	8.17	0.029	20.42	0.072
10	8.06	0.032	20.42	0.071
11	8.01	0.036	20.42	0.071
12	7.90	0.039	20.51	0.068
13	7.82	0.042	20.35	0.073
14	7.75	0.044	20.37	0.071
15	7.67	0.044	20.51	0.068
16	7.63	0.050	20.38	0.072
17	7.60	0.054	20.28	0.071
18	7.49	0.055	20.30	0.071
19	7.42	0.056	20.29	0.070
20	7.39	0.060	20.30	0.071
21	7.28	0.060	20.13	0.073
22	7.18	0.059	20.12	0.073
23	7.12	0.061	20.10	0.073
24	7.06	0.064	20.07	0.074
25	7.00	0.065	20.07	0.073
26	6.94	0.066	20.13	0.077
27	6.87	0.066	20.08	0.070
28	6.86	0.068	20.15	0.069
29	6.81	0.065	20.09	0.070
30	6.82	0.071	20.05	0.070
31	6.72	0.068	20.05	0.070
32	6.69	0.066	20.08	0.069
33	6.66	0.067	19.91	0.073
34	6.63	0.068	19.81	0.076
35	6.58	0.066	19.80	0.073
36	6.52	0.068	19.76	0.073
37	6.50	0.067	19.77	0.075
38	6.51	0.070	19.69	0.065
39	6.46	0.072	19.58	0.063
40	6.48	0.076	19.54	0.064
41	6.49	0.080	19.54	0.065
42	6.45	0.083	19.36	0.070
43	6.47	0.084	19.37	0.069
44	6.44	0.085	19.38	0.070
45	6.37	0.084	19.35	0.071

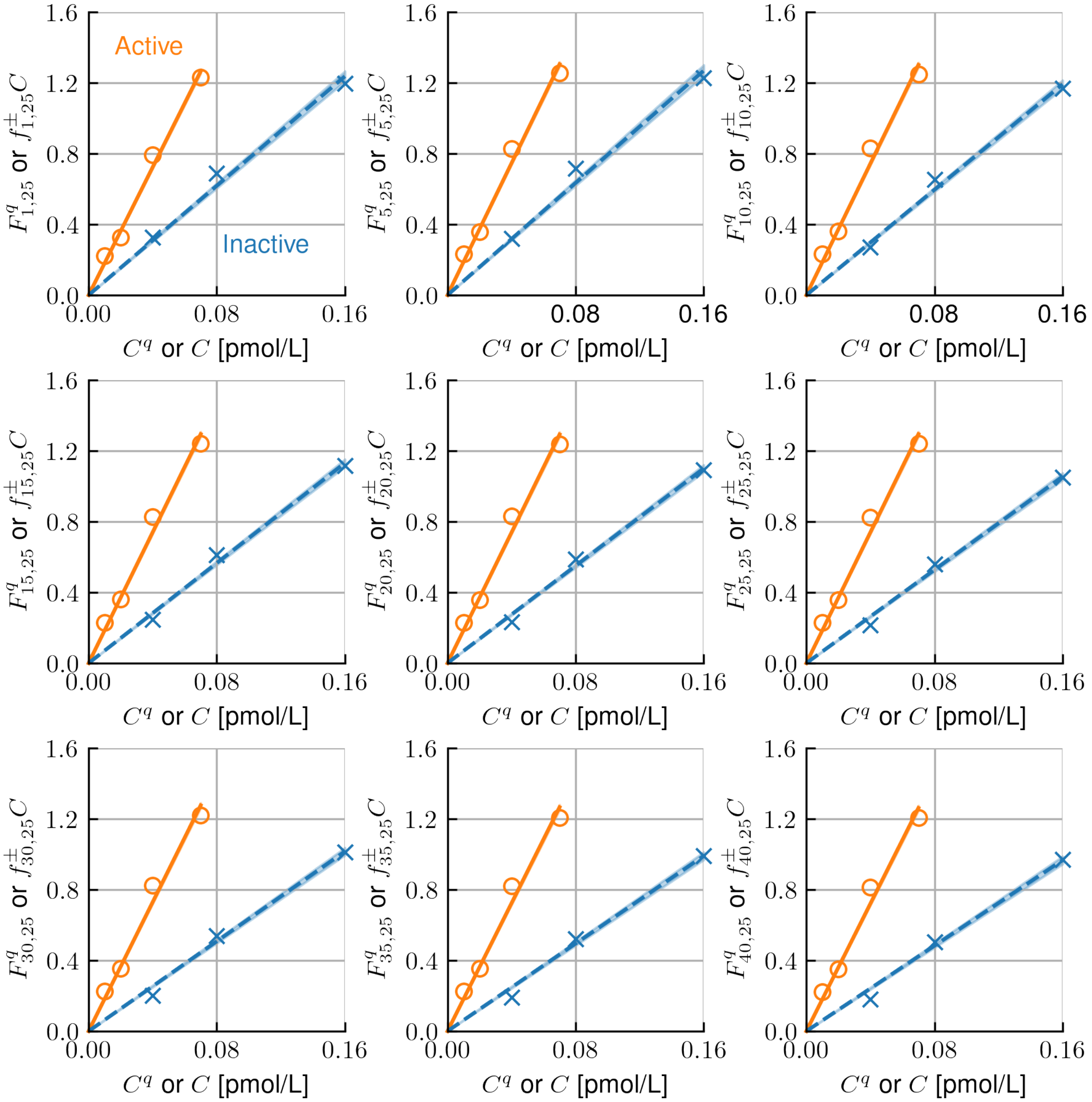

Fig. S25: As Figure S1 with well $w = 25$ (or C1).

Table S25: Molar Fluorescence Parameters for Well C1 ($w = 25$)

Cycle	Inactive		Active	
i	$f^-_{i,25}$	$\sigma^-_{i,25}$	$f^+_{i,25}$	$\sigma^+_{i,25}$
1	7.73	0.059	18.08	0.055
2	8.04	0.071	18.30	0.053
3	8.14	0.070	18.55	0.055
4	8.06	0.068	18.62	0.058
5	7.93	0.065	18.65	0.061
6	7.82	0.061	18.64	0.062
7	7.72	0.056	18.64	0.062
8	7.61	0.053	18.66	0.062
9	7.52	0.050	18.63	0.063
10	7.45	0.047	18.59	0.064
11	7.36	0.047	18.57	0.065
12	7.30	0.046	18.54	0.064
13	7.25	0.046	18.56	0.063
14	7.15	0.046	18.50	0.065
15	7.07	0.043	18.50	0.065
16	7.02	0.043	18.48	0.066
17	6.98	0.040	18.50	0.067
18	6.93	0.040	18.50	0.068
19	6.88	0.040	18.47	0.066
20	6.88	0.041	18.47	0.067
21	6.84	0.041	18.68	0.062
22	6.79	0.040	18.50	0.064
23	6.77	0.040	18.46	0.065
24	6.70	0.040	18.40	0.068
25	6.59	0.042	18.49	0.064
26	6.56	0.042	18.34	0.067
27	6.51	0.043	18.28	0.069
28	6.44	0.042	18.27	0.069
29	6.38	0.043	18.26	0.068
30	6.35	0.042	18.23	0.068
31	6.32	0.045	18.28	0.073
32	6.30	0.044	18.15	0.070
33	6.26	0.044	18.13	0.070
34	6.25	0.044	18.10	0.070
35	6.19	0.044	18.08	0.070
36	6.14	0.045	18.09	0.069
37	6.08	0.046	18.07	0.068
38	6.06	0.046	18.04	0.068
39	6.05	0.045	18.02	0.068
40	6.04	0.045	18.02	0.067
41	5.98	0.045	17.83	0.073
42	5.95	0.048	17.83	0.071
43	5.90	0.047	17.83	0.070
44	5.89	0.048	17.72	0.072
45	5.87	0.049	17.70	0.072

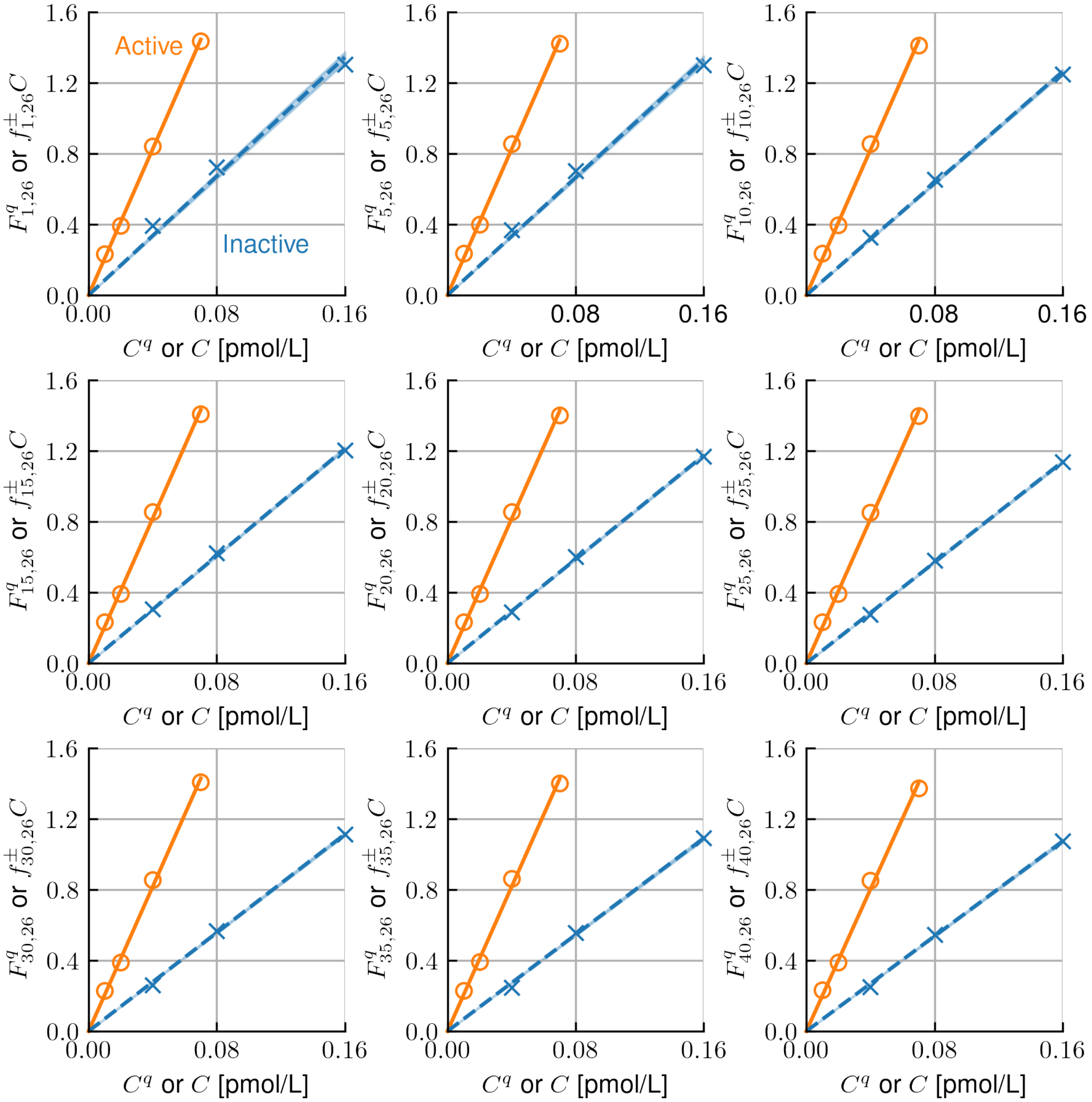

Fig. S26: As Figure S1 with well $w = 26$ (or C2).

Table S26: Molar Fluorescence Parameters for Well C2 ($w = 26$)

Cycle	Inactive		Active	
i	$f^-_{i,26}$	$\sigma^-_{i,26}$	$f^+_{i,26}$	$\sigma^+_{i,26}$
1	8.40	0.060	20.63	0.022
2	8.51	0.059	20.43	0.024
3	8.56	0.055	20.52	0.028
4	8.45	0.049	20.56	0.029
5	8.31	0.043	20.60	0.028
6	8.20	0.036	20.59	0.029
7	8.12	0.030	20.56	0.030
8	8.03	0.026	20.54	0.030
9	7.95	0.024	20.54	0.031
10	7.90	0.021	20.51	0.031
11	7.82	0.019	20.48	0.031
12	7.77	0.017	20.49	0.030
13	7.69	0.016	20.46	0.032
14	7.66	0.014	20.52	0.030
15	7.58	0.014	20.45	0.032
16	7.51	0.015	20.44	0.032
17	7.45	0.014	20.40	0.033
18	7.42	0.013	20.45	0.032
19	7.39	0.011	20.39	0.032
20	7.34	0.012	20.37	0.033
21	7.29	0.013	20.44	0.031
22	7.20	0.013	20.35	0.033
23	7.22	0.011	20.33	0.033
24	7.16	0.011	20.32	0.033
25	7.12	0.011	20.33	0.033
26	7.05	0.013	20.44	0.030
27	7.02	0.014	20.36	0.032
28	7.00	0.015	20.34	0.033
29	6.97	0.014	20.29	0.033
30	6.96	0.016	20.41	0.031
31	6.97	0.023	20.36	0.035
32	6.92	0.019	20.30	0.035
33	6.90	0.017	20.21	0.036
34	6.92	0.019	20.25	0.043
35	6.82	0.019	20.38	0.035
36	6.78	0.018	20.23	0.037
37	6.83	0.019	20.04	0.040
38	6.72	0.017	20.09	0.038
39	6.77	0.015	20.05	0.039
40	6.71	0.015	20.04	0.039
41	6.63	0.021	20.03	0.039
42	6.60	0.018	19.96	0.044
43	6.60	0.017	19.92	0.043
44	6.50	0.021	19.95	0.042
45	6.55	0.019	19.92	0.042

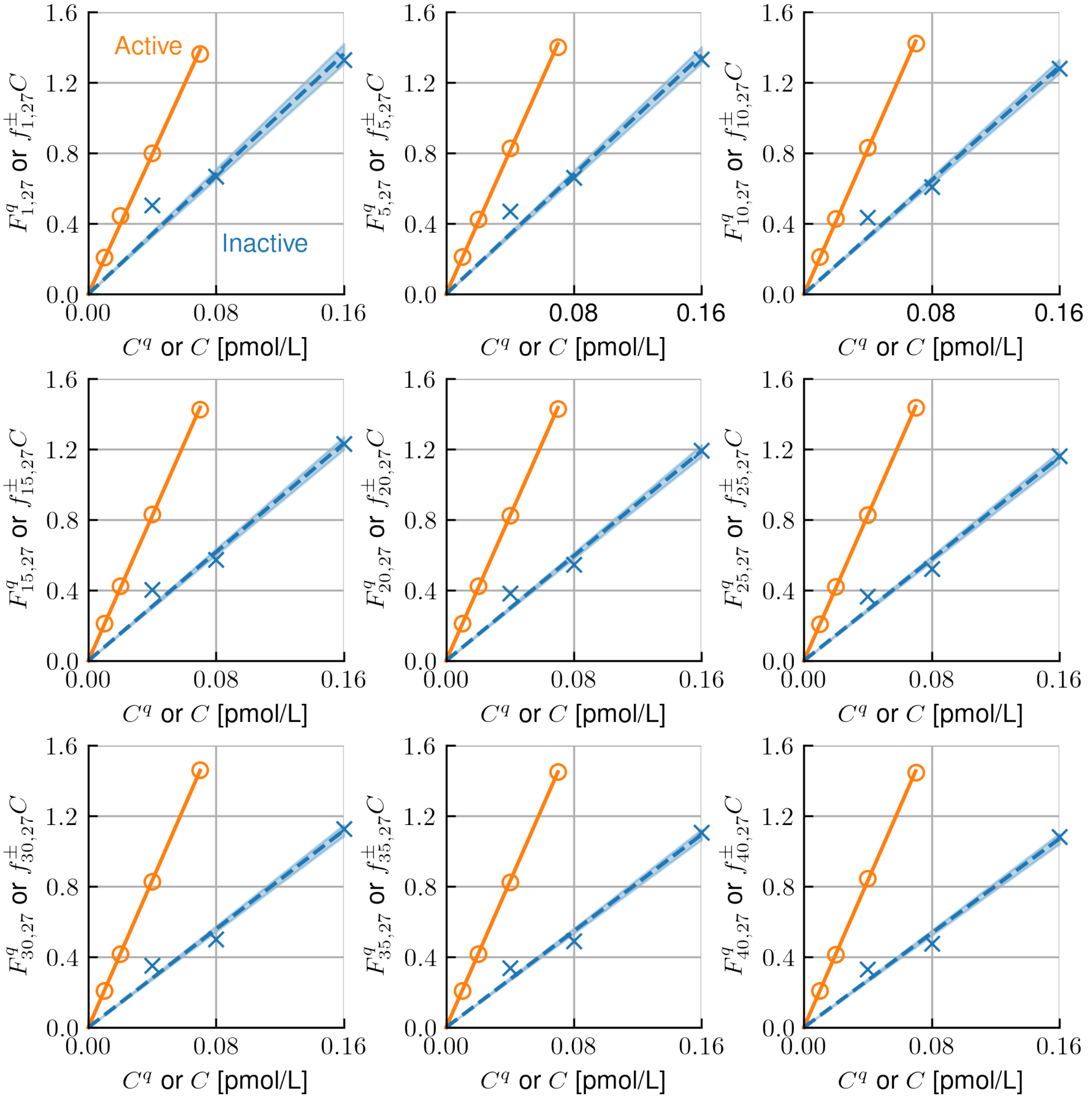

Fig. S27: As Figure S1 with well $w = 27$ (or C3).

Table S27: Molar Fluorescence Parameters for Well C3 ($w = 27$)

Cycle	Inactive		Active	
i	$f^-_{i,27}$	$\sigma^-_{i,27}$	$f^+_{i,27}$	$\sigma^+_{i,27}$
1	8.5	0.12	19.78	0.032
2	8.4	0.10	19.87	0.018
3	8.6	0.10	20.09	0.018
4	8.56	0.099	20.22	0.019
5	8.47	0.095	20.28	0.019
6	8.41	0.092	20.36	0.019
7	8.33	0.089	20.41	0.018
8	8.24	0.086	20.45	0.016
9	8.15	0.083	20.50	0.016
10	8.07	0.082	20.49	0.015
11	7.98	0.080	20.51	0.014
12	7.91	0.078	20.53	0.014
13	7.85	0.077	20.53	0.013
14	7.78	0.075	20.52	0.013
15	7.71	0.075	20.53	0.012
16	7.67	0.073	20.54	0.012
17	7.60	0.072	20.55	0.011
18	7.54	0.072	20.54	0.011
19	7.49	0.071	20.54	0.010
20	7.44	0.070	20.515	0.0096
21	7.39	0.068	20.540	0.0096
22	7.35	0.069	20.549	0.0089
23	7.30	0.068	20.56	0.012
24	7.26	0.068	20.526	0.0097
25	7.21	0.067	20.597	0.0067
26	7.18	0.068	20.569	0.0065
27	7.12	0.066	20.539	0.0071
28	7.06	0.064	20.602	0.0046
29	7.02	0.065	20.580	0.0067
30	6.98	0.066	20.817	0.0032
31	6.96	0.061	20.595	0.0047
32	6.92	0.061	20.879	0.0063
33	6.89	0.059	20.760	0.0043
34	6.86	0.059	20.753	0.0031
35	6.83	0.060	20.701	0.0032
36	6.79	0.059	20.767	0.0048
37	6.77	0.060	20.811	0.0073
38	6.75	0.061	20.90	0.010
39	6.72	0.062	20.832	0.0082
40	6.69	0.061	20.776	0.0091
41	6.72	0.059	20.769	0.0096
42	6.68	0.060	20.737	0.0092
43	6.66	0.058	20.727	0.0071
44	6.62	0.055	20.638	0.0087
45	6.60	0.058	20.609	0.0092

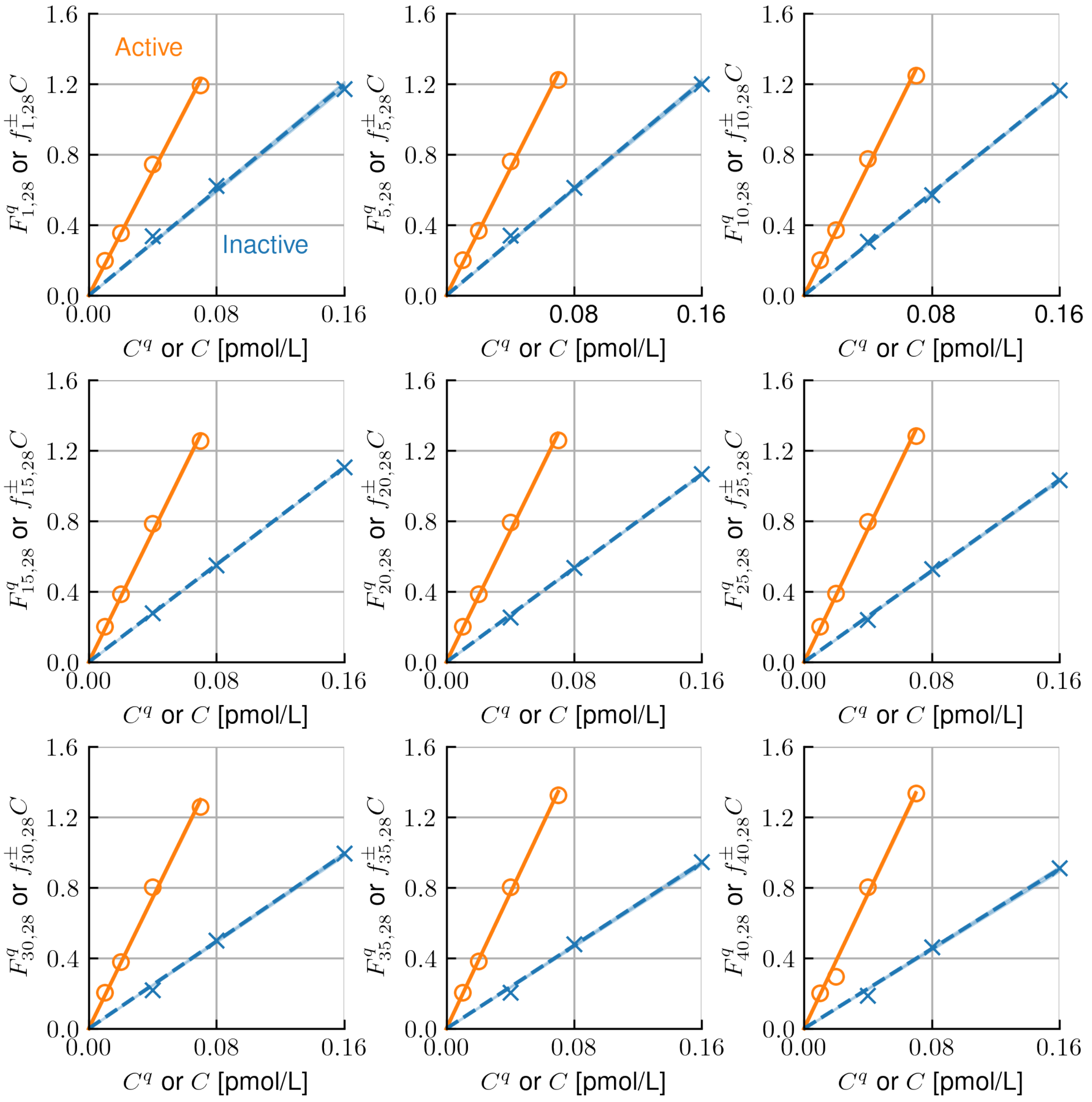

Fig. S28: As Figure S1 with well $w = 28$ (or C4).

Table S28: Molar Fluorescence Parameters for Well C4 ($w = 28$)

Cycle	Inactive		Active	
i	$f^-_{i,28}$	$\sigma^-_{i,28}$	$f^+_{i,28}$	$\sigma^+_{i,28}$
1	7.47	0.035	17.47	0.034
2	7.40	0.038	17.34	0.036
3	7.55	0.036	17.57	0.037
4	7.59	0.033	17.78	0.034
5	7.58	0.029	17.94	0.034
6	7.54	0.024	18.04	0.034
7	7.47	0.021	18.13	0.033
8	7.40	0.018	18.21	0.032
9	7.33	0.014	18.24	0.033
10	7.27	0.013	18.28	0.033
11	7.18	0.010	18.36	0.031
12	7.107	0.0074	18.31	0.034
13	7.052	0.0047	18.39	0.035
14	6.988	0.0040	18.41	0.036
15	6.906	0.0017	18.43	0.038
16	6.856	0.0013	18.44	0.039
17	6.816	0.0024	18.48	0.039
18	6.763	0.0044	18.54	0.039
19	6.726	0.0066	18.51	0.041
20	6.663	0.0082	18.52	0.040
21	6.630	0.0098	18.55	0.040
22	6.57	0.011	18.62	0.040
23	6.53	0.012	18.61	0.040
24	6.50	0.015	18.67	0.039
25	6.46	0.016	18.80	0.034
26	6.43	0.017	18.79	0.035
27	6.28	0.014	18.69	0.039
28	6.25	0.018	18.77	0.037
29	6.23	0.019	18.75	0.037
30	6.19	0.020	18.54	0.044
31	6.15	0.022	18.54	0.045
32	6.13	0.024	18.55	0.043
33	6.09	0.026	19.17	0.025
34	6.07	0.027	19.15	0.025
35	5.89	0.024	19.21	0.024
36	5.86	0.024	19.26	0.023
37	5.80	0.026	18.97	0.057
38	5.76	0.026	19.04	0.055
39	5.70	0.026	19.08	0.055
40	5.66	0.027	19.09	0.056
41	5.63	0.030	19.14	0.056
42	5.60	0.031	19.20	0.056
43	5.57	0.031	19.35	0.062
44	5.54	0.032	19.37	0.066
45	5.51	0.032	19.15	0.067

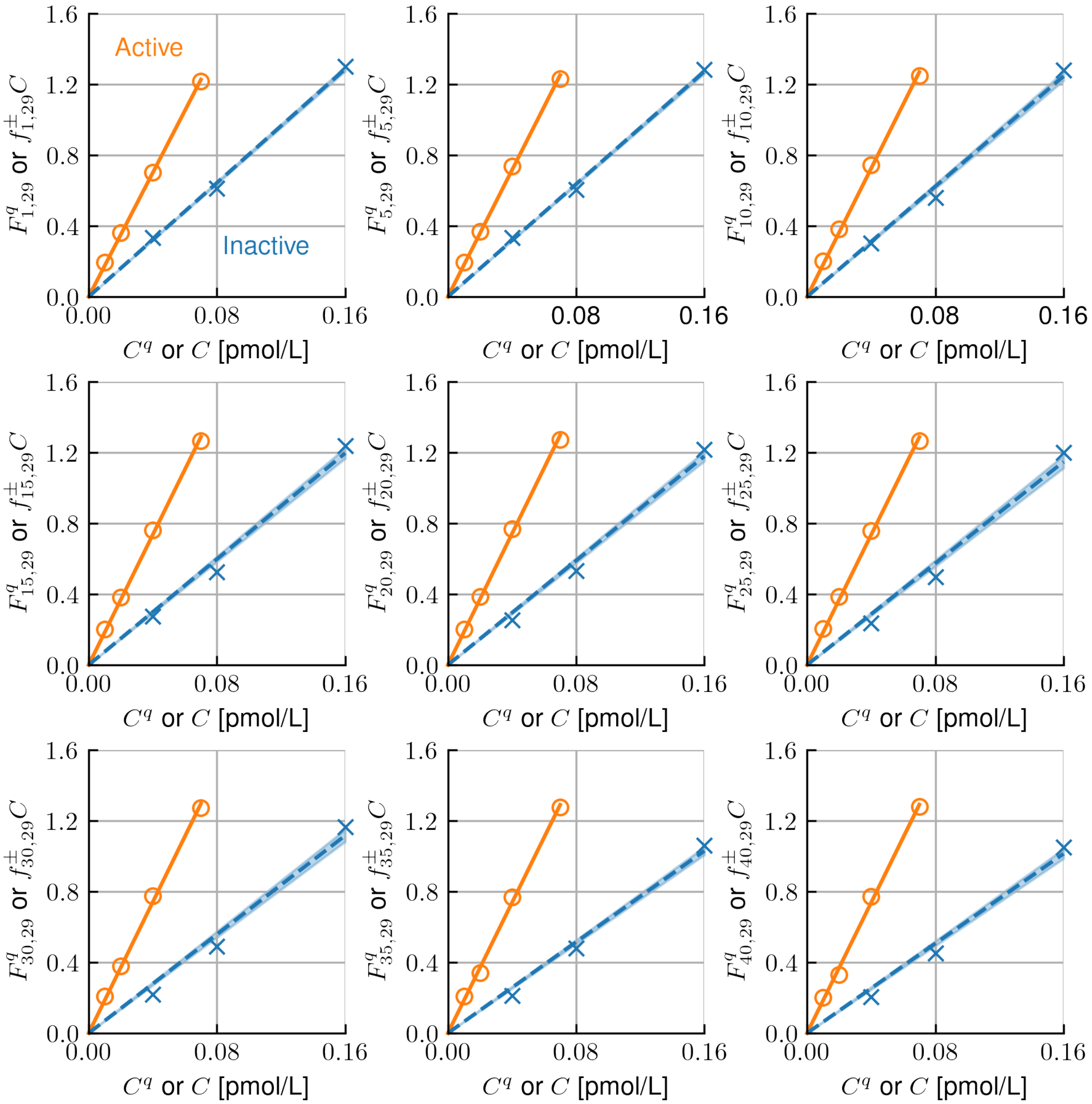

Fig. S29: As Figure S1 with well $w = 29$ (or C5).

Table S29: Molar Fluorescence Parameters for Well C5 ($w = 29$)

Cycle	Inactive		Active	
i	$f^{-}_{i,29}$	$\sigma^{-}_{i,29}$	$f^{+}_{i,29}$	$\sigma^{+}_{i,29}$
1	8.04	0.026	17.52	0.013
2	7.82	0.021	17.44	0.020
3	7.95	0.019	17.61	0.021
4	7.99	0.023	17.77	0.020
5	7.95	0.026	17.88	0.021
6	7.89	0.029	18.00	0.020
7	7.91	0.038	18.07	0.021
8	7.89	0.045	18.07	0.023
9	7.85	0.049	18.10	0.023
10	7.79	0.052	18.13	0.022
11	7.71	0.053	18.27	0.019
12	7.65	0.055	18.27	0.019
13	7.60	0.057	18.29	0.020
14	7.53	0.060	18.35	0.022
15	7.48	0.062	18.40	0.024
16	7.47	0.053	18.43	0.025
17	7.44	0.051	18.41	0.027
18	7.43	0.055	18.52	0.024
19	7.40	0.056	18.52	0.025
20	7.37	0.057	18.53	0.025
21	7.31	0.056	18.59	0.026
22	7.27	0.058	18.58	0.026
23	7.23	0.065	18.45	0.030
24	7.18	0.073	18.36	0.024
25	7.17	0.074	18.38	0.024
26	7.11	0.071	18.42	0.023
27	7.06	0.070	18.44	0.024
28	7.04	0.070	18.46	0.025
29	7.01	0.070	18.47	0.026
30	6.98	0.071	18.52	0.029
31	6.54	0.042	18.49	0.025
32	6.50	0.046	18.39	0.030
33	6.49	0.046	18.41	0.028
34	6.47	0.046	18.42	0.028
35	6.44	0.046	18.42	0.029
36	6.42	0.048	18.36	0.031
37	6.42	0.049	18.38	0.029
38	6.37	0.060	18.41	0.030
39	6.37	0.058	18.43	0.031
40	6.33	0.057	18.44	0.032
41	6.33	0.057	17.88	0.042
42	6.32	0.058	17.79	0.052
43	6.30	0.059	17.79	0.052
44	6.28	0.058	17.58	0.052
45	6.23	0.060	17.56	0.053

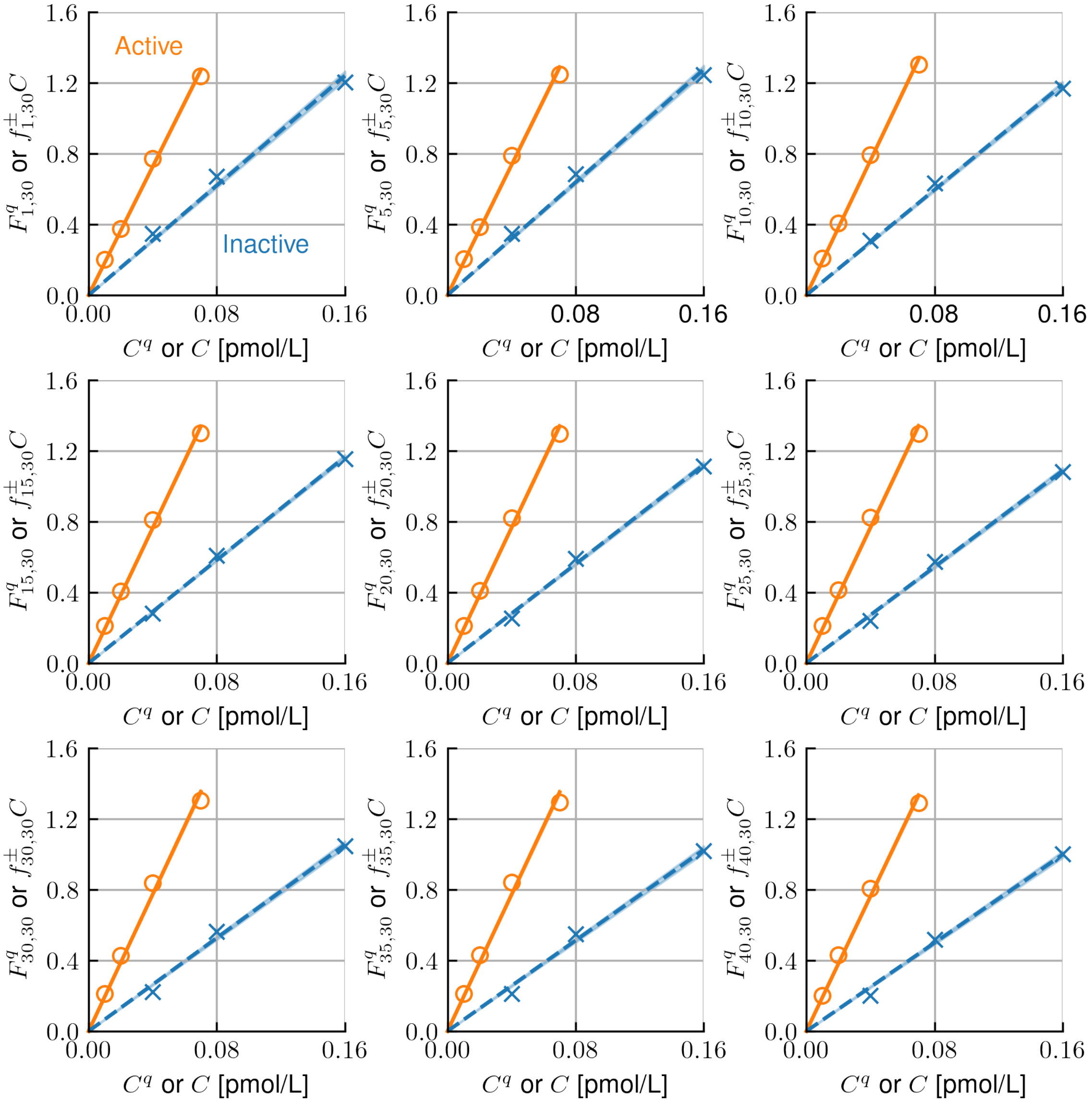

Fig. S30: As Figure S1 with well $w = 30$ (or C6).

Table S30: Molar Fluorescence Parameters for Well C6 ($w = 30$)

Cycle	Inactive		Active	
i	$f^-_{i,30}$	$\sigma^-_{i,30}$	$f^+_{i,30}$	$\sigma^+_{i,30}$
1	7.75	0.051	18.15	0.036
2	7.78	0.044	17.89	0.039
3	7.89	0.047	18.09	0.041
4	7.97	0.048	18.28	0.040
5	7.97	0.045	18.39	0.042
6	7.90	0.042	18.38	0.046
7	7.79	0.037	18.69	0.038
8	7.68	0.036	18.89	0.033
9	7.57	0.033	18.96	0.031
10	7.44	0.031	19.02	0.031
11	7.45	0.027	19.06	0.030
12	7.43	0.025	19.09	0.034
13	7.39	0.022	19.12	0.035
14	7.35	0.022	19.11	0.037
15	7.29	0.022	19.10	0.038
16	7.22	0.023	19.11	0.039
17	7.18	0.025	19.14	0.039
18	7.12	0.026	19.14	0.040
19	7.07	0.027	19.12	0.042
20	7.01	0.029	19.13	0.044
21	6.95	0.031	19.15	0.045
22	6.90	0.028	19.16	0.045
23	6.92	0.029	19.12	0.046
24	6.88	0.030	19.15	0.048
25	6.80	0.031	19.15	0.047
26	6.74	0.031	19.15	0.047
27	6.71	0.034	19.14	0.047
28	6.67	0.033	19.20	0.051
29	6.62	0.037	19.22	0.055
30	6.59	0.038	19.33	0.053
31	6.54	0.039	19.36	0.052
32	6.52	0.039	19.37	0.056
33	6.50	0.041	19.33	0.057
34	6.46	0.041	19.32	0.057
35	6.41	0.041	19.30	0.059
36	6.37	0.035	19.35	0.061
37	6.36	0.036	19.25	0.057
38	6.30	0.037	19.25	0.059
39	6.27	0.037	19.24	0.059
40	6.24	0.036	19.04	0.046
41	6.23	0.037	18.94	0.039
42	6.20	0.038	18.95	0.040
43	6.18	0.039	18.54	0.053
44	6.09	0.041	18.50	0.056
45	5.85	0.033	18.48	0.055

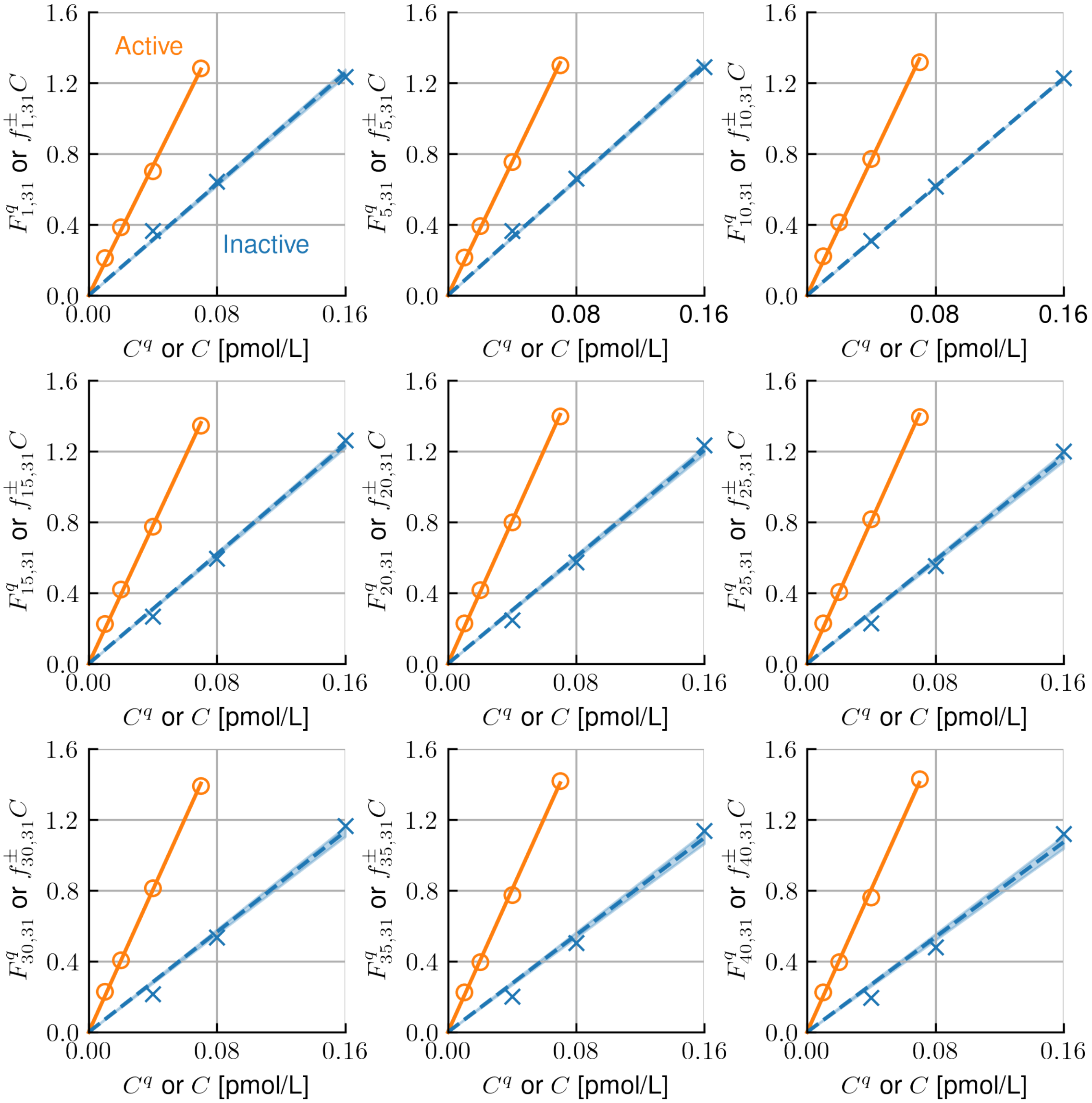

Fig. S31: As Figure S1 with well $w = 31$ (or C7).

Table S31: Molar Fluorescence Parameters for Well C7 ($w = 31$)

Cycle	Inactive		Active	
i	$f_{i,31}^{-}$	$\sigma_{i,31}^{-}$	$f_{i,31}^{+}$	$\sigma_{i,31}^{+}$
1	7.85	0.039	18.24	0.026
2	7.86	0.032	17.99	0.020
3	8.02	0.035	18.27	0.020
4	8.16	0.033	18.54	0.020
5	8.15	0.029	18.77	0.020
6	8.08	0.024	18.87	0.020
7	7.96	0.019	18.92	0.021
8	7.84	0.013	18.98	0.023
9	7.754	0.0073	19.00	0.025
10	7.669	0.0017	19.07	0.028
11	7.81	0.016	19.10	0.030
12	7.78	0.022	19.09	0.031
13	7.78	0.029	19.10	0.032
14	7.76	0.035	19.11	0.032
15	7.74	0.039	19.43	0.027
16	7.70	0.042	19.74	0.023
17	7.68	0.042	19.82	0.023
18	7.65	0.045	19.87	0.023
19	7.61	0.046	19.92	0.022
20	7.54	0.047	20.09	0.019
21	7.53	0.051	20.10	0.019
22	7.49	0.054	20.15	0.019
23	7.41	0.053	20.09	0.020
24	7.37	0.055	20.21	0.018
25	7.30	0.054	20.12	0.019
26	7.18	0.050	20.16	0.020
27	7.11	0.052	20.18	0.019
28	7.09	0.053	20.20	0.019
29	7.09	0.057	20.18	0.018
30	7.07	0.056	20.06	0.020
31	7.01	0.060	20.17	0.016
32	6.98	0.062	20.12	0.017
33	6.97	0.065	20.09	0.020
34	6.91	0.064	20.06	0.021
35	6.85	0.066	20.09	0.023
36	6.82	0.067	20.09	0.024
37	6.79	0.070	20.10	0.027
38	6.75	0.072	20.12	0.029
39	6.72	0.073	20.14	0.031
40	6.70	0.074	20.12	0.032
41	6.63	0.070	19.89	0.028
42	6.56	0.069	19.75	0.021
43	6.49	0.068	19.83	0.027
44	6.44	0.070	19.85	0.027
45	6.41	0.070	19.86	0.029

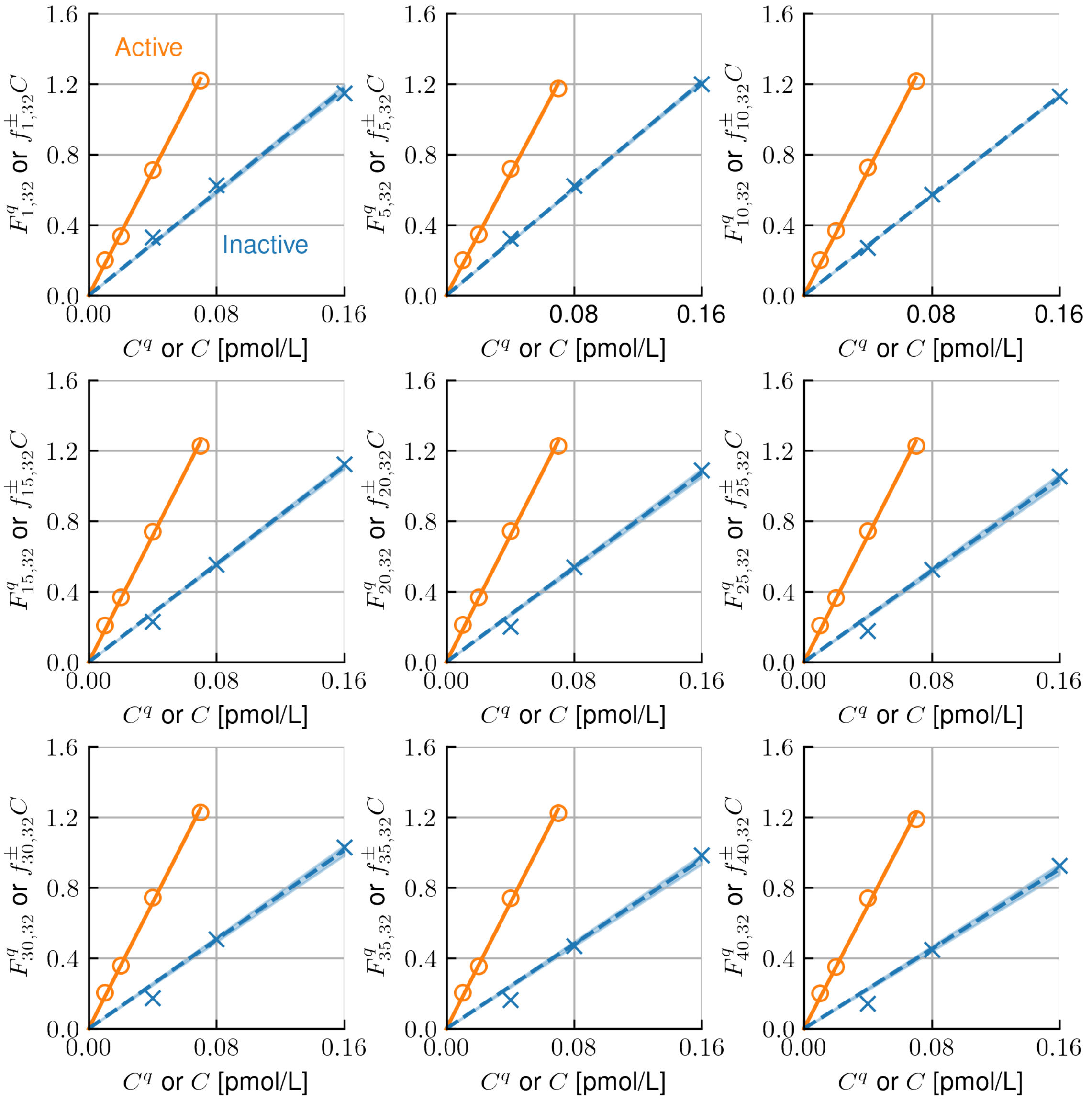

Fig. S32: As Figure S1 with well $w = 32$ (or C8).

Table S32: Molar Fluorescence Parameters for Well C8 ($w = 32$)

Cycle	Inactive		Active	
i	$f^-_{i,32}$	$\sigma^-_{i,32}$	$f^+_{i,32}$	$\sigma^+_{i,32}$
1	7.34	0.042	17.57	0.019
2	7.41	0.031	16.99	0.022
3	7.69	0.023	17.20	0.026
4	7.66	0.023	17.29	0.025
5	7.58	0.021	17.16	0.030
6	7.39	0.023	17.26	0.029
7	7.32	0.016	17.41	0.026
8	7.24	0.011	17.51	0.025
9	7.155	0.0080	17.57	0.024
10	7.08	0.011	17.65	0.024
11	7.01	0.014	17.73	0.021
12	6.98	0.019	17.75	0.021
13	7.03	0.027	17.79	0.021
14	6.99	0.031	17.84	0.025
15	6.95	0.035	17.88	0.027
16	6.89	0.037	17.90	0.027
17	6.84	0.040	17.89	0.028
18	6.81	0.044	17.90	0.028
19	6.76	0.047	17.92	0.027
20	6.71	0.049	17.90	0.029
21	6.66	0.052	17.89	0.028
22	6.61	0.054	17.89	0.028
23	6.56	0.056	17.91	0.028
24	6.54	0.059	17.89	0.028
25	6.48	0.060	17.88	0.028
26	6.43	0.054	17.88	0.028
27	6.42	0.056	17.87	0.028
28	6.39	0.057	17.91	0.027
29	6.33	0.057	17.87	0.027
30	6.31	0.058	17.85	0.027
31	6.26	0.058	17.86	0.026
32	6.24	0.059	17.80	0.026
33	6.17	0.058	17.83	0.026
34	6.04	0.058	17.79	0.026
35	6.00	0.056	17.79	0.026
36	5.95	0.048	17.90	0.023
37	5.90	0.048	17.94	0.021
38	5.83	0.045	17.68	0.028
39	5.73	0.059	17.49	0.032
40	5.65	0.060	17.43	0.034
41	5.65	0.059	17.30	0.037
42	5.61	0.059	17.19	0.043
43	5.48	0.055	17.15	0.041
44	5.32	0.070	17.03	0.044
45	5.33	0.059	16.86	0.040

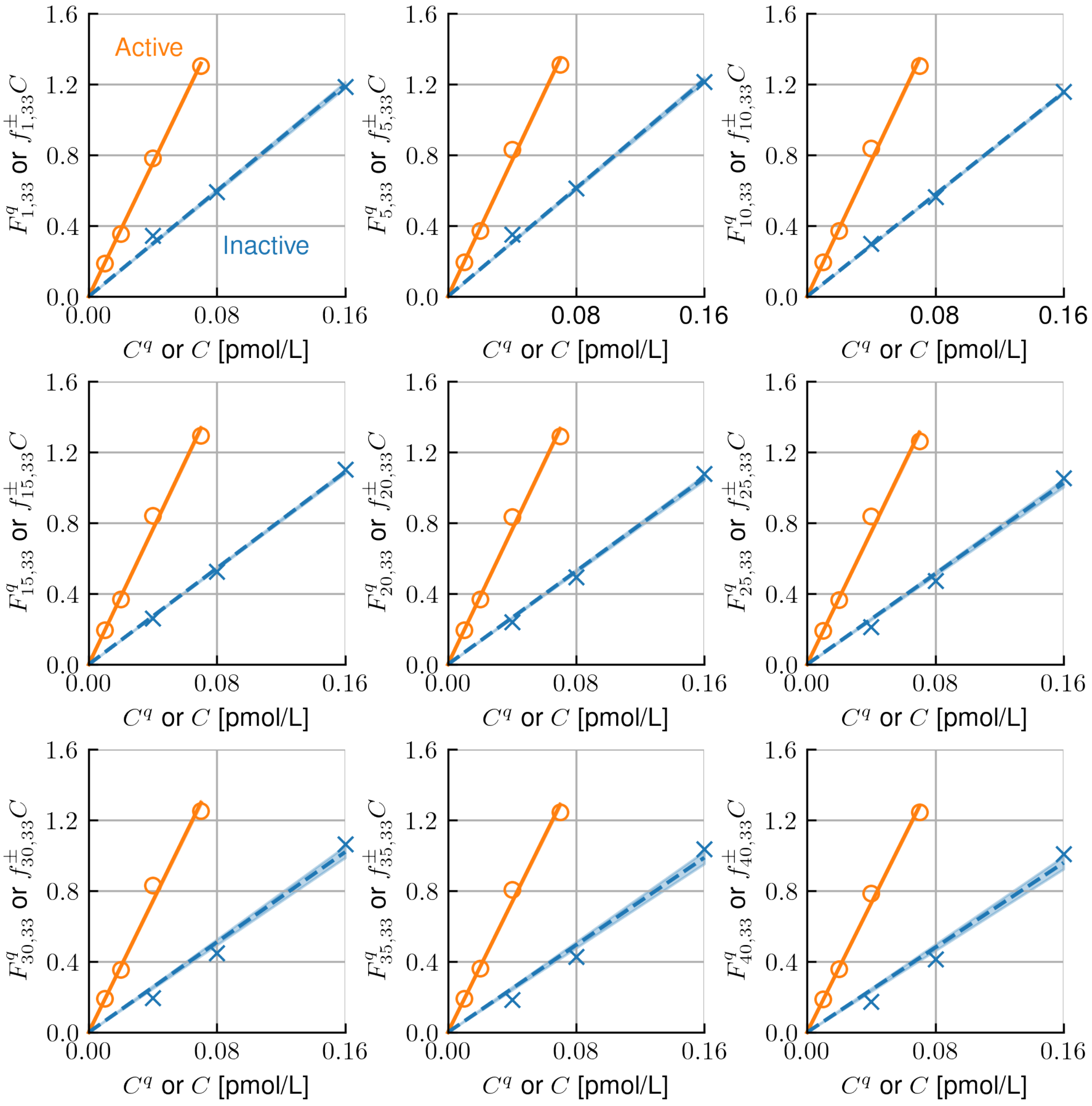

Fig. S33: As Figure S1 with well $w = 33$ (or C9).

Table S33: Molar Fluorescence Parameters for Well C9 ($w = 33$)

Cycle	Inactive		Active	
i	$f^-_{i,33}$	$\sigma^-_{i,33}$	$f^+_{i,33}$	$\sigma^+_{i,33}$
1	7.48	0.034	18.82	0.023
2	7.50	0.041	18.66	0.031
3	7.66	0.038	18.97	0.038
4	7.70	0.035	19.10	0.040
5	7.65	0.032	19.19	0.041
6	7.57	0.027	19.18	0.041
7	7.48	0.022	19.19	0.044
8	7.41	0.019	19.17	0.045
9	7.31	0.016	19.15	0.048
10	7.22	0.014	19.16	0.048
11	7.13	0.015	19.14	0.049
12	7.05	0.014	19.14	0.049
13	6.99	0.016	19.10	0.051
14	6.91	0.020	19.11	0.050
15	6.81	0.019	19.08	0.052
16	6.77	0.024	19.04	0.052
17	6.70	0.020	19.03	0.053
18	6.67	0.024	19.01	0.054
19	6.72	0.035	19.00	0.054
20	6.59	0.032	19.02	0.050
21	6.56	0.031	18.97	0.053
22	6.48	0.034	18.96	0.052
23	6.46	0.038	18.94	0.053
24	6.45	0.044	18.78	0.062
25	6.40	0.046	18.73	0.059
26	6.41	0.052	18.77	0.057
27	6.36	0.051	18.67	0.059
28	6.38	0.057	18.63	0.058
29	6.39	0.063	18.56	0.057
30	6.37	0.068	18.54	0.059
31	6.34	0.070	18.59	0.061
32	6.28	0.069	18.52	0.056
33	6.20	0.067	18.37	0.050
34	6.18	0.069	18.41	0.050
35	6.17	0.073	18.38	0.049
36	6.10	0.072	18.40	0.048
37	6.06	0.071	18.40	0.048
38	6.04	0.073	18.37	0.047
39	6.04	0.075	18.38	0.045
40	5.99	0.075	18.24	0.039
41	5.94	0.074	18.20	0.044
42	5.76	0.066	18.10	0.049
43	5.78	0.070	17.82	0.056
44	5.78	0.073	17.83	0.056
45	5.76	0.073	17.83	0.055

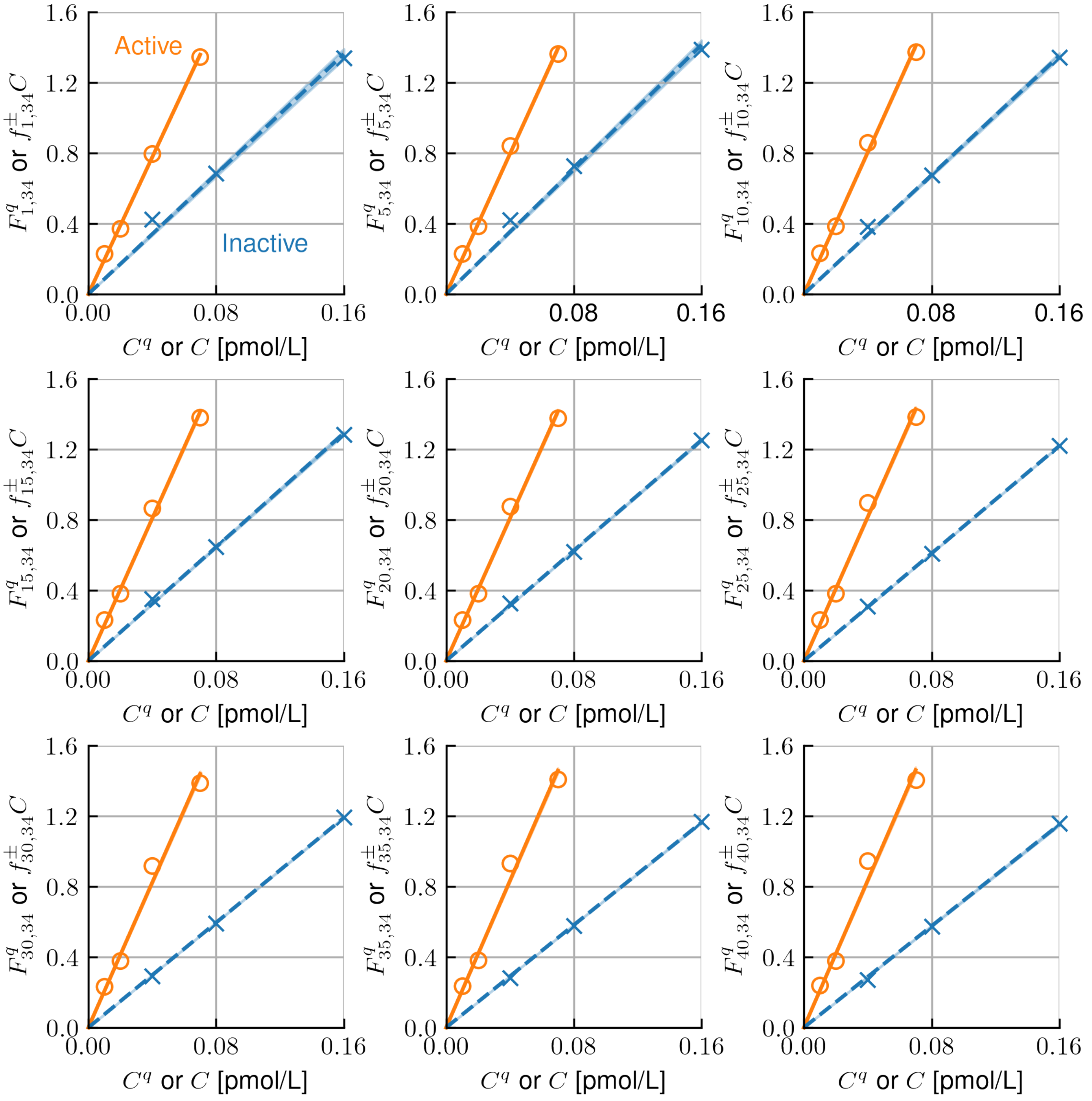

Fig. S34: As Figure S1 with well $w = 34$ (or C10).

Table S34: Molar Fluorescence Parameters for Well C10 ($w = 34$)

Cycle	Inactive		Active	
i	$f^-_{i,34}$	$\sigma^-_{i,34}$	$f^+_{i,34}$	$\sigma^+_{i,34}$
1	8.51	0.061	19.40	0.026
2	8.71	0.064	19.26	0.033
3	8.86	0.061	19.64	0.034
4	8.89	0.059	19.77	0.036
5	8.84	0.054	19.89	0.037
6	8.74	0.049	19.94	0.038
7	8.66	0.044	20.03	0.040
8	8.55	0.041	20.07	0.041
9	8.50	0.036	20.10	0.041
10	8.45	0.031	20.09	0.042
11	8.33	0.030	20.12	0.041
12	8.24	0.029	20.13	0.041
13	8.16	0.027	20.14	0.044
14	8.09	0.025	20.16	0.044
15	8.07	0.021	20.17	0.043
16	8.02	0.019	20.18	0.045
17	7.98	0.016	20.19	0.045
18	7.92	0.015	20.19	0.046
19	7.90	0.013	20.18	0.047
20	7.83	0.012	20.20	0.049
21	7.803	0.0085	20.25	0.050
22	7.756	0.0073	20.28	0.051
23	7.710	0.0059	20.31	0.053
24	7.684	0.0041	20.30	0.054
25	7.622	0.0034	20.39	0.058
26	7.585	0.0034	20.43	0.060
27	7.540	0.0023	20.45	0.064
28	7.508	0.0026	20.52	0.064
29	7.470	0.0031	20.47	0.066
30	7.441	0.0057	20.56	0.066
31	7.402	0.0061	20.56	0.069
32	7.362	0.0072	20.61	0.068
33	7.332	0.0062	20.65	0.070
34	7.303	0.0069	20.86	0.066
35	7.274	0.0066	20.82	0.068
36	7.218	0.0091	20.80	0.072
37	7.25	0.011	20.88	0.072
38	7.27	0.012	20.88	0.075
39	7.24	0.011	20.89	0.076
40	7.20	0.013	20.86	0.077
41	7.19	0.014	20.87	0.080
42	7.16	0.019	20.86	0.081
43	7.15	0.020	20.92	0.082
44	7.08	0.018	20.90	0.084
45	7.08	0.019	20.96	0.085

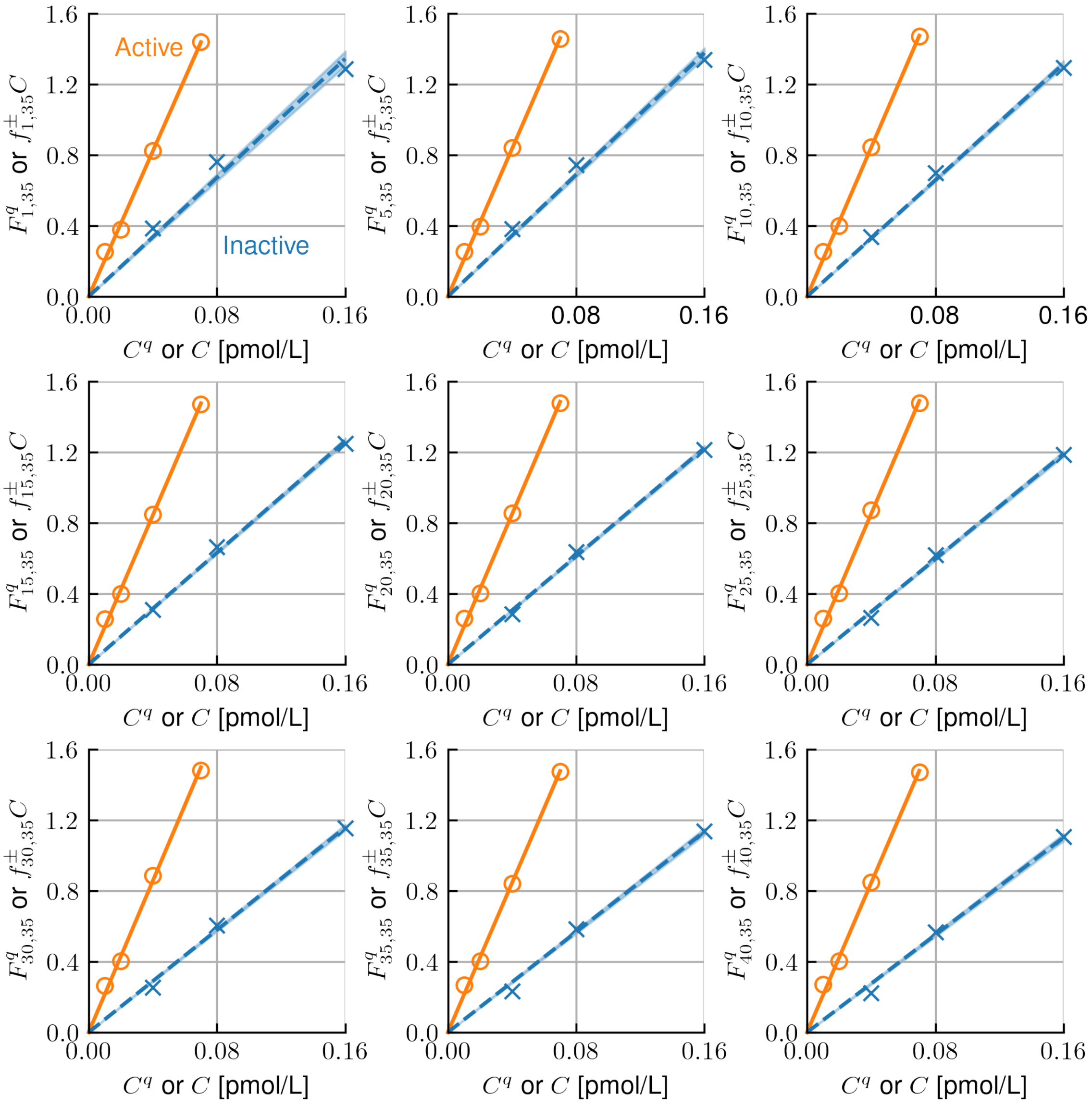

Fig. S35: As Figure S1 with well $w = 35$ (or C11).

Table S35: Molar Fluorescence Parameters for Well C11 ($w = 35$)

Cycle	Inactive		Active	
i	$f^-_{i,35}$	$\sigma^-_{i,35}$	$f^+_{i,35}$	$\sigma^+_{i,35}$
1	8.40	0.084	20.54	0.033
2	8.55	0.074	20.36	0.030
3	8.69	0.066	20.62	0.029
4	8.68	0.062	20.78	0.029
5	8.61	0.055	20.87	0.029
6	8.53	0.048	20.93	0.029
7	8.46	0.043	20.98	0.029
8	8.37	0.039	21.05	0.029
9	8.30	0.036	21.06	0.029
10	8.22	0.033	21.06	0.028
11	8.15	0.032	21.13	0.028
12	8.08	0.031	21.09	0.029
13	8.01	0.029	21.12	0.029
14	7.96	0.028	21.10	0.029
15	7.90	0.027	21.08	0.029
16	7.83	0.025	21.09	0.029
17	7.78	0.026	21.09	0.029
18	7.74	0.025	21.07	0.030
19	7.68	0.024	21.20	0.031
20	7.64	0.024	21.21	0.031
21	7.64	0.024	21.18	0.032
22	7.57	0.025	21.17	0.032
23	7.52	0.026	21.21	0.032
24	7.47	0.027	21.29	0.033
25	7.44	0.028	21.28	0.035
26	7.42	0.029	21.25	0.035
27	7.36	0.029	21.26	0.036
28	7.30	0.032	21.28	0.036
29	7.25	0.033	21.30	0.036
30	7.24	0.031	21.40	0.038
31	7.23	0.033	21.37	0.037
32	7.20	0.035	21.37	0.040
33	7.16	0.034	21.22	0.036
34	7.13	0.036	21.13	0.035
35	7.09	0.037	21.10	0.035
36	7.03	0.037	21.07	0.034
37	6.98	0.038	21.12	0.035
38	6.95	0.039	21.05	0.036
39	6.91	0.040	21.29	0.036
40	6.88	0.039	21.13	0.036
41	6.87	0.039	21.02	0.038
42	6.85	0.037	21.09	0.038
43	6.84	0.039	21.05	0.039
44	6.85	0.041	21.08	0.039
45	6.92	0.043	21.04	0.040

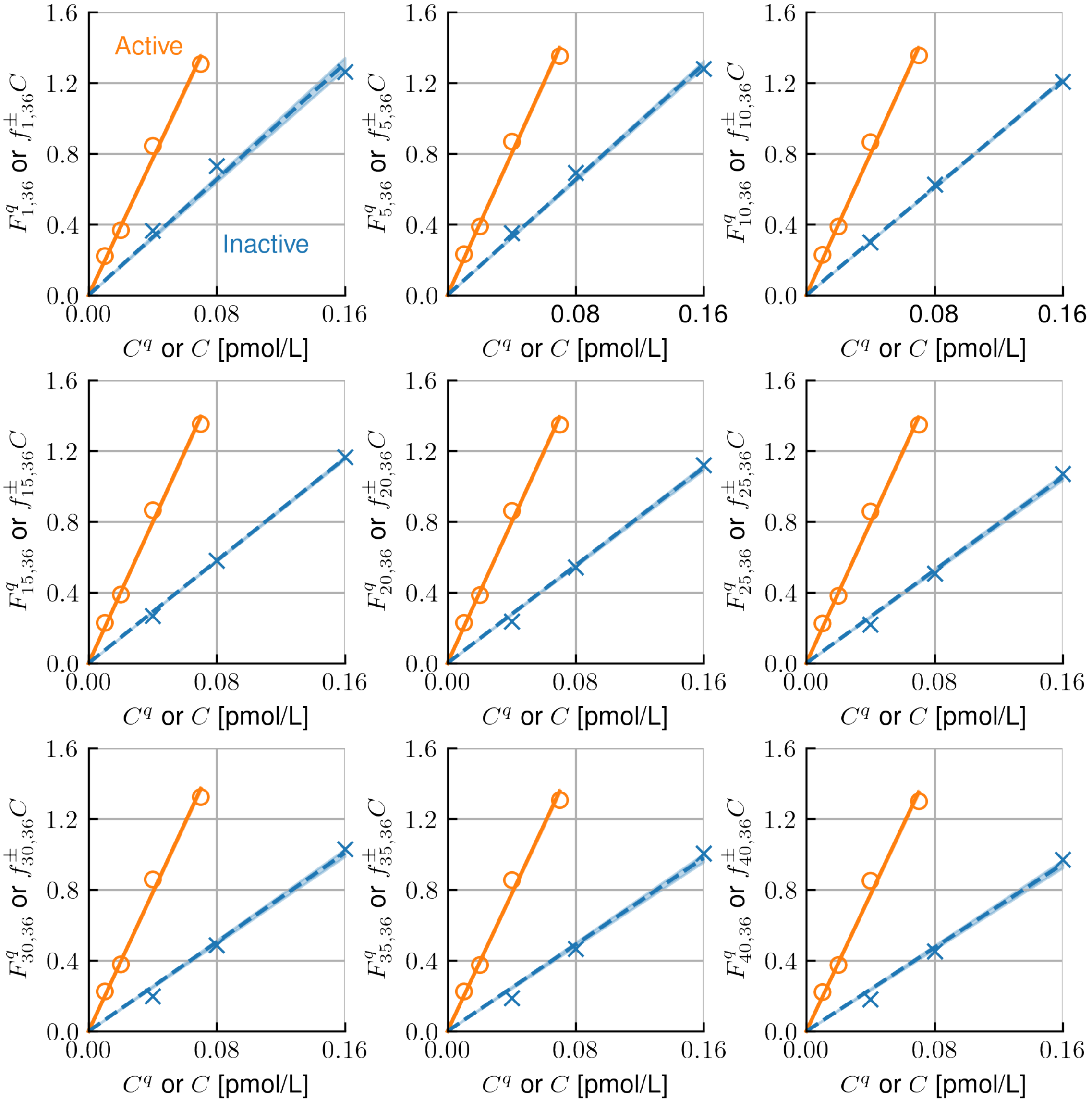

Fig. S36: As Figure S1 with well $w = 36$ (or C12).

Table S36: Molar Fluorescence Parameters for Well C12 ($w = 36$)

Cycle	Inactive		Active	
i	$f^-_{i,36}$	$\sigma^-_{i,36}$	$f^+_{i,36}$	$\sigma^+_{i,36}$
1	8.18	0.069	19.27	0.053
2	8.30	0.062	19.73	0.047
3	8.38	0.054	19.86	0.051
4	8.27	0.047	19.97	0.052
5	8.16	0.038	19.96	0.053
6	8.04	0.031	19.98	0.052
7	7.91	0.026	19.97	0.052
8	7.83	0.019	19.96	0.052
9	7.72	0.016	19.94	0.052
10	7.60	0.015	19.95	0.050
11	7.52	0.013	19.94	0.052
12	7.45	0.013	19.90	0.050
13	7.37	0.014	19.90	0.051
14	7.31	0.016	19.89	0.051
15	7.24	0.017	19.90	0.050
16	7.18	0.018	19.88	0.048
17	7.10	0.022	19.93	0.051
18	7.01	0.023	19.88	0.051
19	6.98	0.025	19.87	0.050
20	6.91	0.030	19.85	0.049
21	6.85	0.033	19.85	0.050
22	6.74	0.035	19.86	0.051
23	6.67	0.037	19.86	0.046
24	6.64	0.039	19.85	0.048
25	6.57	0.036	19.84	0.048
26	6.53	0.043	19.72	0.051
27	6.50	0.043	19.71	0.050
28	6.44	0.046	19.80	0.050
29	6.39	0.045	19.79	0.049
30	6.29	0.043	19.57	0.055
31	6.27	0.045	19.58	0.054
32	6.21	0.048	19.57	0.054
33	6.17	0.046	19.43	0.058
34	6.11	0.042	19.40	0.058
35	6.12	0.047	19.36	0.058
36	6.07	0.046	19.40	0.057
37	6.02	0.047	19.46	0.057
38	5.98	0.046	19.37	0.057
39	5.99	0.049	19.32	0.057
40	5.90	0.046	19.28	0.058
41	5.88	0.046	19.15	0.063
42	5.84	0.045	19.25	0.060
43	5.81	0.045	19.16	0.060
44	5.77	0.044	19.19	0.058
45	5.73	0.045	18.95	0.044

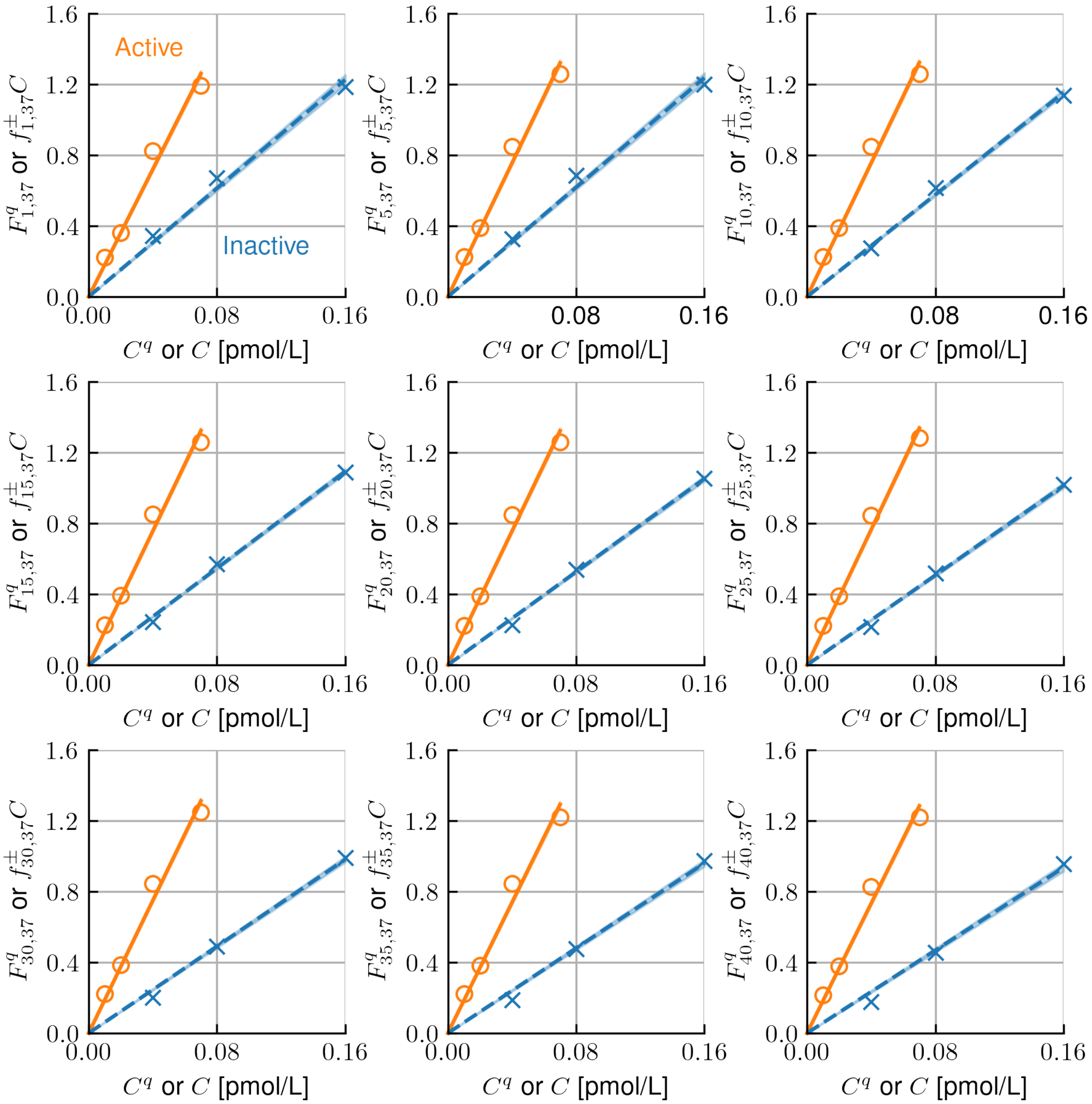

Fig. S37: As Figure S1 with well $w = 37$ (or D1).

Table S37: Molar Fluorescence Parameters for Well D1 ($w = 37$)

Cycle	Inactive		Active	
i	$f^-_{i,37}$	$\sigma^-_{i,37}$	$f^+_{i,37}$	$\sigma^+_{i,37}$
1	7.66	0.057	18.00	0.076
2	7.93	0.062	18.47	0.067
3	7.96	0.065	18.69	0.069
4	7.86	0.061	18.78	0.069
5	7.73	0.056	18.87	0.069
6	7.61	0.048	18.86	0.069
7	7.50	0.042	18.89	0.069
8	7.39	0.039	18.87	0.069
9	7.30	0.034	18.89	0.069
10	7.21	0.032	18.89	0.069
11	7.13	0.030	18.89	0.070
12	7.05	0.029	18.91	0.070
13	6.99	0.028	18.92	0.071
14	6.88	0.028	18.90	0.070
15	6.83	0.027	18.91	0.071
16	6.79	0.028	18.89	0.071
17	6.70	0.028	18.88	0.071
18	6.65	0.028	18.90	0.072
19	6.61	0.027	18.86	0.073
20	6.58	0.028	18.90	0.068
21	6.58	0.030	18.83	0.069
22	6.59	0.031	18.85	0.070
23	6.50	0.030	18.84	0.073
24	6.40	0.029	18.85	0.069
25	6.33	0.029	19.11	0.060
26	6.29	0.028	18.89	0.065
27	6.25	0.028	18.86	0.065
28	6.21	0.030	18.80	0.066
29	6.18	0.031	18.75	0.068
30	6.13	0.032	18.74	0.068
31	6.10	0.033	18.83	0.066
32	6.07	0.034	18.42	0.077
33	6.04	0.035	18.44	0.077
34	6.00	0.036	18.47	0.076
35	6.00	0.037	18.45	0.078
36	5.96	0.039	18.41	0.071
37	5.95	0.039	18.37	0.070
38	5.89	0.041	18.38	0.068
39	5.87	0.043	18.32	0.069
40	5.85	0.043	18.32	0.068
41	5.82	0.045	18.37	0.065
42	5.80	0.046	18.32	0.066
43	5.80	0.048	18.27	0.068
44	5.75	0.048	18.26	0.067
45	5.73	0.049	18.09	0.073

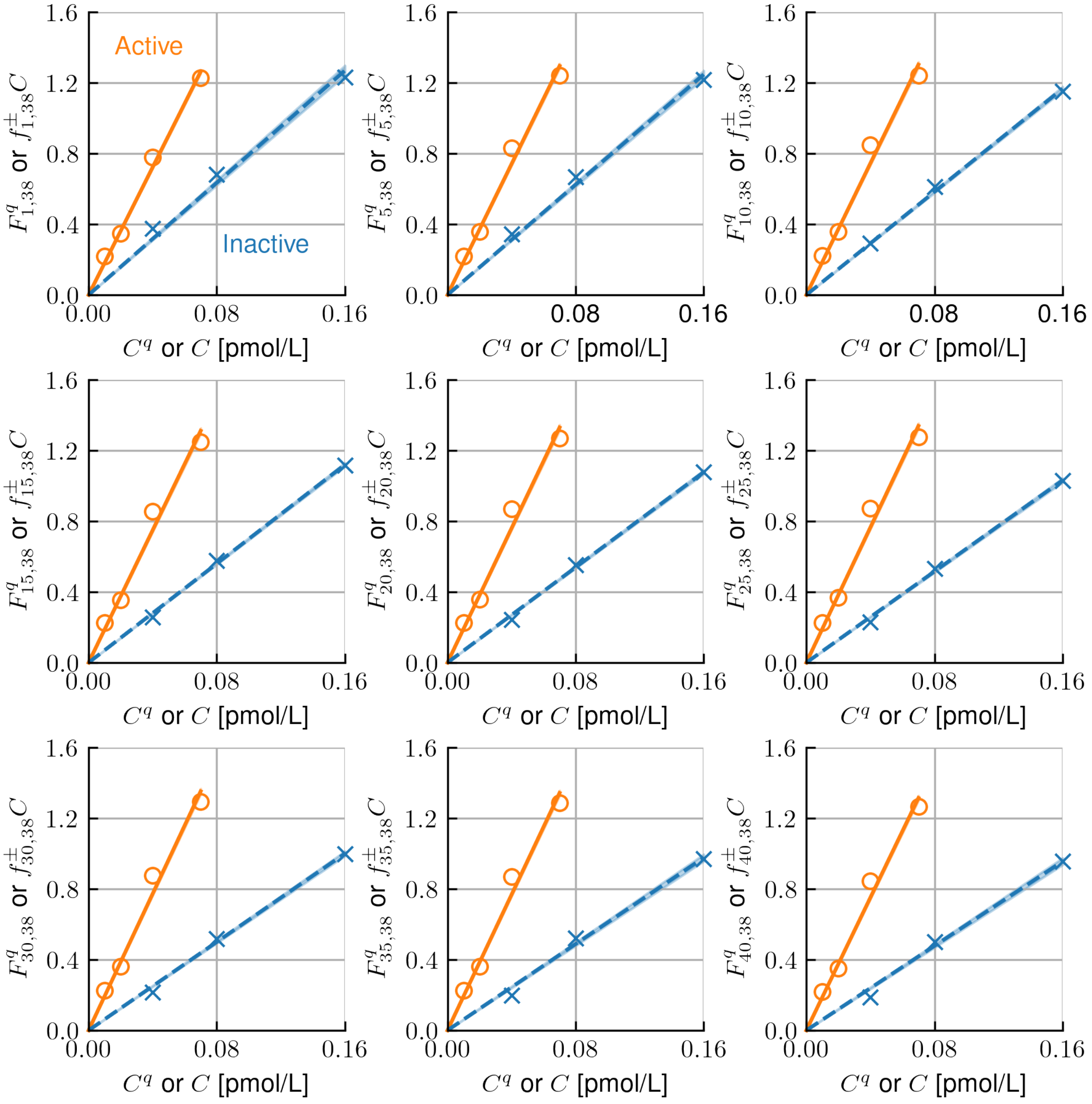

Fig. S38: As Figure S1 with well $w = 38$ (or D2).

Table S38: Molar Fluorescence Parameters for Well D2 ($w = 38$)

Cycle	Inactive		Active	
i	$f^-_{i,38}$	$\sigma^-_{i,38}$	$f^+_{i,38}$	$\sigma^+_{i,38}$
1	7.93	0.059	18.05	0.046
2	8.06	0.058	18.09	0.057
3	8.09	0.055	18.28	0.063
4	7.94	0.052	18.44	0.065
5	7.79	0.046	18.50	0.065
6	7.69	0.038	18.51	0.068
7	7.59	0.032	18.54	0.069
8	7.49	0.027	18.59	0.069
9	7.39	0.025	18.60	0.072
10	7.29	0.024	18.61	0.074
11	7.20	0.019	18.62	0.074
12	7.14	0.018	18.59	0.076
13	7.07	0.019	18.65	0.075
14	7.02	0.019	18.71	0.076
15	7.00	0.020	18.73	0.075
16	6.95	0.020	18.78	0.075
17	6.87	0.024	18.82	0.075
18	6.81	0.023	18.91	0.074
19	6.84	0.023	18.97	0.076
20	6.74	0.021	19.00	0.077
21	6.66	0.027	19.03	0.075
22	6.60	0.026	19.06	0.074
23	6.54	0.025	19.02	0.076
24	6.50	0.024	19.03	0.076
25	6.45	0.023	19.13	0.074
26	6.47	0.026	19.15	0.076
27	6.38	0.028	19.20	0.074
28	6.32	0.027	19.21	0.075
29	6.28	0.026	19.24	0.074
30	6.25	0.027	19.32	0.073
31	6.20	0.029	19.27	0.074
32	6.15	0.031	19.26	0.076
33	6.12	0.034	19.22	0.072
34	6.11	0.035	19.19	0.070
35	6.10	0.040	19.18	0.071
36	6.06	0.037	19.20	0.070
37	6.03	0.037	19.10	0.066
38	6.02	0.038	18.88	0.074
39	5.98	0.038	18.87	0.074
40	5.97	0.040	18.78	0.065
41	5.97	0.040	18.81	0.066
42	5.95	0.041	18.80	0.067
43	5.94	0.042	18.77	0.068
44	5.92	0.043	18.67	0.072
45	5.89	0.045	18.71	0.071

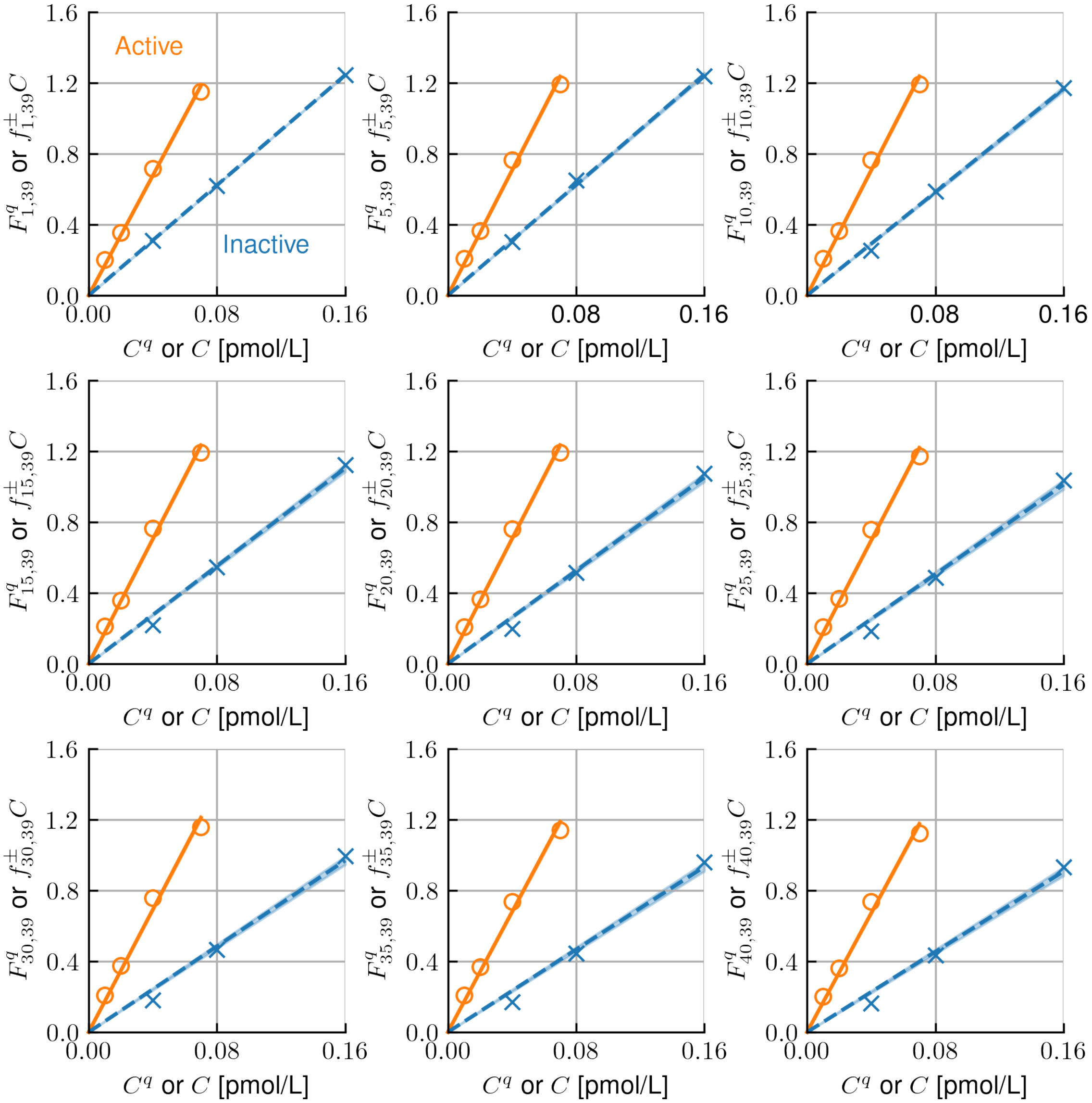

Fig. S39: As Figure S1 with well $w = 39$ (or D3).

Table S39: Molar Fluorescence Parameters for Well D3 ($w = 39$)

Cycle	Inactive		Active	
i	$f^-_{i,39}$	$\sigma^-_{i,39}$	$f^+_{i,39}$	$\sigma^+_{i,39}$
1	7.779	0.0033	16.92	0.037
2	7.91	0.018	17.20	0.040
3	7.99	0.022	17.43	0.043
4	7.92	0.023	17.56	0.046
5	7.80	0.021	17.64	0.046
6	7.69	0.019	17.69	0.046
7	7.58	0.020	17.71	0.045
8	7.48	0.022	17.66	0.047
9	7.38	0.026	17.68	0.046
10	7.29	0.028	17.66	0.047
11	7.20	0.032	17.65	0.046
12	7.12	0.035	17.64	0.047
13	7.04	0.037	17.64	0.047
14	6.97	0.040	17.64	0.047
15	6.90	0.042	17.62	0.046
16	6.83	0.044	17.65	0.045
17	6.74	0.045	17.64	0.045
18	6.68	0.046	17.64	0.045
19	6.63	0.047	17.64	0.044
20	6.57	0.049	17.63	0.046
21	6.51	0.050	17.66	0.046
22	6.46	0.051	17.60	0.046
23	6.47	0.055	17.54	0.048
24	6.39	0.055	17.41	0.051
25	6.32	0.055	17.41	0.051
26	6.33	0.058	17.36	0.053
27	6.22	0.049	17.37	0.053
28	6.16	0.046	17.32	0.054
29	6.13	0.048	17.28	0.054
30	6.06	0.048	17.30	0.055
31	6.01	0.047	17.48	0.050
32	5.97	0.048	17.32	0.052
33	5.94	0.048	17.25	0.053
34	5.89	0.049	17.20	0.056
35	5.83	0.050	16.96	0.052
36	5.81	0.050	17.01	0.052
37	5.79	0.051	16.96	0.053
38	5.73	0.051	16.93	0.054
39	5.69	0.051	16.89	0.055
40	5.67	0.051	16.79	0.054
41	5.68	0.054	16.79	0.054
42	5.60	0.054	16.70	0.058
43	5.53	0.051	16.73	0.057
44	5.52	0.053	16.66	0.057
45	5.48	0.053	16.65	0.057

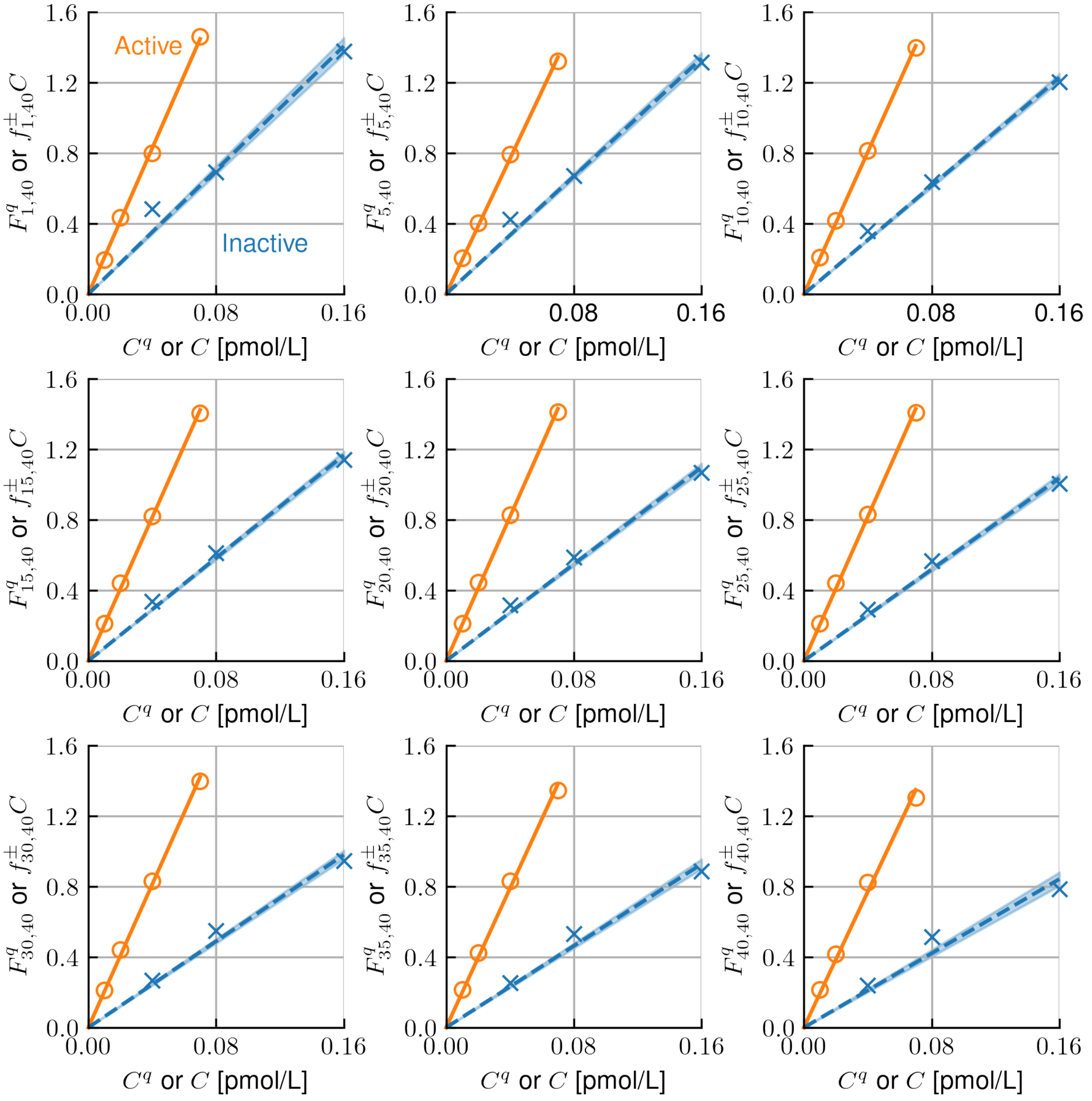

Fig. S40: As Figure S1 with well $w = 40$ (or D4).

Table S40: Molar Fluorescence Parameters for Well D4 ($w = 40$)

Cycle	Inactive		Active	
i	$f_{i,40}^{-}$	$\sigma_{i,40}^{-}$	$f_{i,40}^{+}$	$\sigma_{i,40}^{+}$
1	8.79	0.096	20.68	0.022
2	8.42	0.074	19.53	0.011
3	8.54	0.069	19.41	0.013
4	8.49	0.069	19.39	0.016
5	8.36	0.065	19.20	0.024
6	8.21	0.060	19.35	0.022
7	8.05	0.054	19.71	0.014
8	7.92	0.051	19.92	0.010
9	7.80	0.047	20.031	0.0096
10	7.68	0.044	20.13	0.013
11	7.54	0.032	20.21	0.014
12	7.45	0.039	20.27	0.017
13	7.38	0.049	20.30	0.020
14	7.35	0.045	20.32	0.022
15	7.29	0.043	20.33	0.024
16	7.21	0.042	20.35	0.019
17	7.12	0.042	20.35	0.022
18	7.03	0.043	20.39	0.023
19	6.95	0.044	20.42	0.025
20	6.86	0.046	20.43	0.025
21	6.78	0.045	20.42	0.027
22	6.69	0.047	20.45	0.028
23	6.62	0.046	20.45	0.026
24	6.55	0.046	20.45	0.025
25	6.48	0.046	20.41	0.025
26	6.40	0.047	20.47	0.023
27	6.33	0.047	20.45	0.023
28	6.26	0.047	20.45	0.024
29	6.19	0.049	20.50	0.022
30	6.12	0.051	20.31	0.027
31	6.04	0.052	20.21	0.027
32	5.97	0.055	20.07	0.029
33	5.92	0.055	19.95	0.031
34	5.85	0.058	19.88	0.034
35	5.79	0.058	19.73	0.037
36	5.75	0.060	19.64	0.039
37	5.69	0.060	19.48	0.042
38	5.38	0.078	19.42	0.045
39	5.32	0.071	19.46	0.042
40	5.26	0.080	19.25	0.047
41	5.15	0.076	19.01	0.047
42	5.10	0.072	18.91	0.051
43	5.03	0.071	18.75	0.056
44	4.96	0.072	18.50	0.045
45	4.90	0.072	18.22	0.054

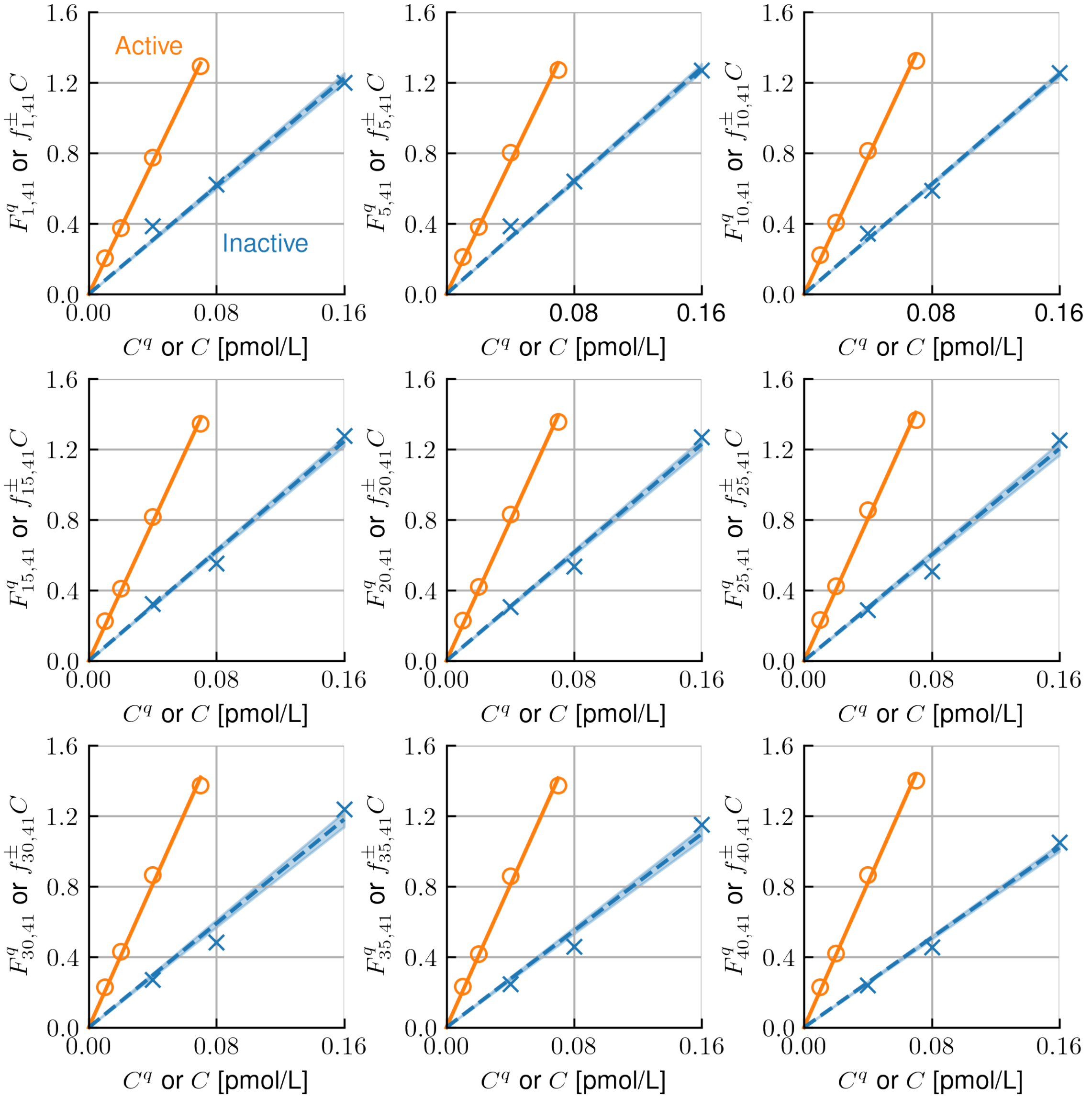

Fig. S41: As Figure S1 with well $w = 41$ (or D5).

Table S41: Molar Fluorescence Parameters for Well D5 ($w = 41$)

Cycle	Inactive		Active	
i	$f^-_{i,41}$	$\sigma^-_{i,41}$	$f^+_{i,41}$	$\sigma^+_{i,41}$
1	7.65	0.060	18.71	0.020
2	7.63	0.057	18.15	0.030
3	7.85	0.054	18.43	0.035
4	7.93	0.053	18.59	0.039
5	8.03	0.047	18.72	0.040
6	7.97	0.042	18.79	0.041
7	7.93	0.038	19.02	0.037
8	7.88	0.035	19.13	0.037
9	7.84	0.034	19.36	0.034
10	7.80	0.035	19.39	0.035
11	7.75	0.035	19.48	0.033
12	7.71	0.037	19.50	0.033
13	7.76	0.043	19.56	0.033
14	7.80	0.050	19.59	0.032
15	7.78	0.055	19.62	0.032
16	7.79	0.054	19.67	0.031
17	7.75	0.055	19.70	0.031
18	7.71	0.056	19.72	0.033
19	7.72	0.061	19.77	0.032
20	7.68	0.062	19.86	0.036
21	7.65	0.067	19.87	0.036
22	7.63	0.069	19.94	0.039
23	7.55	0.069	20.01	0.043
24	7.52	0.071	20.05	0.043
25	7.51	0.075	20.10	0.043
26	7.46	0.076	20.13	0.043
27	7.47	0.079	20.16	0.045
28	7.45	0.082	20.30	0.041
29	7.42	0.085	20.24	0.046
30	7.38	0.088	20.26	0.047
31	7.22	0.084	20.22	0.041
32	7.19	0.080	20.22	0.039
33	7.16	0.085	20.24	0.039
34	7.12	0.086	20.28	0.038
35	6.86	0.076	20.19	0.042
36	6.81	0.077	20.26	0.040
37	6.78	0.081	20.34	0.038
38	6.38	0.055	20.36	0.038
39	6.36	0.055	20.44	0.036
40	6.37	0.045	20.50	0.036
41	6.32	0.047	20.26	0.044
42	6.36	0.060	20.32	0.044
43	6.35	0.061	20.39	0.044
44	6.32	0.063	20.41	0.045
45	6.30	0.065	20.36	0.044

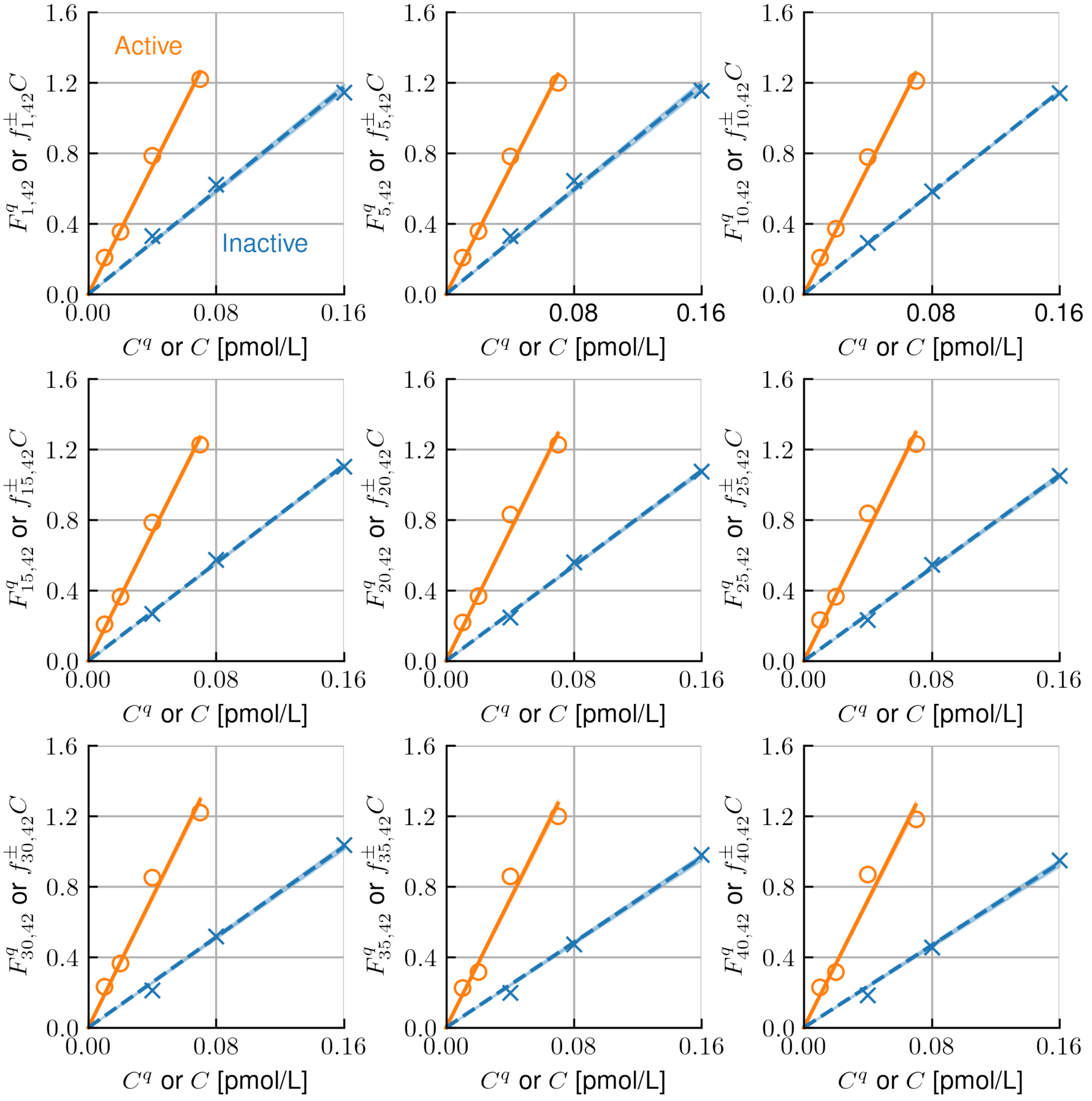

Fig. S42: As Figure S1 with well $w = 42$ (or D6).

Table S42: Molar Fluorescence Parameters for Well D6 ($w = 42$)

Cycle	Inactive		Active	
i	$f^-_{i,42}$	$\sigma^-_{i,42}$	$f^+_{i,42}$	$\sigma^+_{i,42}$
1	7.32	0.042	17.99	0.047
2	7.15	0.049	17.39	0.048
3	7.37	0.047	17.62	0.050
4	7.45	0.049	17.70	0.052
5	7.41	0.048	17.79	0.051
6	7.32	0.046	17.83	0.051
7	7.27	0.036	17.86	0.051
8	7.24	0.025	17.89	0.050
9	7.24	0.014	17.92	0.050
10	7.18	0.010	17.91	0.049
11	7.14	0.013	17.94	0.048
12	7.09	0.011	17.95	0.048
13	7.050	0.0095	17.97	0.047
14	7.00	0.012	18.05	0.049
15	6.94	0.017	18.10	0.046
16	6.90	0.017	18.19	0.047
17	6.86	0.018	18.29	0.052
18	6.81	0.019	18.42	0.061
19	6.76	0.021	18.45	0.065
20	6.74	0.022	18.40	0.068
21	6.71	0.024	18.39	0.068
22	6.67	0.024	18.40	0.070
23	6.62	0.025	18.43	0.071
24	6.62	0.024	18.47	0.073
25	6.58	0.026	18.47	0.074
26	6.56	0.027	18.62	0.072
27	6.51	0.026	18.52	0.076
28	6.49	0.029	18.51	0.078
29	6.46	0.031	18.50	0.079
30	6.41	0.033	18.45	0.082
31	6.36	0.034	18.46	0.082
32	6.33	0.035	18.48	0.084
33	6.16	0.032	18.38	0.085
34	6.12	0.033	18.35	0.087
35	6.03	0.034	18.15	0.094
36	6.01	0.036	18.11	0.097
37	5.99	0.037	18.06	0.099
38	5.96	0.038	18.12	0.099
39	5.71	0.028	18.0	0.10
40	5.83	0.037	18.0	0.11
41	5.80	0.037	17.9	0.10
42	5.78	0.038	17.9	0.10
43	5.76	0.040	17.9	0.11
44	5.77	0.040	17.0	0.13
45	5.74	0.043	16.9	0.13

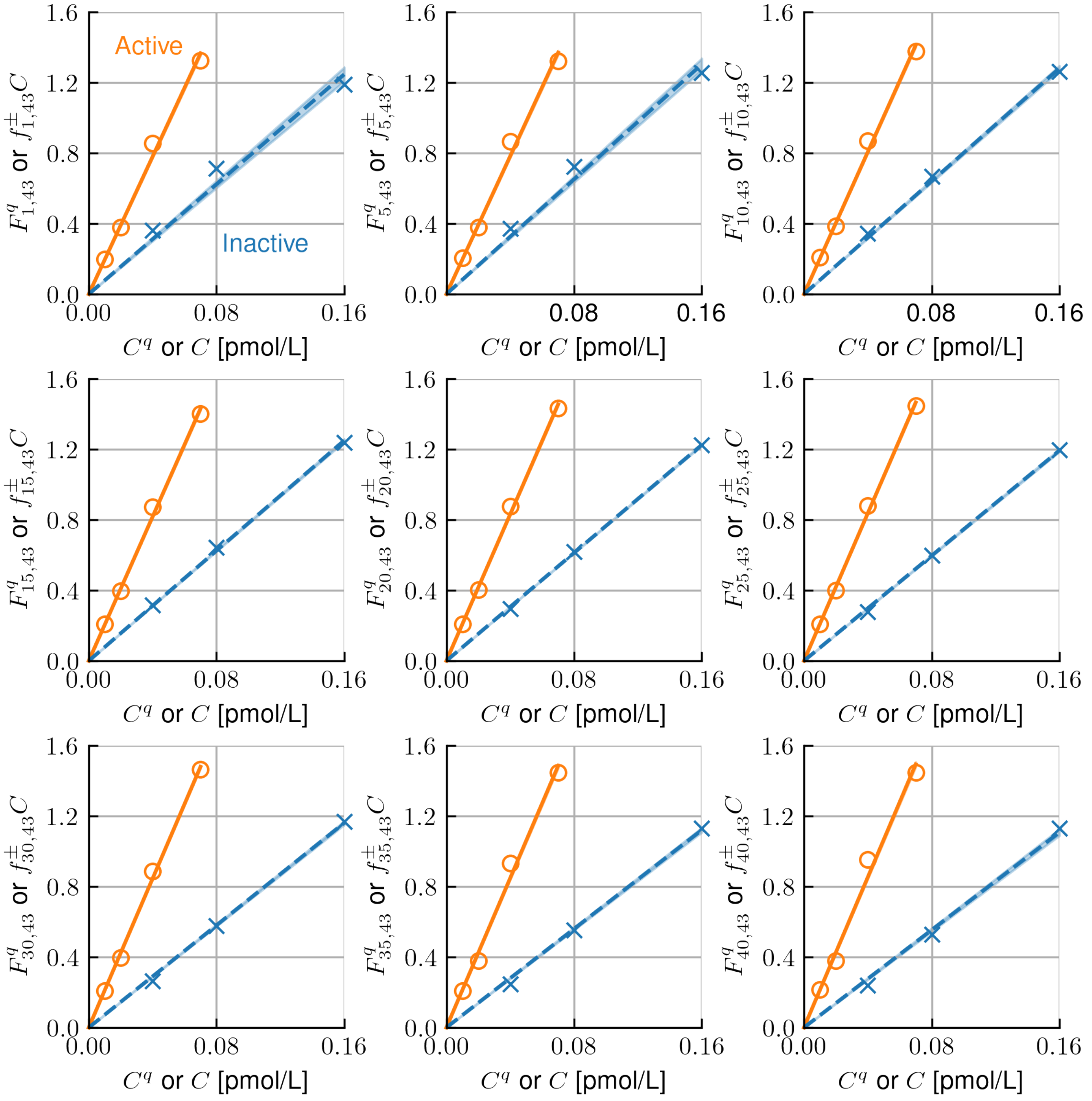

Fig. S43: As Figure S1 with well $w = 43$ (or D7).

Table S43: Molar Fluorescence Parameters for Well D7 ($w = 43$)

Cycle	Inactive		Active	
i	$f^-_{i,43}$	$\sigma^-_{i,43}$	$f^+_{i,43}$	$\sigma^+_{i,43}$
1	7.79	0.082	19.50	0.049
2	7.82	0.075	19.02	0.054
3	8.01	0.074	19.27	0.056
4	8.13	0.071	19.46	0.056
5	8.14	0.069	19.56	0.056
6	8.10	0.062	19.86	0.050
7	8.13	0.048	19.92	0.049
8	8.08	0.040	20.00	0.047
9	8.06	0.034	20.08	0.045
10	8.01	0.029	20.16	0.043
11	7.99	0.027	20.18	0.043
12	7.95	0.023	20.28	0.042
13	7.91	0.020	20.32	0.041
14	7.86	0.017	20.36	0.040
15	7.81	0.015	20.44	0.037
16	7.80	0.013	20.49	0.037
17	7.743	0.0098	20.55	0.033
18	7.698	0.0083	20.64	0.034
19	7.650	0.0091	20.73	0.032
20	7.656	0.0080	20.78	0.031
21	7.562	0.0082	20.88	0.029
22	7.519	0.0085	20.90	0.031
23	7.482	0.0089	20.86	0.030
24	7.48	0.010	20.90	0.029
25	7.46	0.014	20.93	0.029
26	7.43	0.013	20.99	0.030
27	7.38	0.015	21.03	0.029
28	7.36	0.017	21.08	0.028
29	7.33	0.020	21.13	0.029
30	7.25	0.020	21.13	0.029
31	7.24	0.022	20.86	0.035
32	7.07	0.018	20.92	0.035
33	7.06	0.019	20.95	0.034
34	7.03	0.025	21.13	0.049
35	7.00	0.025	21.19	0.059
36	7.00	0.027	21.09	0.063
37	6.97	0.031	21.16	0.061
38	6.95	0.034	21.23	0.064
39	6.93	0.035	21.30	0.066
40	6.92	0.036	21.33	0.069
41	6.93	0.040	21.39	0.069
42	6.91	0.042	21.36	0.072
43	6.81	0.037	21.50	0.071
44	6.39	0.020	21.51	0.073
45	6.38	0.020	20.79	0.079

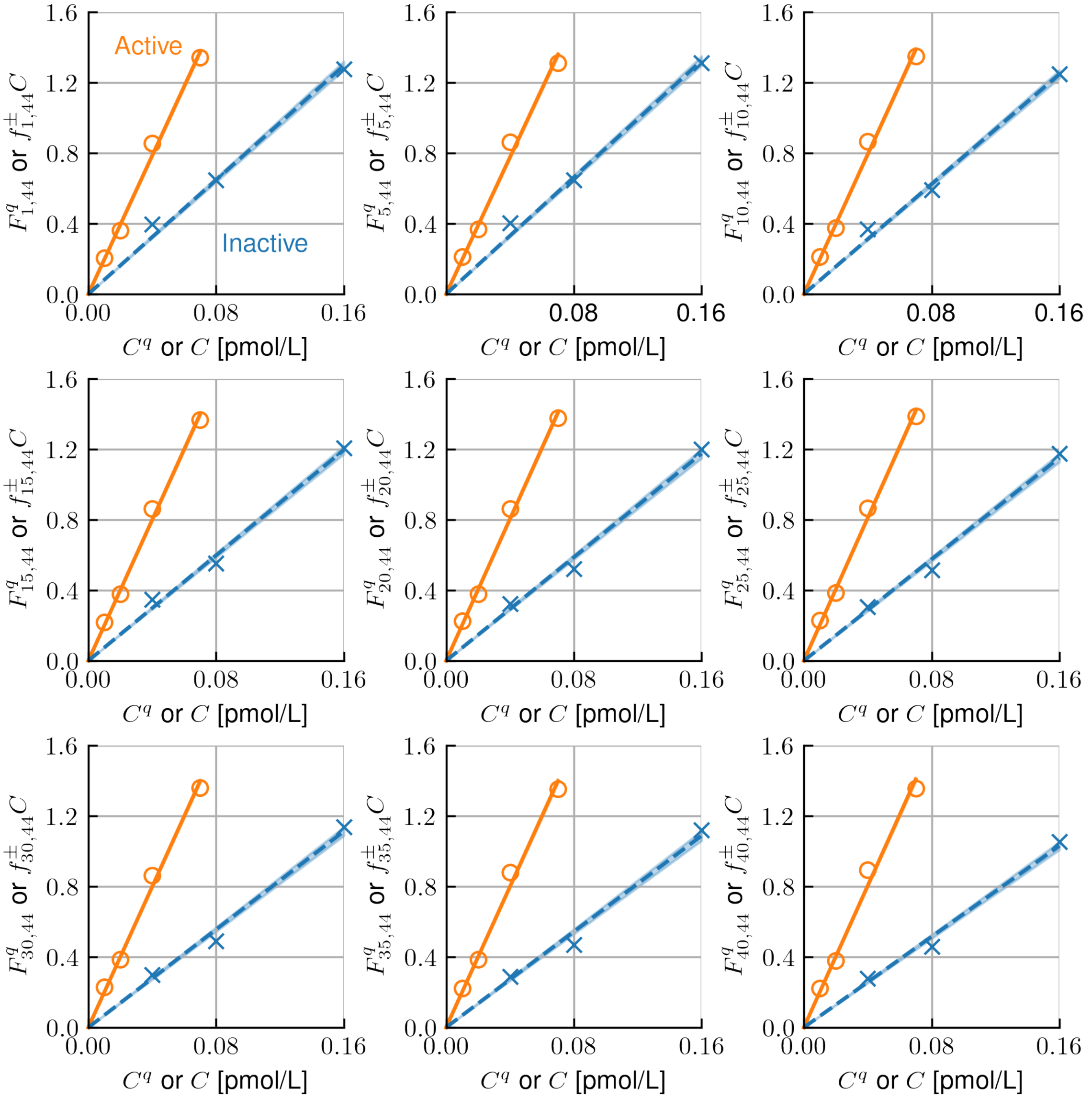

Fig. S44: As Figure S1 with well $w = 44$ (or D8).

Table S44: Molar Fluorescence Parameters for Well D8 ($w = 44$)

Cycle	Inactive		Active	
i	$f^-_{i,44}$	$\sigma^-_{i,44}$	$f^+_{i,44}$	$\sigma^+_{i,44}$
1	8.09	0.052	19.63	0.048
2	8.04	0.055	18.98	0.056
3	8.21	0.054	19.15	0.058
4	8.30	0.053	19.33	0.059
5	8.27	0.053	19.40	0.058
6	8.20	0.050	19.46	0.057
7	8.13	0.049	19.65	0.053
8	8.02	0.048	19.71	0.052
9	7.89	0.048	19.77	0.049
10	7.79	0.047	19.81	0.049
11	7.70	0.047	19.88	0.048
12	7.61	0.046	19.92	0.047
13	7.54	0.045	19.92	0.046
14	7.52	0.047	19.93	0.046
15	7.47	0.048	19.98	0.045
16	7.45	0.049	20.03	0.044
17	7.38	0.048	20.05	0.044
18	7.36	0.051	20.10	0.046
19	7.36	0.052	20.11	0.043
20	7.34	0.053	20.11	0.043
21	7.30	0.053	20.15	0.042
22	7.27	0.051	20.17	0.042
23	7.28	0.051	20.19	0.041
24	7.23	0.048	20.27	0.045
25	7.20	0.049	20.25	0.042
26	7.17	0.051	20.26	0.040
27	7.13	0.051	20.23	0.042
28	7.03	0.047	20.15	0.043
29	6.98	0.049	20.06	0.045
30	6.95	0.051	19.96	0.047
31	6.91	0.052	19.96	0.045
32	6.87	0.053	19.69	0.052
33	6.84	0.054	19.92	0.048
34	6.81	0.054	19.86	0.047
35	6.80	0.057	19.97	0.056
36	6.74	0.052	19.97	0.059
37	6.71	0.055	20.01	0.060
38	6.54	0.047	20.02	0.061
39	6.47	0.046	20.04	0.062
40	6.44	0.046	20.10	0.062
41	6.40	0.046	19.83	0.066
42	6.34	0.045	19.82	0.067
43	6.29	0.046	19.79	0.067
44	6.25	0.049	19.70	0.071
45	6.23	0.047	18.83	0.094

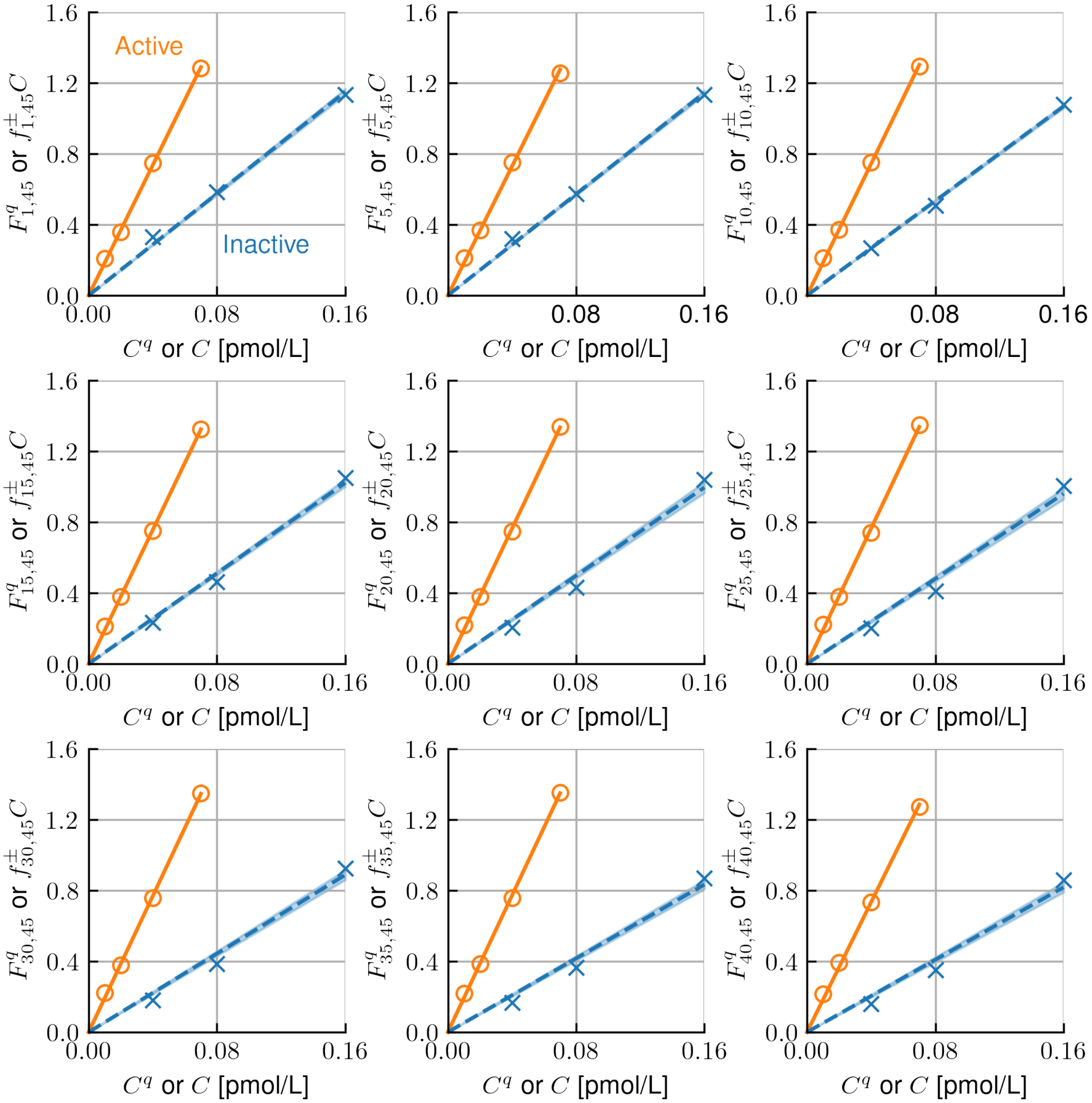

Fig. S45: As Figure S1 with well $w = 45$ (or D9).

Table S45: Molar Fluorescence Parameters for Well D9 ($w = 45$)

Cycle	Inactive		Active	
i	$f^-_{i,45}$	$\sigma^-_{i,45}$	$f^+_{i,45}$	$\sigma^+_{i,45}$
1	7.18	0.033	18.41	0.018
2	7.22	0.044	17.80	0.021
3	7.36	0.036	18.02	0.025
4	7.27	0.031	18.14	0.025
5	7.15	0.026	18.22	0.025
6	7.05	0.020	18.35	0.022
7	6.95	0.016	18.41	0.021
8	6.87	0.015	18.48	0.020
9	6.76	0.017	18.57	0.018
10	6.67	0.019	18.60	0.018
11	6.60	0.024	18.67	0.016
12	6.54	0.029	18.70	0.016
13	6.51	0.034	18.79	0.015
14	6.42	0.039	18.86	0.015
15	6.38	0.043	18.91	0.015
16	6.30	0.044	18.97	0.015
17	6.39	0.058	18.99	0.015
18	6.33	0.060	19.02	0.015
19	6.27	0.062	19.07	0.018
20	6.22	0.063	19.07	0.019
21	6.17	0.061	19.06	0.021
22	6.11	0.061	19.09	0.021
23	6.04	0.059	19.11	0.024
24	5.98	0.060	19.14	0.025
25	6.00	0.064	19.12	0.023
26	5.95	0.069	19.12	0.022
27	5.90	0.067	19.20	0.020
28	5.68	0.059	19.25	0.019
29	5.59	0.058	19.21	0.019
30	5.55	0.057	19.22	0.019
31	5.33	0.046	19.13	0.019
32	5.30	0.047	19.21	0.019
33	5.27	0.049	19.24	0.016
34	5.24	0.052	19.27	0.017
35	5.21	0.052	19.27	0.018
36	5.17	0.057	18.41	0.029
37	5.15	0.059	18.43	0.029
38	5.14	0.055	18.48	0.028
39	5.13	0.056	18.50	0.028
40	5.11	0.058	18.35	0.024
41	5.09	0.059	18.39	0.025
42	5.08	0.061	18.40	0.022
43	5.06	0.062	18.44	0.023
44	5.05	0.063	17.87	0.034
45	5.03	0.063	18.06	0.031

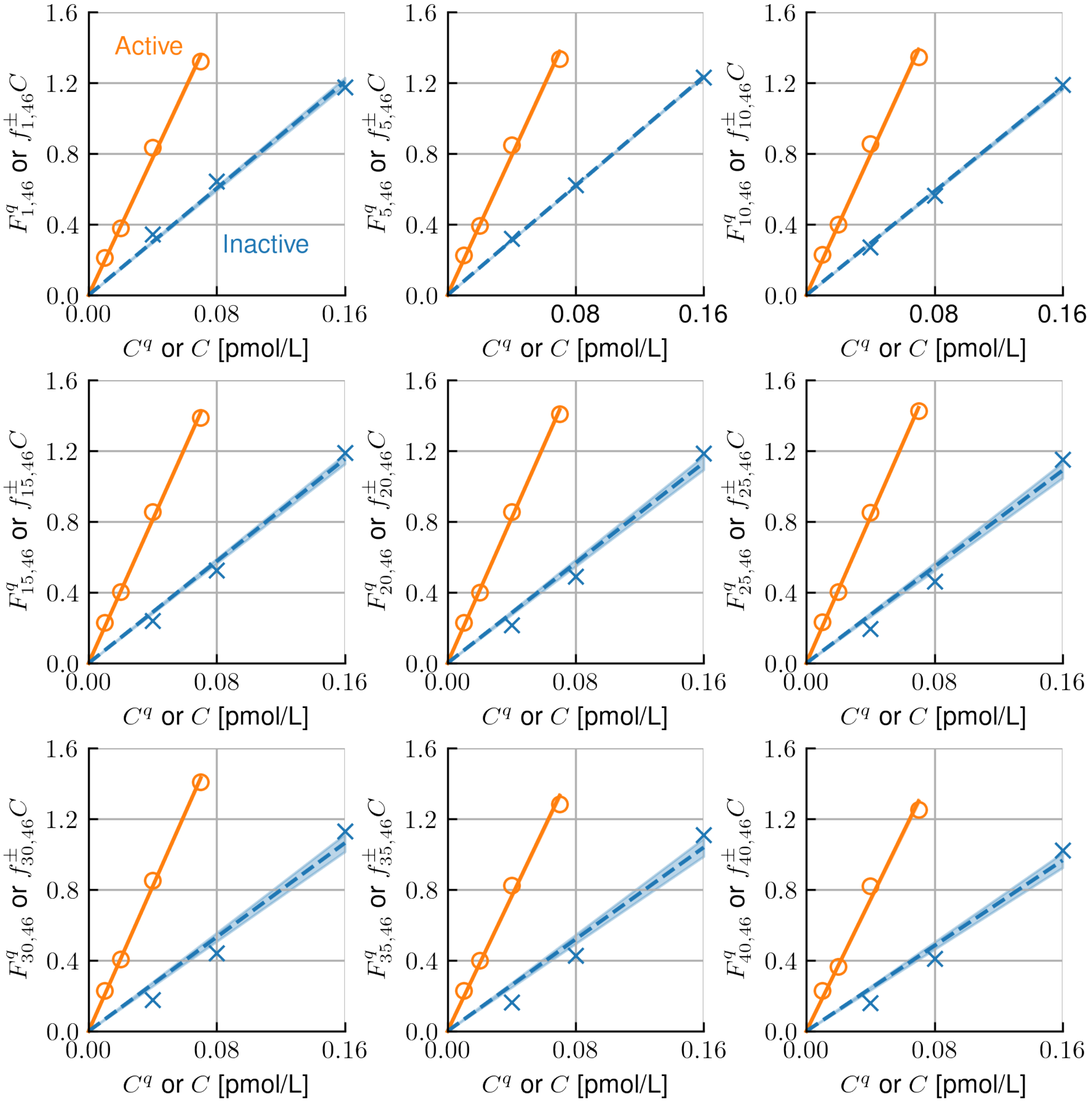

Fig. S46: As Figure S1 with well $w = 46$ (or D10).

Table S46: Molar Fluorescence Parameters for Well D10 ($w = 46$)

Cycle	Inactive		Active	
i	$f^-_{i,46}$	$\sigma^-_{i,46}$	$f^+_{i,46}$	$\sigma^+_{i,46}$
1	7.54	0.046	19.37	0.041
2	7.67	0.037	19.14	0.044
3	7.83	0.023	19.46	0.046
4	7.79	0.018	19.60	0.047
5	7.725	0.0095	19.66	0.047
6	7.628	0.0031	19.62	0.050
7	7.563	0.0078	19.67	0.049
8	7.49	0.014	19.75	0.048
9	7.41	0.020	19.81	0.048
10	7.33	0.025	19.83	0.048
11	7.25	0.028	19.86	0.047
12	7.20	0.032	19.93	0.046
13	7.13	0.036	19.77	0.050
14	7.07	0.040	19.87	0.047
15	7.21	0.056	20.25	0.034
16	7.09	0.056	20.36	0.032
17	7.18	0.069	20.43	0.031
18	7.18	0.076	20.52	0.031
19	7.14	0.078	20.53	0.026
20	7.08	0.081	20.45	0.030
21	6.88	0.071	20.52	0.028
22	6.90	0.078	20.56	0.027
23	6.84	0.080	20.59	0.025
24	6.84	0.090	20.60	0.026
25	6.81	0.092	20.62	0.025
26	6.78	0.092	20.63	0.022
27	6.74	0.096	20.68	0.021
28	6.71	0.098	20.68	0.021
29	6.67	0.099	20.65	0.021
30	6.6	0.10	20.45	0.027
31	6.7	0.10	20.47	0.027
32	6.6	0.10	20.47	0.026
33	6.5	0.10	20.55	0.026
34	6.5	0.11	19.07	0.049
35	6.5	0.11	19.02	0.052
36	6.5	0.11	18.98	0.054
37	6.4	0.11	18.92	0.057
38	6.4	0.11	18.73	0.057
39	6.4	0.11	18.63	0.059
40	6.04	0.087	18.57	0.060
41	6.02	0.088	18.49	0.062
42	5.96	0.088	18.41	0.064
43	5.90	0.087	18.30	0.059
44	5.35	0.054	18.09	0.064
45	5.34	0.055	18.06	0.069

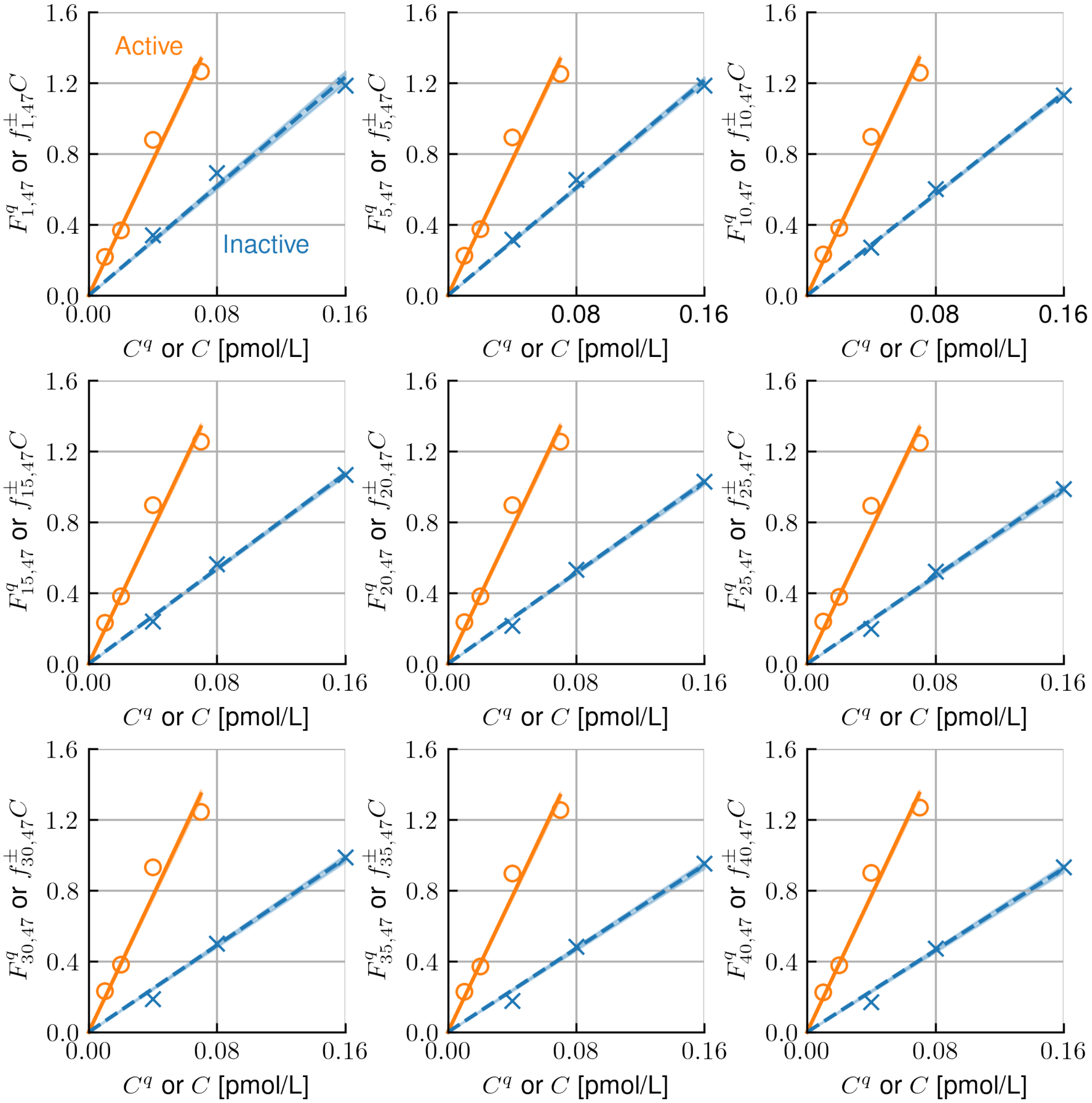

Fig. S47: As Figure S1 with well $w = 47$ (or D11).

Table S47: Molar Fluorescence Parameters for Well D11 ($w = 47$)

Cycle	Inactive		Active	
i	$f^-_{i,47}$	$\sigma^-_{i,47}$	$f^+_{i,47}$	$\sigma^+_{i,47}$
1	7.70	0.067	19.05	0.082
2	7.63	0.066	18.68	0.083
3	7.71	0.056	18.85	0.087
4	7.67	0.047	18.97	0.089
5	7.59	0.039	19.03	0.091
6	7.50	0.032	19.08	0.090
7	7.42	0.028	19.07	0.092
8	7.30	0.028	19.10	0.093
9	7.21	0.026	19.11	0.093
10	7.14	0.025	19.14	0.093
11	7.04	0.025	19.19	0.093
12	6.91	0.028	19.13	0.091
13	6.83	0.027	19.13	0.093
14	6.77	0.028	19.11	0.095
15	6.70	0.028	19.10	0.094
16	6.61	0.025	19.11	0.095
17	6.56	0.026	19.17	0.092
18	6.50	0.028	19.22	0.093
19	6.45	0.029	19.16	0.093
20	6.42	0.032	19.12	0.094
21	6.39	0.036	19.10	0.094
22	6.35	0.037	19.07	0.095
23	6.30	0.039	19.08	0.096
24	6.24	0.041	19.07	0.098
25	6.18	0.041	19.02	0.095
26	6.14	0.042	19.16	0.098
27	6.12	0.040	19.2	0.11
28	6.06	0.039	19.2	0.11
29	6.07	0.036	19.2	0.12
30	6.13	0.041	19.2	0.11
31	6.13	0.044	19.2	0.11
32	6.07	0.043	19.2	0.12
33	6.04	0.042	18.95	0.096
34	5.96	0.043	19.03	0.097
35	5.90	0.042	19.07	0.093
36	5.80	0.041	19.10	0.093
37	5.76	0.041	19.14	0.092
38	5.79	0.042	19.18	0.091
39	5.82	0.044	19.17	0.092
40	5.77	0.043	19.25	0.089
41	5.68	0.042	18.95	0.098
42	5.59	0.040	18.8	0.10
43	5.54	0.040	18.7	0.10
44	5.56	0.042	18.8	0.10
45	5.56	0.045	18.8	0.10

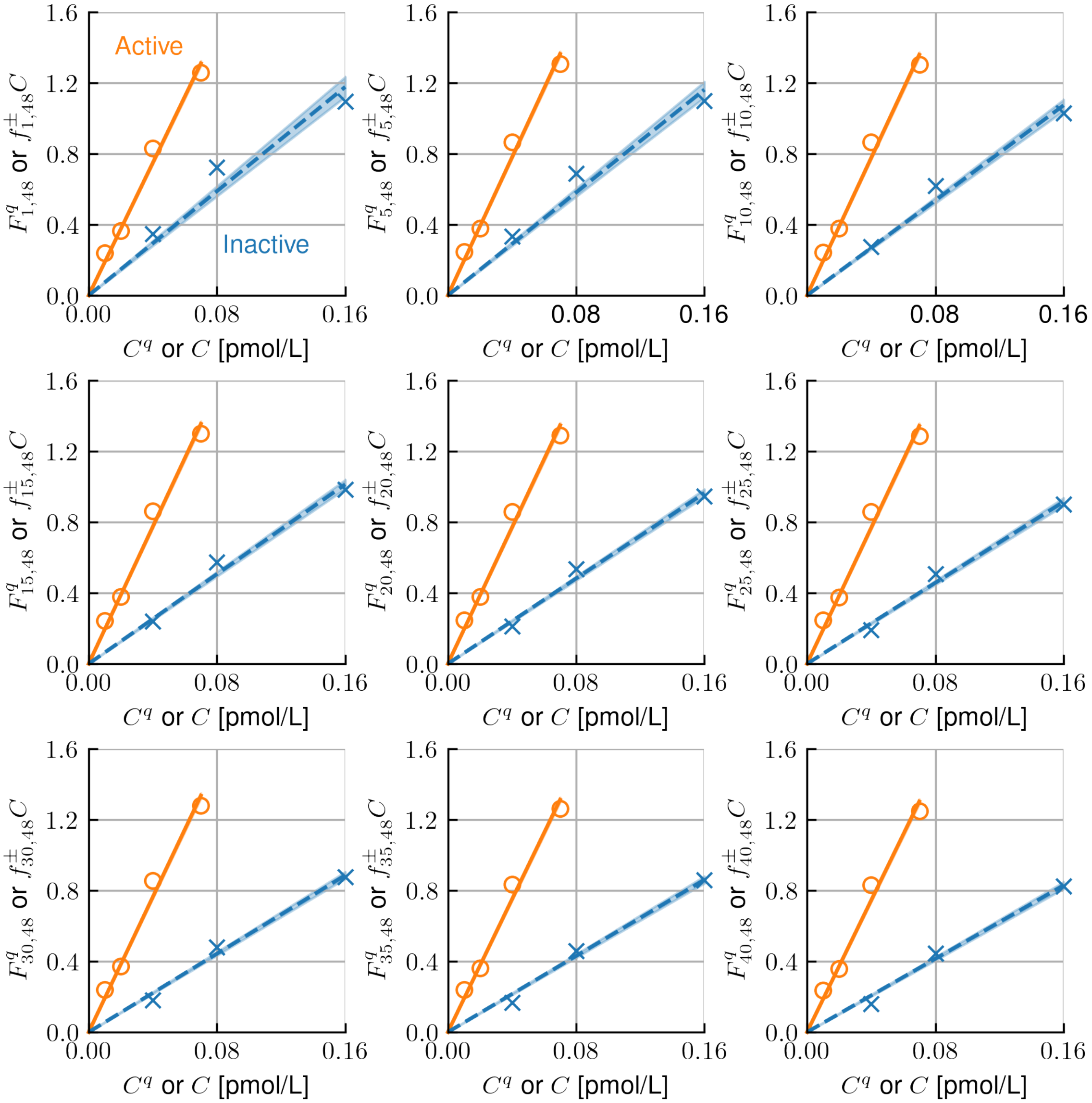

Fig. S48: As Figure S1 with well $w = 48$ (or D12).

Table S48: Molar Fluorescence Parameters for Well D12 ($w = 48$)

Cycle	Inactive		Active	
i	$f^{-}_{i,48}$	$\sigma^{-}_{i,48}$	$f^{+}_{i,48}$	$\sigma^{+}_{i,48}$
1	7.4	0.12	18.72	0.064
2	7.4	0.12	19.11	0.065
3	7.5	0.11	19.35	0.066
4	7.4	0.10	19.45	0.066
5	7.26	0.093	19.48	0.067
6	7.12	0.086	19.48	0.067
7	7.01	0.080	19.46	0.067
8	6.90	0.074	19.45	0.068
9	6.80	0.068	19.44	0.068
10	6.71	0.066	19.41	0.068
11	6.65	0.062	19.43	0.070
12	6.55	0.061	19.41	0.069
13	6.48	0.057	19.37	0.068
14	6.37	0.058	19.41	0.070
15	6.33	0.053	19.36	0.067
16	6.27	0.054	19.32	0.068
17	6.19	0.052	19.28	0.068
18	6.14	0.049	19.30	0.070
19	6.08	0.048	19.26	0.069
20	6.02	0.044	19.24	0.069
21	5.95	0.044	19.27	0.067
22	5.88	0.046	19.22	0.069
23	5.82	0.045	19.29	0.067
24	5.78	0.046	19.26	0.067
25	5.71	0.045	19.19	0.070
26	5.70	0.044	19.22	0.068
27	5.68	0.039	19.22	0.066
28	5.64	0.040	19.22	0.065
29	5.59	0.040	19.14	0.072
30	5.54	0.039	19.08	0.069
31	5.50	0.041	19.06	0.069
32	5.47	0.045	18.89	0.073
33	5.41	0.041	18.93	0.071
34	5.42	0.042	18.90	0.070
35	5.38	0.040	18.75	0.064
36	5.29	0.040	18.71	0.067
37	5.30	0.042	18.71	0.068
38	5.26	0.041	18.74	0.064
39	5.22	0.040	18.66	0.066
40	5.18	0.040	18.60	0.066
41	5.14	0.040	18.56	0.068
42	5.12	0.041	18.49	0.069
43	5.10	0.039	18.52	0.069
44	5.07	0.042	18.43	0.069
45	5.04	0.040	18.47	0.069

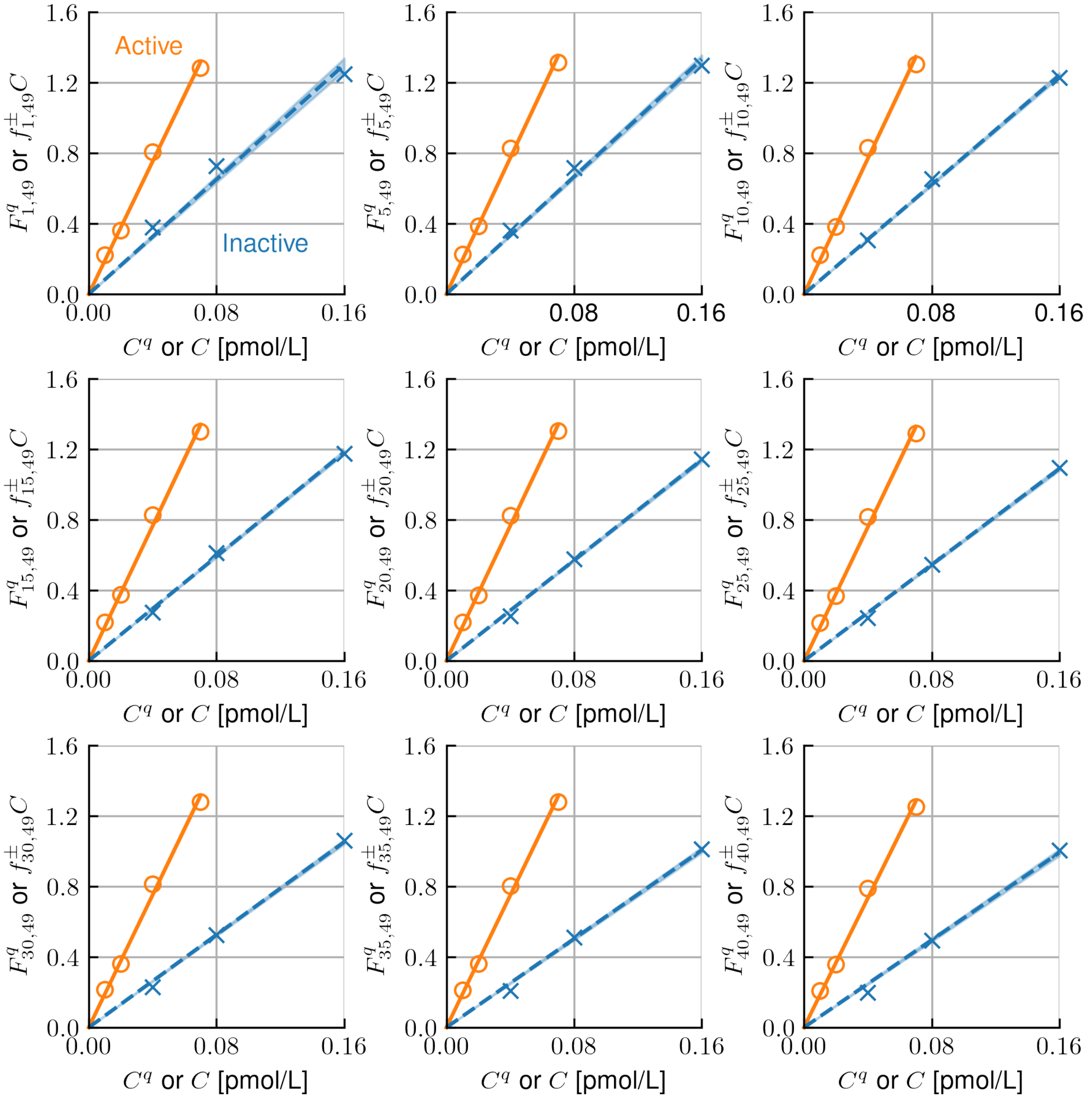

Fig. S49: As Figure S1 with well $w = 49$ (or E1).

Table S49: Molar Fluorescence Parameters for Well E1 ($w = 49$)

Cycle	Inactive		Active	
i	$f^-_{i,49}$	$\sigma^-_{i,49}$	$f^+_{i,49}$	$\sigma^+_{i,49}$
1	8.13	0.074	18.81	0.042
2	8.56	0.066	19.02	0.039
3	8.58	0.061	19.18	0.043
4	8.47	0.054	19.29	0.043
5	8.32	0.047	19.30	0.043
6	8.19	0.041	19.31	0.044
7	8.06	0.036	19.27	0.045
8	7.95	0.032	19.28	0.045
9	7.84	0.028	19.25	0.045
10	7.76	0.026	19.22	0.046
11	7.70	0.023	19.21	0.045
12	7.59	0.022	19.26	0.043
13	7.51	0.025	19.20	0.045
14	7.42	0.024	19.14	0.046
15	7.39	0.021	19.12	0.046
16	7.33	0.019	19.13	0.047
17	7.26	0.019	19.15	0.043
18	7.20	0.022	19.26	0.041
19	7.19	0.022	19.15	0.043
20	7.12	0.023	19.14	0.043
21	7.09	0.022	19.36	0.038
22	7.00	0.021	19.10	0.043
23	6.92	0.020	19.11	0.049
24	6.86	0.020	19.00	0.043
25	6.81	0.021	18.95	0.043
26	6.71	0.021	18.88	0.043
27	6.67	0.022	18.80	0.044
28	6.65	0.026	18.82	0.043
29	6.61	0.025	18.74	0.044
30	6.57	0.025	18.79	0.044
31	6.56	0.026	18.77	0.042
32	6.55	0.029	18.70	0.044
33	6.52	0.033	18.71	0.041
34	6.50	0.035	18.76	0.039
35	6.29	0.030	18.73	0.040
36	6.34	0.034	18.73	0.038
37	6.32	0.035	18.72	0.038
38	6.30	0.037	18.71	0.038
39	6.24	0.037	18.72	0.033
40	6.20	0.037	18.33	0.040
41	6.18	0.038	18.34	0.042
42	6.12	0.039	18.34	0.042
43	6.04	0.038	18.31	0.040
44	6.02	0.040	18.30	0.039
45	6.01	0.042	18.30	0.039

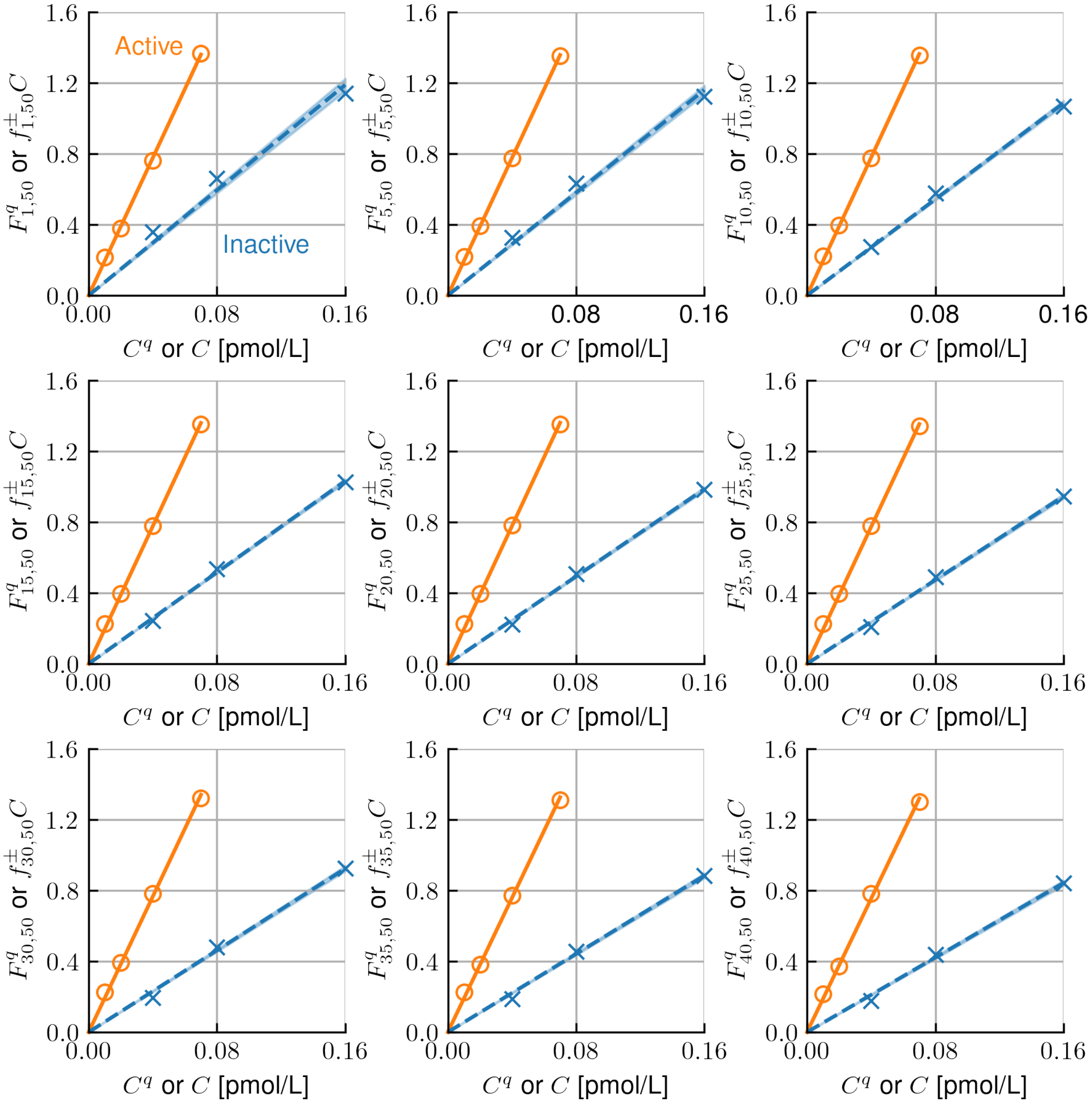

Fig. S50: As Figure S1 with well $w = 50$ (or E2).

Table S50: Molar Fluorescence Parameters for Well E2 ($w = 50$)

Cycle	Inactive		Active	
i	$f^-_{i,50}$	$\sigma^-_{i,50}$	$f^+_{i,50}$	$\sigma^+_{i,50}$
1	7.43	0.071	19.42	0.017
2	7.49	0.065	19.16	0.014
3	7.53	0.062	19.25	0.014
4	7.38	0.057	19.32	0.015
5	7.25	0.052	19.39	0.016
6	7.14	0.046	19.39	0.017
7	7.06	0.040	19.42	0.017
8	6.95	0.034	19.43	0.017
9	6.86	0.030	19.49	0.017
10	6.79	0.027	19.47	0.017
11	6.68	0.027	19.44	0.017
12	6.62	0.024	19.45	0.018
13	6.56	0.023	19.44	0.019
14	6.53	0.023	19.44	0.018
15	6.45	0.019	19.42	0.019
16	6.39	0.020	19.51	0.018
17	6.28	0.020	19.54	0.018
18	6.23	0.020	19.46	0.019
19	6.21	0.019	19.43	0.019
20	6.17	0.020	19.44	0.019
21	6.11	0.021	19.38	0.020
22	6.10	0.023	19.39	0.020
23	6.05	0.020	19.44	0.019
24	6.00	0.020	19.50	0.019
25	5.91	0.023	19.33	0.021
26	5.93	0.025	19.28	0.022
27	5.92	0.027	19.29	0.023
28	5.93	0.029	19.18	0.024
29	5.83	0.028	19.12	0.026
30	5.77	0.028	19.15	0.026
31	5.67	0.027	19.15	0.029
32	5.63	0.028	19.13	0.027
33	5.61	0.027	19.11	0.028
34	5.58	0.027	19.05	0.028
35	5.52	0.025	18.95	0.025
36	5.50	0.026	18.92	0.023
37	5.37	0.026	18.89	0.018
38	5.34	0.026	18.92	0.021
39	5.30	0.026	18.83	0.025
40	5.27	0.027	18.85	0.027
41	5.24	0.026	18.87	0.028
42	5.22	0.027	18.83	0.032
43	5.19	0.027	18.75	0.034
44	5.16	0.027	18.73	0.037
45	5.13	0.028	18.75	0.034

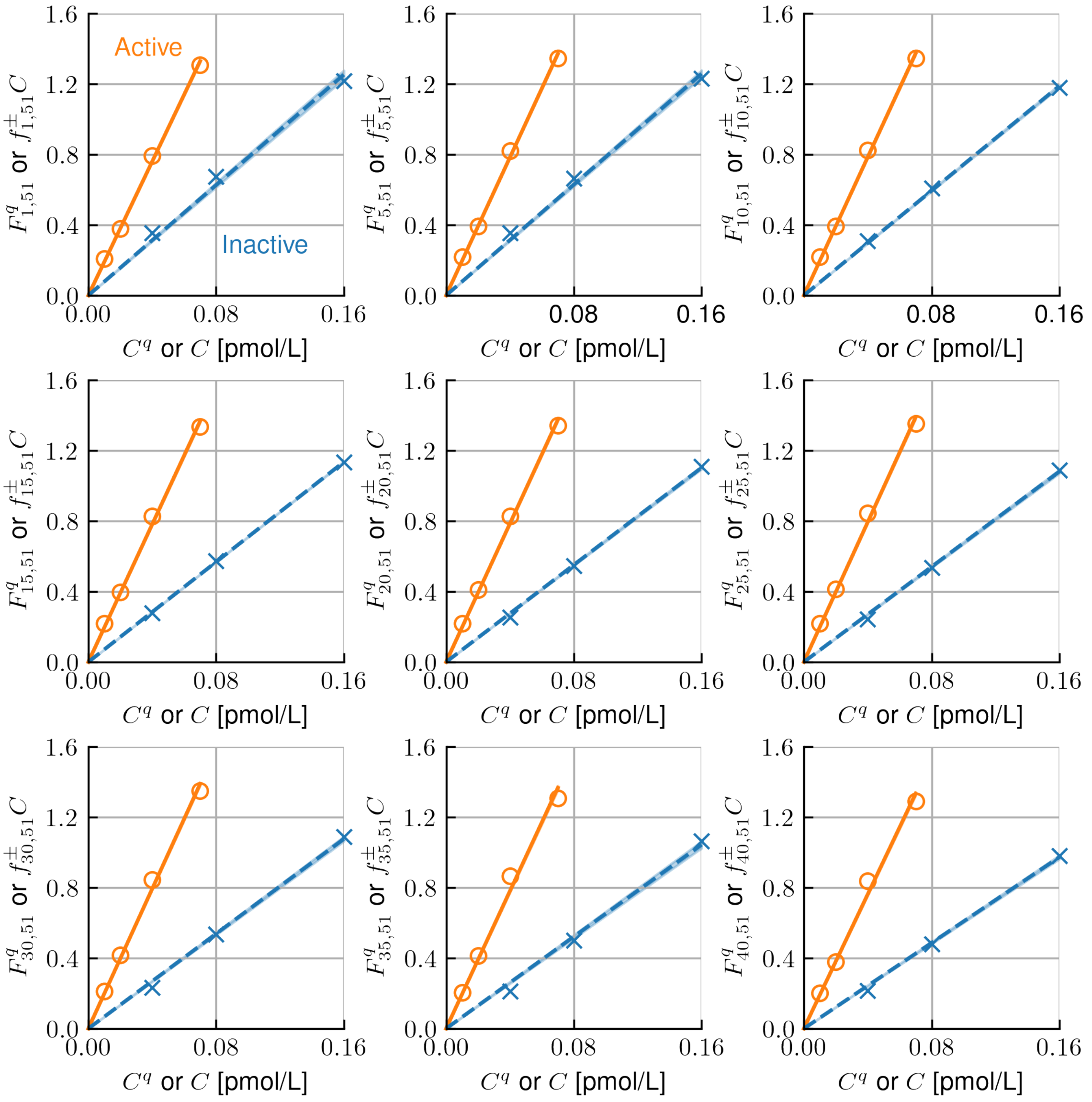

Fig. S51: As Figure S1 with well $w = 51$ (or E3).

Table S51: Molar Fluorescence Parameters for Well E3 ($w = 51$)

Cycle	Inactive		Active	
i	$f^-_{i,51}$	$\sigma^-_{i,51}$	$f^+_{i,51}$	$\sigma^+_{i,51}$
1	7.83	0.051	18.99	0.026
2	7.83	0.053	19.12	0.027
3	7.93	0.050	19.33	0.028
4	7.92	0.047	19.49	0.029
5	7.86	0.041	19.59	0.029
6	7.77	0.035	19.63	0.030
7	7.68	0.029	19.62	0.031
8	7.60	0.024	19.62	0.030
9	7.50	0.020	19.62	0.030
10	7.43	0.015	19.61	0.031
11	7.35	0.011	19.58	0.031
12	7.269	0.0089	19.56	0.032
13	7.205	0.0068	19.54	0.033
14	7.138	0.0058	19.53	0.034
15	7.096	0.0056	19.52	0.036
16	7.106	0.0092	19.43	0.040
17	7.03	0.010	19.41	0.040
18	7.03	0.014	19.47	0.038
19	6.96	0.016	19.64	0.035
20	6.89	0.016	19.65	0.035
21	6.86	0.019	19.67	0.035
22	6.79	0.019	19.78	0.035
23	6.78	0.018	19.85	0.039
24	6.78	0.020	19.84	0.040
25	6.76	0.021	19.84	0.039
26	6.73	0.022	19.79	0.040
27	6.76	0.026	19.87	0.041
28	6.72	0.028	19.83	0.040
29	6.71	0.029	19.77	0.043
30	6.73	0.027	19.83	0.040
31	6.65	0.031	19.76	0.044
32	6.64	0.034	19.72	0.044
33	6.63	0.032	19.69	0.045
34	6.55	0.038	19.63	0.046
35	6.52	0.040	19.52	0.061
36	6.49	0.040	19.45	0.055
37	6.51	0.042	19.27	0.057
38	6.50	0.045	19.11	0.051
39	6.47	0.047	19.12	0.052
40	6.08	0.022	19.05	0.051
41	6.03	0.019	19.04	0.051
42	6.00	0.019	18.99	0.053
43	6.01	0.020	18.92	0.049
44	6.00	0.019	18.87	0.050
45	5.93	0.043	18.72	0.040

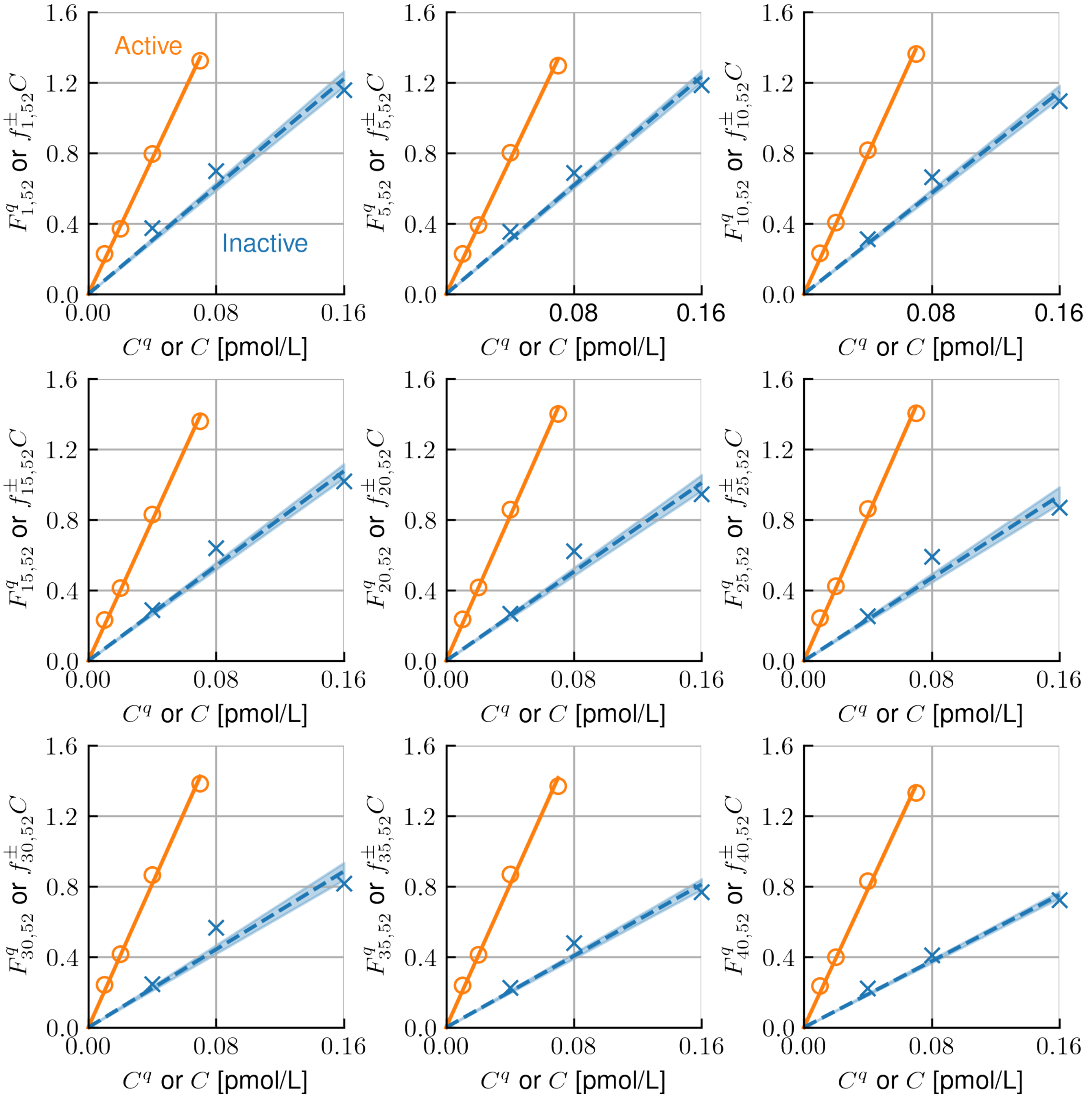

Fig. S52: As Figure S1 with well $w = 52$ (or E4).

Table S52: Molar Fluorescence Parameters for Well E4 ($w = 52$)

Cycle	Inactive		Active	
i	$f^-_{i,52}$	$\sigma^-_{i,52}$	$f^+_{i,52}$	$\sigma^+_{i,52}$
1	7.62	0.092	19.19	0.031
2	7.75	0.075	18.69	0.032
3	7.87	0.075	18.89	0.036
4	7.82	0.073	18.97	0.038
5	7.71	0.068	19.02	0.040
6	7.59	0.064	19.04	0.042
7	7.47	0.061	19.10	0.042
8	7.37	0.067	19.31	0.037
9	7.27	0.073	19.70	0.028
10	7.16	0.076	19.81	0.029
11	7.06	0.073	19.78	0.031
12	6.97	0.075	19.88	0.030
13	6.89	0.079	19.94	0.030
14	6.79	0.083	19.79	0.036
15	6.72	0.084	19.87	0.034
16	6.63	0.086	19.97	0.034
17	6.57	0.092	20.09	0.032
18	6.49	0.094	20.16	0.032
19	6.39	0.089	20.22	0.031
20	6.31	0.094	20.46	0.035
21	6.19	0.100	20.50	0.032
22	6.1	0.10	20.52	0.032
23	6.02	0.098	20.61	0.034
24	5.92	0.094	20.54	0.038
25	5.9	0.10	20.55	0.038
26	5.8	0.10	20.22	0.049
27	5.7	0.10	20.28	0.047
28	5.6	0.10	20.31	0.046
29	5.6	0.10	20.36	0.046
30	5.5	0.10	20.34	0.045
31	5.46	0.099	20.21	0.049
32	5.32	0.080	20.18	0.045
33	5.04	0.028	20.22	0.047
34	5.14	0.061	20.26	0.047
35	5.07	0.063	20.21	0.049
36	5.02	0.061	19.85	0.042
37	4.94	0.060	19.70	0.041
38	4.87	0.054	19.75	0.041
39	4.77	0.046	19.60	0.042
40	4.70	0.040	19.55	0.043
41	4.62	0.038	17.6	0.10
42	4.44	0.043	17.77	0.095
43	4.42	0.042	17.63	0.080
44	4.35	0.044	17.71	0.087
45	4.30	0.046	17.68	0.088

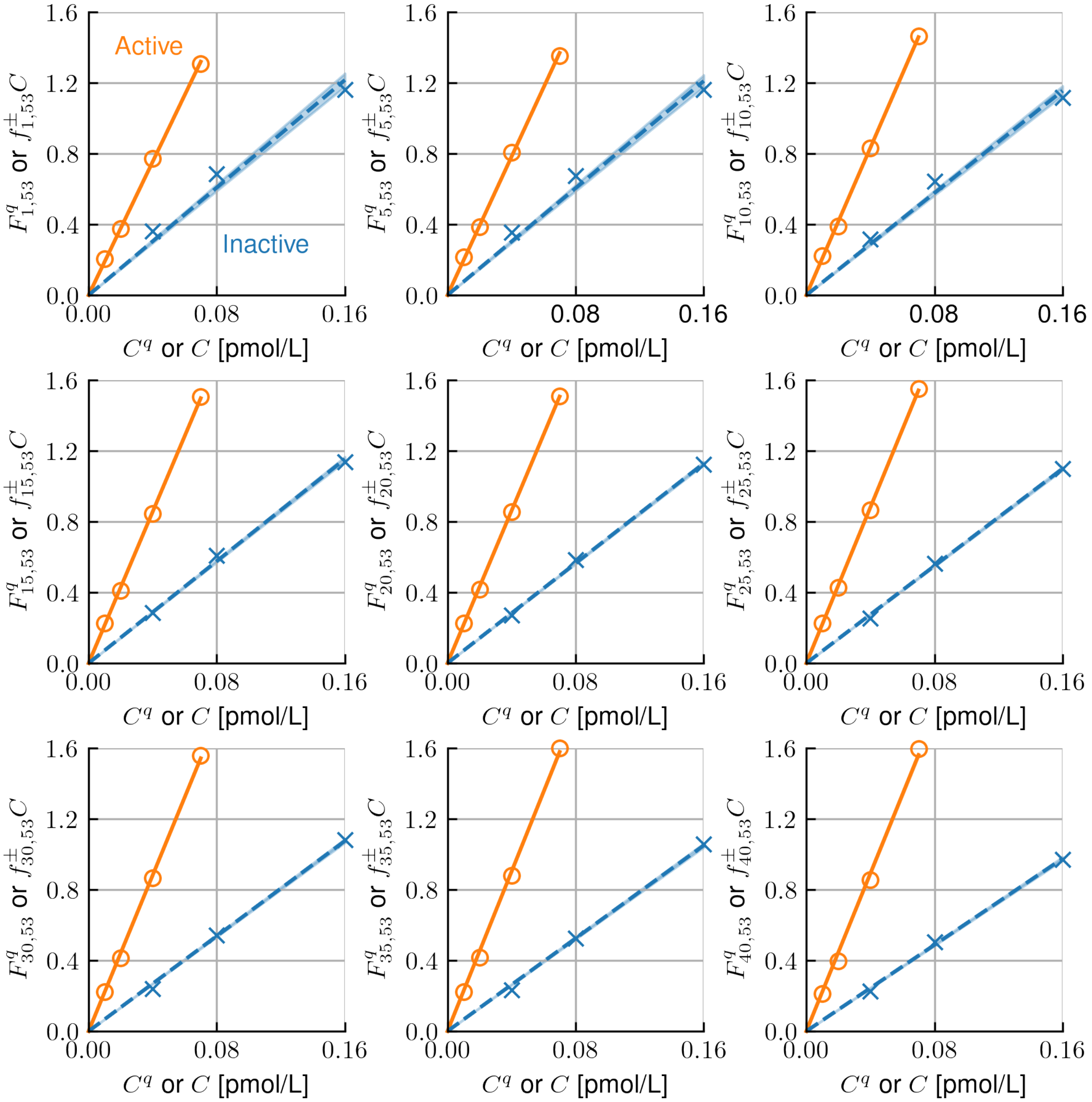

Fig. S53: As Figure S1 with well $w = 53$ (or E5).

Table S53: Molar Fluorescence Parameters for Well E5 ($w = 53$)

Cycle	Inactive		Active	
i	$f^-_{i,53}$	$\sigma^-_{i,53}$	$f^+_{i,53}$	$\sigma^+_{i,53}$
1	7.60	0.078	18.85	0.017
2	7.53	0.081	18.82	0.016
3	7.70	0.077	19.21	0.018
4	7.67	0.074	19.39	0.019
5	7.56	0.070	19.56	0.020
6	7.51	0.067	19.79	0.024
7	7.44	0.063	19.88	0.022
8	7.37	0.059	20.51	0.015
9	7.30	0.057	20.68	0.016
10	7.22	0.056	20.83	0.017
11	7.15	0.054	20.99	0.013
12	7.09	0.053	21.10	0.013
13	7.02	0.052	21.27	0.014
14	7.23	0.032	21.34	0.014
15	7.21	0.026	21.39	0.014
16	7.16	0.022	21.47	0.014
17	7.12	0.022	21.54	0.014
18	7.10	0.020	21.67	0.016
19	7.07	0.019	21.54	0.012
20	7.06	0.017	21.524	0.0097
21	7.03	0.016	21.60	0.010
22	7.02	0.017	21.73	0.014
23	6.99	0.016	21.77	0.012
24	6.96	0.016	21.92	0.011
25	6.87	0.017	22.02	0.012
26	6.72	0.020	22.05	0.010
27	6.72	0.019	22.21	0.012
28	6.75	0.018	21.980	0.0078
29	6.73	0.019	22.17	0.015
30	6.72	0.020	22.04	0.019
31	6.70	0.022	22.18	0.020
32	6.65	0.021	22.35	0.026
33	6.64	0.025	22.39	0.025
34	6.60	0.021	22.48	0.026
35	6.56	0.023	22.55	0.027
36	6.55	0.024	22.59	0.027
37	6.19	0.021	22.33	0.033
38	6.16	0.023	22.41	0.033
39	6.12	0.021	22.48	0.034
40	6.10	0.019	22.30	0.041
41	6.02	0.017	21.76	0.035
42	6.00	0.018	21.82	0.035
43	5.97	0.020	21.82	0.056
44	5.94	0.019	21.64	0.064
45	5.83	0.020	21.59	0.063

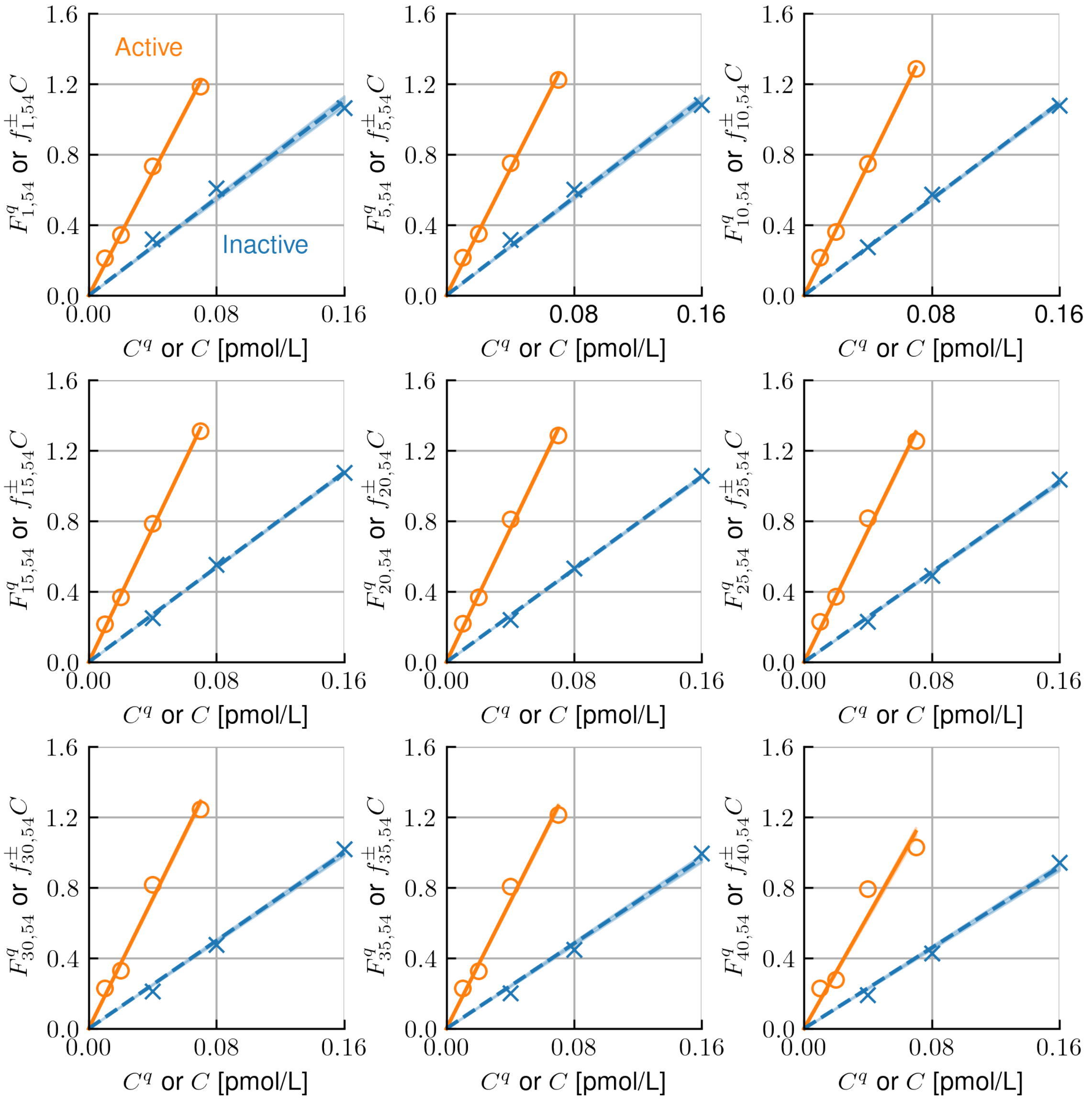

Fig. S54: As Figure S1 with well $w = 54$ (or E6).

Table S54: Molar Fluorescence Parameters for Well E6 ($w = 54$)

Cycle	Inactive		Active	
i	$f^-_{i,54}$	$\sigma^-_{i,54}$	$f^+_{i,54}$	$\sigma^+_{i,54}$
1	6.90	0.057	17.35	0.036
2	6.86	0.054	17.48	0.033
3	7.01	0.054	17.64	0.035
4	7.00	0.053	17.76	0.035
5	6.95	0.047	17.87	0.034
6	6.90	0.038	18.00	0.030
7	6.87	0.037	18.17	0.027
8	6.84	0.034	18.34	0.023
9	6.78	0.032	18.43	0.020
10	6.84	0.023	18.48	0.020
11	6.81	0.020	18.56	0.019
12	6.85	0.014	18.69	0.021
13	6.82	0.014	18.76	0.021
14	6.78	0.015	18.84	0.024
15	6.73	0.016	18.96	0.025
16	6.69	0.017	19.10	0.029
17	6.65	0.017	19.04	0.032
18	6.61	0.013	18.97	0.035
19	6.61	0.015	18.96	0.037
20	6.58	0.016	18.86	0.042
21	6.53	0.017	18.82	0.046
22	6.52	0.017	18.78	0.050
23	6.51	0.019	18.73	0.053
24	6.47	0.020	18.66	0.056
25	6.38	0.026	18.62	0.056
26	6.36	0.026	18.55	0.056
27	6.34	0.029	18.50	0.057
28	6.30	0.030	18.46	0.058
29	6.28	0.033	18.42	0.061
30	6.24	0.033	18.39	0.062
31	6.21	0.035	18.36	0.063
32	6.15	0.037	18.23	0.061
33	6.13	0.040	18.18	0.064
34	6.11	0.042	18.11	0.065
35	6.04	0.042	18.01	0.066
36	5.93	0.040	17.82	0.077
37	5.83	0.038	17.75	0.076
38	5.80	0.039	15.7	0.12
39	5.77	0.039	16.1	0.11
40	5.72	0.039	15.9	0.11
41	5.68	0.038	15.9	0.11
42	5.65	0.041	15.8	0.11
43	5.62	0.043	14.85	0.056
44	5.58	0.045	15.13	0.075
45	5.55	0.047	15.07	0.073

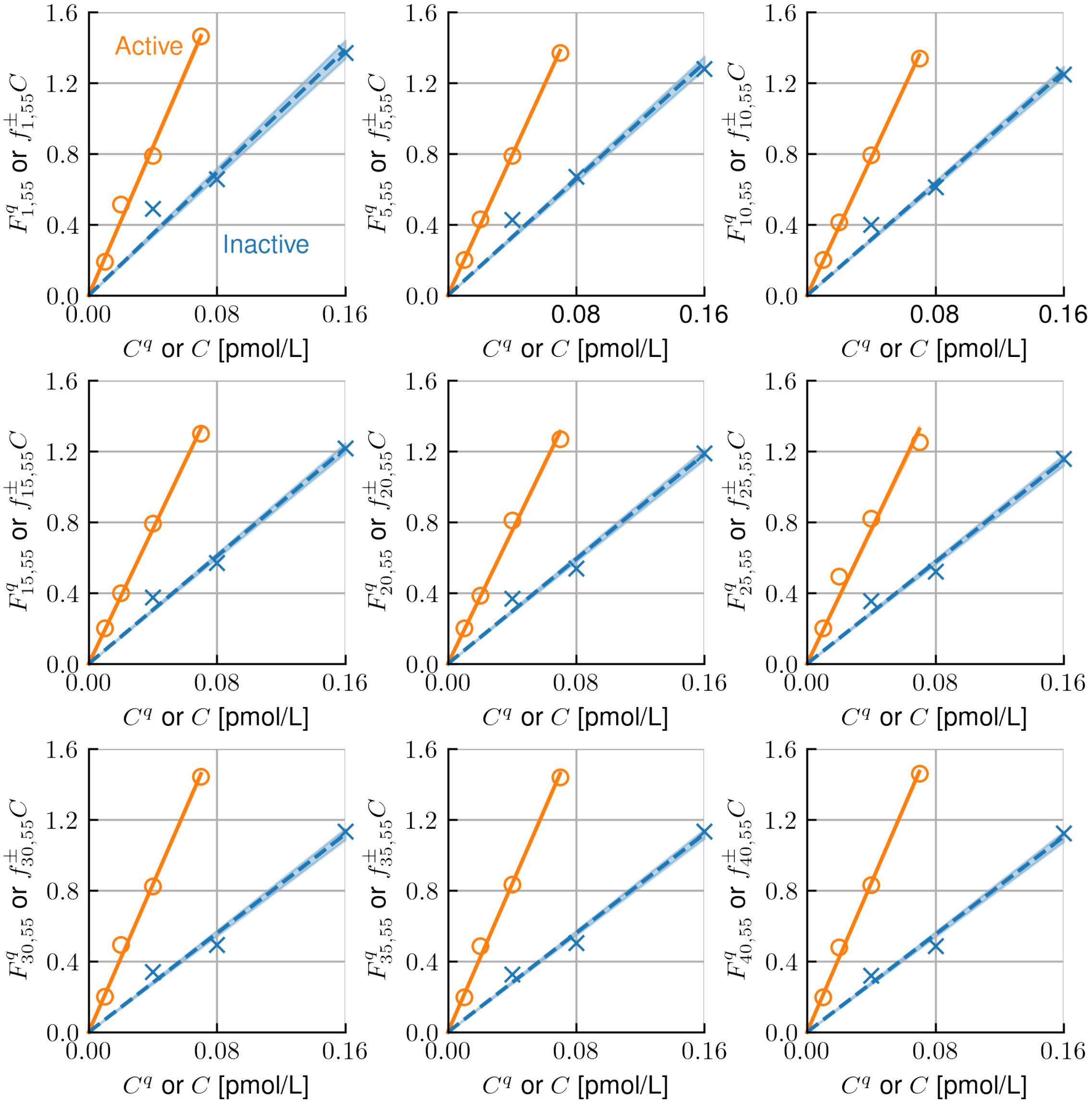

Fig. S55: As Figure S1 with well $w = 55$ (or E7).

Table S55: Molar Fluorescence Parameters for Well E7 ($w = 55$)

Cycle	Inactive		Active	
i	$f^-_{i,55}$	$\sigma^-_{i,55}$	$f^+_{i,55}$	$\sigma^+_{i,55}$
1	8.7	0.11	20.92	0.063
2	8.25	0.087	19.97	0.034
3	8.35	0.083	19.98	0.038
4	8.31	0.081	19.85	0.040
5	8.21	0.075	19.75	0.022
6	8.14	0.069	19.67	0.017
7	8.07	0.072	19.66	0.014
8	7.99	0.067	19.53	0.016
9	7.93	0.064	19.55	0.016
10	7.88	0.062	19.39	0.021
11	7.83	0.061	19.21	0.025
12	7.77	0.059	19.16	0.024
13	7.73	0.059	19.16	0.024
14	7.67	0.058	19.05	0.026
15	7.60	0.057	18.97	0.029
16	7.56	0.057	18.87	0.032
17	7.52	0.056	18.82	0.036
18	7.46	0.056	18.80	0.040
19	7.45	0.062	18.73	0.043
20	7.39	0.063	18.72	0.045
21	7.36	0.062	18.89	0.066
22	7.29	0.062	18.90	0.087
23	7.24	0.068	18.86	0.088
24	7.21	0.066	18.89	0.087
25	7.18	0.061	18.91	0.088
26	7.11	0.064	18.94	0.090
27	7.14	0.065	20.47	0.054
28	7.07	0.067	20.86	0.047
29	7.01	0.064	20.83	0.046
30	6.98	0.064	20.83	0.046
31	7.04	0.068	20.86	0.045
32	7.00	0.068	20.88	0.044
33	7.03	0.056	20.88	0.044
34	7.03	0.051	20.88	0.043
35	6.99	0.052	20.86	0.042
36	6.96	0.053	20.90	0.048
37	6.93	0.055	20.94	0.042
38	6.92	0.057	20.95	0.040
39	6.91	0.055	21.09	0.037
40	6.89	0.058	21.02	0.035
41	6.88	0.059	20.91	0.034
42	6.88	0.066	20.91	0.033
43	6.87	0.063	20.93	0.032
44	6.82	0.062	20.88	0.030
45	6.71	0.057	20.80	0.028

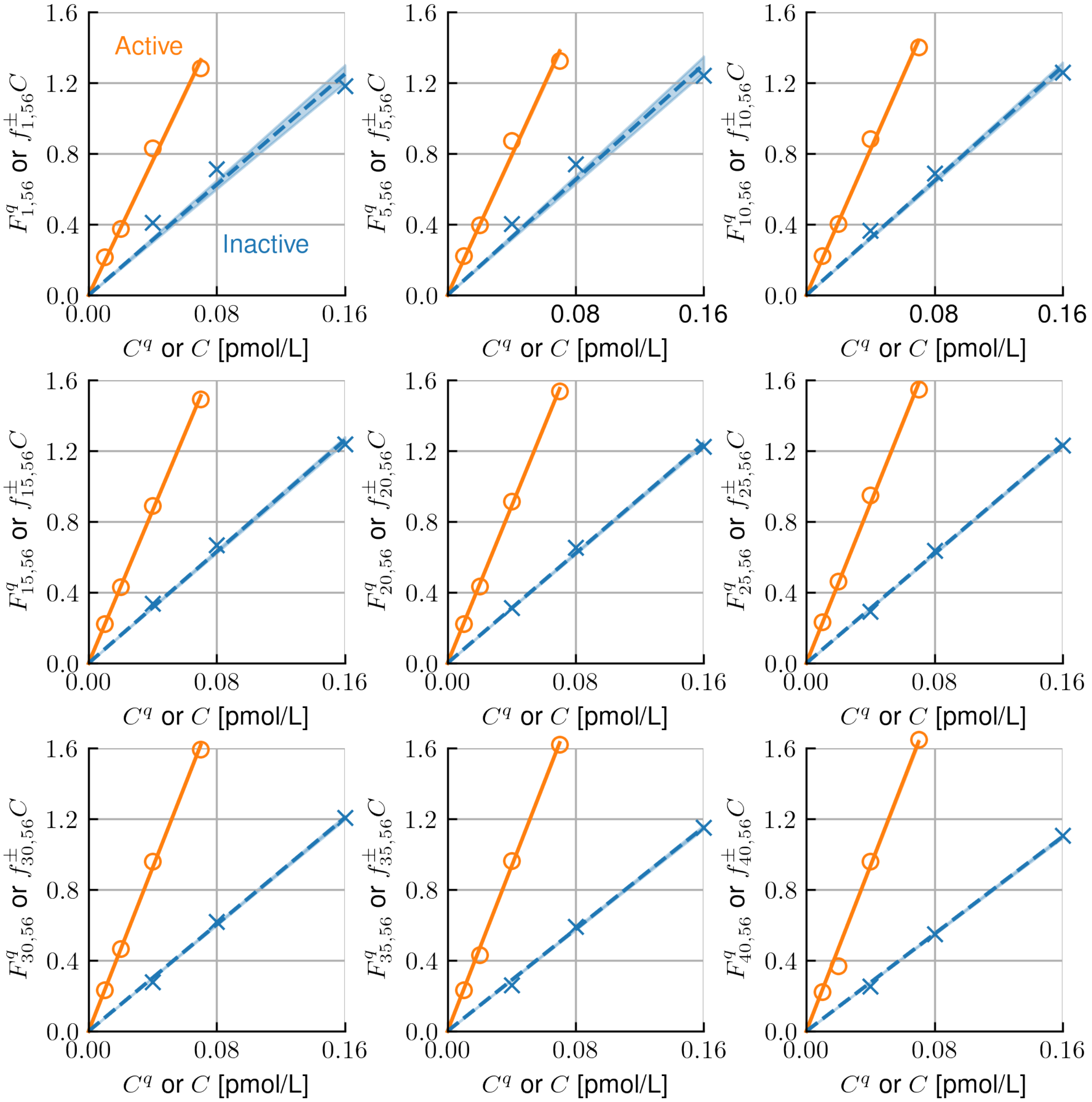

Fig. S56: As Figure S1 with well $w = 56$ (or E8).

Table S56: Molar Fluorescence Parameters for Well E8 ($w = 56$)

Cycle	Inactive		Active	
i	$f^-_{i,56}$	$\sigma^-_{i,56}$	$f^+_{i,56}$	$\sigma^+_{i,56}$
1	7.8	0.10	18.97	0.050
2	7.96	0.095	19.05	0.053
3	8.16	0.097	19.33	0.057
4	8.20	0.097	19.56	0.060
5	8.15	0.095	19.69	0.060
6	8.23	0.079	19.76	0.061
7	8.20	0.071	19.84	0.060
8	8.14	0.063	20.20	0.051
9	8.11	0.056	20.35	0.047
10	8.07	0.049	20.55	0.042
11	8.06	0.051	20.82	0.037
12	7.98	0.050	21.06	0.031
13	7.95	0.046	21.30	0.025
14	7.92	0.041	21.46	0.021
15	7.88	0.035	21.58	0.019
16	7.83	0.031	21.71	0.017
17	7.78	0.030	21.69	0.017
18	7.77	0.023	21.80	0.017
19	7.75	0.022	21.94	0.016
20	7.76	0.025	22.15	0.020
21	7.83	0.025	22.25	0.014
22	7.83	0.024	22.54	0.014
23	7.81	0.025	22.28	0.028
24	7.77	0.024	22.35	0.033
25	7.73	0.017	22.55	0.033
26	7.71	0.016	22.67	0.032
27	7.66	0.016	22.80	0.032
28	7.62	0.018	22.89	0.029
29	7.60	0.018	23.00	0.026
30	7.54	0.020	23.10	0.025
31	7.47	0.022	23.10	0.026
32	7.47	0.022	23.20	0.023
33	7.44	0.024	23.27	0.021
34	7.23	0.021	23.33	0.029
35	7.21	0.021	23.28	0.027
36	6.93	0.028	23.39	0.028
37	6.95	0.025	23.37	0.049
38	6.87	0.015	23.27	0.048
39	6.87	0.016	23.37	0.047
40	6.88	0.016	23.34	0.059
41	6.84	0.015	23.34	0.059
42	6.85	0.020	23.49	0.061
43	6.83	0.024	23.05	0.059
44	6.83	0.022	23.07	0.059
45	6.83	0.023	23.04	0.059

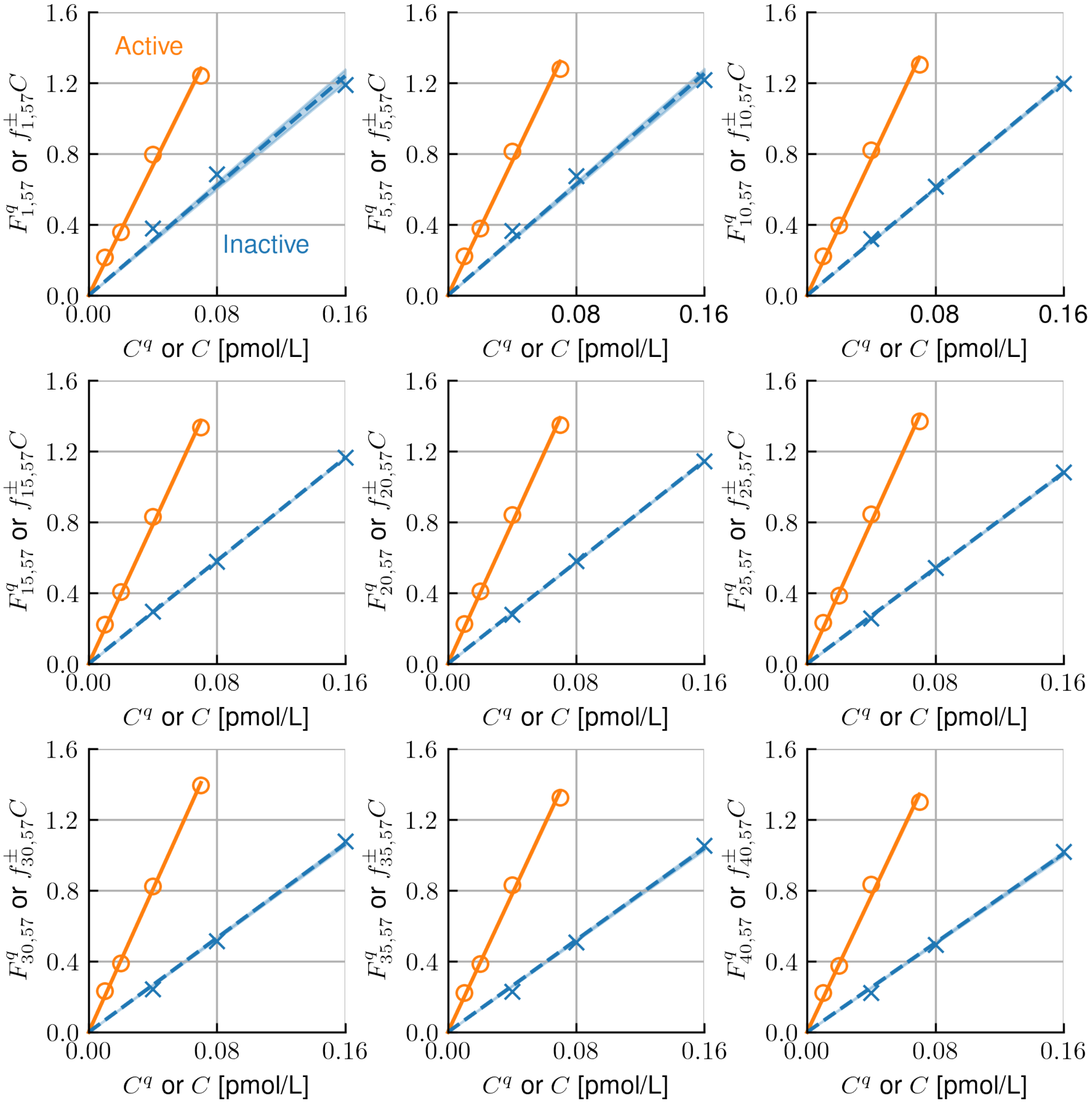

Fig. S57: As Figure S1 with well $w = 57$ (or E9).

Table S57: Molar Fluorescence Parameters for Well E9 ($w = 57$)

Cycle	Inactive		Active	
i	$f^-_{i,57}$	$\sigma^-_{i,57}$	$f^+_{i,57}$	$\sigma^+_{i,57}$
1	7.74	0.077	18.30	0.048
2	7.75	0.075	18.31	0.041
3	7.85	0.073	18.55	0.046
4	7.86	0.066	18.73	0.047
5	7.83	0.055	18.86	0.045
6	7.79	0.045	18.94	0.045
7	7.74	0.036	19.05	0.045
8	7.65	0.030	19.08	0.043
9	7.59	0.023	19.12	0.043
10	7.53	0.016	19.18	0.044
11	7.47	0.012	19.23	0.046
12	7.43	0.012	19.31	0.045
13	7.380	0.0088	19.46	0.041
14	7.318	0.0041	19.52	0.041
15	7.277	0.0051	19.61	0.039
16	7.228	0.0048	19.55	0.045
17	7.190	0.0051	19.61	0.041
18	7.193	0.0043	19.71	0.040
19	7.177	0.0053	19.77	0.041
20	7.168	0.0089	19.82	0.040
21	7.119	0.0083	19.85	0.040
22	7.059	0.0100	19.88	0.037
23	7.06	0.012	19.93	0.038
24	6.98	0.017	19.95	0.036
25	6.748	0.0098	19.98	0.038
26	6.68	0.017	20.05	0.036
27	6.66	0.018	20.16	0.035
28	6.66	0.019	20.31	0.045
29	6.65	0.019	20.08	0.024
30	6.65	0.022	20.11	0.024
31	6.64	0.023	20.16	0.021
32	6.64	0.026	20.08	0.022
33	6.59	0.024	20.16	0.020
34	6.52	0.022	19.37	0.040
35	6.50	0.024	19.43	0.040
36	6.46	0.024	19.45	0.040
37	6.45	0.025	19.50	0.039
38	6.41	0.026	19.07	0.053
39	6.39	0.026	19.15	0.051
40	6.30	0.023	19.17	0.049
41	6.22	0.023	19.20	0.048
42	5.64	0.027	19.27	0.047
43	6.22	0.029	19.34	0.045
44	5.92	0.014	19.36	0.046
45	5.64	0.014	19.38	0.043

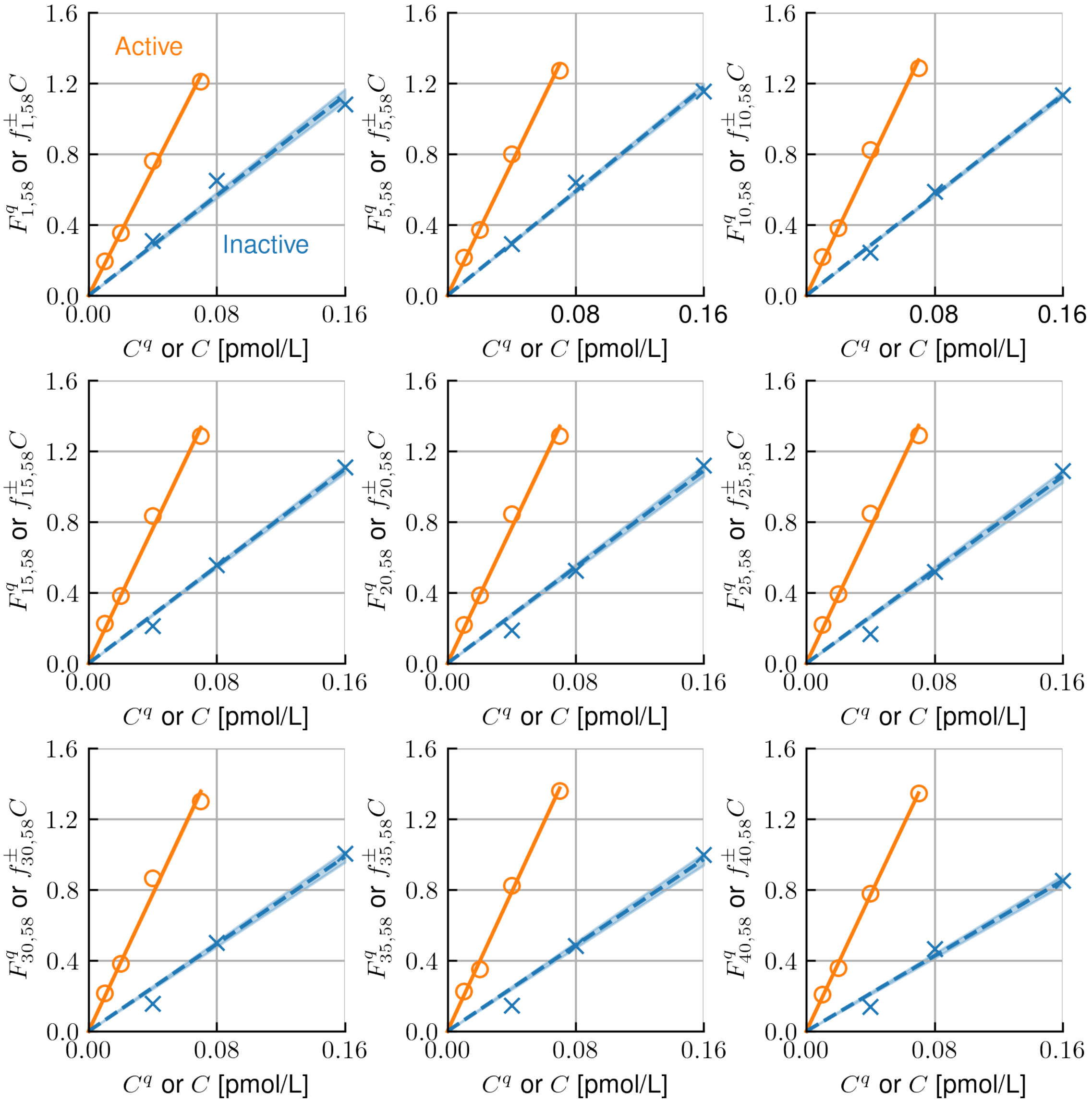

Fig. S58: As Figure S1 with well $w = 58$ (or E10).

Table S58: Molar Fluorescence Parameters for Well E10 ($w = 58$)

Cycle	Inactive		Active	
i	$f^-_{i,58}$	$\sigma^-_{i,58}$	$f^+_{i,58}$	$\sigma^+_{i,58}$
1	7.07	0.073	17.76	0.037
2	7.32	0.062	18.03	0.035
3	7.41	0.054	18.32	0.040
4	7.42	0.048	18.49	0.042
5	7.37	0.040	18.67	0.041
6	7.29	0.035	18.71	0.042
7	7.22	0.030	18.79	0.043
8	7.20	0.027	18.86	0.042
9	7.15	0.028	18.96	0.044
10	7.09	0.032	19.00	0.048
11	7.03	0.034	19.03	0.047
12	6.98	0.037	19.04	0.050
13	6.94	0.040	18.99	0.048
14	6.91	0.043	19.02	0.050
15	6.86	0.046	19.04	0.053
16	6.81	0.049	19.11	0.053
17	6.78	0.053	19.09	0.054
18	6.83	0.057	19.13	0.055
19	6.82	0.061	19.12	0.057
20	6.81	0.065	19.12	0.058
21	6.81	0.070	19.10	0.059
22	6.78	0.073	19.14	0.062
23	6.71	0.071	19.23	0.061
24	6.67	0.072	19.20	0.053
25	6.61	0.073	19.18	0.059
26	6.55	0.074	19.25	0.060
27	6.52	0.074	19.23	0.061
28	6.33	0.067	19.27	0.060
29	6.21	0.064	19.38	0.059
30	6.17	0.066	19.36	0.062
31	6.13	0.068	19.45	0.045
32	6.14	0.068	19.60	0.045
33	6.13	0.070	19.71	0.048
34	6.09	0.069	19.77	0.045
35	6.08	0.070	19.62	0.037
36	5.97	0.067	19.71	0.039
37	5.72	0.061	19.51	0.042
38	5.70	0.064	19.74	0.041
39	5.32	0.060	19.57	0.044
40	5.32	0.058	19.23	0.019
41	5.31	0.058	19.24	0.021
42	5.27	0.055	19.26	0.023
43	5.27	0.055	19.36	0.024
44	5.26	0.055	19.34	0.027
45	5.25	0.057	19.23	0.025

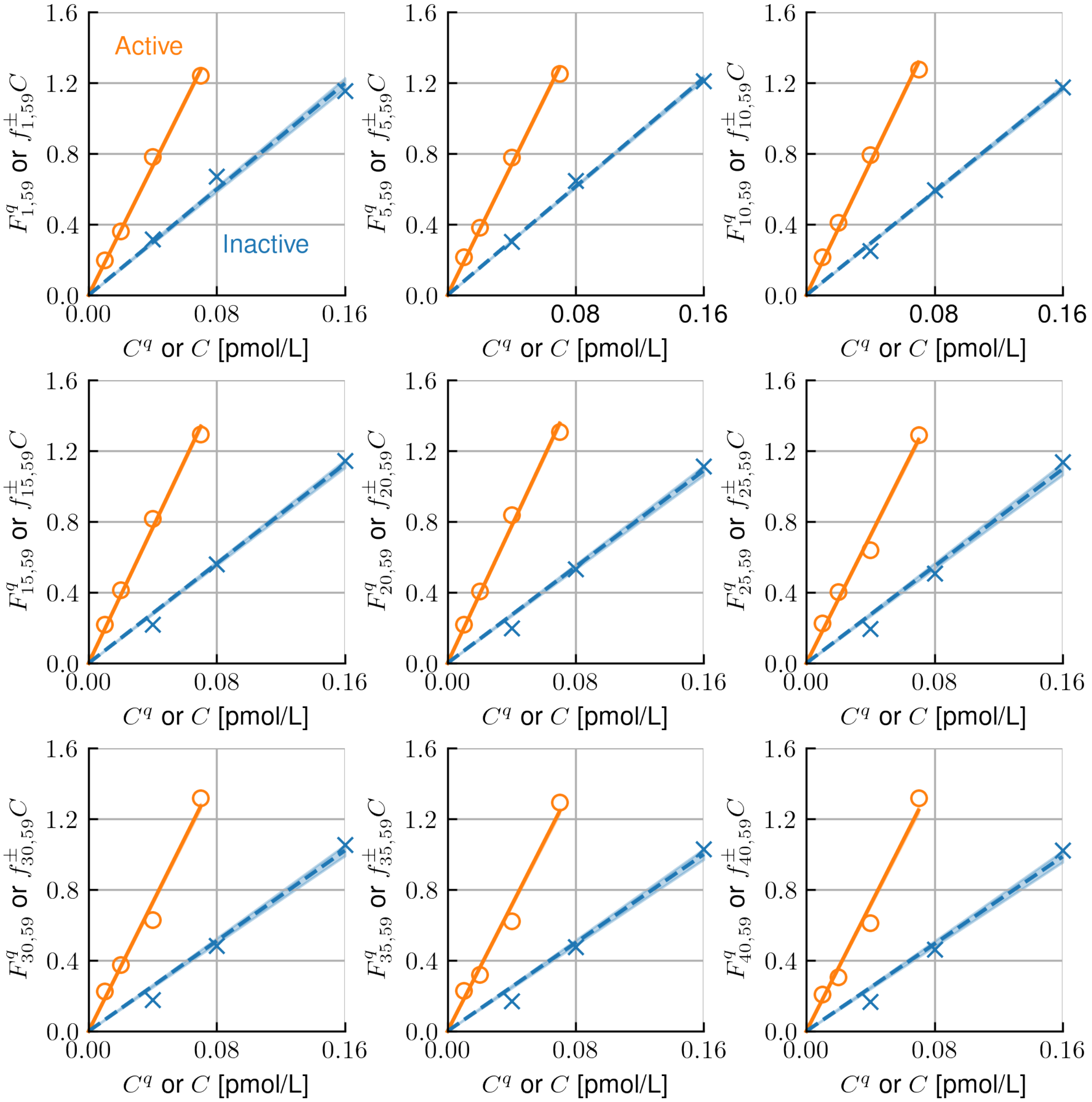

Fig. S59: As Figure S1 with well $w = 59$ (or E11).

Table S59: Molar Fluorescence Parameters for Well E11 ($w = 59$)

Cycle	Inactive		Active	
i	$f^-_{i,59}$	$\sigma^-_{i,59}$	$f^+_{i,59}$	$\sigma^+_{i,59}$
1	7.48	0.062	18.20	0.038
2	7.70	0.040	17.68	0.044
3	7.80	0.036	17.90	0.045
4	7.75	0.032	18.19	0.041
5	7.67	0.026	18.39	0.037
6	7.59	0.023	18.51	0.040
7	7.52	0.022	18.59	0.041
8	7.45	0.024	18.67	0.041
9	7.37	0.027	18.74	0.042
10	7.31	0.030	18.78	0.042
11	7.24	0.033	18.83	0.040
12	7.18	0.036	18.91	0.040
13	7.12	0.039	18.96	0.042
14	7.07	0.042	19.04	0.047
15	7.04	0.045	19.11	0.047
16	6.98	0.048	19.18	0.048
17	6.93	0.050	19.26	0.046
18	6.88	0.052	19.29	0.048
19	6.87	0.054	19.33	0.049
20	6.81	0.056	19.35	0.050
21	6.78	0.057	19.14	0.059
22	6.81	0.058	19.18	0.060
23	6.82	0.062	19.12	0.064
24	6.89	0.071	19.03	0.045
25	6.86	0.069	18.03	0.060
26	6.59	0.057	18.02	0.060
27	6.54	0.060	18.51	0.072
28	6.49	0.062	18.15	0.067
29	6.45	0.063	18.19	0.068
30	6.38	0.063	18.17	0.068
31	6.37	0.059	18.14	0.068
32	6.34	0.061	18.10	0.070
33	6.32	0.060	17.98	0.068
34	6.28	0.061	17.99	0.073
35	6.25	0.062	17.75	0.069
36	6.29	0.066	17.81	0.073
37	6.22	0.064	17.81	0.077
38	6.21	0.066	17.84	0.081
39	6.19	0.066	17.82	0.078
40	6.17	0.066	17.83	0.079
41	6.17	0.068	17.73	0.082
42	6.14	0.067	17.79	0.083
43	6.13	0.070	17.79	0.086
44	6.11	0.072	16.68	0.056
45	5.90	0.062	17.71	0.091

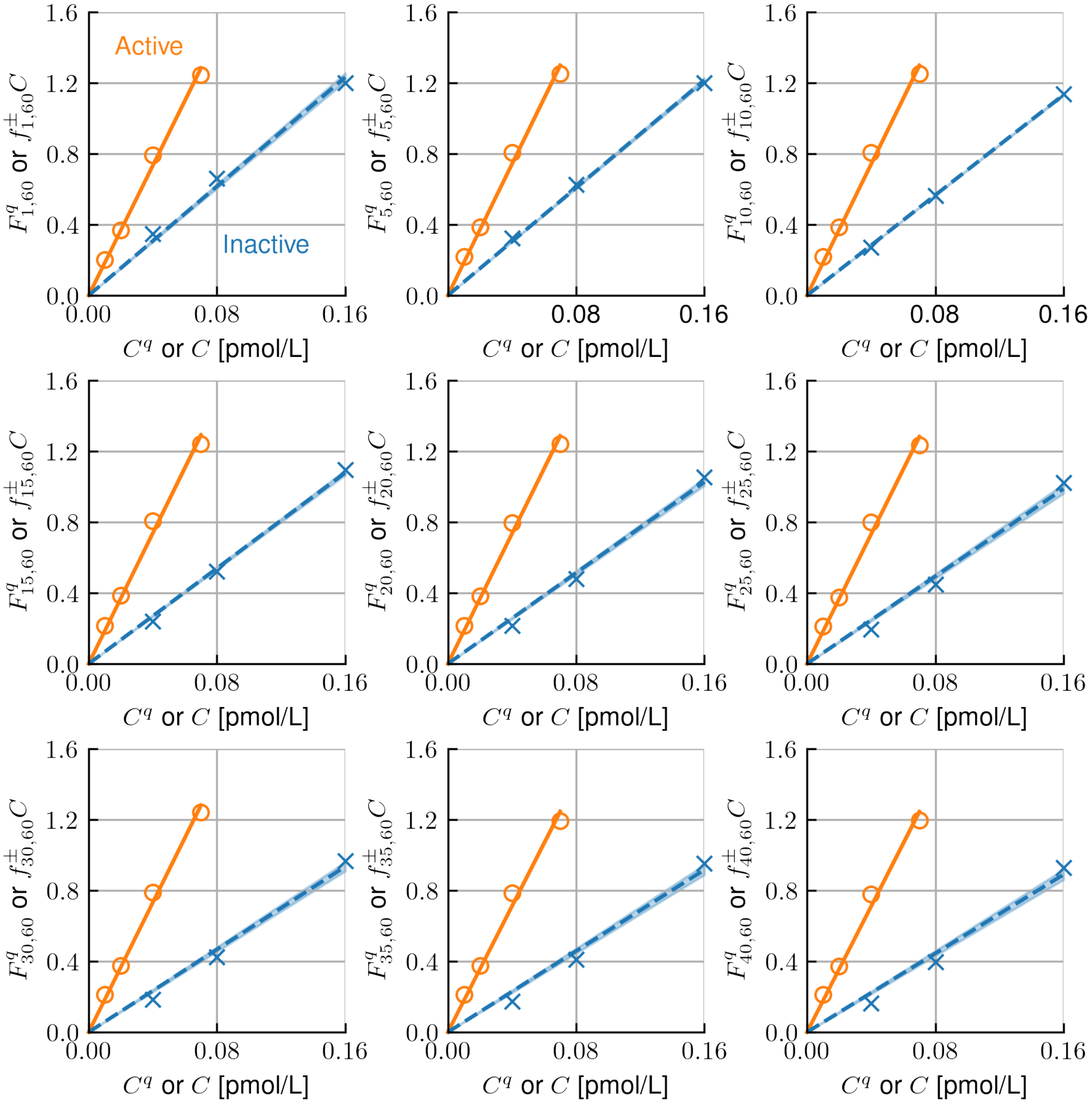

Fig. S60: As Figure S1 with well $w = 60$ (or E12).

Table S60: Molar Fluorescence Parameters for Well E12 ($w = 60$)

Cycle	Inactive		Active	
i	$f^-_{i,60}$	$\sigma^-_{i,60}$	$f^+_{i,60}$	$\sigma^+_{i,60}$
1	7.70	0.049	18.33	0.043
2	7.81	0.045	18.25	0.046
3	7.82	0.038	18.45	0.049
4	7.72	0.030	18.52	0.050
5	7.60	0.022	18.55	0.050
6	7.47	0.014	18.55	0.051
7	7.370	0.0072	18.56	0.050
8	7.282	0.0028	18.55	0.050
9	7.181	0.0057	18.55	0.050
10	7.081	0.0083	18.54	0.051
11	7.01	0.013	18.51	0.050
12	6.94	0.017	18.53	0.049
13	6.91	0.024	18.47	0.050
14	6.81	0.025	18.53	0.051
15	6.74	0.028	18.45	0.053
16	6.67	0.027	18.39	0.050
17	6.62	0.035	18.43	0.048
18	6.53	0.035	18.40	0.051
19	6.48	0.039	18.35	0.050
20	6.42	0.042	18.37	0.049
21	6.37	0.043	18.30	0.049
22	6.32	0.046	18.31	0.049
23	6.32	0.052	18.27	0.049
24	6.26	0.055	18.37	0.050
25	6.17	0.055	18.31	0.051
26	6.09	0.052	18.21	0.049
27	6.04	0.055	18.18	0.048
28	5.98	0.054	18.25	0.046
29	5.88	0.051	18.20	0.047
30	5.84	0.052	18.30	0.043
31	5.82	0.054	18.17	0.045
32	5.78	0.056	18.10	0.049
33	5.76	0.057	18.02	0.051
34	5.76	0.058	17.96	0.051
35	5.73	0.058	17.79	0.057
36	5.70	0.061	17.76	0.053
37	5.66	0.060	17.79	0.053
38	5.63	0.060	17.78	0.052
39	5.58	0.059	17.73	0.052
40	5.56	0.060	17.77	0.053
41	5.53	0.062	17.66	0.049
42	5.53	0.064	17.67	0.049
43	5.39	0.058	17.68	0.049
44	5.39	0.060	17.61	0.045
45	5.32	0.058	17.63	0.045

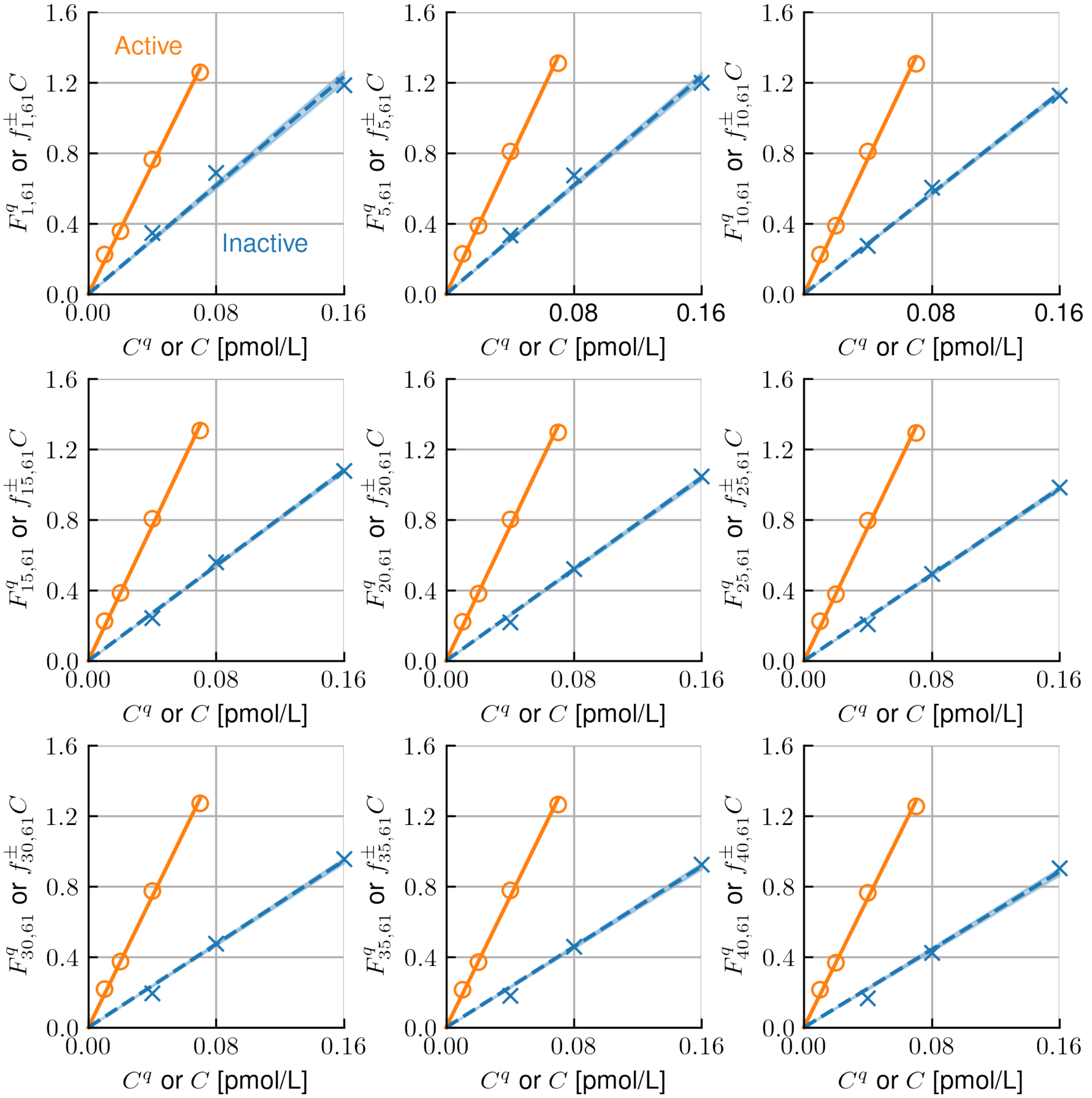

Fig. S61: As Figure S1 with well $w = 61$ (or F1).

Table S61: Molar Fluorescence Parameters for Well F1 ($w = 61$)

Cycle	Inactive		Active	
i	$f^-_{i,61}$	$\sigma^-_{i,61}$	$f^+_{i,61}$	$\sigma^+_{i,61}$
1	7.70	0.068	18.30	0.034
2	7.94	0.063	18.67	0.036
3	8.00	0.061	18.94	0.039
4	7.89	0.056	19.11	0.038
5	7.71	0.050	19.19	0.039
6	7.57	0.043	19.20	0.039
7	7.44	0.038	19.22	0.037
8	7.33	0.034	19.20	0.037
9	7.23	0.030	19.18	0.038
10	7.13	0.027	19.14	0.039
11	7.05	0.025	19.16	0.038
12	6.98	0.022	19.14	0.037
13	6.92	0.024	19.12	0.037
14	6.83	0.024	19.11	0.038
15	6.75	0.024	19.10	0.037
16	6.69	0.026	19.07	0.037
17	6.62	0.025	19.07	0.036
18	6.57	0.023	19.08	0.035
19	6.55	0.028	19.11	0.033
20	6.49	0.029	18.99	0.036
21	6.34	0.027	18.97	0.036
22	6.26	0.022	18.94	0.036
23	6.20	0.023	19.02	0.037
24	6.15	0.024	18.91	0.036
25	6.11	0.026	18.89	0.036
26	6.05	0.025	18.87	0.035
27	6.01	0.026	18.64	0.039
28	5.97	0.028	18.73	0.044
29	5.93	0.028	18.56	0.031
30	5.91	0.030	18.55	0.031
31	5.86	0.030	18.55	0.030
32	5.82	0.032	18.53	0.030
33	5.80	0.033	18.53	0.030
34	5.75	0.034	18.51	0.030
35	5.72	0.034	18.48	0.033
36	5.66	0.034	18.40	0.031
37	5.63	0.035	18.36	0.031
38	5.60	0.036	18.36	0.032
39	5.58	0.039	18.30	0.031
40	5.52	0.042	18.29	0.030
41	5.49	0.043	18.28	0.030
42	5.47	0.046	18.24	0.027
43	5.45	0.052	18.14	0.025
44	5.21	0.040	18.14	0.026
45	5.14	0.038	18.13	0.026

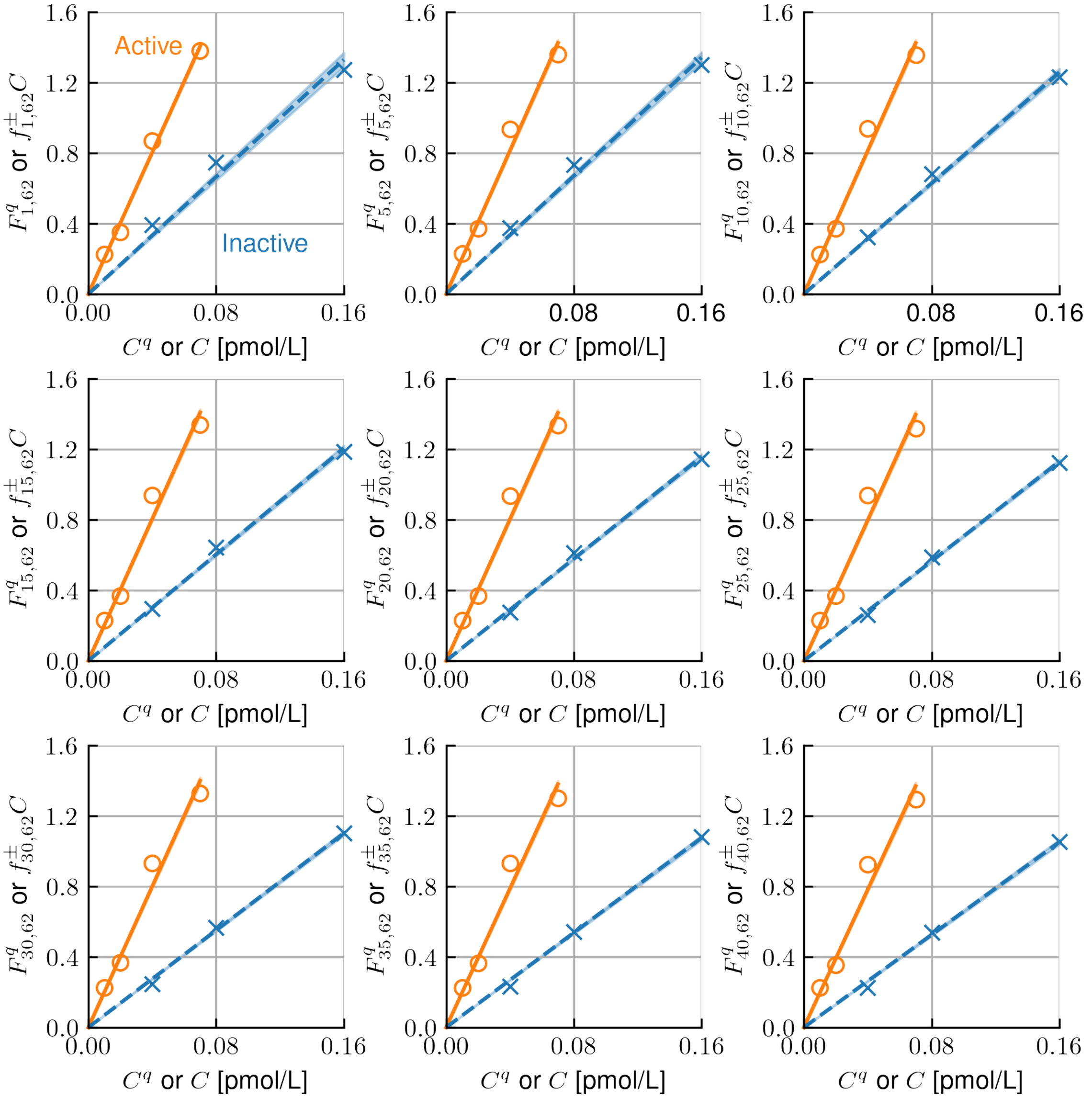

Fig. S62: As Figure S1 with well $w = 62$ (or F2).

Table S62: Molar Fluorescence Parameters for Well F2 ($w = 62$)

Cycle	Inactive		Active	
i	$f^-_{i,62}$	$\sigma^-_{i,62}$	$f^+_{i,62}$	$\sigma^+_{i,62}$
1	8.31	0.084	20.09	0.052
2	8.54	0.075	20.05	0.064
3	8.59	0.074	20.21	0.079
4	8.53	0.067	20.32	0.080
5	8.39	0.061	20.35	0.082
6	8.25	0.058	20.37	0.084
7	8.14	0.052	20.33	0.084
8	8.03	0.048	20.34	0.084
9	7.95	0.044	20.34	0.085
10	7.86	0.042	20.34	0.086
11	7.78	0.039	20.30	0.086
12	7.73	0.035	20.29	0.086
13	7.66	0.033	20.25	0.087
14	7.59	0.032	20.17	0.089
15	7.54	0.032	20.12	0.091
16	7.48	0.029	20.11	0.090
17	7.38	0.029	20.19	0.089
18	7.33	0.028	20.10	0.090
19	7.29	0.028	20.04	0.092
20	7.24	0.027	20.08	0.091
21	7.21	0.025	20.08	0.090
22	7.18	0.026	20.05	0.095
23	7.16	0.021	20.00	0.096
24	7.11	0.022	20.02	0.097
25	7.06	0.022	19.94	0.097
26	7.06	0.023	19.92	0.099
27	7.02	0.022	19.88	0.098
28	6.99	0.023	19.90	0.095
29	6.93	0.023	19.86	0.095
30	6.89	0.023	19.99	0.089
31	6.86	0.024	20.00	0.099
32	6.82	0.024	20.0	0.10
33	6.81	0.024	19.93	0.098
34	6.78	0.025	19.80	0.097
35	6.72	0.026	19.70	0.097
36	6.71	0.026	19.8	0.10
37	6.67	0.025	19.66	0.099
38	6.64	0.025	19.65	0.096
39	6.59	0.026	19.58	0.097
40	6.57	0.028	19.56	0.096
41	6.61	0.028	19.54	0.096
42	6.55	0.028	19.52	0.096
43	6.55	0.029	19.55	0.096
44	6.50	0.030	19.55	0.096
45	6.42	0.029	19.81	0.091

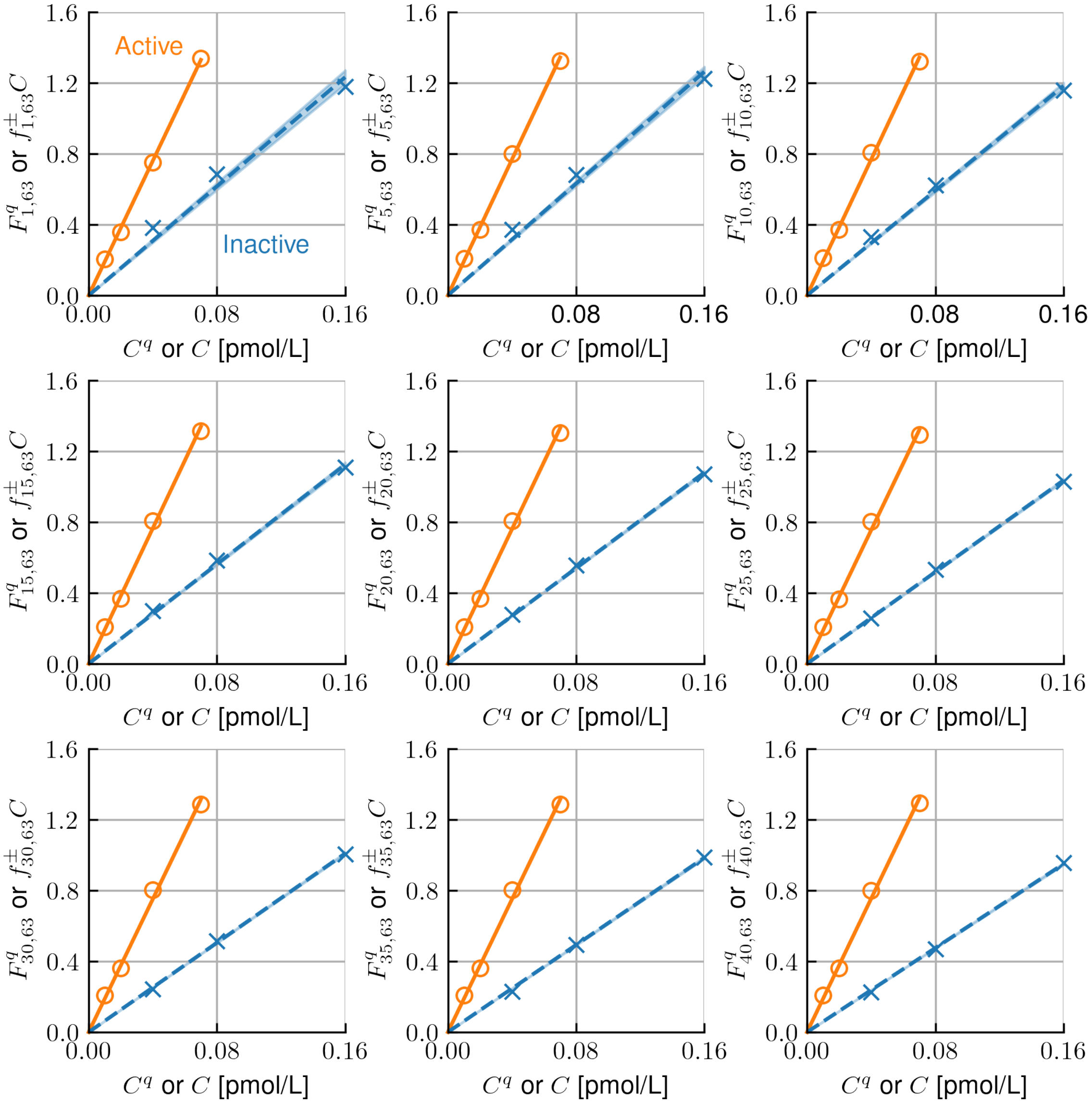

Fig. S63: As Figure S1 with well $w = 63$ (or F3).

Table S63: Molar Fluorescence Parameters for Well F3 ($w = 63$)

Cycle	Inactive		Active	
i	$f^-_{i,63}$	$\sigma^-_{i,63}$	$f^+_{i,63}$	$\sigma^+_{i,63}$
1	7.70	0.082	19.00	0.017
2	7.80	0.079	18.96	0.017
3	7.98	0.072	19.12	0.019
4	7.97	0.065	19.15	0.022
5	7.89	0.059	19.20	0.024
6	7.80	0.053	19.21	0.026
7	7.70	0.048	19.21	0.027
8	7.59	0.044	19.17	0.028
9	7.50	0.040	19.21	0.028
10	7.40	0.037	19.19	0.029
11	7.31	0.034	19.18	0.031
12	7.24	0.031	19.13	0.032
13	7.17	0.028	19.13	0.032
14	7.10	0.025	19.14	0.031
15	7.03	0.024	19.13	0.031
16	6.98	0.021	19.06	0.033
17	6.92	0.019	19.13	0.032
18	6.86	0.017	19.10	0.031
19	6.80	0.016	19.02	0.033
20	6.75	0.015	19.01	0.034
21	6.70	0.014	18.97	0.034
22	6.64	0.013	18.96	0.034
23	6.58	0.012	18.93	0.035
24	6.53	0.011	18.89	0.035
25	6.48	0.012	18.87	0.036
26	6.44	0.011	18.85	0.037
27	6.40	0.011	18.88	0.035
28	6.37	0.011	18.88	0.038
29	6.34	0.012	18.83	0.038
30	6.31	0.010	18.78	0.038
31	6.255	0.0093	18.83	0.036
32	6.23	0.010	18.82	0.037
33	6.21	0.010	18.83	0.036
34	6.18	0.013	18.78	0.037
35	6.16	0.012	18.76	0.037
36	6.11	0.011	18.99	0.031
37	6.06	0.010	18.84	0.035
38	6.017	0.0093	18.80	0.035
39	5.968	0.0091	18.74	0.036
40	5.938	0.0097	18.85	0.034
41	5.911	0.0098	18.61	0.040
42	5.903	0.0082	18.62	0.040
43	5.88	0.011	18.62	0.041
44	5.86	0.012	18.61	0.040
45	5.84	0.011	18.59	0.042

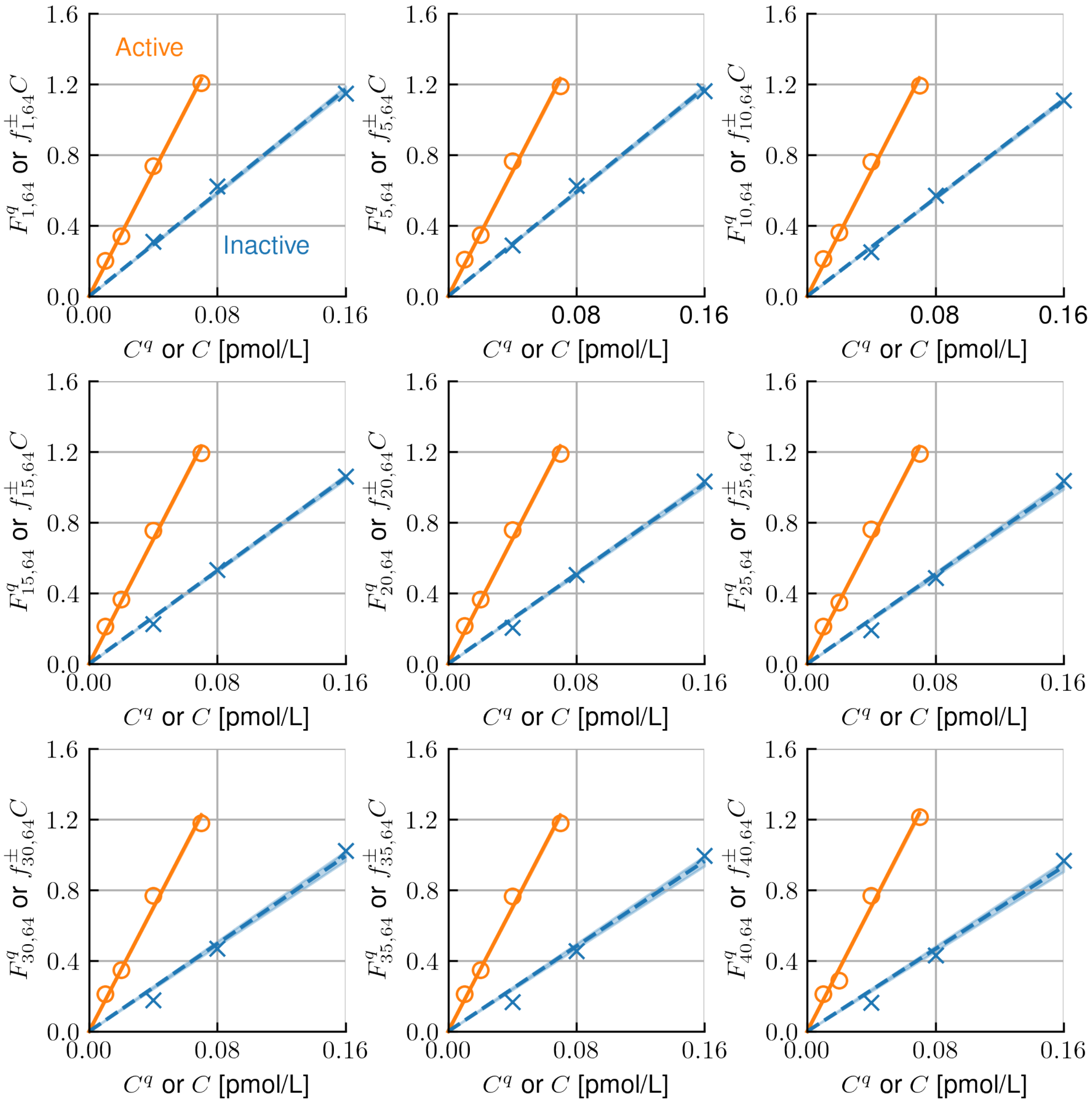

Fig. S64: As Figure S1 with well $w = 64$ (or F4).

Table S64: Molar Fluorescence Parameters for Well F4 ($w = 64$)

Cycle	Inactive		Active	
i	$f^-_{i,64}$	$\sigma^-_{i,64}$	$f^+_{i,64}$	$\sigma^+_{i,64}$
1	7.32	0.035	17.56	0.029
2	7.41	0.030	17.30	0.037
3	7.49	0.035	17.44	0.044
4	7.45	0.033	17.51	0.046
5	7.37	0.029	17.57	0.047
6	7.29	0.026	17.59	0.047
7	7.19	0.024	17.60	0.046
8	7.11	0.022	17.60	0.046
9	7.04	0.022	17.62	0.046
10	6.94	0.023	17.62	0.045
11	6.87	0.023	17.61	0.044
12	6.79	0.025	17.60	0.044
13	6.73	0.025	17.57	0.045
14	6.65	0.026	17.57	0.045
15	6.59	0.028	17.58	0.044
16	6.53	0.029	17.59	0.043
17	6.49	0.031	17.58	0.044
18	6.44	0.032	17.58	0.043
19	6.41	0.034	17.57	0.045
20	6.36	0.036	17.59	0.046
21	6.36	0.039	17.57	0.046
22	6.32	0.040	17.60	0.047
23	6.33	0.043	17.73	0.043
24	6.33	0.045	17.61	0.047
25	6.32	0.049	17.54	0.047
26	6.30	0.051	17.78	0.046
27	6.28	0.051	17.60	0.047
28	6.22	0.052	17.53	0.049
29	6.21	0.055	17.44	0.050
30	6.20	0.057	17.47	0.053
31	6.22	0.062	17.42	0.052
32	6.10	0.058	17.47	0.050
33	6.11	0.056	17.46	0.051
34	6.04	0.058	17.45	0.051
35	6.03	0.059	17.46	0.052
36	6.02	0.062	17.45	0.052
37	5.94	0.060	17.45	0.052
38	5.89	0.059	17.33	0.063
39	5.86	0.058	17.45	0.065
40	5.83	0.060	17.65	0.057
41	5.81	0.062	17.70	0.057
42	5.78	0.063	17.68	0.060
43	5.81	0.067	17.75	0.059
44	5.70	0.062	17.70	0.060
45	5.67	0.064	17.71	0.061

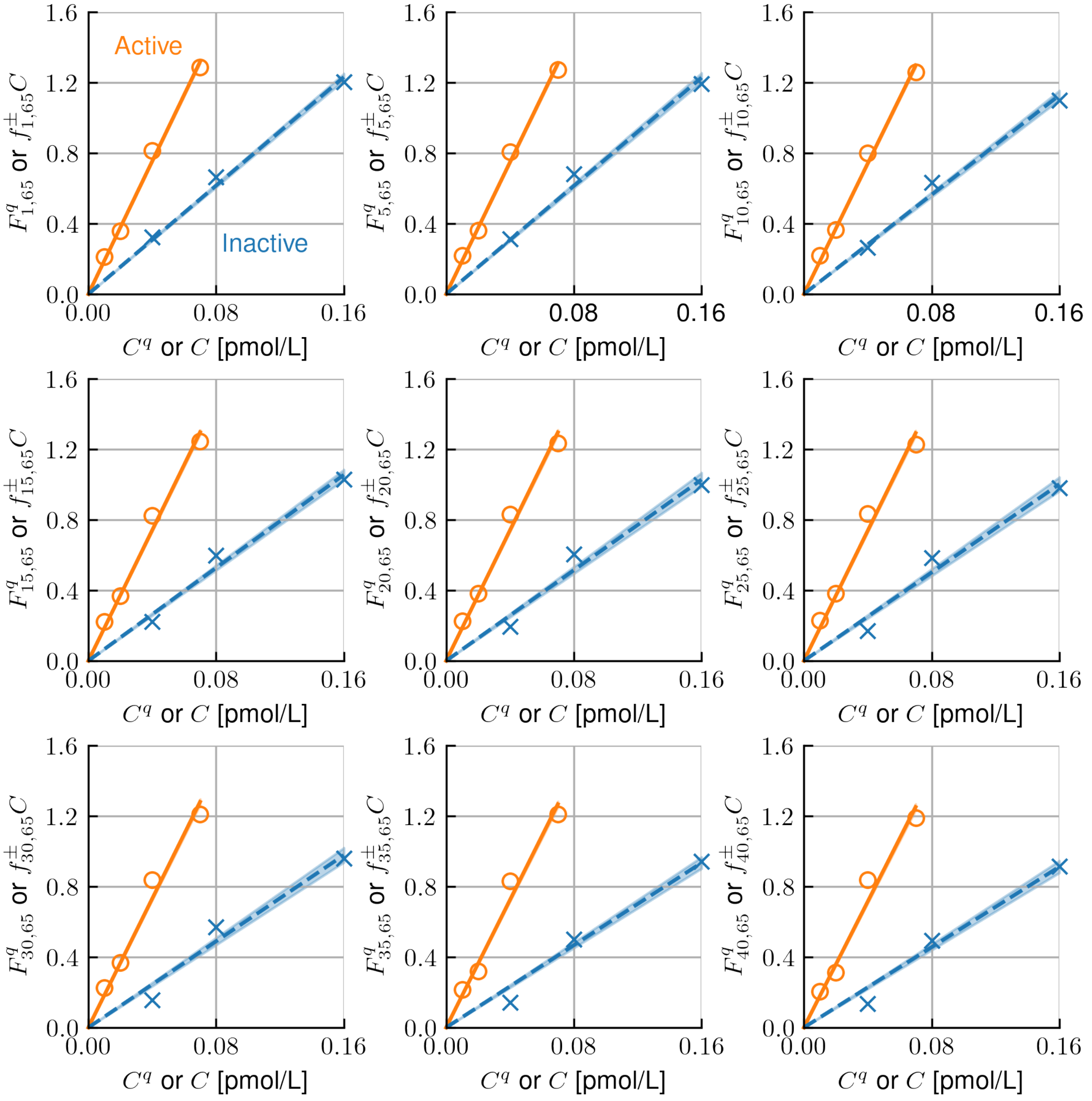

Fig. S65: As Figure S1 with well $w = 65$ (or F5).

Table S65: Molar Fluorescence Parameters for Well F5 ($w = 65$)

Cycle	Inactive		Active	
i	$f^-_{i,65}$	$\sigma^-_{i,65}$	$f^+_{i,65}$	$\sigma^+_{i,65}$
1	7.69	0.041	18.84	0.044
2	7.65	0.056	18.38	0.041
3	7.79	0.055	18.51	0.046
4	7.78	0.056	18.63	0.046
5	7.67	0.055	18.69	0.045
6	7.57	0.053	18.72	0.044
7	7.43	0.052	18.66	0.045
8	7.30	0.052	18.59	0.046
9	7.17	0.054	18.56	0.047
10	7.05	0.056	18.53	0.045
11	6.92	0.058	18.48	0.044
12	6.82	0.058	18.59	0.049
13	6.72	0.060	18.54	0.054
14	6.62	0.061	18.53	0.058
15	6.60	0.060	18.53	0.060
16	6.57	0.072	18.50	0.062
17	6.53	0.078	18.53	0.064
18	6.49	0.079	18.53	0.065
19	6.46	0.079	18.49	0.066
20	6.43	0.081	18.51	0.069
21	6.39	0.081	18.50	0.069
22	6.37	0.081	18.48	0.071
23	6.33	0.082	18.51	0.070
24	6.31	0.082	18.44	0.073
25	6.26	0.083	18.45	0.072
26	6.24	0.083	18.38	0.076
27	6.21	0.086	18.30	0.077
28	6.17	0.085	18.31	0.078
29	6.14	0.086	18.28	0.076
30	6.11	0.086	18.28	0.078
31	6.08	0.085	18.16	0.076
32	6.01	0.086	18.13	0.078
33	5.98	0.091	18.30	0.074
34	5.96	0.088	18.16	0.077
35	5.84	0.067	18.10	0.077
36	5.86	0.075	18.05	0.079
37	5.83	0.074	17.98	0.081
38	5.80	0.071	17.96	0.082
39	5.77	0.071	17.94	0.083
40	5.70	0.071	17.87	0.085
41	5.67	0.067	16.74	0.029
42	5.62	0.066	16.76	0.027
43	5.53	0.064	16.68	0.034
44	5.49	0.065	16.60	0.035
45	5.44	0.063	16.56	0.035

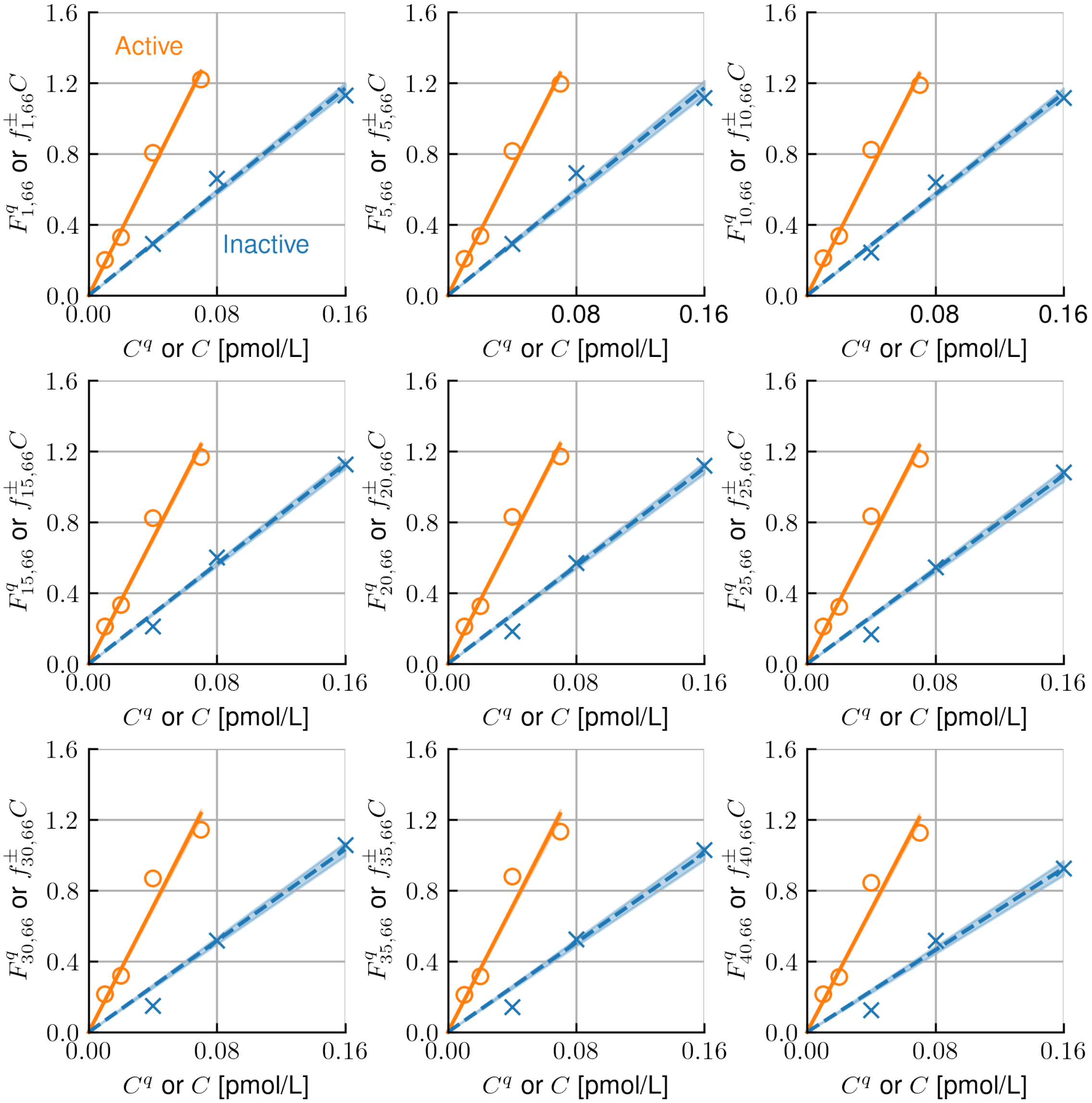

Fig. S66: As Figure S1 with well $w = 66$ (or F6).

Table S66: Molar Fluorescence Parameters for Well F6 ($w = 66$)

Cycle	Inactive		Active	
i	$f^-_{i,66}$	$\sigma^-_{i,66}$	$f^+_{i,66}$	$\sigma^+_{i,66}$
1	7.31	0.061	18.05	0.059
2	7.24	0.078	17.66	0.063
3	7.37	0.083	17.79	0.066
4	7.39	0.086	17.84	0.069
5	7.32	0.085	17.90	0.070
6	7.28	0.079	17.95	0.070
7	7.22	0.072	17.93	0.072
8	7.17	0.068	17.91	0.073
9	7.13	0.063	17.89	0.074
10	7.13	0.059	17.87	0.075
11	7.13	0.056	17.84	0.076
12	7.10	0.055	17.80	0.078
13	7.07	0.056	17.72	0.080
14	7.09	0.054	17.69	0.081
15	7.05	0.056	17.65	0.082
16	7.00	0.059	17.62	0.084
17	6.98	0.060	17.62	0.086
18	6.93	0.061	17.61	0.087
19	6.90	0.063	17.57	0.089
20	6.91	0.066	17.71	0.085
21	6.80	0.066	17.68	0.087
22	6.80	0.068	17.68	0.087
23	6.74	0.068	17.65	0.091
24	6.72	0.070	17.62	0.090
25	6.65	0.071	17.58	0.090
26	6.63	0.073	17.56	0.092
27	6.53	0.072	17.6	0.10
28	6.50	0.075	17.7	0.11
29	6.45	0.076	17.7	0.11
30	6.45	0.079	17.6	0.11
31	6.37	0.079	17.6	0.11
32	6.32	0.080	17.6	0.12
33	6.34	0.081	17.6	0.12
34	6.31	0.082	17.6	0.12
35	6.31	0.081	17.6	0.12
36	5.89	0.078	17.6	0.12
37	5.87	0.079	17.26	0.098
38	5.86	0.082	17.3	0.11
39	5.83	0.080	17.3	0.11
40	5.79	0.084	17.3	0.11
41	5.0	0.10	17.0	0.11
42	4.9	0.10	16.8	0.10
43	5.0	0.10	16.7	0.10
44	5.0	0.10	16.7	0.10
45	4.94	0.100	16.7	0.10

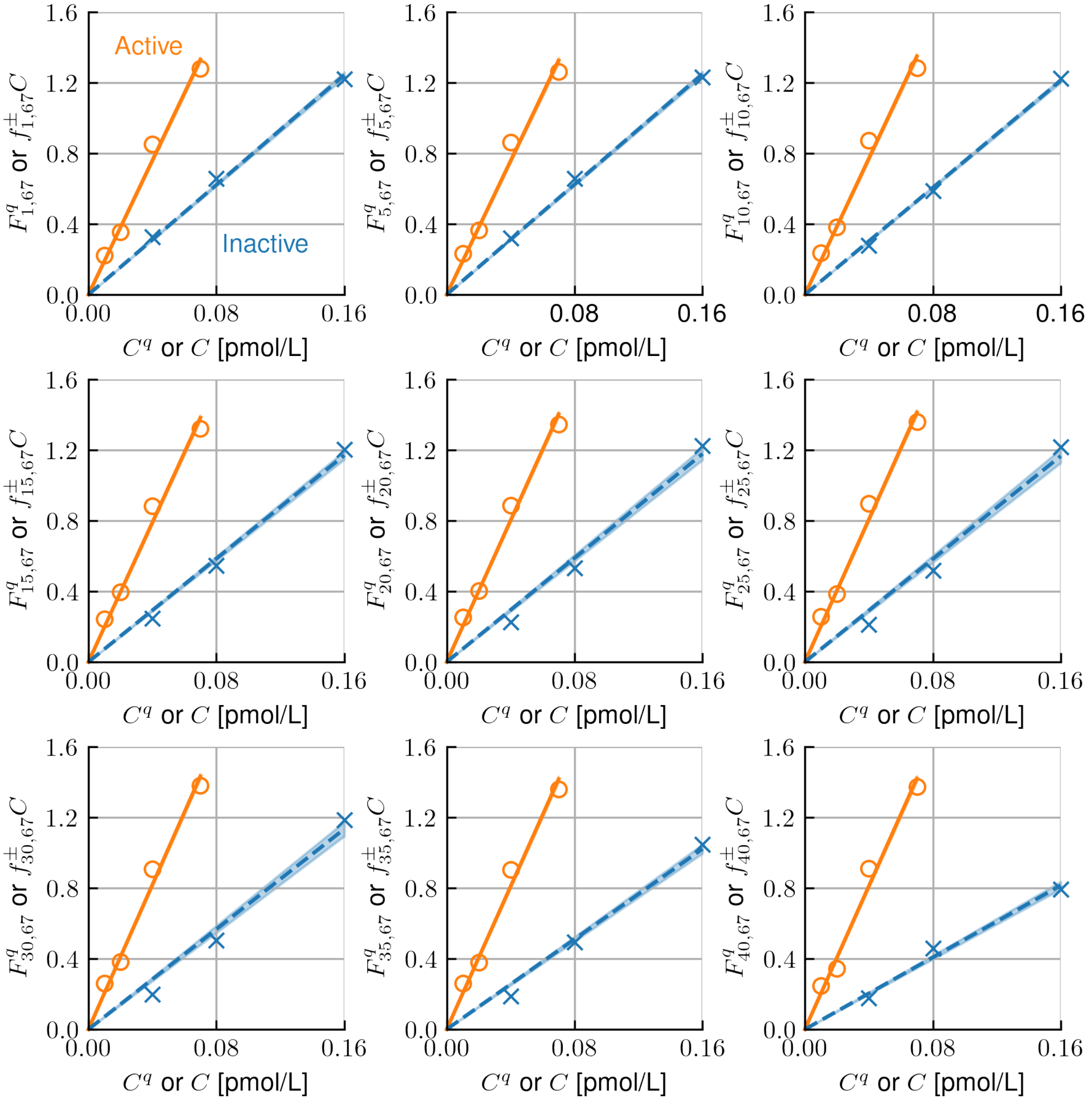

Fig. S67: As Figure S1 with well $w = 67$ (or F7).

Table S67: Molar Fluorescence Parameters for Well F7 ($w = 67$)

Cycle	Inactive		Active	
i	$f^-_{i,67}$	$\sigma^-_{i,67}$	$f^+_{i,67}$	$\sigma^+_{i,67}$
1	7.77	0.031	19.00	0.066
2	7.65	0.038	18.59	0.070
3	7.82	0.036	18.73	0.073
4	7.86	0.033	18.81	0.076
5	7.80	0.027	18.94	0.075
6	7.74	0.018	18.94	0.077
7	7.683	0.0094	18.98	0.078
8	7.658	0.0099	19.08	0.075
9	7.63	0.017	19.14	0.073
10	7.57	0.023	19.27	0.074
11	7.51	0.029	19.53	0.071
12	7.46	0.035	19.59	0.070
13	7.40	0.039	19.67	0.069
14	7.38	0.045	19.70	0.070
15	7.32	0.048	19.75	0.069
16	7.28	0.052	19.82	0.069
17	7.24	0.054	19.88	0.068
18	7.39	0.065	19.97	0.068
19	7.41	0.070	20.04	0.066
20	7.36	0.071	20.04	0.066
21	7.32	0.070	20.03	0.069
22	7.30	0.070	20.11	0.068
23	7.30	0.072	20.13	0.068
24	7.28	0.075	20.13	0.068
25	7.29	0.081	20.18	0.069
26	7.23	0.080	20.23	0.068
27	7.23	0.082	20.36	0.067
28	7.20	0.083	20.42	0.067
29	7.09	0.080	20.48	0.068
30	7.09	0.084	20.47	0.070
31	7.06	0.084	20.20	0.082
32	7.06	0.087	20.25	0.079
33	7.04	0.092	20.30	0.081
34	7.04	0.093	20.26	0.077
35	6.38	0.053	20.23	0.074
36	6.13	0.045	20.24	0.075
37	6.14	0.049	20.28	0.075
38	5.14	0.048	20.36	0.073
39	5.11	0.046	20.28	0.076
40	5.09	0.044	20.28	0.077
41	5.05	0.044	20.35	0.077
42	5.00	0.042	20.35	0.077
43	4.98	0.040	20.36	0.077
44	4.96	0.038	20.35	0.074
45	4.94	0.037	20.37	0.073

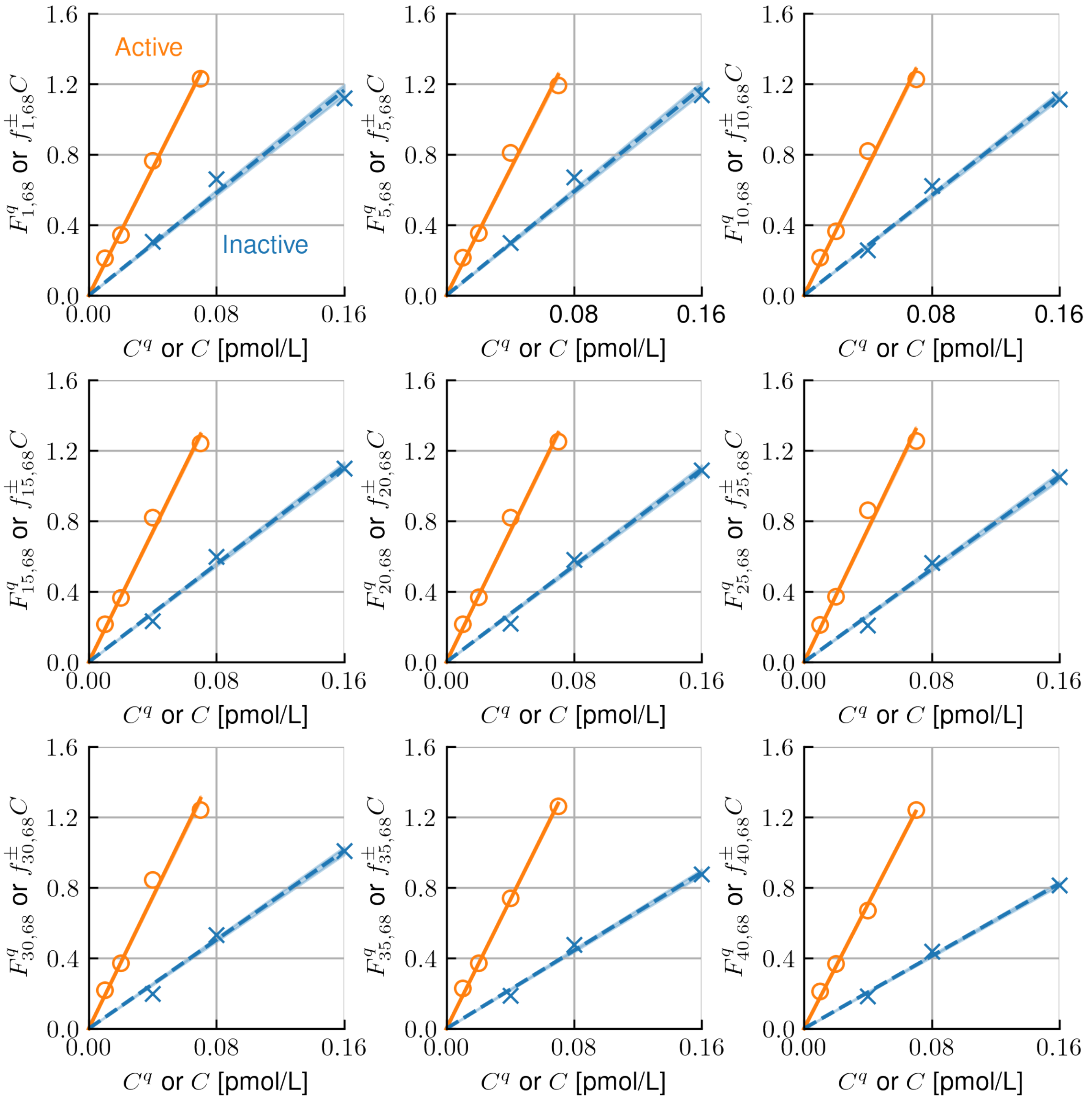

Fig. S68: As Figure S1 with well $w = 68$ (or F8).

Table S68: Molar Fluorescence Parameters for Well F8 ($w = 68$)

Cycle	Inactive		Active	
i	$f^-_{i,68}$	$\sigma^-_{i,68}$	$f^+_{i,68}$	$\sigma^+_{i,68}$
1	7.28	0.065	18.00	0.038
2	7.33	0.063	17.80	0.051
3	7.46	0.066	17.75	0.062
4	7.43	0.068	17.82	0.065
5	7.37	0.065	17.88	0.068
6	7.30	0.059	17.89	0.070
7	7.24	0.055	18.09	0.067
8	7.21	0.049	18.21	0.064
9	7.15	0.047	18.29	0.063
10	7.10	0.045	18.33	0.063
11	7.04	0.045	18.39	0.061
12	6.99	0.045	18.40	0.061
13	6.96	0.046	18.42	0.060
14	6.94	0.046	18.41	0.059
15	6.94	0.046	18.45	0.059
16	6.90	0.046	18.48	0.057
17	6.85	0.041	18.49	0.057
18	6.93	0.044	18.53	0.056
19	6.89	0.045	18.52	0.056
20	6.83	0.046	18.56	0.056
21	6.80	0.047	18.55	0.060
22	6.75	0.046	18.58	0.057
23	6.71	0.047	18.74	0.070
24	6.64	0.048	18.83	0.073
25	6.59	0.047	18.84	0.074
26	6.53	0.047	18.89	0.075
27	6.47	0.046	18.93	0.077
28	6.41	0.045	18.95	0.078
29	6.36	0.045	18.94	0.078
30	6.30	0.044	18.64	0.071
31	6.24	0.044	18.11	0.028
32	5.98	0.040	18.39	0.039
33	5.59	0.041	18.33	0.036
34	5.71	0.035	18.25	0.033
35	5.54	0.034	18.25	0.030
36	5.47	0.031	18.08	0.031
37	5.38	0.032	18.01	0.031
38	5.32	0.026	17.87	0.032
39	5.25	0.025	17.73	0.035
40	5.14	0.024	17.60	0.031
41	5.03	0.024	17.51	0.038
42	4.95	0.022	17.42	0.040
43	4.88	0.020	17.41	0.042
44	4.82	0.019	17.35	0.042
45	4.71	0.018	17.28	0.042

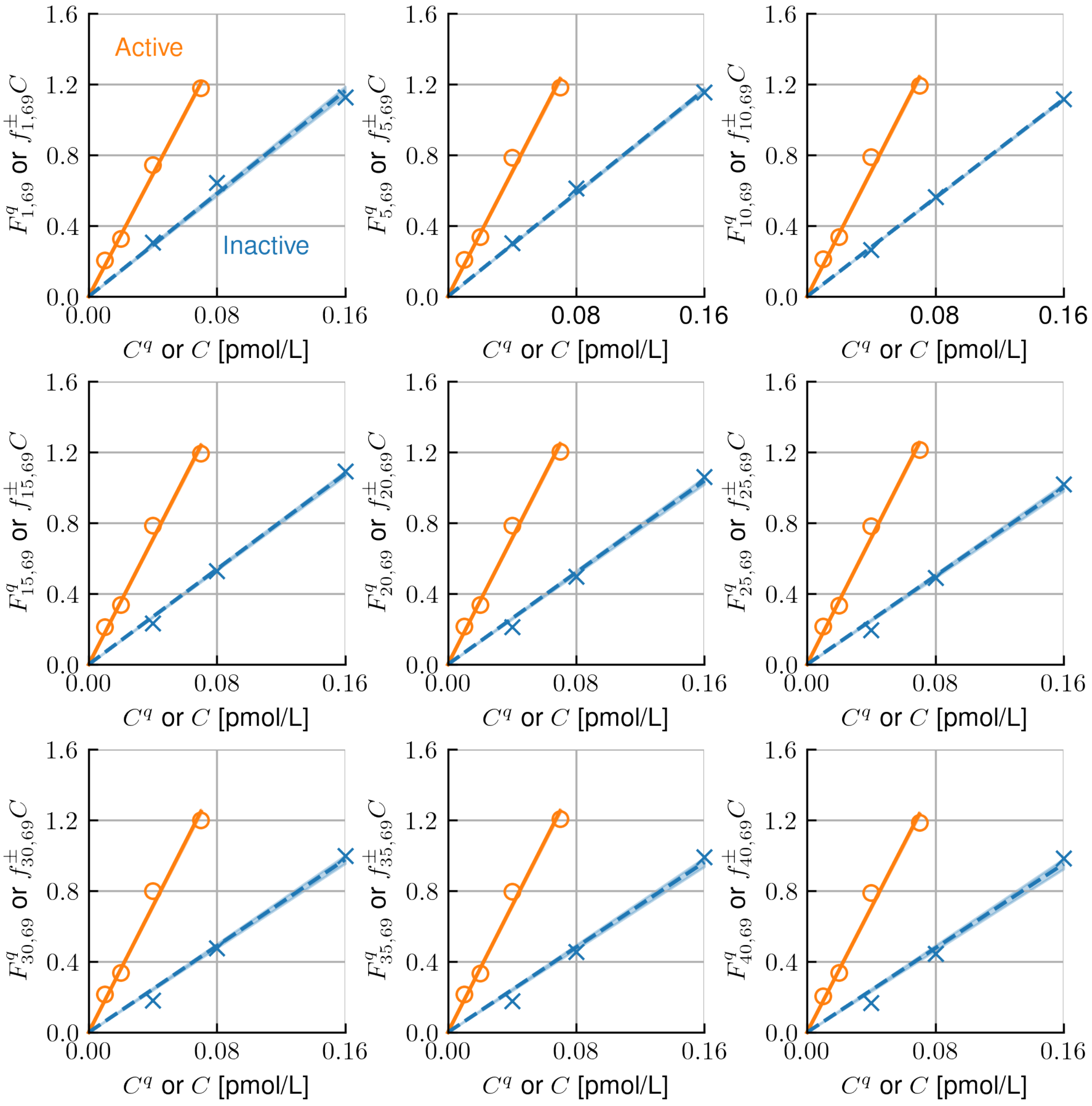

Fig. S69: As Figure S1 with well $w = 69$ (or F9).

Table S69: Molar Fluorescence Parameters for Well F9 ($w = 69$)

Cycle	Inactive		Active	
i	$f^-_{i,69}$	$\sigma^-_{i,69}$	$f^+_{i,69}$	$\sigma^+_{i,69}$
1	7.26	0.051	17.25	0.041
2	7.21	0.049	17.21	0.050
3	7.34	0.038	17.39	0.055
4	7.34	0.031	17.50	0.058
5	7.30	0.023	17.57	0.059
6	7.24	0.017	17.60	0.059
7	7.19	0.012	17.60	0.061
8	7.12	0.010	17.63	0.060
9	7.065	0.0100	17.67	0.059
10	6.97	0.012	17.70	0.059
11	6.90	0.014	17.73	0.057
12	6.85	0.017	17.70	0.058
13	6.87	0.024	17.64	0.059
14	6.83	0.027	17.65	0.059
15	6.74	0.029	17.68	0.058
16	6.70	0.033	17.69	0.059
17	6.65	0.036	17.70	0.057
18	6.60	0.039	17.81	0.054
19	6.57	0.042	17.83	0.053
20	6.49	0.041	17.80	0.054
21	6.56	0.046	17.84	0.054
22	6.56	0.046	17.79	0.053
23	6.50	0.048	17.80	0.053
24	6.50	0.052	17.83	0.052
25	6.25	0.042	17.88	0.051
26	6.27	0.046	17.66	0.060
27	6.12	0.041	17.71	0.062
28	6.15	0.043	17.82	0.061
29	6.11	0.043	17.90	0.061
30	6.11	0.048	17.83	0.062
31	6.07	0.048	17.83	0.062
32	6.06	0.049	17.93	0.061
33	6.03	0.050	17.84	0.062
34	6.01	0.052	17.82	0.058
35	6.01	0.052	17.87	0.059
36	6.01	0.056	17.95	0.065
37	5.98	0.058	17.97	0.061
38	5.98	0.063	17.59	0.061
39	5.97	0.061	17.64	0.057
40	5.95	0.060	17.62	0.059
41	5.95	0.061	17.54	0.061
42	5.96	0.064	17.56	0.063
43	5.84	0.059	17.53	0.062
44	5.86	0.061	17.63	0.061
45	5.85	0.061	17.69	0.064

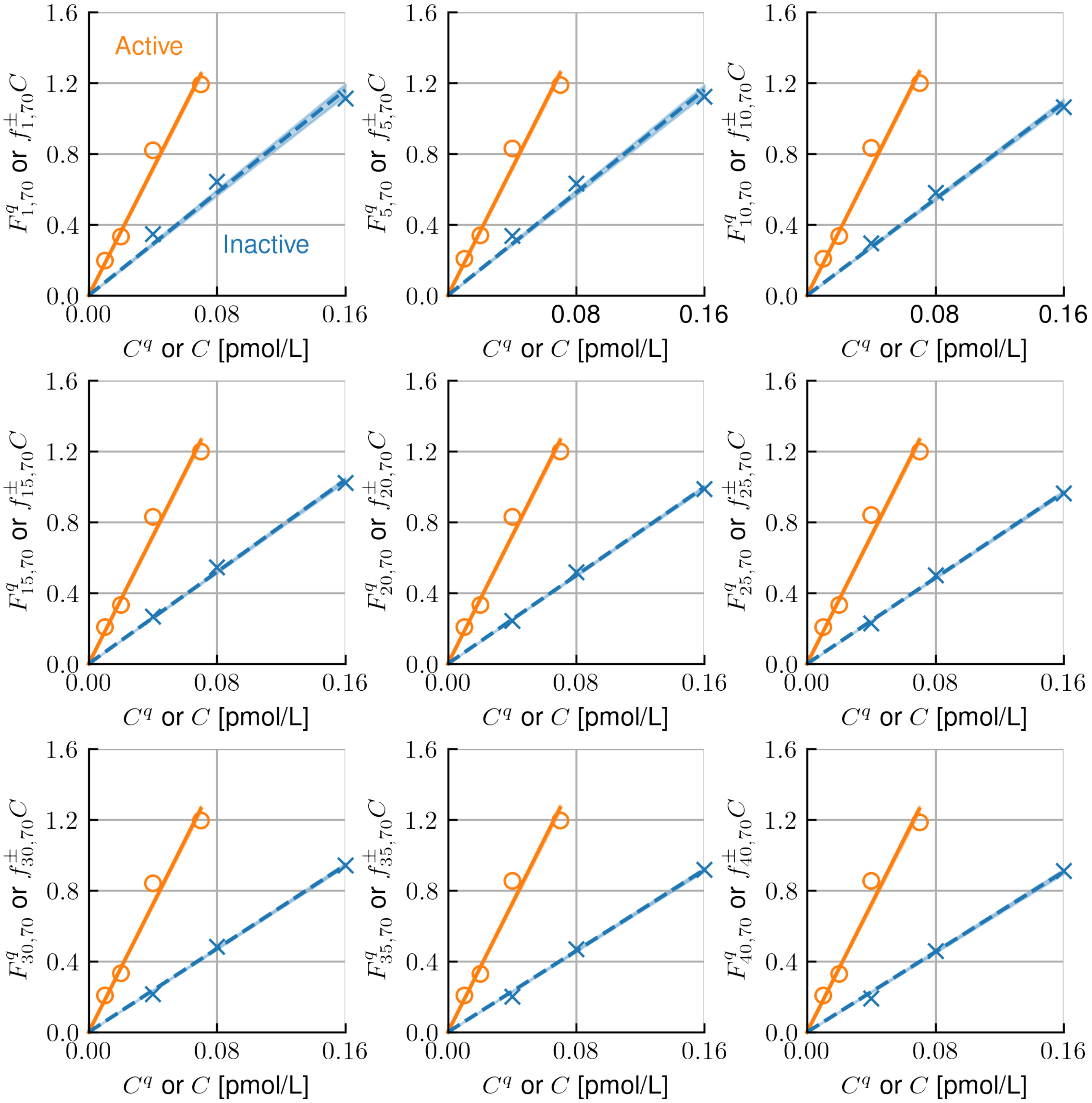

Fig. S70: As Figure S1 with well $w = 70$ (or F10).

Table S70: Molar Fluorescence Parameters for Well F10 ($w = 70$)

Cycle	Inactive		Active	
i	$f_{i,70}^{-}$	$\sigma_{i,70}^{-}$	$f_{i,70}^{+}$	$\sigma_{i,70}^{+}$
1	7.25	0.069	17.85	0.071
2	7.28	0.071	17.59	0.079
3	7.36	0.065	17.75	0.078
4	7.33	0.061	17.87	0.078
5	7.25	0.057	17.93	0.078
6	7.15	0.051	17.96	0.079
7	7.06	0.045	17.98	0.080
8	6.97	0.041	17.98	0.079
9	6.90	0.037	18.00	0.080
10	6.81	0.035	18.01	0.078
11	6.74	0.032	18.04	0.077
12	6.67	0.029	18.01	0.077
13	6.60	0.027	18.02	0.076
14	6.55	0.025	18.03	0.075
15	6.48	0.023	18.02	0.075
16	6.44	0.021	18.02	0.075
17	6.38	0.019	18.02	0.075
18	6.32	0.018	18.02	0.075
19	6.29	0.017	18.04	0.075
20	6.24	0.017	18.02	0.076
21	6.20	0.016	18.02	0.077
22	6.17	0.015	18.03	0.076
23	6.13	0.015	18.08	0.077
24	6.09	0.016	18.05	0.077
25	6.05	0.016	18.04	0.081
26	6.02	0.017	18.03	0.080
27	5.99	0.017	18.04	0.080
28	5.95	0.017	18.05	0.081
29	5.93	0.017	18.01	0.082
30	5.89	0.018	18.03	0.083
31	5.86	0.018	18.03	0.084
32	5.82	0.020	18.16	0.081
33	5.80	0.019	18.14	0.083
34	5.77	0.019	18.11	0.085
35	5.73	0.020	18.09	0.089
36	5.72	0.021	18.04	0.089
37	5.77	0.023	18.07	0.089
38	5.75	0.024	18.07	0.091
39	5.72	0.024	18.04	0.091
40	5.66	0.025	18.00	0.092
41	5.67	0.026	18.01	0.093
42	5.66	0.028	18.01	0.093
43	5.62	0.028	18.04	0.094
44	5.62	0.029	18.05	0.095
45	5.60	0.029	18.04	0.095

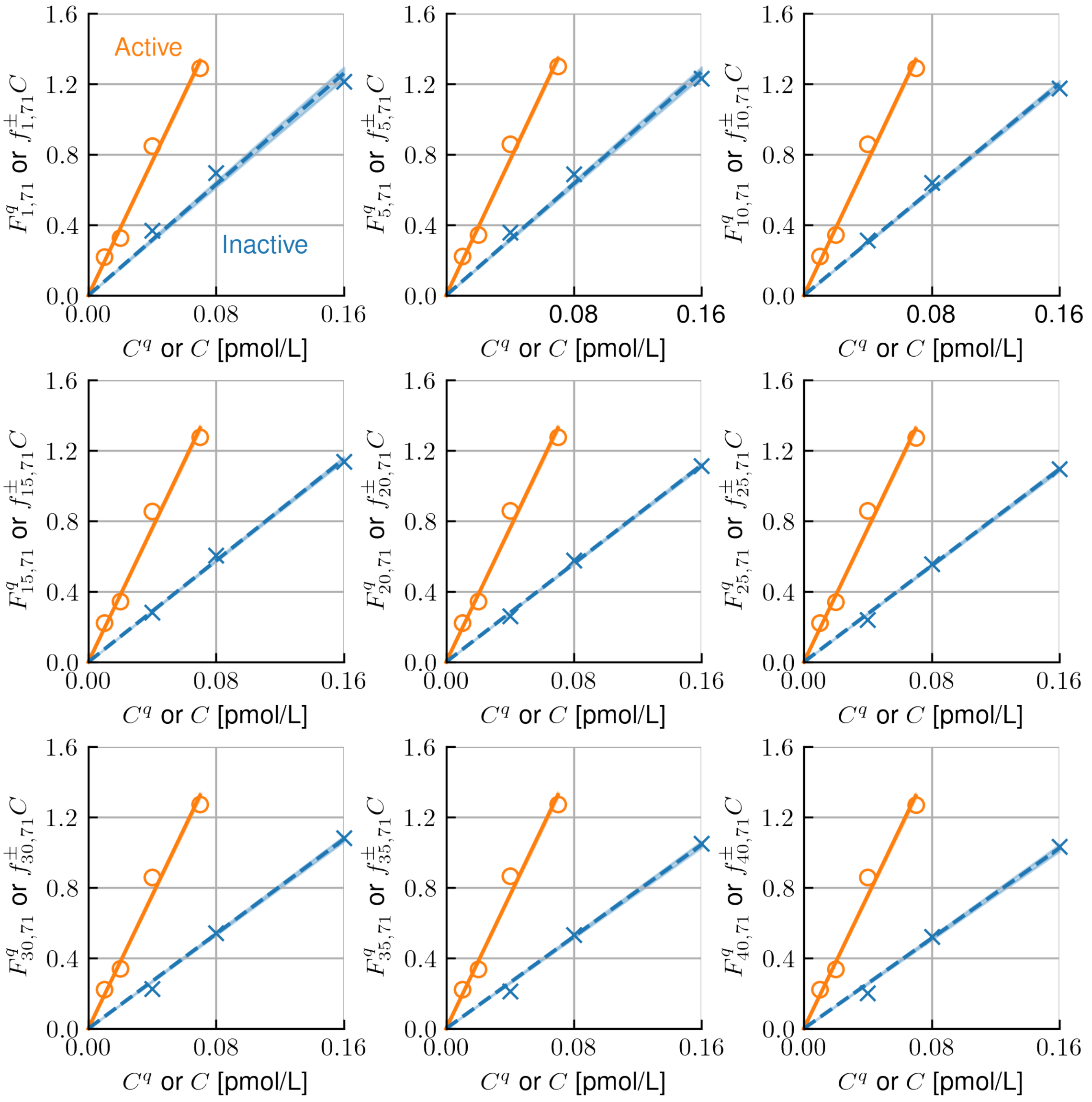

Fig. S71: As Figure S1 with well $w = 71$ (or F11).

Table S71: Molar Fluorescence Parameters for Well F11 ($w = 71$)

Cycle	Inactive		Active	
i	$f_{i,71}^-$	$\sigma_{i,71}^-$	$f_{i,71}^+$	$\sigma_{i,71}^+$
1	7.88	0.069	19.02	0.066
2	7.84	0.073	18.91	0.062
3	7.97	0.069	19.05	0.064
4	7.98	0.063	19.15	0.064
5	7.93	0.056	19.20	0.065
6	7.83	0.050	19.21	0.065
7	7.74	0.045	19.18	0.066
8	7.66	0.040	19.16	0.066
9	7.57	0.037	19.13	0.067
10	7.50	0.034	19.10	0.067
11	7.43	0.032	19.11	0.068
12	7.36	0.029	19.09	0.067
13	7.30	0.027	19.09	0.067
14	7.25	0.025	19.04	0.068
15	7.19	0.024	18.97	0.069
16	7.14	0.022	19.00	0.069
17	7.09	0.022	18.98	0.069
18	7.05	0.020	18.98	0.069
19	7.02	0.022	18.98	0.070
20	6.99	0.020	18.97	0.070
21	6.99	0.021	18.96	0.070
22	6.96	0.021	18.94	0.071
23	6.91	0.022	18.91	0.072
24	6.88	0.022	18.93	0.071
25	6.84	0.024	18.93	0.071
26	6.79	0.026	18.91	0.072
27	6.76	0.027	18.95	0.071
28	6.74	0.028	18.94	0.071
29	6.74	0.030	18.93	0.072
30	6.72	0.031	18.92	0.073
31	6.68	0.031	19.06	0.069
32	6.73	0.035	19.02	0.070
33	6.61	0.033	18.98	0.072
34	6.56	0.034	18.95	0.073
35	6.53	0.035	18.94	0.076
36	6.49	0.036	18.92	0.077
37	6.45	0.036	18.90	0.075
38	6.44	0.037	18.89	0.074
39	6.41	0.037	18.89	0.074
40	6.40	0.039	18.87	0.074
41	6.37	0.039	18.95	0.073
42	6.34	0.040	18.90	0.075
43	6.31	0.039	18.87	0.076
44	6.31	0.040	18.84	0.078
45	6.33	0.041	19.38	0.066

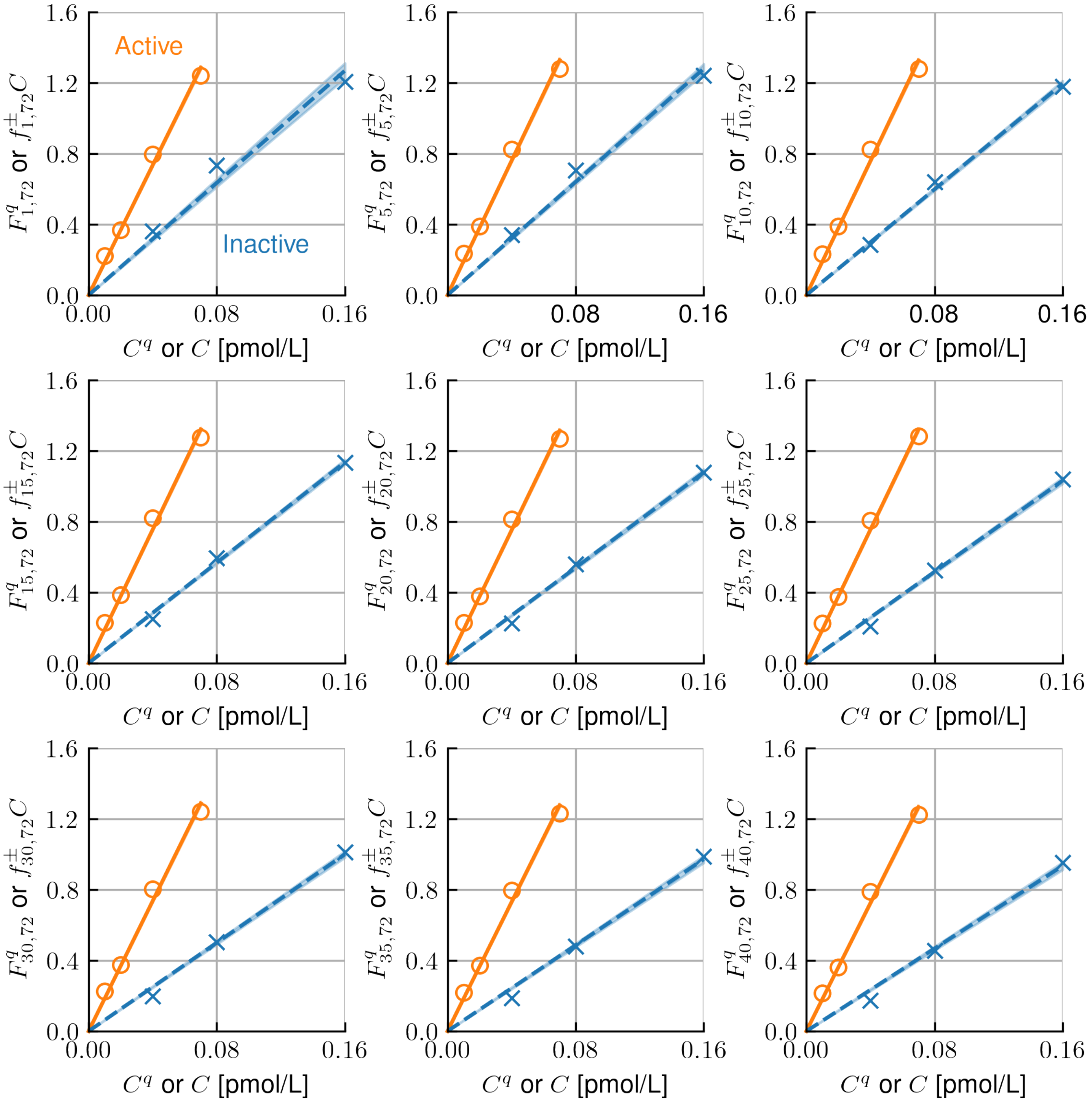

Fig. S72: As Figure S1 with well $w = 72$ (or F12).

Table S72: Molar Fluorescence Parameters for Well F12 ($w = 72$)

Cycle	Inactive		Active	
i	$f^-_{i,72}$	$\sigma^-_{i,72}$	$f^+_{i,72}$	$\sigma^+_{i,72}$
1	7.93	0.090	18.36	0.049
2	8.19	0.078	18.51	0.054
3	8.22	0.073	18.80	0.055
4	8.13	0.064	18.93	0.055
5	8.00	0.056	18.98	0.054
6	7.86	0.049	18.97	0.055
7	7.75	0.044	19.01	0.053
8	7.65	0.039	18.99	0.054
9	7.55	0.035	18.99	0.053
10	7.47	0.034	18.94	0.053
11	7.38	0.031	18.99	0.052
12	7.29	0.031	18.96	0.051
13	7.20	0.032	18.98	0.050
14	7.13	0.031	18.94	0.051
15	7.10	0.030	18.88	0.052
16	7.01	0.030	18.87	0.052
17	6.93	0.031	18.87	0.054
18	6.88	0.031	18.83	0.050
19	6.81	0.033	18.85	0.049
20	6.74	0.035	18.77	0.051
21	6.68	0.037	18.78	0.052
22	6.64	0.035	18.76	0.050
23	6.57	0.035	18.79	0.051
24	6.50	0.037	18.81	0.052
25	6.45	0.035	18.86	0.043
26	6.40	0.039	18.75	0.045
27	6.39	0.041	18.72	0.045
28	6.35	0.034	18.66	0.047
29	6.33	0.036	18.68	0.046
30	6.25	0.037	18.39	0.054
31	6.17	0.036	18.40	0.051
32	6.18	0.039	18.43	0.051
33	6.14	0.039	18.33	0.052
34	6.14	0.041	18.26	0.051
35	6.07	0.041	18.23	0.051
36	6.01	0.040	18.22	0.050
37	5.95	0.040	18.21	0.049
38	5.92	0.042	18.14	0.048
39	5.87	0.043	18.13	0.047
40	5.84	0.044	18.09	0.049
41	5.80	0.045	18.03	0.042
42	5.78	0.045	17.99	0.045
43	5.75	0.047	17.95	0.046
44	5.72	0.047	17.95	0.046
45	5.70	0.050	17.97	0.045

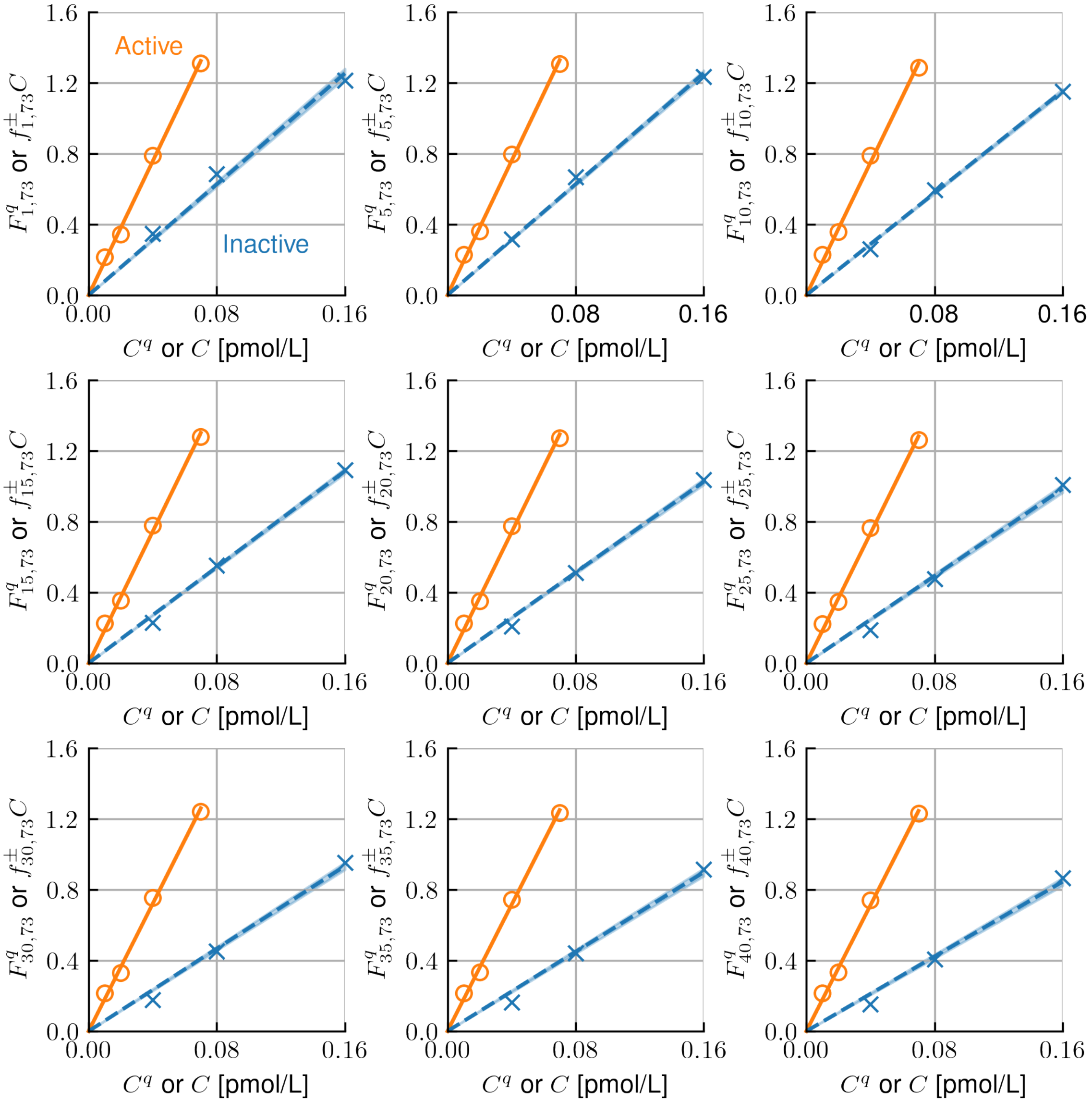

Fig. S73: As Figure S1 with well $w = 73$ (or G1).

Table S73: Molar Fluorescence Parameters for Well G1 ($w = 73$)

Cycle	Inactive		Active	
i	$f^-_{i,73}$	$\sigma^-_{i,73}$	$f^+_{i,73}$	$\sigma^+_{i,73}$
1	7.83	0.055	18.91	0.032
2	8.13	0.043	18.91	0.032
3	8.14	0.042	18.99	0.035
4	8.00	0.037	18.98	0.036
5	7.84	0.032	18.97	0.036
6	7.69	0.028	18.92	0.036
7	7.54	0.025	18.85	0.036
8	7.41	0.024	18.80	0.037
9	7.31	0.023	18.78	0.036
10	7.21	0.023	18.72	0.037
11	7.12	0.024	18.70	0.035
12	7.03	0.025	18.69	0.035
13	6.93	0.027	18.66	0.036
14	6.87	0.030	18.63	0.035
15	6.79	0.030	18.59	0.035
16	6.72	0.028	18.51	0.036
17	6.67	0.030	18.54	0.035
18	6.58	0.032	18.50	0.036
19	6.48	0.033	18.59	0.035
20	6.41	0.034	18.48	0.036
21	6.34	0.034	18.47	0.036
22	6.33	0.035	18.54	0.034
23	6.24	0.039	18.43	0.037
24	6.19	0.042	18.38	0.038
25	6.16	0.045	18.32	0.033
26	6.04	0.038	18.23	0.033
27	5.97	0.039	18.03	0.036
28	5.95	0.041	18.10	0.034
29	5.89	0.043	17.99	0.038
30	5.83	0.043	18.00	0.034
31	5.79	0.043	17.92	0.033
32	5.76	0.044	17.90	0.033
33	5.74	0.046	17.87	0.033
34	5.68	0.046	17.89	0.033
35	5.61	0.045	17.85	0.033
36	5.59	0.046	17.83	0.032
37	5.52	0.046	17.83	0.033
38	5.36	0.044	17.83	0.033
39	5.30	0.044	17.77	0.033
40	5.28	0.046	17.79	0.032
41	5.26	0.048	17.78	0.031
42	5.23	0.048	17.86	0.031
43	5.19	0.049	17.64	0.034
44	5.19	0.052	17.62	0.031
45	5.14	0.051	17.64	0.031

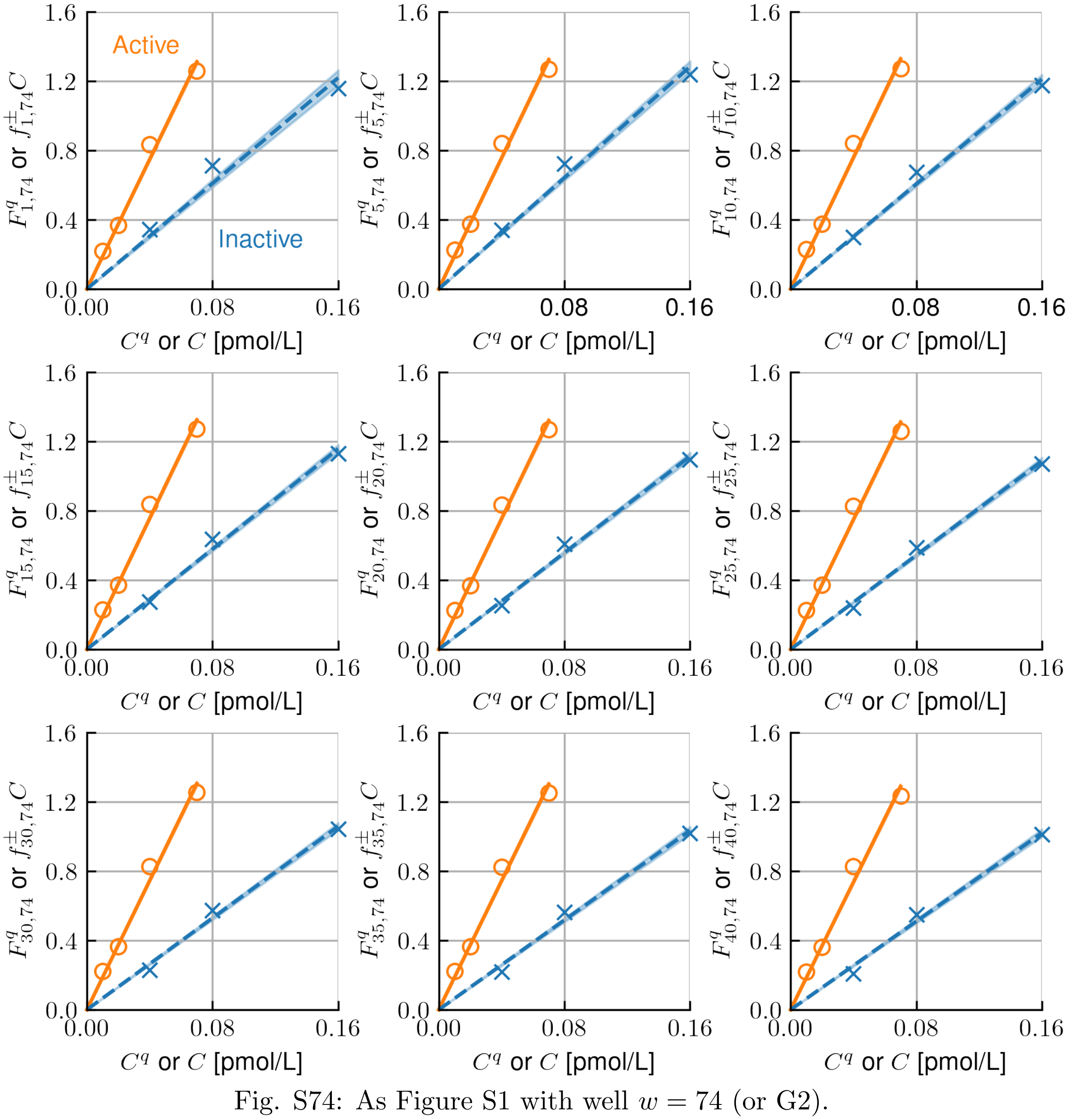

Fig. S74: As Figure S1 with well $w = 74$ (or G2).

Table S74: Molar Fluorescence Parameters for Well G2 ($w = 74$)

Cycle	Inactive		Active	
i	$f^-_{i,74}$	$\sigma^-_{i,74}$	$f^+_{i,74}$	$\sigma^+_{i,74}$
1	7.62	0.091	18.71	0.061
2	8.02	0.079	18.54	0.062
3	8.16	0.076	18.73	0.063
4	8.12	0.072	18.81	0.063
5	8.02	0.067	18.90	0.062
6	7.93	0.064	18.95	0.061
7	7.84	0.060	18.93	0.061
8	7.74	0.058	18.97	0.064
9	7.65	0.055	18.97	0.062
10	7.57	0.054	18.96	0.062
11	7.49	0.052	18.96	0.062
12	7.45	0.049	18.95	0.062
13	7.38	0.047	18.96	0.061
14	7.30	0.047	18.95	0.061
15	7.23	0.047	18.91	0.061
16	7.17	0.045	18.90	0.062
17	7.10	0.044	18.90	0.061
18	7.06	0.043	19.00	0.063
19	7.02	0.044	18.91	0.060
20	6.97	0.043	18.85	0.060
21	6.92	0.042	18.86	0.059
22	6.88	0.042	18.79	0.059
23	6.85	0.041	18.80	0.059
24	6.82	0.041	18.71	0.060
25	6.79	0.040	18.72	0.059
26	6.74	0.040	18.81	0.057
27	6.72	0.040	18.73	0.061
28	6.72	0.038	18.68	0.062
29	6.66	0.040	18.62	0.061
30	6.61	0.041	18.63	0.061
31	6.71	0.038	18.57	0.061
32	6.56	0.042	18.61	0.062
33	6.53	0.042	18.67	0.059
34	6.45	0.045	18.72	0.058
35	6.47	0.045	18.59	0.060
36	6.42	0.045	18.51	0.062
37	6.37	0.045	18.69	0.057
38	6.40	0.047	18.53	0.061
39	6.35	0.046	18.46	0.063
40	6.38	0.044	18.41	0.064
41	6.26	0.047	18.36	0.065
42	6.20	0.048	18.36	0.065
43	6.17	0.050	18.39	0.065
44	6.12	0.049	18.37	0.066
45	6.08	0.045	18.39	0.065

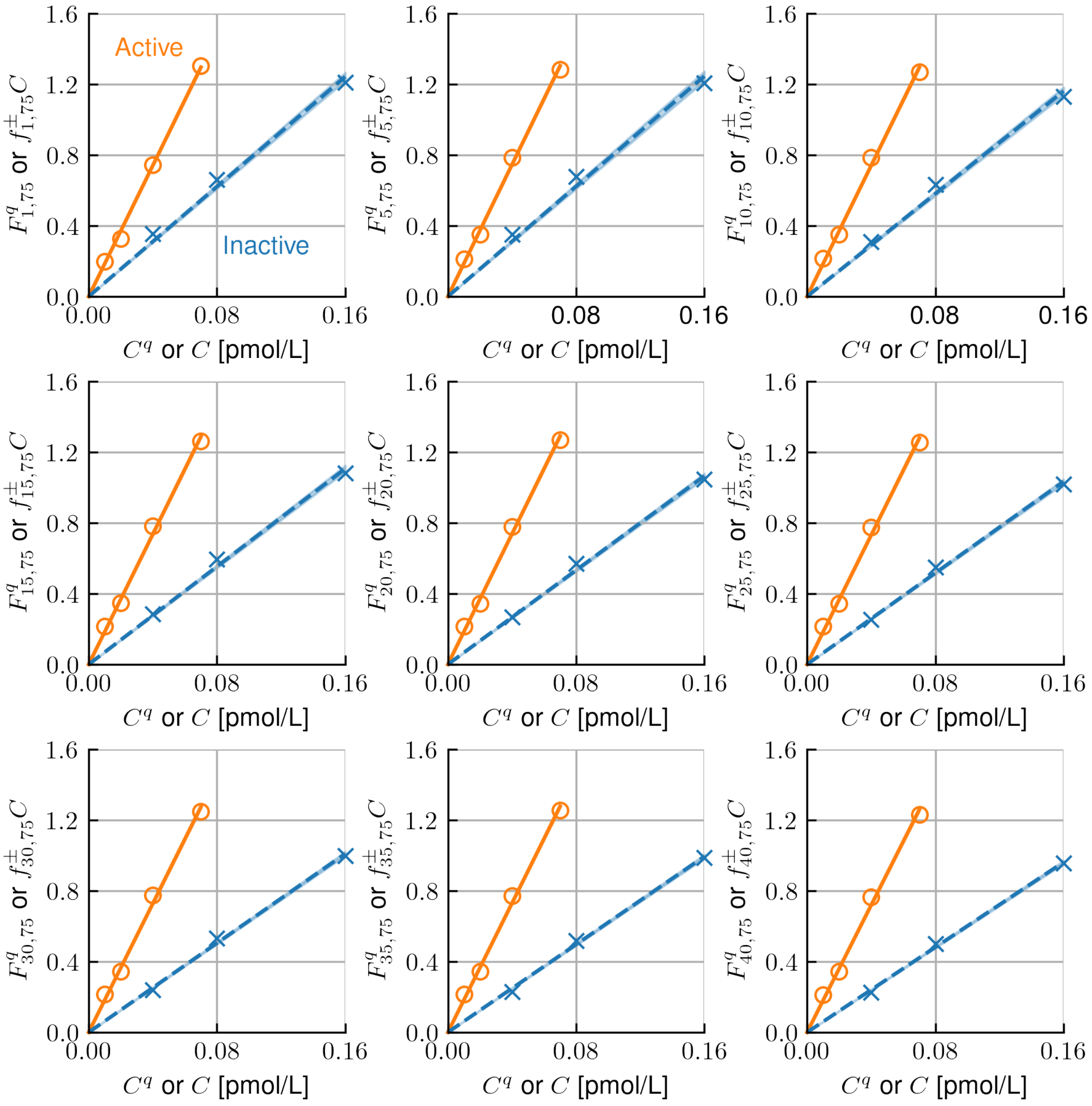

Fig. S75: As Figure S1 with well $w = 75$ (or G3).

Table S75: Molar Fluorescence Parameters for Well G3 ($w = 75$)

Cycle	Inactive		Active	
i	$f^-_{i,75}$	$\sigma^-_{i,75}$	$f^+_{i,75}$	$\sigma^+_{i,75}$
1	7.76	0.047	18.51	0.027
2	7.94	0.054	18.51	0.024
3	8.03	0.057	18.61	0.027
4	7.90	0.060	18.67	0.031
5	7.78	0.057	18.63	0.033
6	7.65	0.055	18.62	0.034
7	7.54	0.052	18.56	0.036
8	7.44	0.049	18.54	0.036
9	7.35	0.046	18.51	0.037
10	7.26	0.045	18.50	0.037
11	7.16	0.043	18.48	0.037
12	7.10	0.041	18.46	0.037
13	7.03	0.040	18.41	0.038
14	6.98	0.037	18.40	0.037
15	6.92	0.035	18.40	0.039
16	6.87	0.033	18.38	0.037
17	6.81	0.033	18.40	0.037
18	6.76	0.032	18.39	0.037
19	6.71	0.030	18.38	0.037
20	6.66	0.029	18.44	0.035
21	6.63	0.027	18.42	0.035
22	6.56	0.028	18.40	0.037
23	6.54	0.026	18.35	0.036
24	6.49	0.025	18.28	0.037
25	6.46	0.025	18.27	0.036
26	6.43	0.024	18.25	0.037
27	6.40	0.024	18.25	0.036
28	6.37	0.023	18.25	0.040
29	6.34	0.022	18.23	0.038
30	6.31	0.022	18.20	0.038
31	6.28	0.022	18.21	0.037
32	6.25	0.022	18.19	0.035
33	6.25	0.026	18.26	0.035
34	6.20	0.022	18.25	0.036
35	6.21	0.021	18.25	0.036
36	6.21	0.018	18.32	0.033
37	6.15	0.019	18.23	0.034
38	6.08	0.019	18.21	0.033
39	6.04	0.019	18.13	0.033
40	6.01	0.017	17.97	0.037
41	5.97	0.021	17.92	0.038
42	5.93	0.019	17.92	0.038
43	5.86	0.020	17.91	0.037
44	5.84	0.018	17.89	0.038
45	5.83	0.018	17.87	0.039

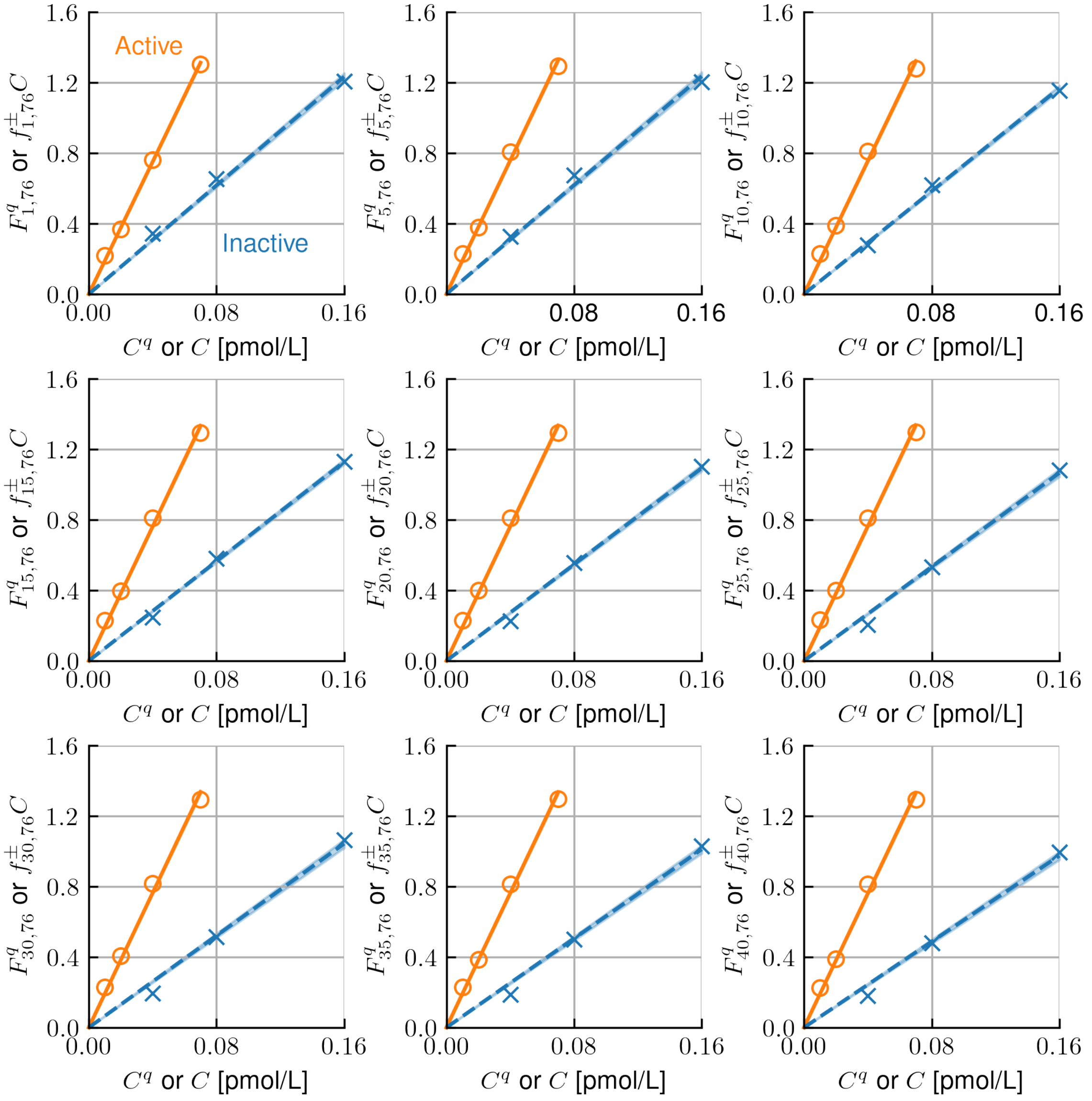

Fig. S76: As Figure S1 with well $w = 76$ (or G4).

Table S76: Molar Fluorescence Parameters for Well G4 ($w = 76$)

Cycle	Inactive		Active	
i	$f_{i,76}^-$	$\sigma_{i,76}^-$	$f_{i,76}^+$	$\sigma_{i,76}^+$
1	7.71	0.042	18.75	0.021
2	7.82	0.055	18.70	0.031
3	7.89	0.054	18.89	0.035
4	7.82	0.051	18.92	0.039
5	7.72	0.047	18.95	0.041
6	7.62	0.043	18.96	0.042
7	7.53	0.037	18.93	0.044
8	7.43	0.034	18.91	0.045
9	7.34	0.032	18.87	0.047
10	7.30	0.028	18.87	0.047
11	7.24	0.026	19.04	0.046
12	7.21	0.026	19.05	0.045
13	7.17	0.026	19.02	0.045
14	7.12	0.027	19.04	0.045
15	7.06	0.029	19.03	0.045
16	7.01	0.030	19.03	0.044
17	6.95	0.031	19.13	0.042
18	6.90	0.032	19.07	0.044
19	6.88	0.033	19.06	0.044
20	6.84	0.036	19.05	0.044
21	6.80	0.037	19.08	0.043
22	6.76	0.039	19.06	0.043
23	6.72	0.041	19.06	0.044
24	6.69	0.043	19.04	0.040
25	6.66	0.044	19.08	0.045
26	6.63	0.046	19.06	0.044
27	6.69	0.050	19.04	0.044
28	6.65	0.051	19.13	0.046
29	6.52	0.047	19.10	0.047
30	6.52	0.048	19.08	0.047
31	6.54	0.049	19.17	0.051
32	6.43	0.046	19.11	0.046
33	6.37	0.046	19.12	0.045
34	6.36	0.046	19.15	0.042
35	6.32	0.047	19.06	0.042
36	6.26	0.047	19.17	0.036
37	6.21	0.047	19.01	0.041
38	6.18	0.048	19.03	0.040
39	6.16	0.048	19.01	0.041
40	6.09	0.047	19.01	0.042
41	6.05	0.048	19.00	0.041
42	6.02	0.048	18.99	0.042
43	6.01	0.048	19.09	0.040
44	6.00	0.049	18.81	0.048
45	6.00	0.048	18.57	0.056

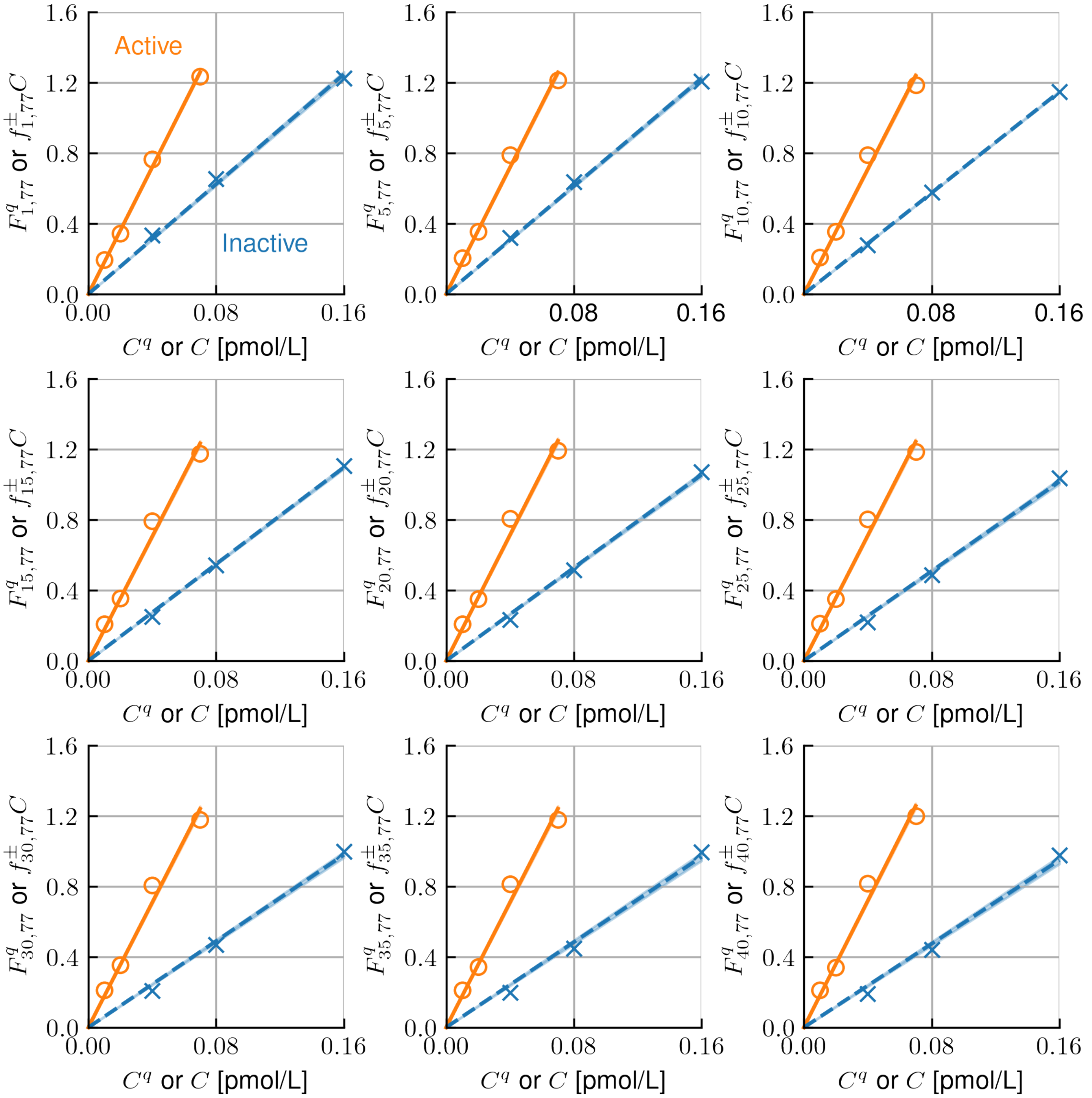

Fig. S77: As Figure S1 with well $w = 77$ (or G5).

Table S77: Molar Fluorescence Parameters for Well G5 ($w = 77$)

Cycle	Inactive		Active	
i	$f^-_{i,77}$	$\sigma^-_{i,77}$	$f^+_{i,77}$	$\sigma^+_{i,77}$
1	7.79	0.031	17.98	0.032
2	7.87	0.028	17.91	0.037
3	7.91	0.025	17.98	0.043
4	7.85	0.023	18.02	0.045
5	7.65	0.024	17.95	0.051
6	7.54	0.016	17.86	0.052
7	7.43	0.012	17.81	0.055
8	7.333	0.0086	17.75	0.056
9	7.241	0.0065	17.71	0.057
10	7.173	0.0069	17.70	0.058
11	7.083	0.0088	17.65	0.058
12	7.03	0.012	17.63	0.058
13	6.97	0.014	17.58	0.060
14	6.92	0.016	17.61	0.063
15	6.86	0.018	17.61	0.065
16	6.80	0.020	17.57	0.066
17	6.75	0.022	17.76	0.060
18	6.69	0.022	17.86	0.059
19	6.69	0.024	17.83	0.064
20	6.60	0.026	17.83	0.066
21	6.53	0.024	17.76	0.064
22	6.47	0.026	17.76	0.065
23	6.42	0.027	17.80	0.067
24	6.40	0.029	17.76	0.066
25	6.35	0.031	17.75	0.066
26	6.35	0.036	17.77	0.067
27	6.26	0.034	17.76	0.068
28	6.23	0.036	17.77	0.068
29	6.23	0.040	17.72	0.070
30	6.12	0.034	17.71	0.070
31	6.08	0.037	17.71	0.070
32	6.05	0.041	17.76	0.071
33	6.03	0.041	17.77	0.071
34	6.06	0.045	17.74	0.072
35	6.05	0.045	17.73	0.074
36	6.02	0.044	17.74	0.073
37	6.03	0.046	17.79	0.073
38	5.99	0.042	17.77	0.074
39	5.96	0.043	17.78	0.074
40	5.93	0.044	17.95	0.070
41	5.89	0.045	17.93	0.071
42	5.87	0.045	17.98	0.070
43	5.87	0.047	17.98	0.071
44	5.87	0.050	18.00	0.071
45	5.86	0.052	17.98	0.073

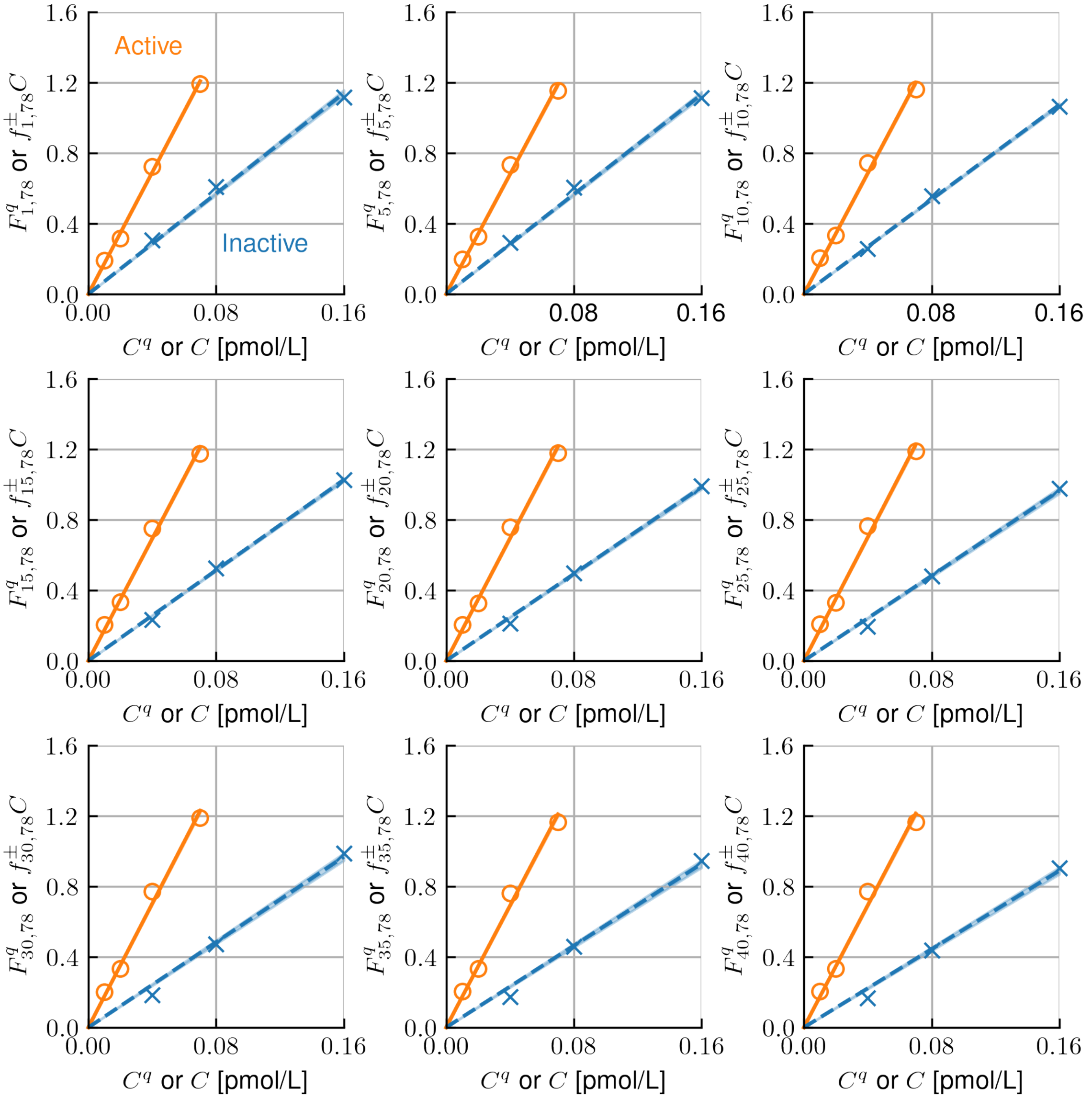

Fig. S78: As Figure S1 with well $w = 78$ (or G6).

Table S78: Molar Fluorescence Parameters for Well G6 ($w = 78$)

Cycle	Inactive		Active	
i	$f^-_{i,78}$	$\sigma^-_{i,78}$	$f^+_{i,78}$	$\sigma^+_{i,78}$
1	7.13	0.036	17.25	0.029
2	7.09	0.045	16.83	0.033
3	7.16	0.044	16.85	0.038
4	7.15	0.038	16.92	0.039
5	7.08	0.031	16.97	0.041
6	6.99	0.023	16.96	0.041
7	6.90	0.020	16.95	0.042
8	6.83	0.016	16.98	0.044
9	6.77	0.017	17.00	0.047
10	6.71	0.017	17.13	0.044
11	6.64	0.017	17.27	0.041
12	6.59	0.017	17.31	0.040
13	6.54	0.017	17.31	0.041
14	6.48	0.017	17.33	0.043
15	6.42	0.019	17.31	0.044
16	6.37	0.020	17.31	0.044
17	6.33	0.022	17.36	0.044
18	6.27	0.023	17.37	0.045
19	6.22	0.024	17.36	0.047
20	6.16	0.025	17.35	0.047
21	6.14	0.027	17.41	0.047
22	6.09	0.028	17.40	0.047
23	6.10	0.031	17.41	0.048
24	6.08	0.033	17.42	0.048
25	6.03	0.034	17.51	0.049
26	6.01	0.036	17.48	0.049
27	5.99	0.037	17.53	0.051
28	6.02	0.038	17.56	0.050
29	6.02	0.040	17.55	0.050
30	6.05	0.044	17.54	0.051
31	5.93	0.041	17.56	0.052
32	5.91	0.043	17.70	0.050
33	5.89	0.044	17.58	0.041
34	5.85	0.043	17.28	0.050
35	5.80	0.043	17.26	0.052
36	5.77	0.045	17.29	0.053
37	5.61	0.040	17.27	0.054
38	5.61	0.041	17.42	0.052
39	5.62	0.039	17.34	0.055
40	5.56	0.041	17.32	0.056
41	5.55	0.039	17.30	0.060
42	5.56	0.043	17.37	0.059
43	5.53	0.043	17.14	0.043
44	5.55	0.046	17.18	0.048
45	5.49	0.045	17.15	0.047

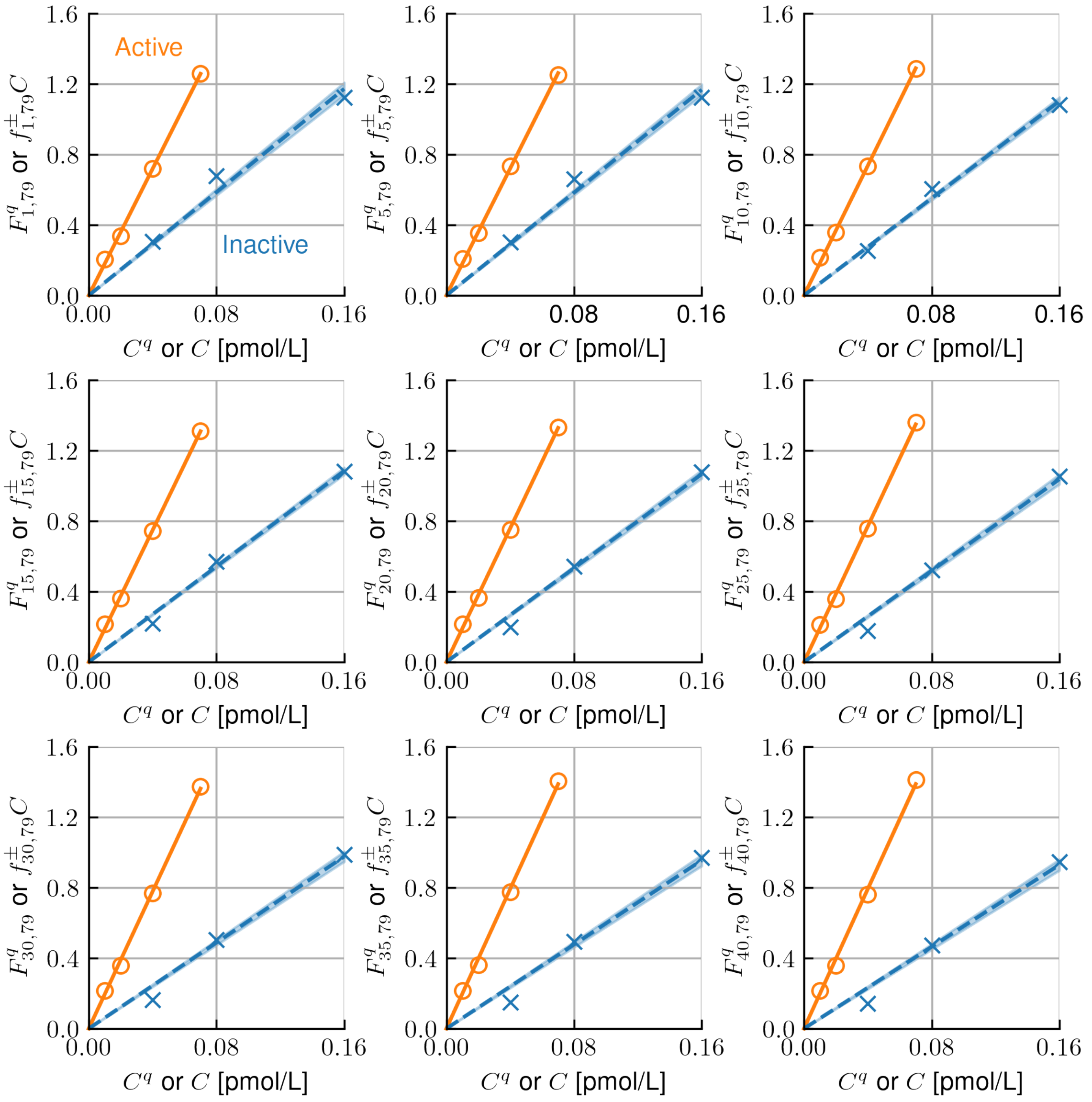

Fig. S79: As Figure S1 with well $w = 79$ (or G7).

Table S79: Molar Fluorescence Parameters for Well G7 ($w = 79$)

Cycle	Inactive		Active	
i	$f^-_{i,79}$	$\sigma^-_{i,79}$	$f^+_{i,79}$	$\sigma^+_{i,79}$
1	7.33	0.075	17.96	0.019
2	7.37	0.074	17.75	0.017
3	7.47	0.071	17.87	0.018
4	7.40	0.069	17.98	0.019
5	7.30	0.064	18.02	0.019
6	7.20	0.059	18.05	0.020
7	7.12	0.054	18.20	0.019
8	7.05	0.049	18.27	0.019
9	6.98	0.046	18.36	0.018
10	6.89	0.045	18.41	0.018
11	6.84	0.043	18.48	0.018
12	6.79	0.042	18.53	0.018
13	6.78	0.041	18.55	0.018
14	6.78	0.040	18.60	0.018
15	6.77	0.041	18.70	0.018
16	6.78	0.043	18.73	0.018
17	6.76	0.043	18.81	0.018
18	6.72	0.045	18.88	0.018
19	6.69	0.048	18.93	0.019
20	6.66	0.049	18.98	0.018
21	6.65	0.052	19.08	0.018
22	6.60	0.053	19.18	0.019
23	6.57	0.055	19.21	0.021
24	6.50	0.056	19.24	0.024
25	6.48	0.059	19.26	0.022
26	6.29	0.056	19.36	0.022
27	6.27	0.057	19.40	0.023
28	6.14	0.057	19.49	0.024
29	6.12	0.057	19.55	0.025
30	6.10	0.059	19.45	0.022
31	6.06	0.061	19.58	0.025
32	6.01	0.061	19.68	0.026
33	6.03	0.063	19.76	0.025
34	6.00	0.064	19.76	0.025
35	5.97	0.066	19.82	0.027
36	5.93	0.066	19.84	0.027
37	5.89	0.066	19.92	0.028
38	5.86	0.067	19.88	0.027
39	5.84	0.068	19.90	0.028
40	5.81	0.065	19.82	0.033
41	5.80	0.064	19.39	0.023
42	5.78	0.065	19.41	0.026
43	5.78	0.066	19.46	0.025
44	5.75	0.066	18.90	0.021
45	5.74	0.068	18.92	0.023

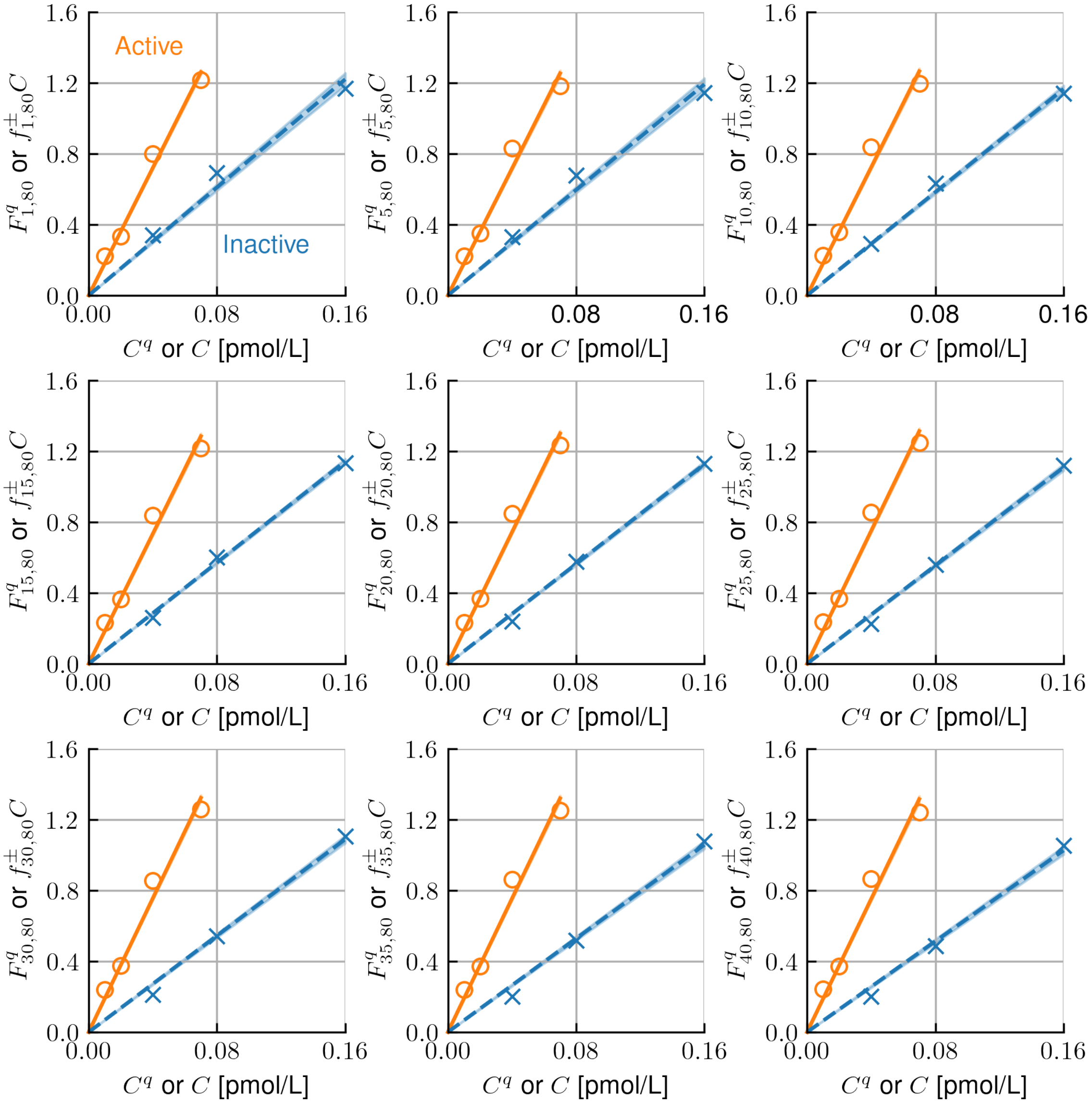

Fig. S80: As Figure S1 with well $w = 80$ (or G8).

Table S80: Molar Fluorescence Parameters for Well G8 ($w = 80$)

Cycle	Inactive		Active	
i	$f^-_{i,80}$	$\sigma^-_{i,80}$	$f^+_{i,80}$	$\sigma^+_{i,80}$
1	7.63	0.073	18.01	0.060
2	7.55	0.084	17.78	0.067
3	7.60	0.080	17.92	0.073
4	7.54	0.075	17.95	0.078
5	7.45	0.071	17.91	0.082
6	7.38	0.064	18.06	0.080
7	7.39	0.055	18.05	0.082
8	7.35	0.051	18.07	0.082
9	7.33	0.046	18.08	0.082
10	7.28	0.041	18.08	0.081
11	7.25	0.038	18.14	0.080
12	7.22	0.033	18.18	0.077
13	7.20	0.030	18.24	0.077
14	7.16	0.029	18.25	0.076
15	7.15	0.028	18.35	0.077
16	7.14	0.026	18.44	0.079
17	7.12	0.027	18.49	0.078
18	7.10	0.027	18.54	0.078
19	7.09	0.029	18.58	0.078
20	7.05	0.031	18.58	0.078
21	7.00	0.032	18.69	0.075
22	7.01	0.034	18.73	0.073
23	6.98	0.035	18.75	0.078
24	6.95	0.036	18.75	0.076
25	6.93	0.038	18.76	0.077
26	6.88	0.039	18.80	0.077
27	6.90	0.041	18.90	0.074
28	6.92	0.045	18.88	0.074
29	6.85	0.043	18.94	0.074
30	6.81	0.043	18.90	0.075
31	6.77	0.045	18.92	0.075
32	6.75	0.044	18.87	0.077
33	6.70	0.046	18.96	0.078
34	6.70	0.048	18.95	0.078
35	6.61	0.046	18.85	0.079
36	6.54	0.047	18.89	0.081
37	6.50	0.048	18.84	0.082
38	6.45	0.047	18.83	0.082
39	6.44	0.047	18.83	0.083
40	6.41	0.047	18.79	0.085
41	6.38	0.049	18.75	0.087
42	6.38	0.048	18.72	0.087
43	6.36	0.049	18.73	0.085
44	6.35	0.047	18.69	0.088
45	6.30	0.047	18.67	0.094

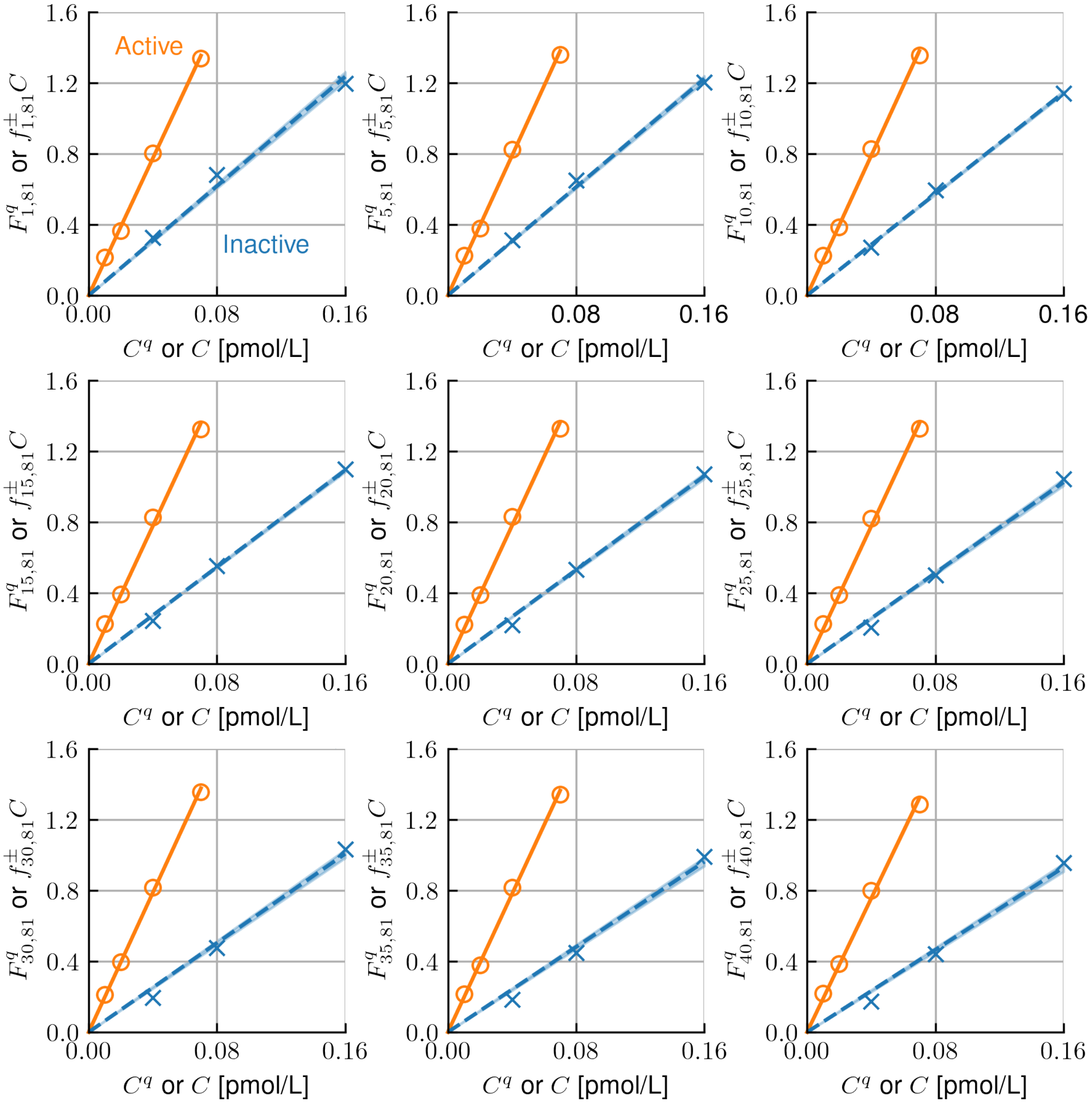

Fig. S81: As Figure S1 with well $w = 81$ (or G9).

Table S81: Molar Fluorescence Parameters for Well G9 ($w = 81$)

Cycle	Inactive		Active	
i	$f^-_{i,81}$	$\sigma^-_{i,81}$	$f^+_{i,81}$	$\sigma^+_{i,81}$
1	7.72	0.055	19.34	0.026
2	7.79	0.048	19.41	0.024
3	7.84	0.043	19.57	0.027
4	7.74	0.038	19.63	0.029
5	7.65	0.033	19.72	0.030
6	7.52	0.029	19.50	0.038
7	7.43	0.024	19.81	0.031
8	7.34	0.021	19.77	0.030
9	7.26	0.021	19.75	0.031
10	7.16	0.019	19.71	0.031
11	7.08	0.019	19.58	0.035
12	7.02	0.019	19.54	0.036
13	6.96	0.020	19.50	0.036
14	6.90	0.021	19.44	0.039
15	6.83	0.022	19.43	0.039
16	6.76	0.024	19.42	0.038
17	6.71	0.025	19.45	0.037
18	6.68	0.028	19.44	0.038
19	6.63	0.029	19.43	0.039
20	6.63	0.033	19.48	0.040
21	6.60	0.035	19.46	0.038
22	6.58	0.037	19.45	0.036
23	6.52	0.040	19.46	0.037
24	6.49	0.040	19.46	0.039
25	6.41	0.040	19.39	0.036
26	6.39	0.041	19.74	0.027
27	6.36	0.045	19.79	0.027
28	6.37	0.047	19.76	0.026
29	6.35	0.046	19.87	0.024
30	6.29	0.048	19.67	0.024
31	6.24	0.048	19.75	0.023
32	6.24	0.054	19.55	0.027
33	6.13	0.053	19.59	0.027
34	6.08	0.052	19.54	0.028
35	6.02	0.051	19.48	0.029
36	5.97	0.051	19.36	0.032
37	5.94	0.050	19.36	0.031
38	5.91	0.049	19.33	0.022
39	5.83	0.046	19.32	0.024
40	5.81	0.048	18.87	0.037
41	5.84	0.052	18.94	0.036
42	5.84	0.053	18.96	0.034
43	5.84	0.054	19.02	0.033
44	5.82	0.054	19.02	0.032
45	5.79	0.056	19.05	0.033

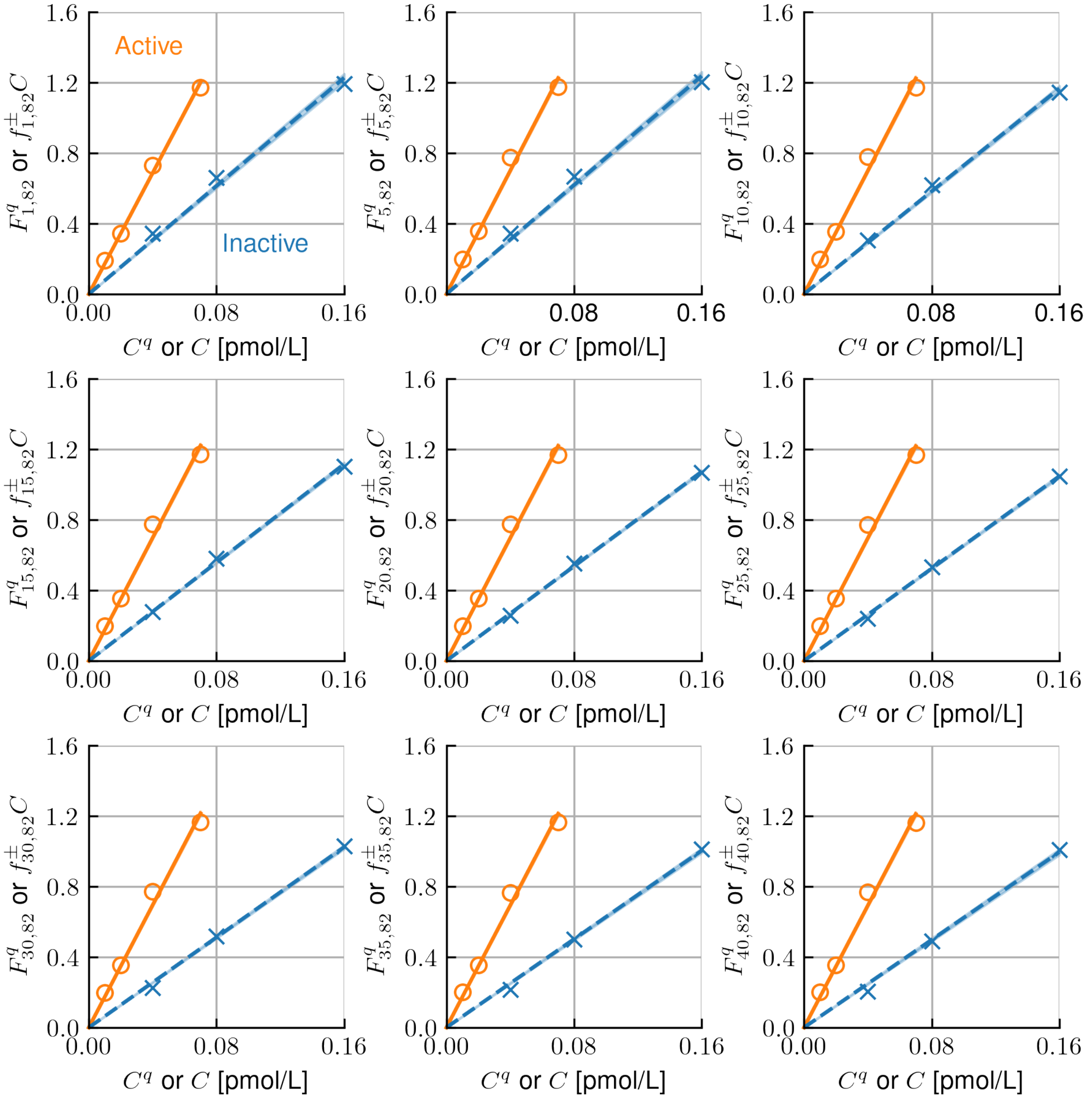

Fig. S82: As Figure S1 with well $w = 82$ (or G10).

Table S82: Molar Fluorescence Parameters for Well G10 ($w = 82$)

Cycle	Inactive		Active	
i	$f^-_{i,82}$	$\sigma^-_{i,82}$	$f^+_{i,82}$	$\sigma^+_{i,82}$
1	7.67	0.047	17.17	0.033
2	7.80	0.059	17.16	0.044
3	7.85	0.059	17.28	0.052
4	7.81	0.055	17.42	0.055
5	7.73	0.049	17.49	0.055
6	7.64	0.044	17.51	0.056
7	7.55	0.039	17.51	0.056
8	7.48	0.035	17.52	0.057
9	7.38	0.032	17.51	0.058
10	7.29	0.030	17.48	0.057
11	7.21	0.028	17.52	0.056
12	7.15	0.025	17.49	0.057
13	7.08	0.022	17.48	0.056
14	7.03	0.020	17.46	0.057
15	6.97	0.019	17.44	0.056
16	6.94	0.017	17.42	0.056
17	6.88	0.014	17.48	0.055
18	6.86	0.013	17.46	0.056
19	6.77	0.014	17.50	0.053
20	6.71	0.014	17.41	0.055
21	6.69	0.014	17.39	0.056
22	6.64	0.014	17.42	0.055
23	6.60	0.014	17.41	0.055
24	6.58	0.015	17.41	0.056
25	6.55	0.016	17.40	0.055
26	6.54	0.017	17.36	0.055
27	6.46	0.016	17.52	0.054
28	6.45	0.019	17.47	0.059
29	6.42	0.020	17.41	0.059
30	6.40	0.022	17.36	0.056
31	6.37	0.022	17.35	0.055
32	6.35	0.023	17.32	0.053
33	6.33	0.024	17.33	0.052
34	6.32	0.026	17.33	0.052
35	6.28	0.026	17.32	0.053
36	6.25	0.028	17.35	0.052
37	6.22	0.028	17.32	0.053
38	6.23	0.029	17.34	0.053
39	6.30	0.036	17.33	0.053
40	6.22	0.033	17.30	0.055
41	6.25	0.037	17.30	0.055
42	6.16	0.032	17.34	0.055
43	6.08	0.032	17.32	0.054
44	6.04	0.030	17.36	0.055
45	5.99	0.027	17.35	0.055

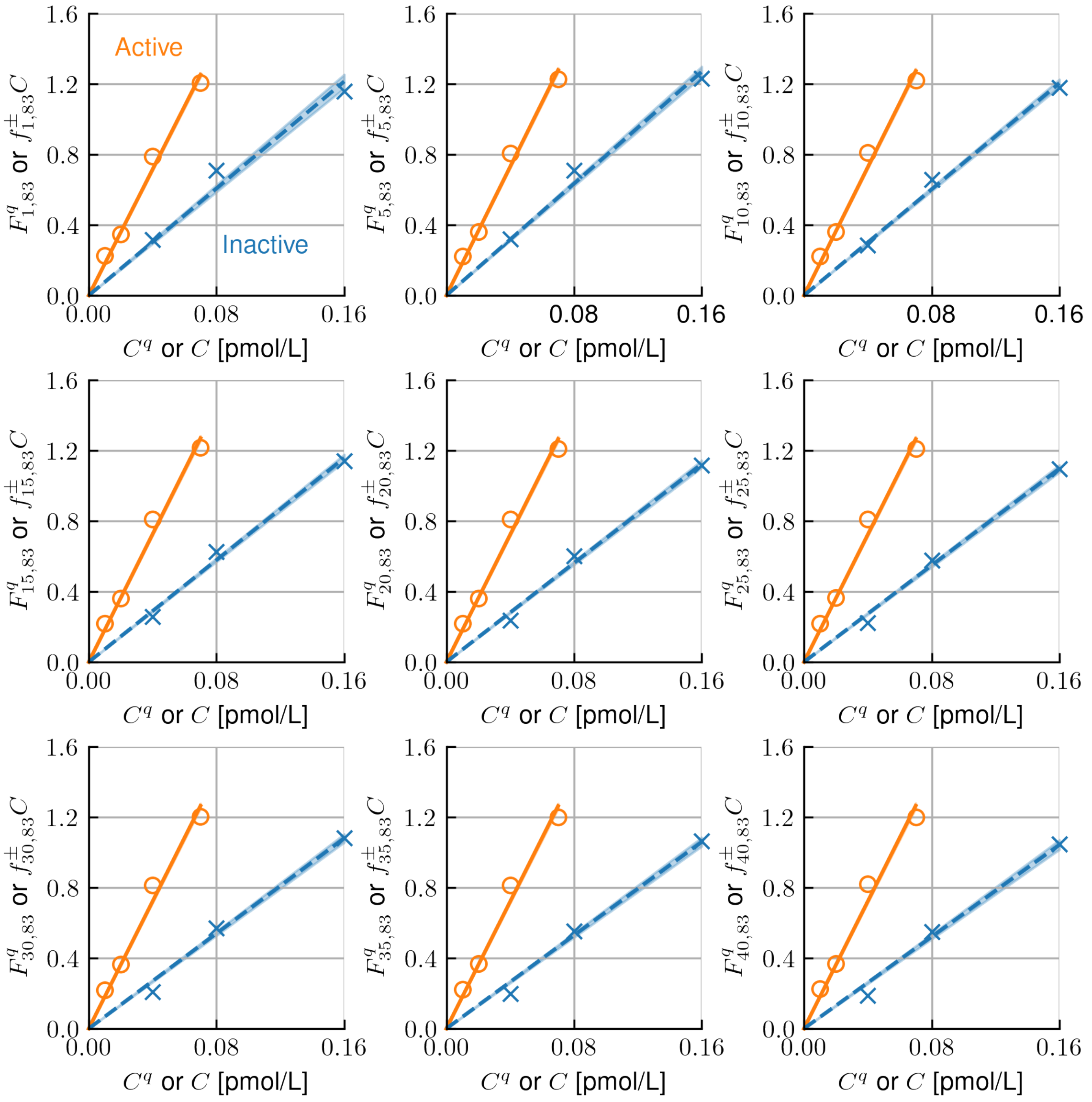

Fig. S83: As Figure S1 with well $w = 83$ (or G11).

Table S83: Molar Fluorescence Parameters for Well G11 ($w = 83$)

Cycle	Inactive		Active	
i	$f^-_{i,83}$	$\sigma^-_{i,83}$	$f^+_{i,83}$	$\sigma^+_{i,83}$
1	7.59	0.082	17.88	0.058
2	7.91	0.071	18.05	0.054
3	8.11	0.061	18.13	0.057
4	8.01	0.063	18.21	0.057
5	7.93	0.060	18.27	0.058
6	7.83	0.055	18.27	0.059
7	7.74	0.052	18.26	0.060
8	7.67	0.048	18.23	0.059
9	7.61	0.046	18.23	0.059
10	7.53	0.044	18.20	0.061
11	7.45	0.044	18.21	0.061
12	7.40	0.042	18.19	0.061
13	7.34	0.042	18.17	0.062
14	7.29	0.041	18.11	0.063
15	7.23	0.041	18.14	0.062
16	7.18	0.042	18.11	0.061
17	7.15	0.041	18.08	0.062
18	7.11	0.041	18.08	0.064
19	7.08	0.041	18.08	0.065
20	7.02	0.042	18.08	0.064
21	6.99	0.043	18.06	0.064
22	6.97	0.043	18.06	0.064
23	6.93	0.044	18.08	0.064
24	6.89	0.044	18.08	0.065
25	6.86	0.042	18.07	0.063
26	6.84	0.046	18.12	0.062
27	6.81	0.046	18.10	0.063
28	6.78	0.046	18.07	0.064
29	6.74	0.047	18.07	0.065
30	6.75	0.048	18.05	0.066
31	6.71	0.048	18.04	0.067
32	6.70	0.048	18.05	0.067
33	6.67	0.048	18.05	0.068
34	6.66	0.050	18.05	0.069
35	6.62	0.051	18.04	0.069
36	6.60	0.053	17.99	0.070
37	6.61	0.053	18.03	0.069
38	6.59	0.053	18.05	0.069
39	6.56	0.054	18.06	0.071
40	6.52	0.056	18.06	0.072
41	6.48	0.057	18.15	0.081
42	6.47	0.057	18.07	0.075
43	6.45	0.058	18.07	0.074
44	6.42	0.058	18.06	0.075
45	6.43	0.057	18.07	0.074

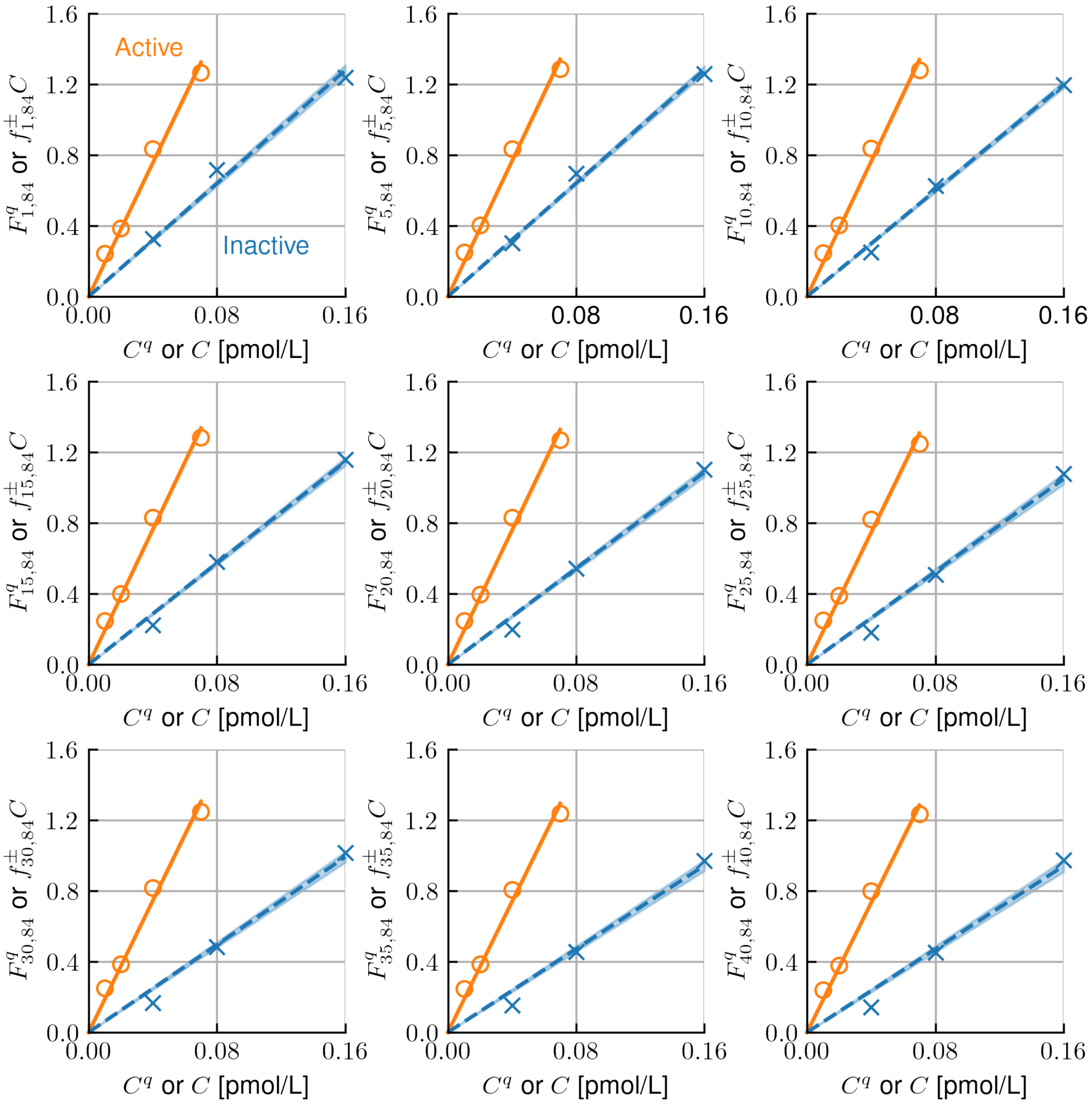

Fig. S84: As Figure S1 with well $w = 84$ (or G12).

Table S84: Molar Fluorescence Parameters for Well G12 ($w = 84$)

Cycle	Inactive		Active	
i	$f^-_{i,84}$	$\sigma^-_{i,84}$	$f^+_{i,84}$	$\sigma^+_{i,84}$
1	7.99	0.062	18.90	0.064
2	8.23	0.058	18.87	0.060
3	8.24	0.054	18.98	0.063
4	8.12	0.048	19.05	0.063
5	8.01	0.043	19.14	0.062
6	7.87	0.039	19.16	0.061
7	7.77	0.037	19.18	0.061
8	7.66	0.036	19.16	0.062
9	7.59	0.038	19.15	0.063
10	7.48	0.039	19.10	0.063
11	7.40	0.042	19.13	0.061
12	7.33	0.044	19.07	0.063
13	7.28	0.046	19.08	0.063
14	7.21	0.046	19.10	0.061
15	7.16	0.046	19.07	0.060
16	7.08	0.045	19.02	0.061
17	7.01	0.048	19.08	0.062
18	6.98	0.053	19.09	0.060
19	6.86	0.052	19.06	0.059
20	6.78	0.052	18.95	0.065
21	6.71	0.054	18.98	0.061
22	6.66	0.055	18.91	0.062
23	6.60	0.056	18.85	0.068
24	6.53	0.057	18.70	0.066
25	6.55	0.063	18.64	0.066
26	6.48	0.063	18.62	0.066
27	6.40	0.066	18.62	0.066
28	6.34	0.063	18.61	0.066
29	6.25	0.062	18.60	0.068
30	6.19	0.062	18.62	0.064
31	6.13	0.062	18.56	0.064
32	6.06	0.063	18.56	0.066
33	5.99	0.063	18.53	0.064
34	5.92	0.062	18.50	0.063
35	5.89	0.063	18.46	0.063
36	5.91	0.066	18.40	0.066
37	5.90	0.066	18.41	0.064
38	5.91	0.068	18.33	0.065
39	5.90	0.068	18.33	0.063
40	5.88	0.070	18.32	0.058
41	5.86	0.070	18.14	0.068
42	5.88	0.073	18.13	0.067
43	5.85	0.075	18.16	0.064
44	5.83	0.074	18.06	0.063
45	5.81	0.075	18.09	0.063

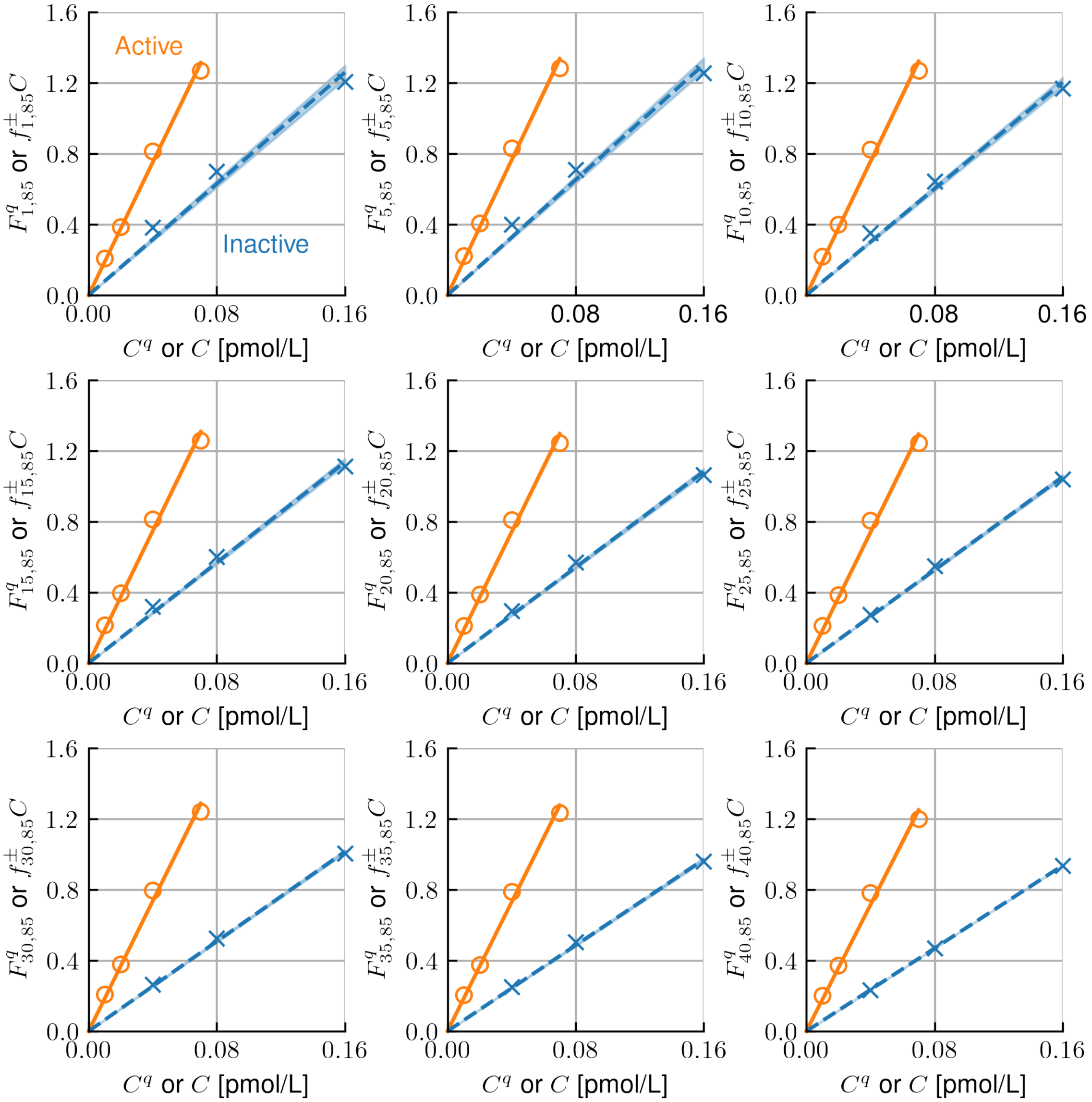

Fig. S85: As Figure S1 with well $w = 85$ (or H1).

Table S85: Molar Fluorescence Parameters for Well H1 ($w = 85$)

Cycle	Inactive		Active	
i	$f^-_{i,85}$	$\sigma^-_{i,85}$	$f^+_{i,85}$	$\sigma^+_{i,85}$
1	7.87	0.079	18.75	0.046
2	8.28	0.076	18.98	0.048
3	8.36	0.078	19.04	0.052
4	8.29	0.078	19.05	0.054
5	8.15	0.074	19.07	0.054
6	8.00	0.070	19.00	0.054
7	7.87	0.065	19.00	0.054
8	7.75	0.060	18.91	0.055
9	7.63	0.057	18.87	0.055
10	7.52	0.054	18.87	0.054
11	7.42	0.049	18.88	0.051
12	7.35	0.046	18.77	0.053
13	7.27	0.042	18.76	0.053
14	7.19	0.039	18.73	0.052
15	7.11	0.038	18.67	0.053
16	7.02	0.036	18.66	0.052
17	6.96	0.035	18.63	0.051
18	6.90	0.031	18.66	0.051
19	6.87	0.028	18.54	0.052
20	6.77	0.029	18.51	0.052
21	6.72	0.027	18.52	0.051
22	6.68	0.025	18.49	0.052
23	6.64	0.023	18.50	0.050
24	6.57	0.023	18.57	0.056
25	6.58	0.020	18.47	0.052
26	6.49	0.020	18.47	0.053
27	6.46	0.015	18.44	0.049
28	6.42	0.015	18.46	0.049
29	6.41	0.012	18.42	0.047
30	6.34	0.016	18.37	0.047
31	6.28	0.019	18.38	0.045
32	6.18	0.020	18.34	0.047
33	6.14	0.019	18.28	0.046
34	6.10	0.018	18.24	0.046
35	6.07	0.017	18.23	0.045
36	5.995	0.0086	18.23	0.044
37	5.960	0.0062	18.26	0.042
38	5.928	0.0025	17.81	0.056
39	5.883	0.0020	17.87	0.053
40	5.8526	0.00033	17.84	0.051
41	5.829	0.0017	17.85	0.050
42	5.787	0.0027	17.85	0.049
43	5.751	0.0035	17.83	0.049
44	5.726	0.0047	17.84	0.048
45	5.698	0.0068	17.81	0.048

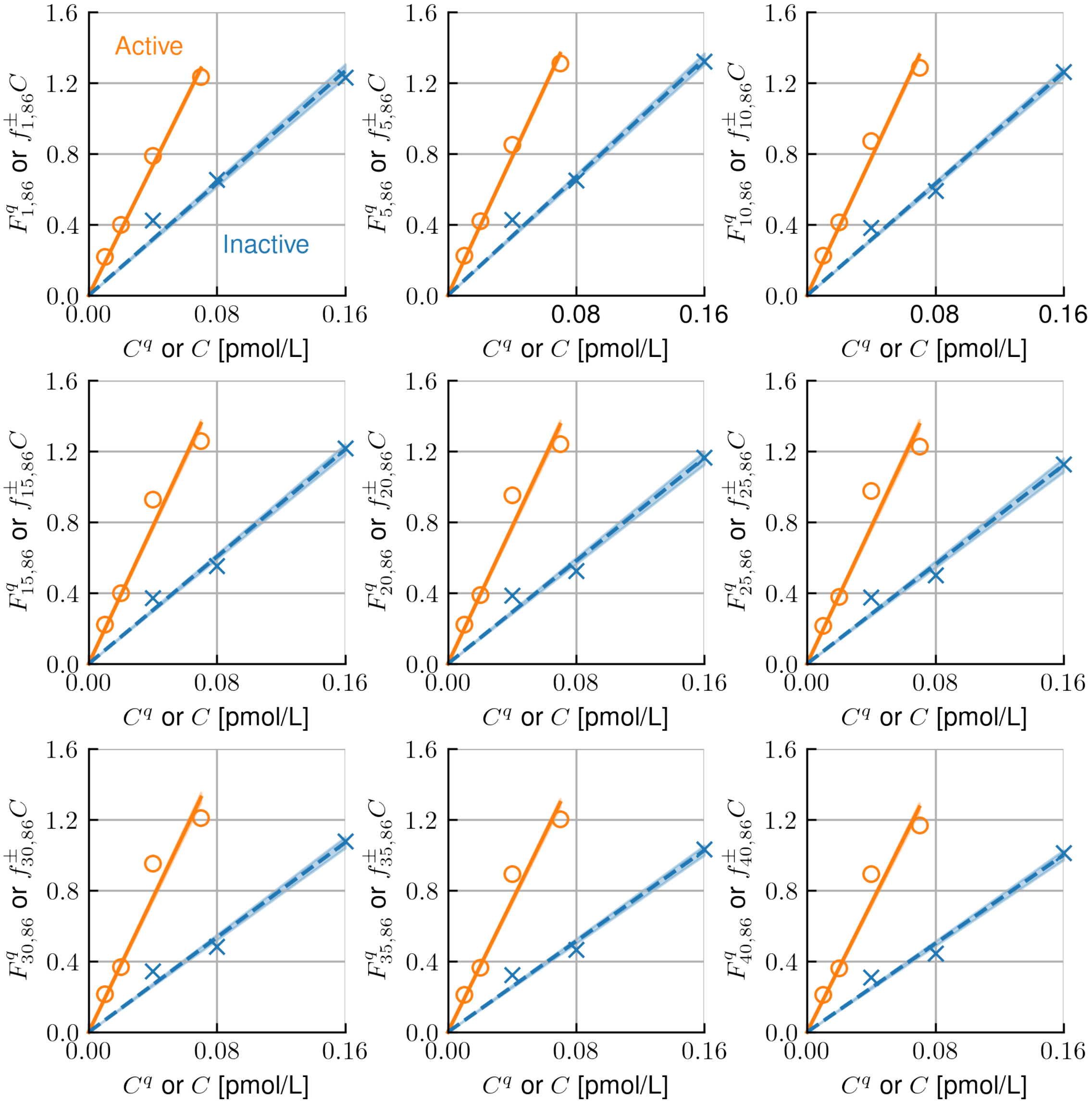

Fig. S86: As Figure S1 with well $w = 86$ (or H2).

Table S86: Molar Fluorescence Parameters for Well H2 ($w = 86$)

Cycle	Inactive		Active	
i	$f^-_{i,86}$	$\sigma^-_{i,86}$	$f^+_{i,86}$	$\sigma^+_{i,86}$
1	7.92	0.081	18.32	0.053
2	8.40	0.076	19.10	0.046
3	8.52	0.075	19.33	0.053
4	8.45	0.072	19.45	0.056
5	8.35	0.068	19.49	0.058
6	8.23	0.065	19.49	0.060
7	8.15	0.062	19.47	0.062
8	8.06	0.058	19.43	0.065
9	7.95	0.057	19.39	0.069
10	7.87	0.056	19.37	0.073
11	7.79	0.056	19.30	0.077
12	7.72	0.056	19.25	0.080
13	7.66	0.056	19.31	0.085
14	7.61	0.059	19.30	0.092
15	7.56	0.062	19.4	0.11
16	7.50	0.065	19.4	0.12
17	7.43	0.069	19.4	0.12
18	7.46	0.074	19.4	0.12
19	7.33	0.075	19.4	0.12
20	7.26	0.077	19.3	0.12
21	7.22	0.079	19.2	0.12
22	7.18	0.081	19.2	0.12
23	7.09	0.082	19.1	0.12
24	7.05	0.082	19.2	0.12
25	7.01	0.081	19.3	0.14
26	6.92	0.078	19.2	0.14
27	6.86	0.076	19.2	0.14
28	6.80	0.072	19.1	0.13
29	6.75	0.067	19.1	0.13
30	6.69	0.065	18.9	0.13
31	6.67	0.059	18.9	0.13
32	6.62	0.061	18.8	0.12
33	6.52	0.060	18.7	0.12
34	6.48	0.059	18.6	0.12
35	6.42	0.058	18.5	0.10
36	6.39	0.057	18.5	0.11
37	6.34	0.058	18.5	0.11
38	6.32	0.059	18.5	0.10
39	6.28	0.058	18.6	0.10
40	6.25	0.058	18.1	0.12
41	6.22	0.060	18.2	0.11
42	6.19	0.059	18.1	0.10
43	6.14	0.061	18.1	0.11
44	6.12	0.060	18.1	0.10
45	6.10	0.058	18.00	0.098

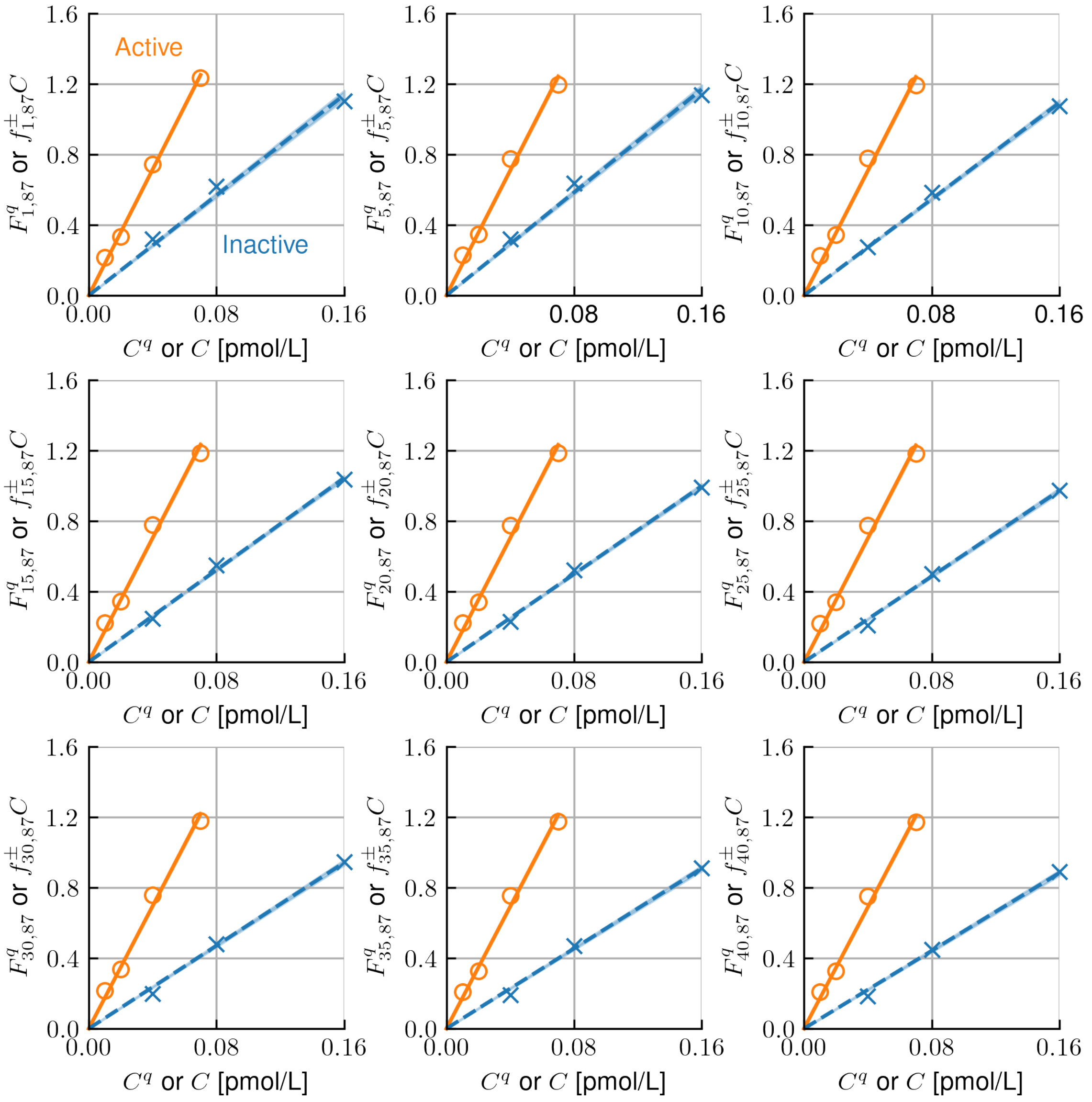

Fig. S87: As Figure S1 with well $w = 87$ (or H3).

Table S87: Molar Fluorescence Parameters for Well H3 ($w = 87$)

Cycle	Inactive		Active	
i	$f^-_{i,87}$	$\sigma^-_{i,87}$	$f^+_{i,87}$	$\sigma^+_{i,87}$
1	7.10	0.050	17.87	0.033
2	7.45	0.053	17.65	0.041
3	7.53	0.051	17.72	0.048
4	7.43	0.050	17.72	0.052
5	7.31	0.048	17.73	0.055
6	7.20	0.044	17.74	0.056
7	7.10	0.040	17.71	0.057
8	7.02	0.034	17.70	0.058
9	6.92	0.032	17.68	0.057
10	6.83	0.030	17.70	0.057
11	6.77	0.028	17.68	0.057
12	6.70	0.026	17.64	0.058
13	6.63	0.024	17.66	0.060
14	6.58	0.023	17.64	0.058
15	6.53	0.022	17.61	0.058
16	6.50	0.020	17.62	0.057
17	6.42	0.021	17.63	0.056
18	6.37	0.021	17.61	0.056
19	6.31	0.021	17.63	0.055
20	6.24	0.022	17.59	0.056
21	6.25	0.021	17.54	0.057
22	6.19	0.021	17.55	0.057
23	6.15	0.025	17.54	0.057
24	6.11	0.025	17.52	0.056
25	6.08	0.026	17.55	0.057
26	6.05	0.027	17.52	0.056
27	6.00	0.026	17.52	0.059
28	6.00	0.029	17.48	0.058
29	5.93	0.027	17.46	0.054
30	5.89	0.028	17.39	0.051
31	5.84	0.026	17.38	0.050
32	5.81	0.027	17.37	0.050
33	5.81	0.028	17.46	0.047
34	5.70	0.027	17.37	0.048
35	5.68	0.028	17.32	0.048
36	5.64	0.027	17.30	0.048
37	5.61	0.027	17.28	0.047
38	5.59	0.028	17.28	0.047
39	5.56	0.028	17.28	0.047
40	5.52	0.027	17.23	0.048
41	5.51	0.028	16.44	0.070
42	5.48	0.029	16.52	0.066
43	5.46	0.029	16.67	0.061
44	5.45	0.029	16.72	0.059
45	5.43	0.034	16.77	0.059

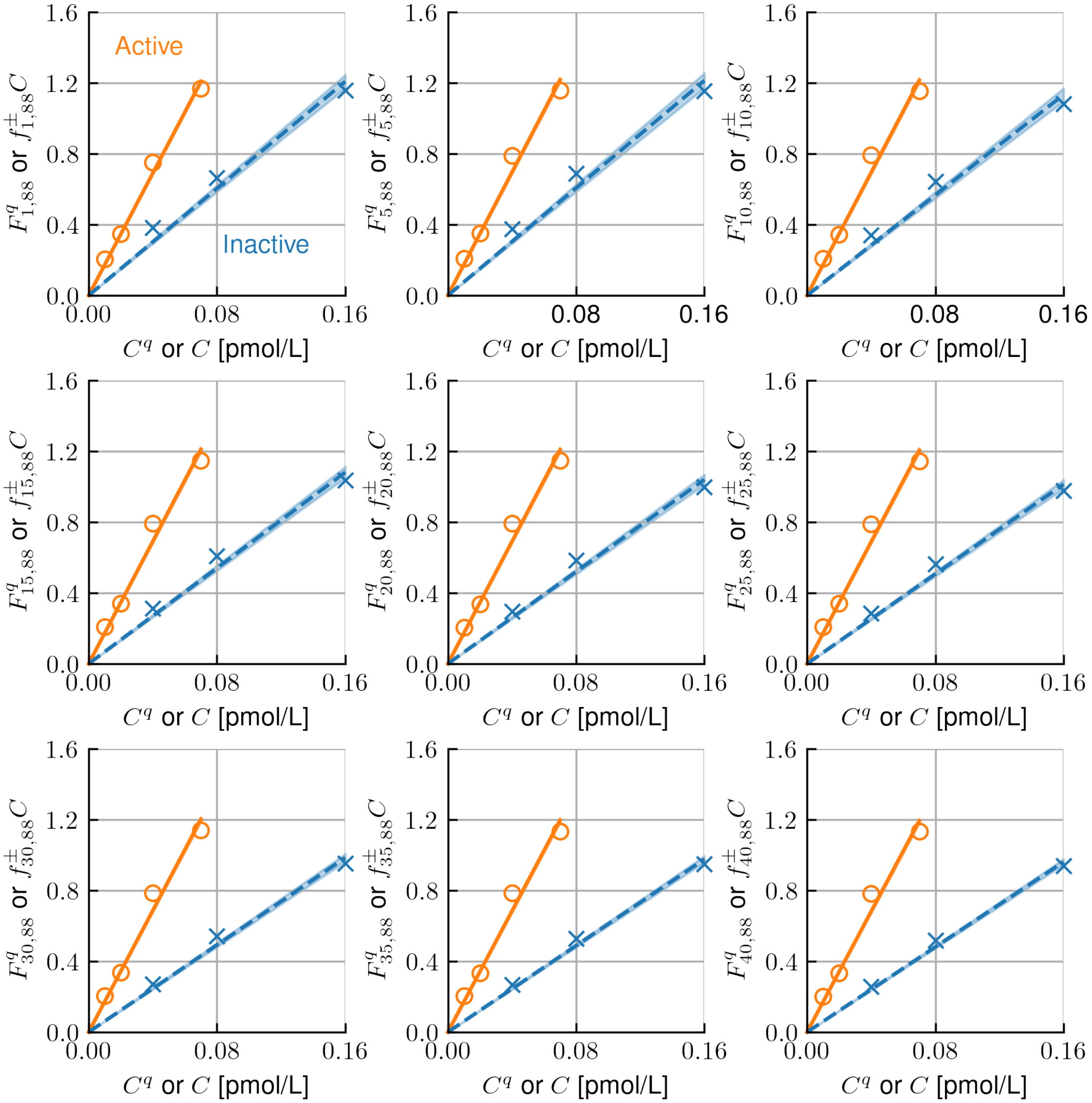

Fig. S88: As Figure S1 with well $w = 88$ (or H4).

Table S88: Molar Fluorescence Parameters for Well H4 ($w = 88$)

Cycle	Inactive		Active	
i	$f^-_{i,88}$	$\sigma^-_{i,88}$	$f^+_{i,88}$	$\sigma^+_{i,88}$
1	7.55	0.079	17.25	0.046
2	7.74	0.087	17.14	0.056
3	7.76	0.088	17.22	0.063
4	7.69	0.090	17.32	0.066
5	7.58	0.089	17.38	0.067
6	7.47	0.088	17.37	0.068
7	7.38	0.085	17.36	0.070
8	7.28	0.082	17.34	0.069
9	7.18	0.080	17.36	0.070
10	7.08	0.077	17.35	0.070
11	7.01	0.073	17.34	0.069
12	6.95	0.071	17.35	0.071
13	6.87	0.068	17.32	0.071
14	6.82	0.067	17.28	0.071
15	6.76	0.064	17.28	0.072
16	6.70	0.062	17.27	0.073
17	6.66	0.060	17.28	0.070
18	6.61	0.059	17.26	0.071
19	6.57	0.058	17.23	0.071
20	6.50	0.059	17.26	0.071
21	6.46	0.058	17.23	0.071
22	6.44	0.055	17.23	0.070
23	6.39	0.055	17.26	0.071
24	6.37	0.054	17.23	0.070
25	6.33	0.053	17.21	0.071
26	6.30	0.051	17.21	0.070
27	6.28	0.048	17.40	0.064
28	6.26	0.045	17.22	0.069
29	6.27	0.041	17.13	0.071
30	6.15	0.046	17.17	0.070
31	6.25	0.037	17.06	0.068
32	6.18	0.038	17.12	0.074
33	6.11	0.040	17.19	0.071
34	6.11	0.038	17.16	0.074
35	6.10	0.037	17.09	0.072
36	6.10	0.034	17.12	0.071
37	6.08	0.038	17.22	0.067
38	6.05	0.035	17.09	0.070
39	6.04	0.034	17.20	0.067
40	6.01	0.032	17.03	0.070
41	5.98	0.033	16.97	0.071
42	5.96	0.029	16.92	0.072
43	5.93	0.027	16.93	0.068
44	5.88	0.027	16.88	0.070
45	5.85	0.028	16.84	0.072

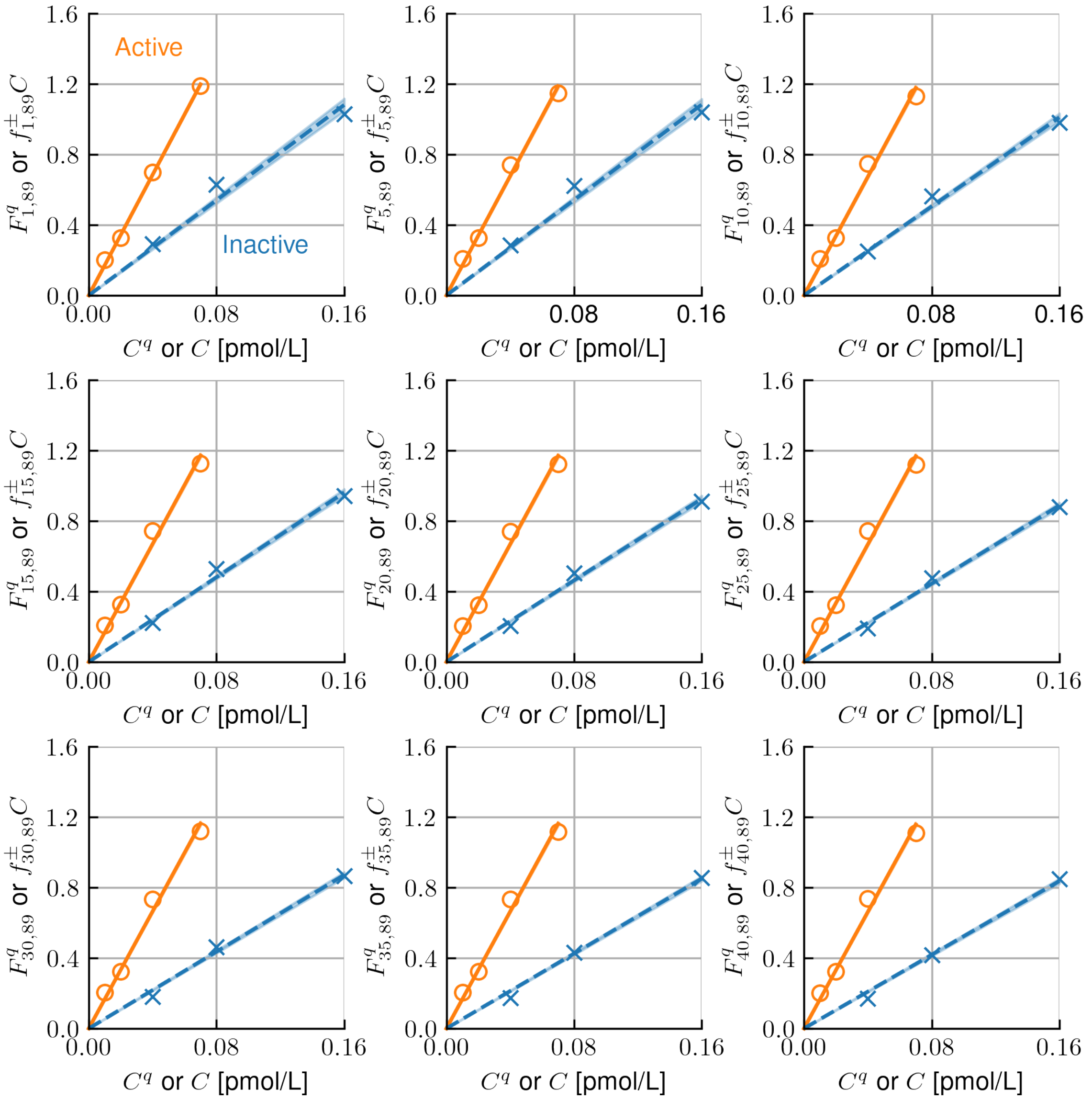

Fig. S89: As Figure S1 with well $w = 89$ (or H5).

Table S89: Molar Fluorescence Parameters for Well H5 ($w = 89$)

Cycle	Inactive		Active	
i	$f^-_{i,89}$	$\sigma^-_{i,89}$	$f^+_{i,89}$	$\sigma^+_{i,89}$
1	6.76	0.074	17.11	0.022
2	6.99	0.068	16.87	0.035
3	7.01	0.068	16.95	0.043
4	6.89	0.068	16.95	0.047
5	6.77	0.065	16.95	0.049
6	6.67	0.060	16.87	0.053
7	6.57	0.056	16.86	0.054
8	6.49	0.052	16.83	0.054
9	6.40	0.049	16.82	0.055
10	6.31	0.047	16.82	0.055
11	6.24	0.046	16.81	0.055
12	6.16	0.044	16.76	0.055
13	6.11	0.042	16.75	0.056
14	6.06	0.040	16.74	0.056
15	6.01	0.039	16.73	0.055
16	5.97	0.039	16.73	0.055
17	5.92	0.037	16.73	0.054
18	5.86	0.037	16.71	0.055
19	5.81	0.036	16.70	0.054
20	5.78	0.037	16.71	0.055
21	5.76	0.035	16.69	0.055
22	5.70	0.034	16.66	0.054
23	5.66	0.032	16.81	0.053
24	5.61	0.032	16.71	0.056
25	5.55	0.032	16.68	0.057
26	5.57	0.030	16.66	0.056
27	5.53	0.030	16.67	0.054
28	5.47	0.030	16.67	0.055
29	5.46	0.030	16.68	0.052
30	5.45	0.033	16.62	0.053
31	5.39	0.029	16.71	0.050
32	5.35	0.028	16.65	0.052
33	5.31	0.029	16.60	0.053
34	5.29	0.028	16.59	0.053
35	5.32	0.028	16.60	0.054
36	5.28	0.028	16.55	0.054
37	5.25	0.028	16.55	0.054
38	5.26	0.027	16.68	0.055
39	5.23	0.030	16.57	0.055
40	5.23	0.029	16.53	0.055
41	5.21	0.029	16.54	0.056
42	5.16	0.029	16.53	0.054
43	5.20	0.032	16.52	0.054
44	5.15	0.031	16.50	0.054
45	5.15	0.032	16.56	0.058

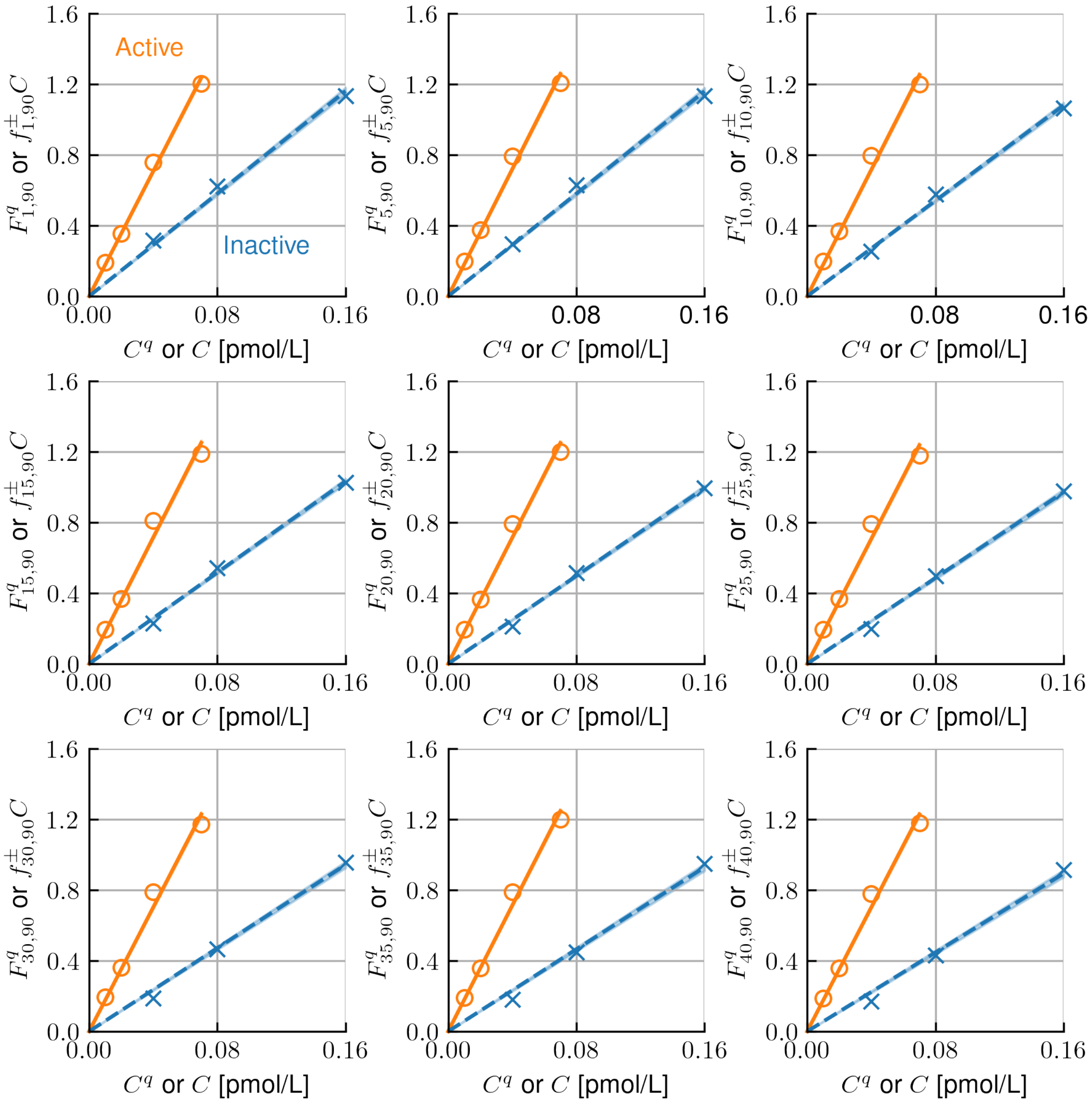

Fig. S90: As Figure S1 with well $w = 90$ (or H6).

Table S90: Molar Fluorescence Parameters for Well H6 ($w = 90$)

Cycle	Inactive		Active	
i	$f^-_{i,90}$	$\sigma^-_{i,90}$	$f^+_{i,90}$	$\sigma^+_{i,90}$
1	7.26	0.040	17.67	0.036
2	7.45	0.038	17.70	0.045
3	7.51	0.038	17.87	0.049
4	7.40	0.040	17.93	0.052
5	7.25	0.040	17.97	0.053
6	7.12	0.039	17.97	0.053
7	7.02	0.035	17.93	0.056
8	6.93	0.033	17.90	0.057
9	6.84	0.033	17.94	0.059
10	6.75	0.031	17.89	0.057
11	6.70	0.030	17.95	0.056
12	6.62	0.029	17.84	0.059
13	6.57	0.030	17.83	0.059
14	6.50	0.029	17.83	0.061
15	6.46	0.028	17.87	0.066
16	6.41	0.030	17.80	0.062
17	6.37	0.029	17.82	0.059
18	6.31	0.029	17.74	0.060
19	6.28	0.029	17.77	0.059
20	6.22	0.029	17.85	0.056
21	6.19	0.030	17.78	0.058
22	6.15	0.030	17.85	0.055
23	6.12	0.031	17.77	0.058
24	6.09	0.032	17.71	0.058
25	6.07	0.034	17.67	0.061
26	6.03	0.034	17.65	0.062
27	6.00	0.034	17.85	0.053
28	5.97	0.036	17.66	0.060
29	5.96	0.037	17.61	0.061
30	5.89	0.037	17.55	0.061
31	5.91	0.039	17.55	0.062
32	5.86	0.038	17.55	0.060
33	5.85	0.040	17.56	0.060
34	5.82	0.040	17.51	0.058
35	5.81	0.041	17.81	0.053
36	5.81	0.044	17.57	0.055
37	5.79	0.047	17.50	0.055
38	5.75	0.046	17.47	0.056
39	5.67	0.044	17.47	0.057
40	5.58	0.041	17.54	0.053
41	5.58	0.044	17.42	0.056
42	5.56	0.045	17.43	0.056
43	5.53	0.044	17.50	0.054
44	5.50	0.045	17.41	0.055
45	5.52	0.046	17.43	0.056

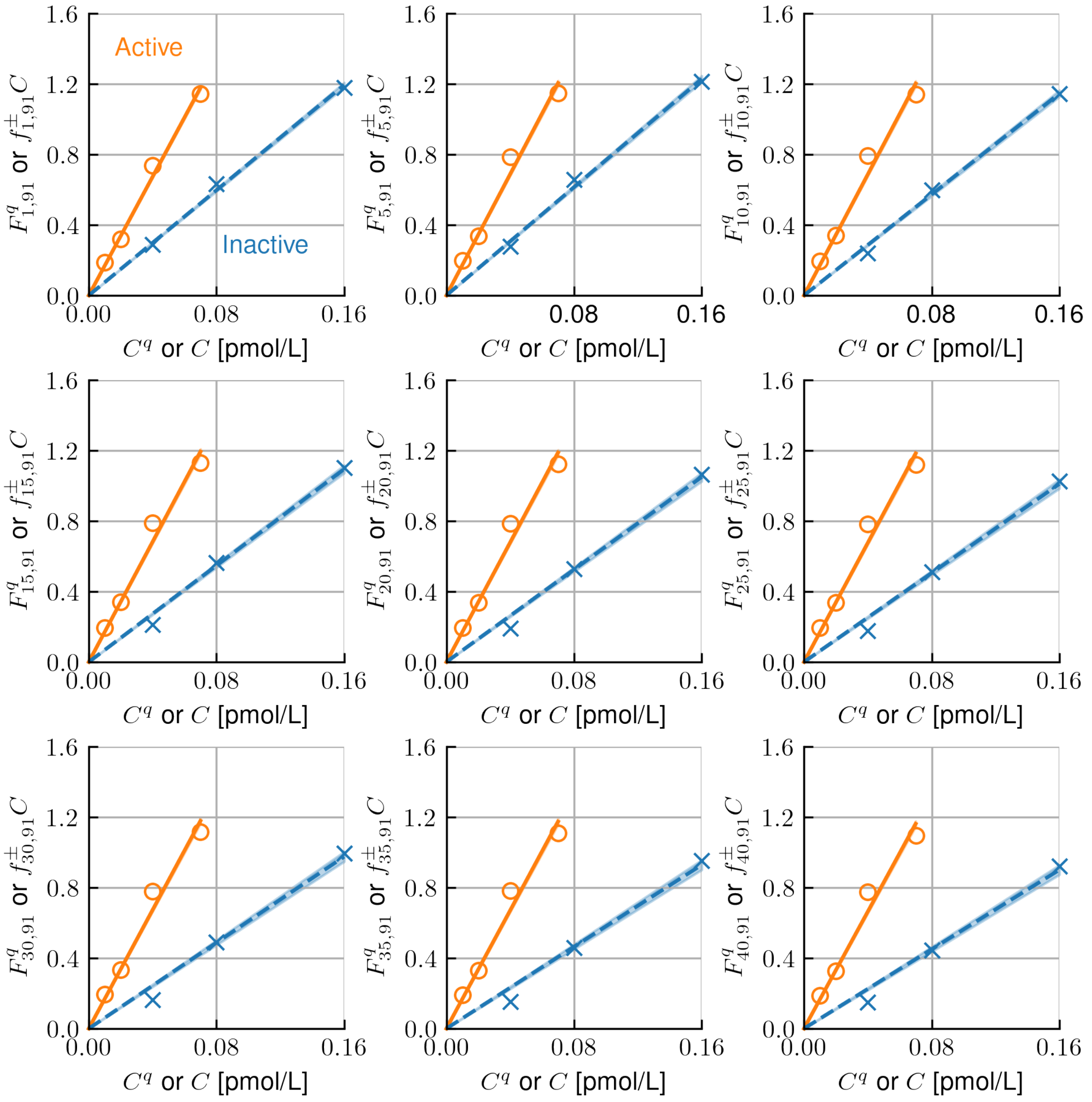

Fig. S91: As Figure S1 with well $w = 91$ (or H7).

Table S91: Molar Fluorescence Parameters for Well H7 ($w = 91$)

Cycle	Inactive		Active	
i	$f^-_{i,91}$	$\sigma^-_{i,91}$	$f^+_{i,91}$	$\sigma^+_{i,91}$
1	7.46	0.028	16.84	0.044
2	7.84	0.032	17.01	0.058
3	7.91	0.034	17.14	0.062
4	7.82	0.035	17.19	0.065
5	7.68	0.036	17.22	0.067
6	7.56	0.036	17.20	0.069
7	7.46	0.036	17.21	0.069
8	7.34	0.036	17.16	0.070
9	7.25	0.037	17.16	0.072
10	7.16	0.038	17.17	0.072
11	7.08	0.040	17.16	0.071
12	7.01	0.041	17.13	0.071
13	6.95	0.042	17.09	0.072
14	6.89	0.044	17.09	0.072
15	6.84	0.046	17.05	0.073
16	6.78	0.047	17.05	0.072
17	6.71	0.047	17.08	0.071
18	6.66	0.048	17.03	0.072
19	6.63	0.049	17.01	0.071
20	6.56	0.051	16.98	0.073
21	6.54	0.053	17.01	0.072
22	6.46	0.053	16.98	0.073
23	6.41	0.054	16.95	0.074
24	6.38	0.054	16.93	0.074
25	6.32	0.054	16.91	0.074
26	6.28	0.056	16.90	0.074
27	6.23	0.053	16.92	0.073
28	6.17	0.056	16.93	0.073
29	6.16	0.059	16.96	0.071
30	6.10	0.058	16.84	0.073
31	6.13	0.062	16.91	0.073
32	6.05	0.061	16.83	0.074
33	5.95	0.061	16.76	0.075
34	5.88	0.059	16.86	0.073
35	5.81	0.058	16.78	0.077
36	5.77	0.059	16.75	0.076
37	5.73	0.060	16.81	0.077
38	5.69	0.058	16.76	0.076
39	5.67	0.058	16.66	0.076
40	5.63	0.057	16.60	0.076
41	5.60	0.057	16.56	0.075
42	5.55	0.057	16.57	0.078
43	5.52	0.058	16.49	0.076
44	5.49	0.058	16.46	0.075
45	5.59	0.064	16.37	0.073

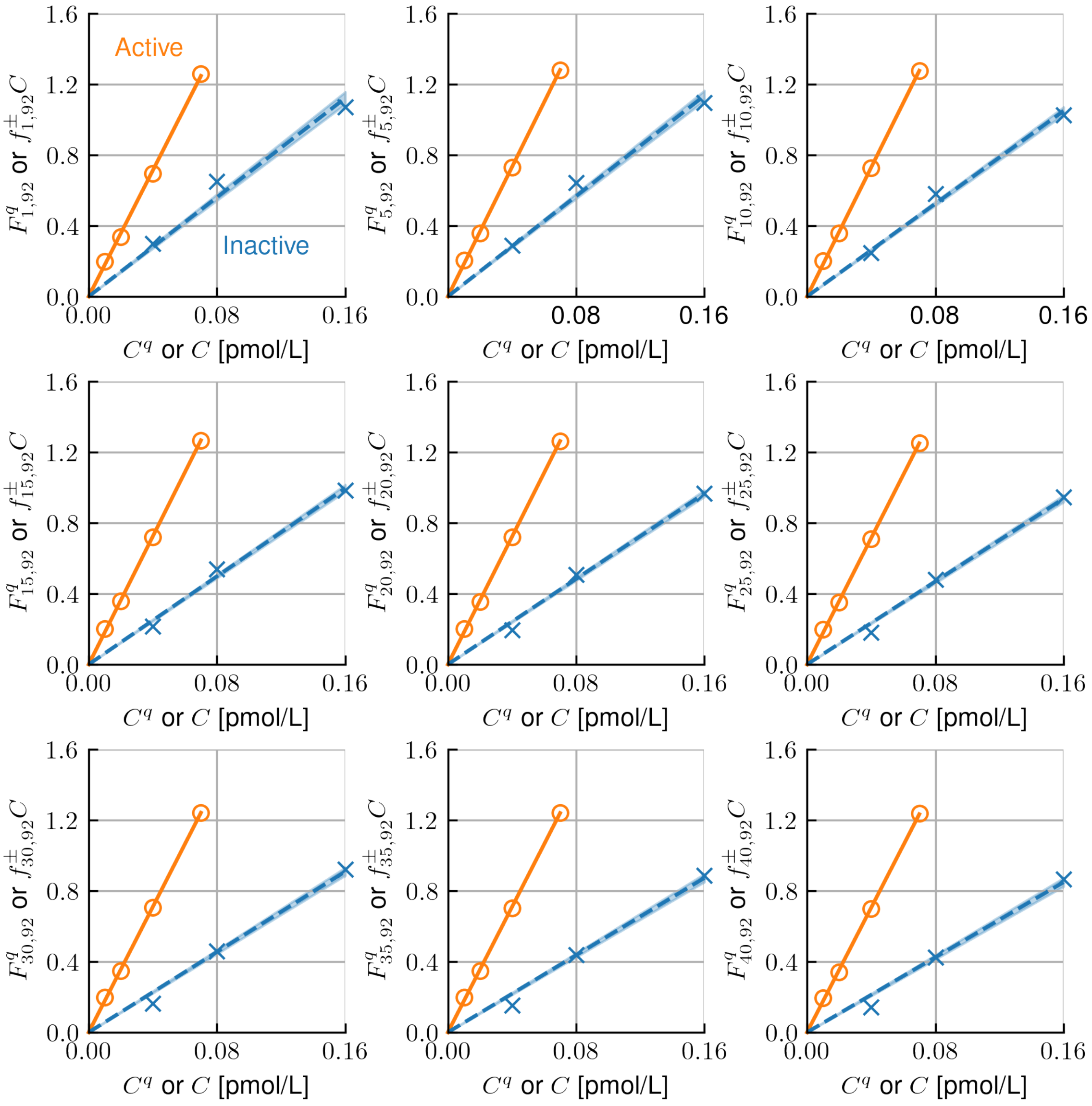

Fig. S92: As Figure S1 with well $w = 92$ (or H8).

Table S92: Molar Fluorescence Parameters for Well H8 ($w = 92$)

Cycle	Inactive		Active	
i	$f^-_{i,92}$	$\sigma^-_{i,92}$	$f^+_{i,92}$	$\sigma^+_{i,92}$
1	7.01	0.072	17.82	0.020
2	7.34	0.055	18.03	0.016
3	7.40	0.055	18.22	0.015
4	7.23	0.061	18.26	0.014
5	7.10	0.060	18.30	0.014
6	6.95	0.057	18.28	0.014
7	6.84	0.054	18.27	0.013
8	6.75	0.050	18.26	0.013
9	6.65	0.046	18.23	0.012
10	6.56	0.044	18.24	0.012
11	6.49	0.043	18.21	0.012
12	6.41	0.042	18.18	0.012
13	6.35	0.041	18.19	0.012
14	6.29	0.039	18.12	0.011
15	6.23	0.039	18.10	0.012
16	6.18	0.039	18.10	0.012
17	6.18	0.037	18.07	0.011
18	6.13	0.036	18.06	0.012
19	6.08	0.036	18.28	0.014
20	6.04	0.037	18.04	0.013
21	6.02	0.038	18.01	0.012
22	5.97	0.038	17.96	0.012
23	5.93	0.039	17.95	0.012
24	5.89	0.039	17.96	0.012
25	5.86	0.039	17.86	0.012
26	5.80	0.040	17.84	0.012
27	5.75	0.039	17.85	0.012
28	5.74	0.041	17.78	0.013
29	5.73	0.043	17.75	0.012
30	5.68	0.045	17.73	0.012
31	5.60	0.046	17.75	0.012
32	5.57	0.044	17.75	0.012
33	5.53	0.046	17.73	0.013
34	5.51	0.048	17.71	0.013
35	5.45	0.047	17.71	0.013
36	5.42	0.047	17.73	0.014
37	5.38	0.047	17.67	0.014
38	5.35	0.051	17.67	0.013
39	5.35	0.050	17.66	0.014
40	5.30	0.050	17.61	0.013
41	5.30	0.052	17.63	0.015
42	5.31	0.054	17.60	0.017
43	5.29	0.054	17.43	0.014
44	5.31	0.056	17.44	0.014
45	5.26	0.055	17.44	0.014

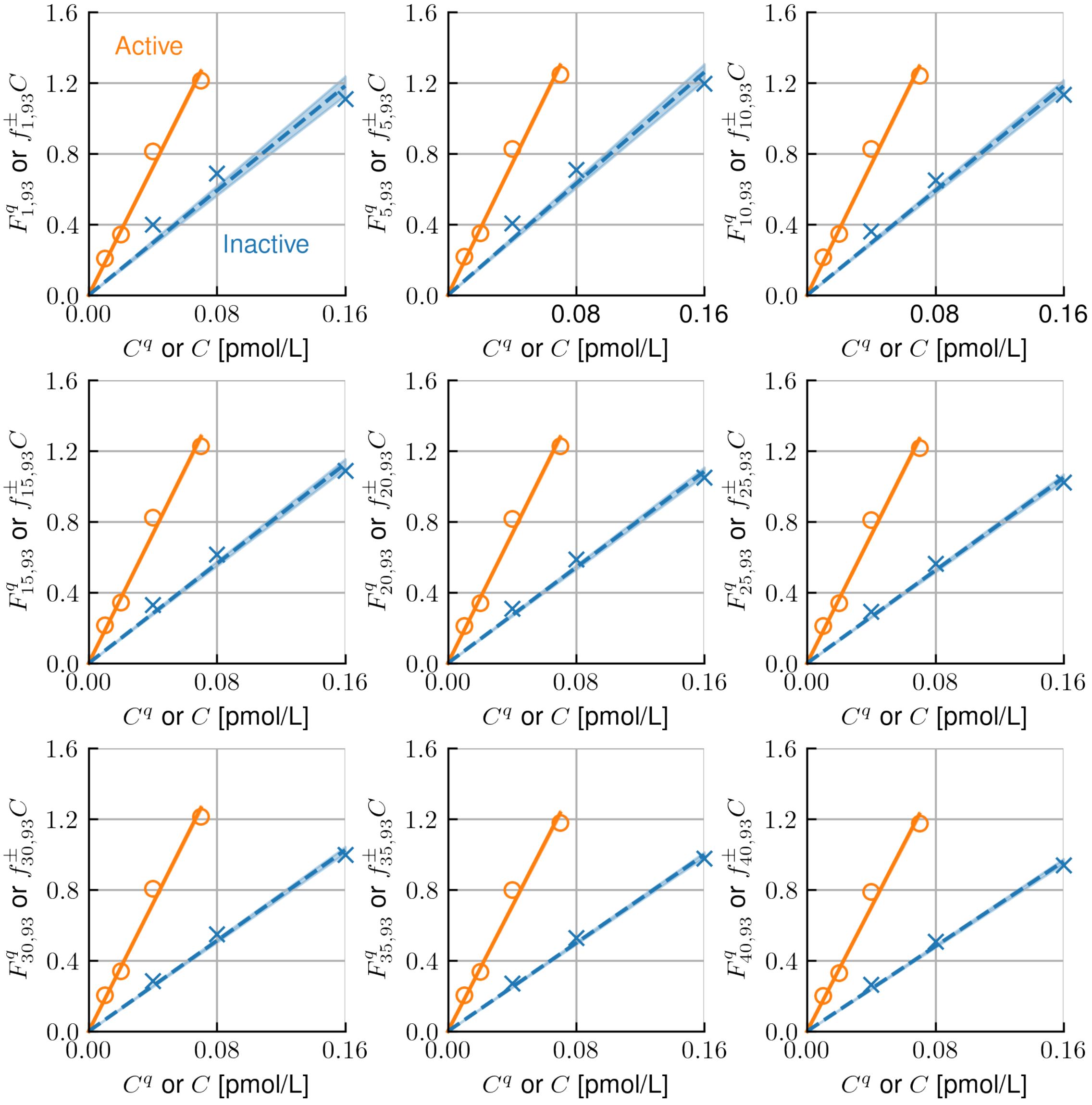

Fig. S93: As Figure S1 with well $w = 93$ (or H9).

Table S93: Molar Fluorescence Parameters for Well H9 ($w = 93$)

Cycle	Inactive		Active	
i	$f^-_{i,93}$	$\sigma^-_{i,93}$	$f^+_{i,93}$	$\sigma^+_{i,93}$
1	7.4	0.11	18.07	0.064
2	7.8	0.11	18.24	0.059
3	8.0	0.10	18.47	0.060
4	7.97	0.099	18.53	0.062
5	7.88	0.096	18.55	0.061
6	7.77	0.091	18.52	0.063
7	7.67	0.086	18.49	0.063
8	7.56	0.080	18.46	0.064
9	7.48	0.076	18.45	0.065
10	7.38	0.072	18.46	0.063
11	7.28	0.069	18.40	0.064
12	7.21	0.066	18.37	0.064
13	7.14	0.063	18.34	0.065
14	7.09	0.060	18.28	0.065
15	7.04	0.058	18.29	0.065
16	6.97	0.056	18.30	0.065
17	6.90	0.055	18.25	0.063
18	6.87	0.054	18.28	0.064
19	6.82	0.049	18.20	0.064
20	6.77	0.049	18.24	0.063
21	6.72	0.049	18.26	0.061
22	6.68	0.045	18.26	0.061
23	6.64	0.043	18.14	0.063
24	6.61	0.041	18.12	0.063
25	6.56	0.040	18.10	0.062
26	6.57	0.042	18.09	0.062
27	6.51	0.042	18.09	0.062
28	6.49	0.043	18.20	0.058
29	6.46	0.038	17.94	0.064
30	6.40	0.037	18.03	0.060
31	6.36	0.035	18.08	0.057
32	6.34	0.032	17.97	0.066
33	6.31	0.030	17.82	0.064
34	6.30	0.028	17.65	0.069
35	6.23	0.029	17.62	0.065
36	6.25	0.022	17.60	0.063
37	6.09	0.033	17.60	0.063
38	6.06	0.032	17.58	0.063
39	6.02	0.031	17.55	0.063
40	6.00	0.029	17.52	0.062
41	5.98	0.028	17.53	0.063
42	5.95	0.027	17.43	0.064
43	5.96	0.023	17.44	0.064
44	5.86	0.027	17.42	0.065
45	5.85	0.026	17.39	0.062

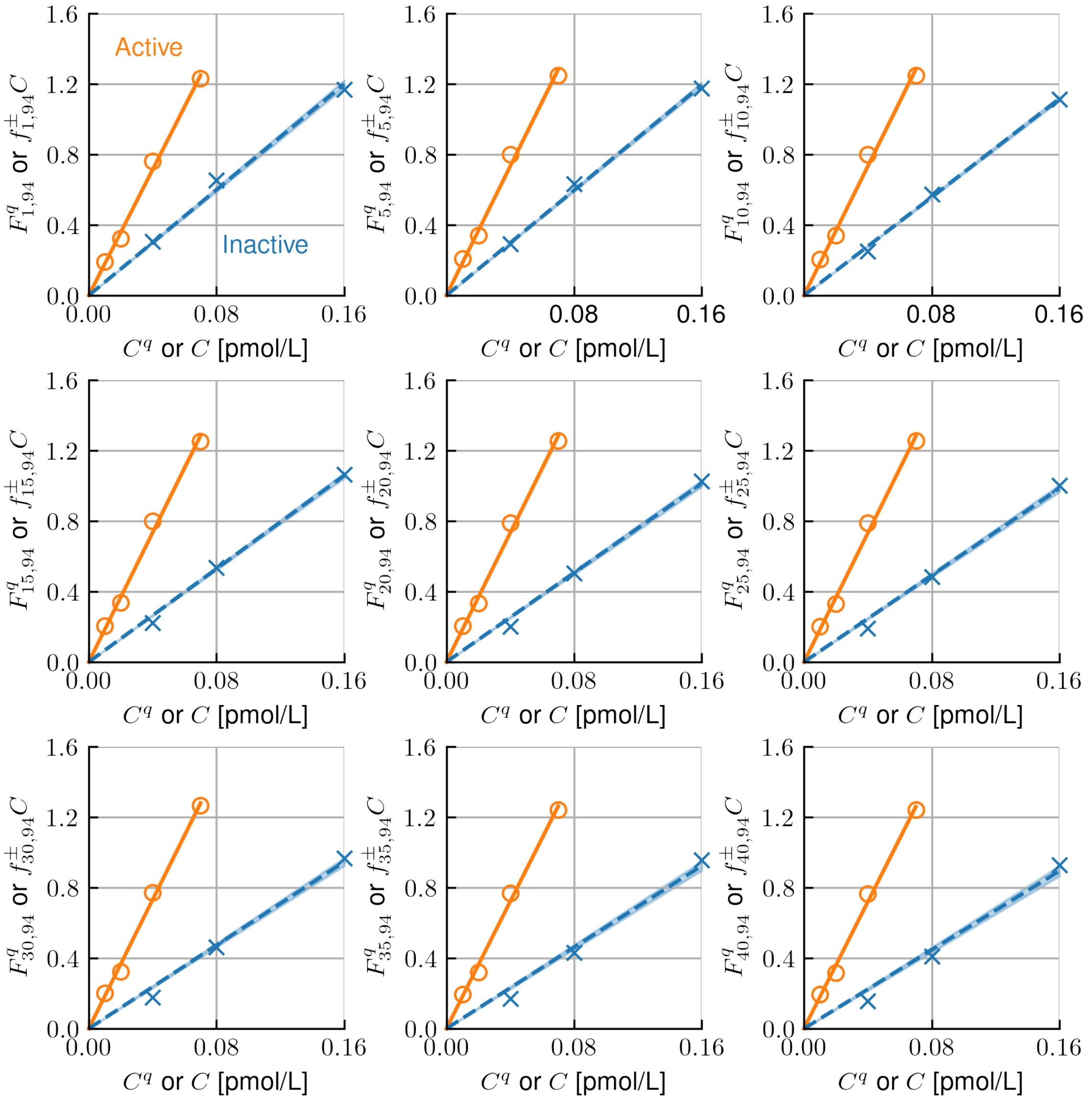

Fig. S94: As Figure S1 with well $w = 94$ (or H10).

Table S94: Molar Fluorescence Parameters for Well H10 ($w = 94$)

Cycle	Inactive		Active	
i	$f^-_{i,94}$	$\sigma^-_{i,94}$	$f^+_{i,94}$	$\sigma^+_{i,94}$
1	7.49	0.043	17.84	0.036
2	7.62	0.043	17.88	0.044
3	7.69	0.037	18.20	0.046
4	7.59	0.032	18.29	0.047
5	7.46	0.029	18.32	0.048
6	7.34	0.026	18.32	0.048
7	7.23	0.024	18.34	0.047
8	7.14	0.022	18.33	0.047
9	7.04	0.023	18.33	0.047
10	6.97	0.023	18.33	0.047
11	6.88	0.025	18.36	0.046
12	6.82	0.027	18.36	0.046
13	6.75	0.028	18.33	0.045
14	6.66	0.029	18.27	0.046
15	6.61	0.030	18.36	0.047
16	6.54	0.032	18.30	0.045
17	6.47	0.033	18.34	0.044
18	6.43	0.035	18.33	0.044
19	6.41	0.037	18.29	0.043
20	6.33	0.038	18.32	0.043
21	6.28	0.035	18.32	0.044
22	6.24	0.035	18.35	0.044
23	6.21	0.037	18.34	0.042
24	6.21	0.041	18.32	0.044
25	6.15	0.041	18.29	0.042
26	6.11	0.042	18.33	0.042
27	6.04	0.043	18.31	0.041
28	6.06	0.046	18.28	0.036
29	5.96	0.045	18.27	0.038
30	5.91	0.045	18.28	0.037
31	5.95	0.051	18.31	0.037
32	5.86	0.050	18.32	0.036
33	5.84	0.050	18.00	0.039
34	5.81	0.055	18.02	0.039
35	5.77	0.054	18.02	0.039
36	5.75	0.057	18.02	0.038
37	5.67	0.055	18.03	0.037
38	5.64	0.056	18.01	0.038
39	5.60	0.057	18.07	0.036
40	5.59	0.059	17.98	0.038
41	5.55	0.060	17.97	0.038
42	5.48	0.058	17.87	0.039
43	5.49	0.061	17.95	0.036
44	5.46	0.063	17.96	0.037
45	5.42	0.065	17.94	0.036

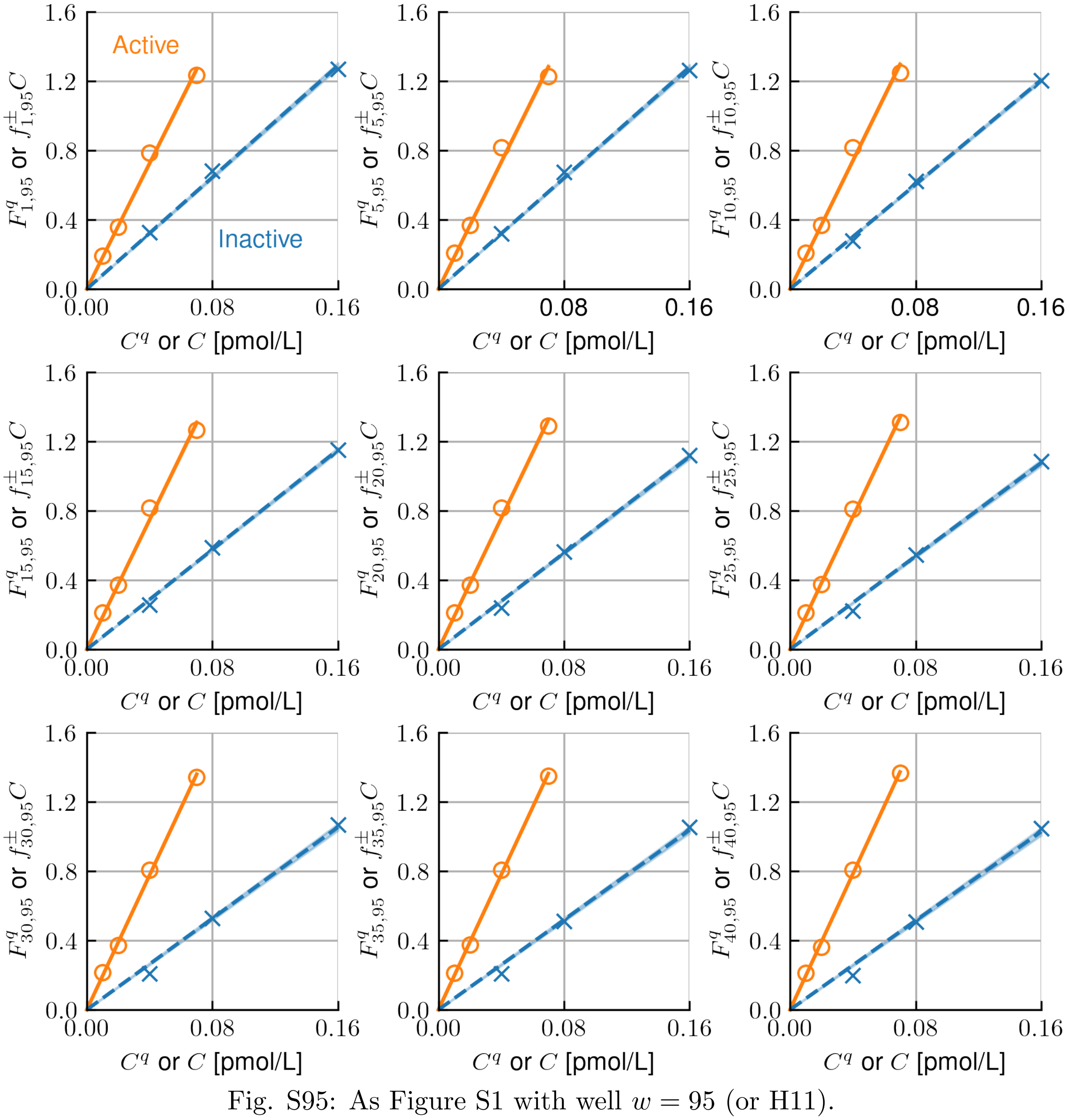

Fig. S95: As Figure S1 with well $w = 95$ (or H11).

Table S95: Molar Fluorescence Parameters for Well H11 ($w = 95$)

Cycle	Inactive		Active	
i	$f^-_{i,95}$	$\sigma^-_{i,95}$	$f^+_{i,95}$	$\sigma^+_{i,95}$
1	8.06	0.030	18.13	0.040
2	8.17	0.042	17.98	0.055
3	8.22	0.038	18.12	0.059
4	8.12	0.032	18.26	0.059
5	8.00	0.027	18.31	0.059
6	7.89	0.024	18.34	0.060
7	7.78	0.021	18.40	0.058
8	7.71	0.019	18.41	0.058
9	7.62	0.020	18.47	0.056
10	7.54	0.020	18.51	0.055
11	7.46	0.020	18.58	0.052
12	7.40	0.020	18.64	0.050
13	7.33	0.021	18.67	0.051
14	7.27	0.022	18.66	0.049
15	7.20	0.023	18.70	0.049
16	7.14	0.024	18.76	0.046
17	7.13	0.026	18.79	0.045
18	7.07	0.027	18.86	0.044
19	7.02	0.028	18.87	0.043
20	6.96	0.029	18.93	0.043
21	6.91	0.030	18.94	0.040
22	6.81	0.030	19.02	0.037
23	6.82	0.031	19.05	0.035
24	6.78	0.033	19.08	0.034
25	6.73	0.033	19.12	0.033
26	6.70	0.034	19.19	0.030
27	6.66	0.035	19.26	0.031
28	6.64	0.037	19.31	0.028
29	6.61	0.038	19.37	0.026
30	6.59	0.039	19.42	0.025
31	6.61	0.041	19.45	0.022
32	6.56	0.041	19.36	0.025
33	6.54	0.042	19.44	0.023
34	6.53	0.038	19.44	0.022
35	6.49	0.038	19.47	0.021
36	6.47	0.043	19.49	0.020
37	6.45	0.045	19.58	0.020
38	6.43	0.041	19.59	0.020
39	6.55	0.046	19.55	0.025
40	6.43	0.044	19.60	0.024
41	6.45	0.046	19.66	0.022
42	6.44	0.049	19.69	0.025
43	6.36	0.044	19.67	0.023
44	6.32	0.045	19.68	0.022
45	6.26	0.045	19.65	0.022

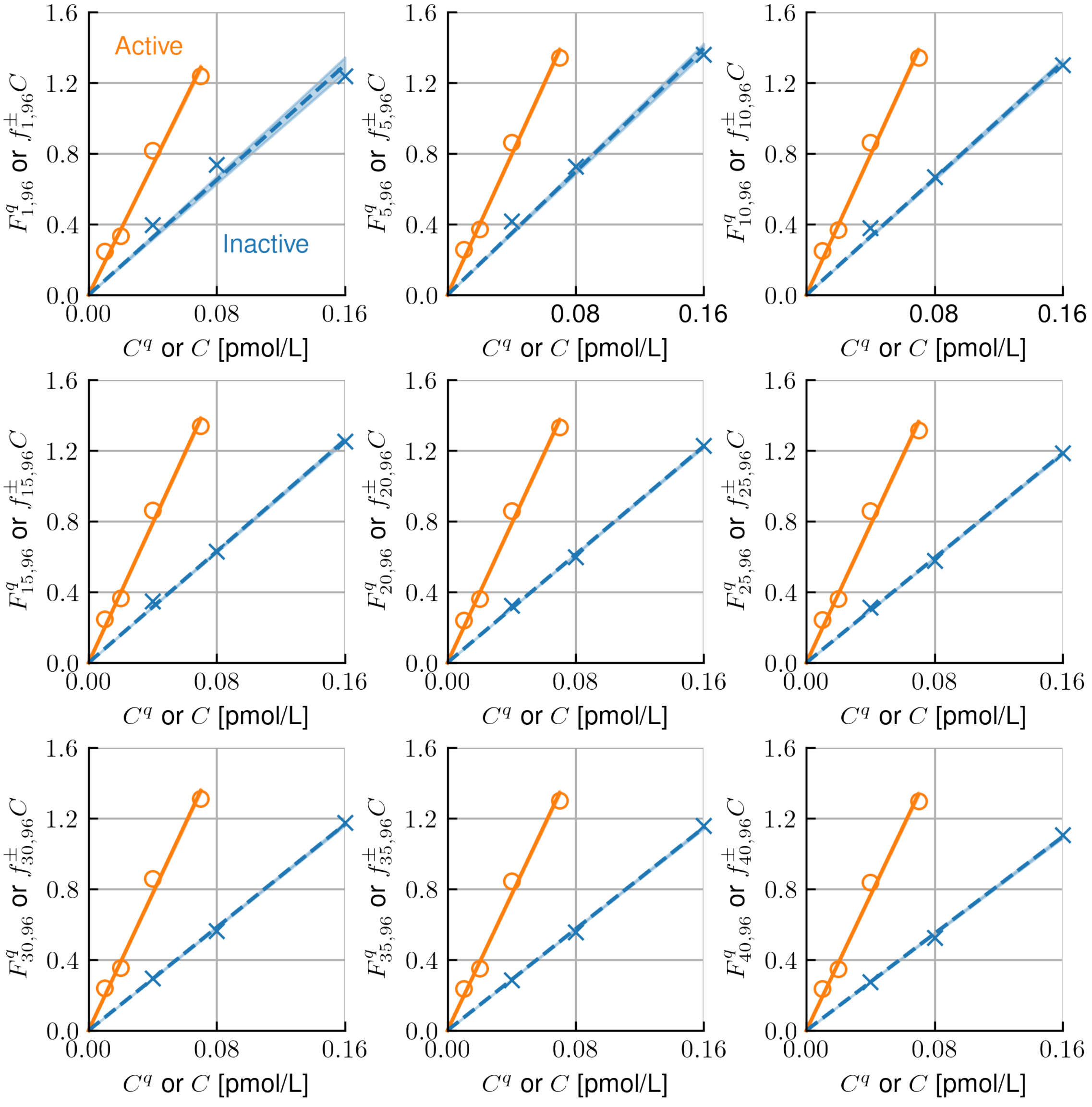

Fig. S96: As Figure S1 with well $w = 96$ (or H12).

Table S96: Molar Fluorescence Parameters for Well H12 ($w = 96$)

Cycle	Inactive		Active	
i	$f^-_{i,96}$	$\sigma^-_{i,96}$	$f^+_{i,96}$	$\sigma^+_{i,96}$
1	8.12	0.090	18.38	0.069
2	8.65	0.069	19.32	0.057
3	8.82	0.066	19.59	0.060
4	8.82	0.060	19.76	0.060
5	8.71	0.057	19.80	0.061
6	8.60	0.052	19.84	0.059
7	8.51	0.048	19.85	0.060
8	8.43	0.042	19.83	0.060
9	8.34	0.038	19.81	0.059
10	8.22	0.037	19.78	0.060
11	8.14	0.033	19.79	0.058
12	8.07	0.030	19.77	0.058
13	8.00	0.029	19.75	0.059
14	7.92	0.026	19.73	0.059
15	7.87	0.024	19.72	0.059
16	7.81	0.024	19.69	0.060
17	7.77	0.020	19.68	0.059
18	7.74	0.017	19.87	0.054
19	7.71	0.015	19.66	0.058
20	7.65	0.016	19.61	0.058
21	7.61	0.018	19.61	0.060
22	7.59	0.016	19.61	0.060
23	7.54	0.017	19.50	0.061
24	7.46	0.015	19.52	0.064
25	7.40	0.015	19.45	0.062
26	7.39	0.016	19.51	0.060
27	7.34	0.016	19.46	0.060
28	7.34	0.017	19.43	0.061
29	7.33	0.017	19.39	0.062
30	7.29	0.017	19.37	0.064
31	7.28	0.018	19.30	0.063
32	7.22	0.018	19.26	0.062
33	7.20	0.018	19.24	0.062
34	7.26	0.023	19.24	0.061
35	7.17	0.015	19.19	0.060
36	7.13	0.018	19.15	0.061
37	7.04	0.016	19.13	0.059
38	6.883	0.0091	19.10	0.058
39	6.87	0.011	19.11	0.058
40	6.85	0.018	19.09	0.058
41	6.82	0.017	19.07	0.058
42	6.78	0.017	19.07	0.058
43	6.75	0.018	19.07	0.058
44	6.74	0.020	19.11	0.058
45	6.72	0.021	19.09	0.057
